# Silylium-Ion-Initiated Disulfidation of Unactivated Alkenes with Disulfides: Methodology and Isolation of the Sulfenium-Ion Carriers

**DOI:** 10.1021/acs.joc.6c01661

**Published:** 2026-08-07

**Authors:** Biping Xu, Xiaojie Liu, Dáiríne M. Morgan, Elisabeth Irran, Martin Oestreich

**Affiliations:** Institut für Chemie, 199915 Technische Universität Berlin, Straße des 17. Juni 115, 10623 Berlin, Germany

## Abstract

A metal-free and operationally simple method for the two-component disulfidation of unactivated alkenes enabled by silylium-ion initiation is reported. The protocol promotes intermolecular coupling of alkenes with readily available disulfides under mild conditions, providing access to vicinal dithioethers with broad substrate scope and excellent functional-group tolerance. More than 30 combinations of alkenes and disulfides demonstrate the generality and practicality of the transformation. Mechanistically, the highly electrophilic silylium ion is proposed to activate the disulfide in the form of a sulfonium ion, thereby turning it into a sulfenium-ion carrier. Both an example of an initially formed sulfonium ion [Et_3_Si­(EtSSEt)]­[HCB_11_H_5_Br_6_] and a subsequently generated electrophile [MeS­(MeSSMe)]­[HCB_11_H_5_Br_6_] were isolated and characterized by X-ray diffraction. Transfer of the sulfenium-ion electrophile from these cations to the C–C double bond, followed by attack of another disulfide molecule as a sulfur nucleophile, allows for the formation of two C–S bonds without the need for a transition-metal catalyst or an external oxidant.

## Graphical abstract

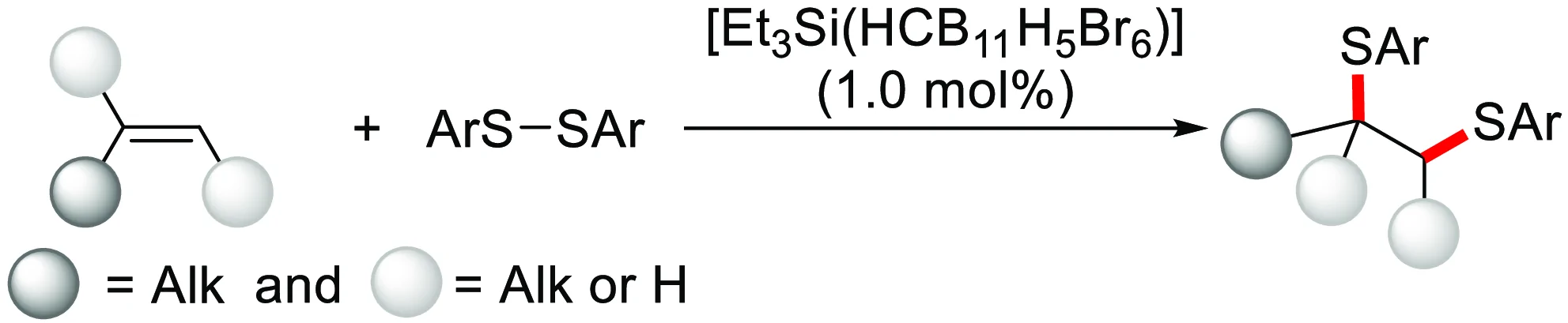

## Introduction

Vicinal dithioethers represent a synthetically valuable subclass of sulfur-containing molecules with broad applications across modern chemistry. These motifs do not only serve as bidentate ligands in transition-metal catalysis^1^ but are also biosensors for tracing and quantifying vicinal dithiol-containing proteins in physiological settings.^2^ In addition, their high sulfur content renders them indispensable building blocks for the construction of functional sulfur-rich materials.^3^ Owing to these properties and diverse applications, the development of practical and efficient methods for the synthesis of vicinal dithioethers has continued to attract attention from synthetic chemists.

Traditionally, vicinal dithioethers are prepared through substitution reactions of prefunctionalized vicinal dithiol-derived nucleophilic motifs,^4^ dihalides,^5^ or related substrates^6^ (Scheme 1A, paths a and b). However, such approaches often suffer from limited substrate availability, multistep synthetic sequences as well as using foul-smelling thiols. In 2009, the Hammond group reported an elegant and environmentally benign synthesis of vicinal dithioethers from alkynes and thiols or thiophenols in water under mild conditions (Scheme 1A, path c).^7^ While this method constitutes a valuable synthetic route, alkenes are inherently preferable substrates for vicinal dithioether synthesis over alkynes, featuring higher abundance, lower cost, and broader structural variation.^8^ Recent advances have provided a variety of thiolation methods for alkynes^9^ and alkenes,^10^ greatly expanding the synthetic toolbox for C–S bond formation. Nevertheless, further improvements in catalytic efficiency are still desirable for alkene disulfidation, as many reported systems, including metal-complex^11^ and metal-free^12^ approaches, rely on relatively high catalyst loadings to achieve effective conversion (Scheme 1A, path d). We therefore asked ourselves whether silylium-ion catalysis could provide an alternative approach, building on the recent success with the silylium-ion-initiated disulfidation of alkynes (Scheme 1B).^13,14^ We herein show that unactivated alkenes (but not styrenes) engage in the same reaction with disulfides, providing a straightforward and metal-free approach to vicinal dithioethers (Scheme 1C).

**Scheme 1:**
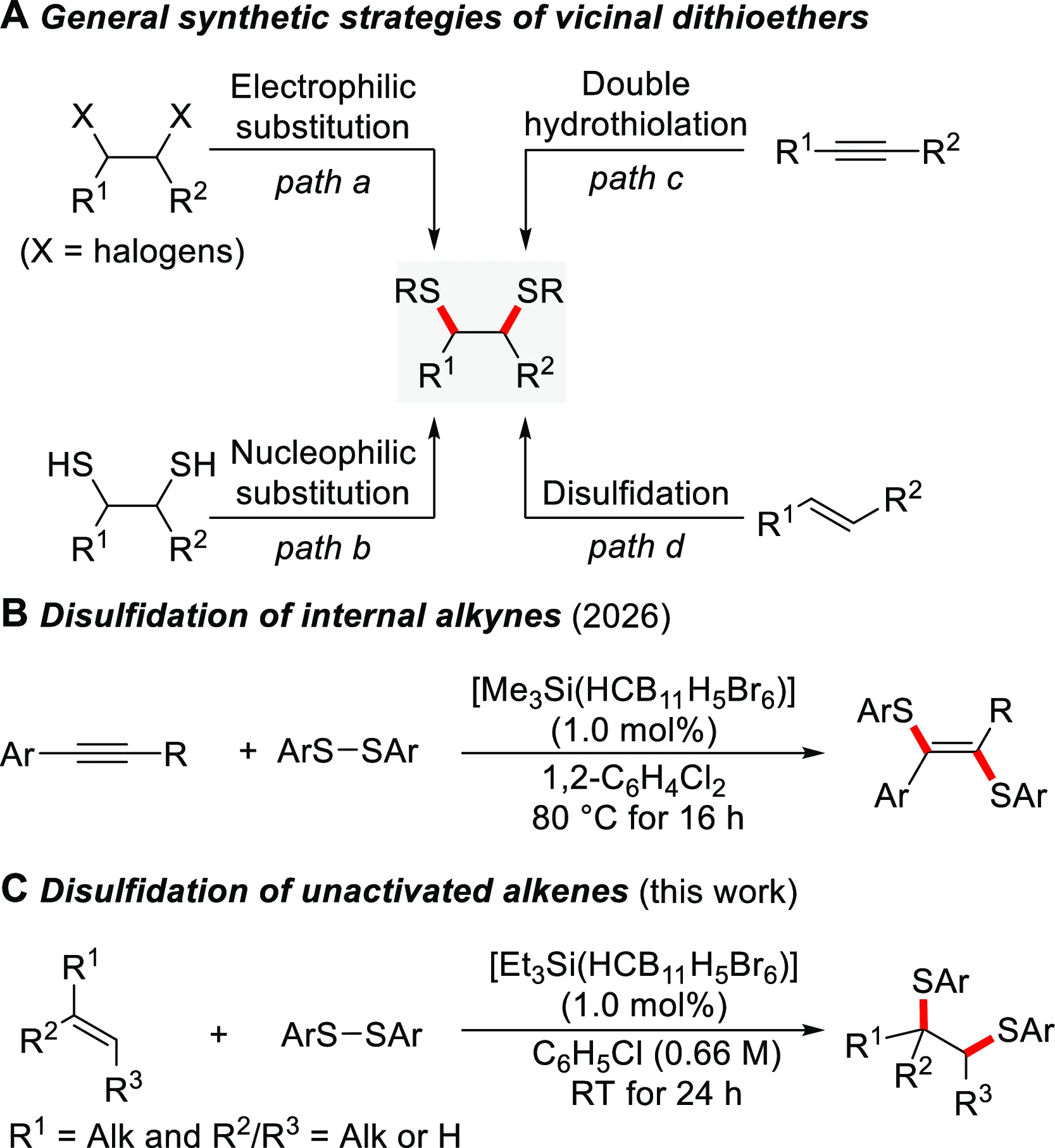
Synthesis of Vicinal Dithioethers

## Results and Discussion

We began the optimization of the reaction conditions using oct-1-ene (**1a**) and bis­(4-methylphenyl) disulfide (**2a**) as model substrates in chlorobenzene as the solvent (Table 1). The desired vicinal dithioether **3aa** was observed in nearly quantitative yield when [Et_3_Si­(HCB_11_H_5_Br_6_)] was employed as the initiator (entry 1). Further investigation of different cations with the same counterion revealed that the trimethylsilylium ion and the trityl cation were also competent promoters of this transformation, albeit in slightly diminished yields (entries 2 and 3). Conversely, the hydronium ion [Et_3_Si­(HSiEt_3_)]­[B­(C_6_F_5_)_4_] led to a substantial decrease in yield, likely owing to the reduced Lewis acidity and weaker ion-pair stabilization relative to the carborane system (entry 4). Neutral tris­(pentafluorophenyl)­borane and AlCl_3_ promoted the reaction only with moderate efficiency (entries 5 and 6). In the absence of catalytic initiation, no product was observed, and all starting materials were recovered intact, underscoring the need of a Lewis acid to initiate the disulfidation process.

**Table 1:**
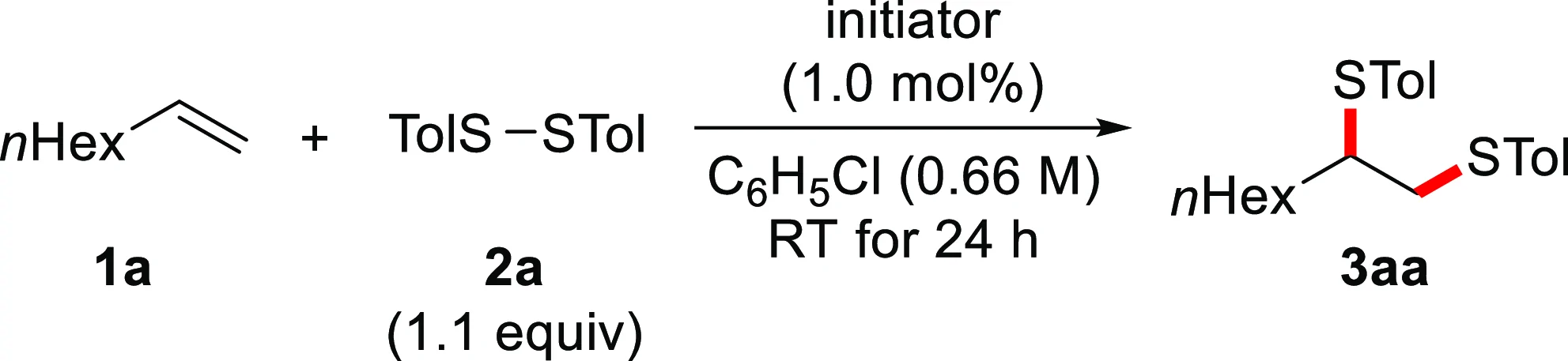
Optimization of the Reaction Conditions^a^

entry	initiator	yield (%)^b^
1	[Et_3_Si(HCB_11_H_5_Br_6_)]	99 (97)^c^
2	[Me_3_Si(HCB_11_H_5_Br_6_)]	94
3	[Ph_3_C][HCB_11_H_5_Br_6_]	91
4	[Et_3_Si(HSiEt_3_)][B(C_6_F_5_)_4_)]	75
5	B(C_6_F_5_)_3_	71
6	AlCl_3_	70
7		n. d.

a Reaction conditions: The indicated initiator (2.0 μmol, 1.0 mol %) and bis­(4-methylphenyl) disulfide (**2a**, 0.22 mmol, 1.1 equiv) were dissolved in chlorobenzene (0.30 mL) and oct-1-ene (**1a**, 0.20 mmol) was subsequently added. The reaction was maintained at RT for 24 h.

b Yield was determined by GLC analysis with dodecane as an internal standard.

c Yield in parentheses is isolated yield after flash chromatography on silica gel.

With the optimized conditions established, we next explored the scope of unactivated alkenes in this disulfidation reaction (Scheme 2). A broad range of α-olefins underwent smooth transformation to furnish the corresponding vicinal dithioethers in good to excellent yields (**3aa**–**d**′**a**). Notably, even sterically demanding substrates bearing a *tert*-butyl group adjacent to the alkene moiety reacted efficiently under the standard conditions (**3ea**). Furthermore, bisalkene substrate **1f**, containing two terminal alkene units, successfully delivered the 2-fold disulfidation product **3fa** in good yield when treated with twice the amount of catalyst and bis­(4-methylphenyl) disulfide, demonstrating the robustness of the catalytic system. Haloalkyl-substituted α-olefins bearing F, Cl, or Br substituents all participated smoothly in the reaction with the carbon–halogen bond intact (**3ga**–**ia**), thereby providing valuable synthetic handles for downstream derivatization. Functional groups such as trimethylsilyl and nitrile substituents were also well tolerated, delivering the corresponding products **3ja** and **3ka** in 81% and 96% yield, respectively. Although ester-substituted alkenes proved less reactive, the desired vicinal dithioether **3la** could still be isolated in 23% yield.

**Scheme 2:**
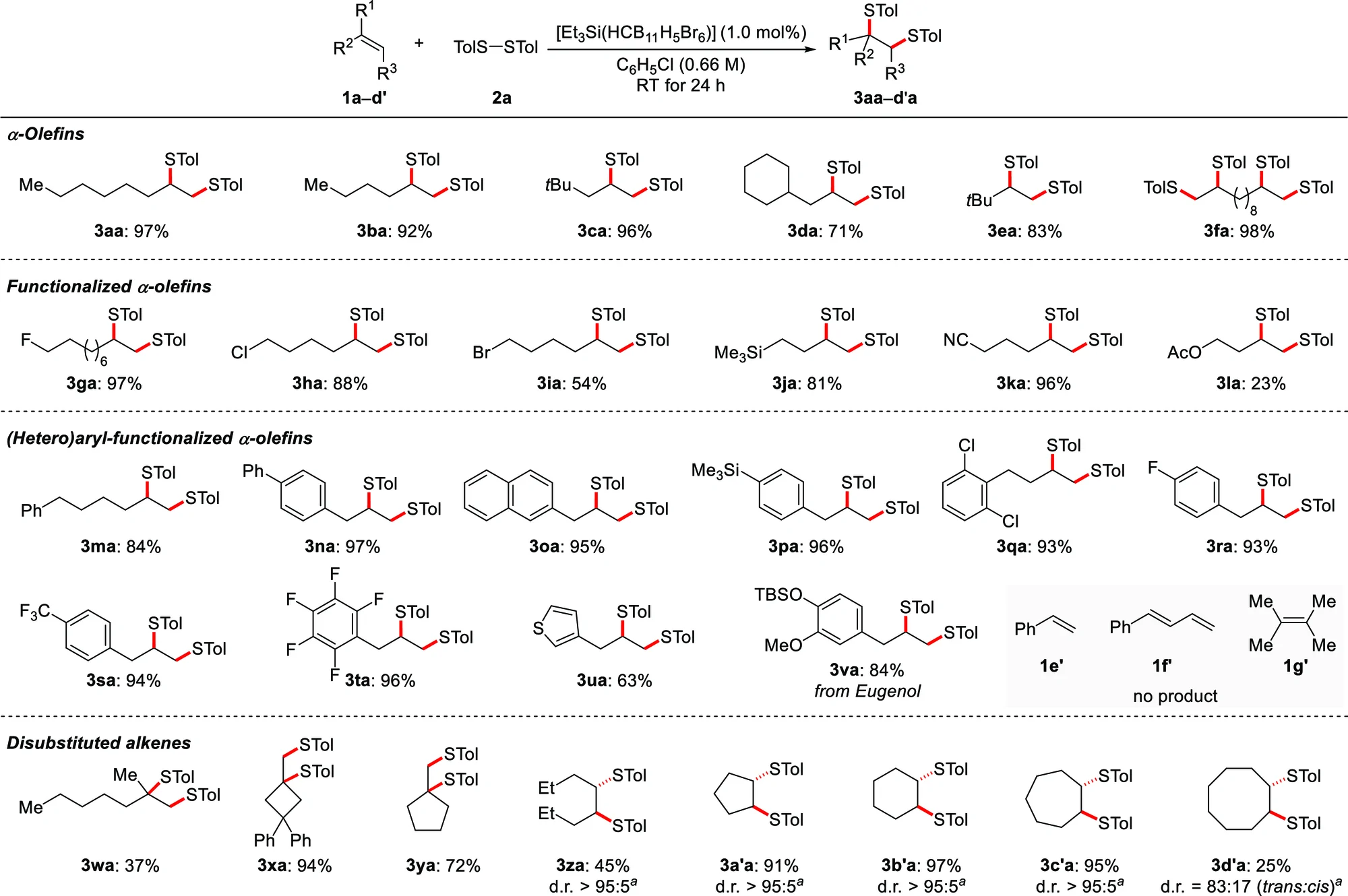
Scope I: Variation of the Unactivated Alkene in the Disulfidation with Bis­(4-methylphenyl) Disulfide^a^

a The diastereomeric ratio was estimated by ^1^H NMR analysis.

Subsequently, we proceeded to assess diverse α-olefins bearing aromatic rings. In every instance, the anticipated products **3ma**−**oa** were furnished in satisfactory yields. In particular, sensitive trimethylsilyl, chloro and fluoro substituents stayed intact and did not undergo any structural alteration under the standard reaction conditions (**3pa**–**ta**). A thiophene-containing substrate readily participated in the disulfidation reaction to afford product **3ua** in good yield. Notably, an electron-rich eugenol derivative bearing multiple oxygen-containing functionalities proved to be a competent substrate, furnishing the desired vicinal dithioether **3va** in good yield without potentially competing functionalization of the aromatic ring.

Encouraged by these results, we further extended the methodology to a variety of disubstituted alkenes. A 1,1-disubstituted alkene afforded the corresponding product **3wa** in moderate yield whereas exocyclic alkenes attached to four- and five-membered rings reacted considerably more efficiently, delivering the desired vicinal dithioethers **3xa** and **3ya** in 94% and 72% yield, respectively. Oct-4-ene also proved to be a viable substrate, albeit with only moderate efficiency (**3za**). Cycloalkenes with normal ring sizes underwent smooth disulfidation in good to excellent yields (**3a**′**a**–**c**′**a**) while the medium-sized cyclooctene exhibited significantly diminished reactivity, affording only 25% yield as a mixture of the *trans* and *cis* diastereomers with an 83:17 ratio (**3d**′**a**). Despite the broad alkene substrate scope, aryl-substituted alkene **1e**′ (styrene) and 1,3-diene **1f**′ as well as tetrasubstituted alkene **1g**′ failed to yield the corresponding products under the standard conditions; the alkene starting materials were fully recovered (gray box). We speculate that this outcome arises from the reversibility of the C–S bond formation under the strongly Lewis acidic reaction conditions when stabilized carbocations can be formed.^11b^ Also, steric hindrance might thwart the initial addition of the sulfenium ion in the case of highly substituted alkenes.

Following the evaluation of the alkene scope, we next examined the compatibility of various disulfides (Scheme 3). A range of diaryl disulfides bearing diverse substituents on the aromatic ring proved to be effective coupling partners, delivering the desired products **3ab**−**ad** and **3af** in high yields. Bis­(4-bromophenyl) disulfide (**2e**) represented a notable exception, exhibiting reduced reactivity under the standard conditions (**3ae**). This behavior may arise from competitive interactions between the bromine substituents and the electrophilic cationic species involved in the catalysis. Moreover, dialkyl disulfides did also act as competent substrates to afford the vicinal dithioether **3qg** and **3vg** in good yield at an elevated temperature of 80 °C. In contrast, di-*tert*-butyl disulfide (**2i**) showed no reactivity with the starting materials being recovered completely;: this is presumably due to the steric congestion around the sulfur atoms that prevents effective interaction with the silylium- and sulfenium-ion electrophiles, respectively. Notably, despite sulfur and selenium belonging to the same main group, no conversion was observed with the corresponding diphenyl diselenide (**2j**). This lack of reactivity was attributed to the lower Lewis basicity of selenium.

**Scheme 3:**
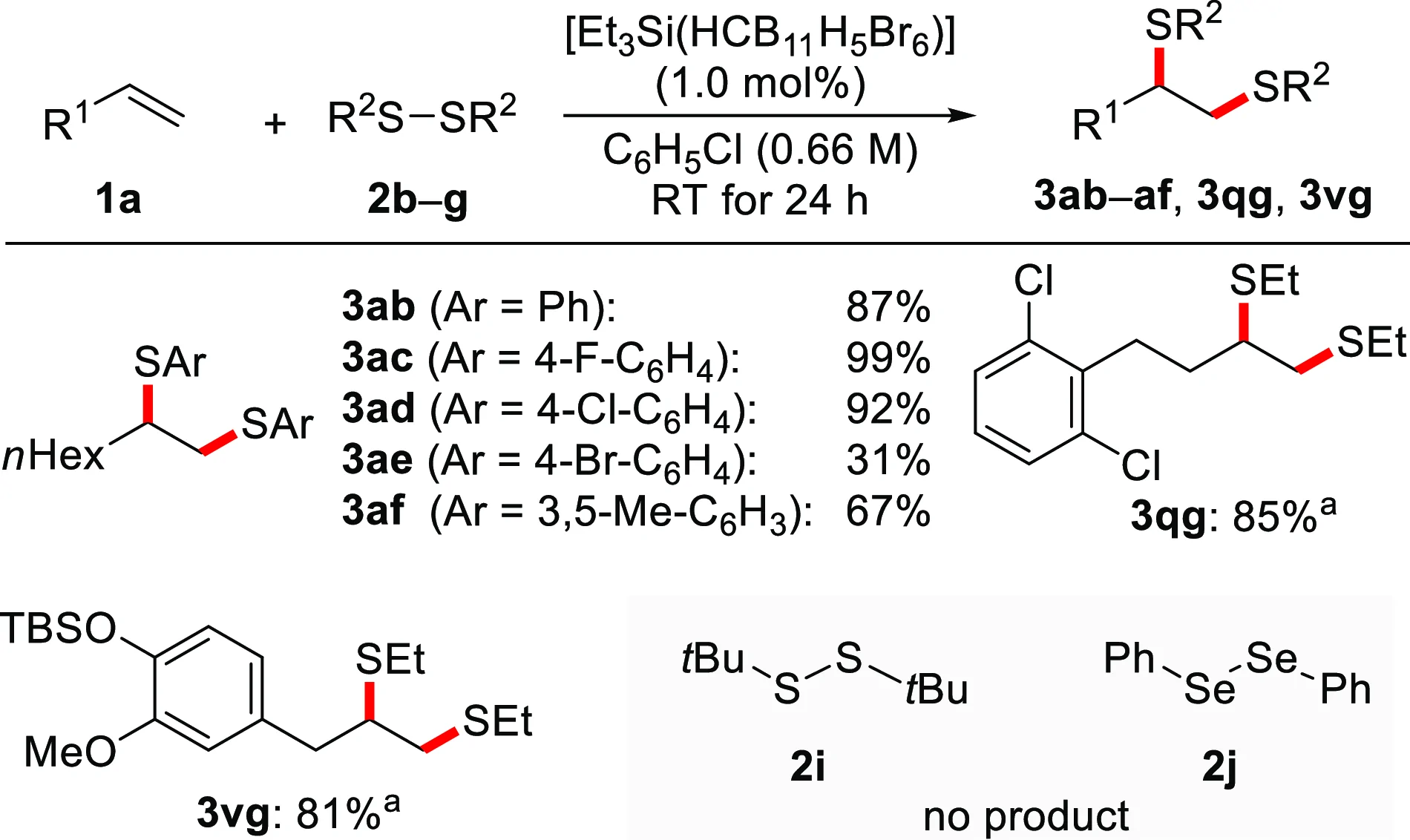
Scope II: Variation of the Disulfide in the Disulfidation of Selected Alkenes^a^

a The reaction was conducted at 80 °C instead of RT.

To further demonstrate the practicality and scalability of this methodology, the reaction was performed on a 2.0 mmol scale under solvent-free (neat) conditions (Scheme 4). The desired vicinal dithioether product **3ba** was obtained in 90% isolated yield, highlighting the operational simplicity and robustness of the protocol. Given the broad synthetic utility of vicinal disulfones in organic synthesis,^15^ the resulting vicinal dithioether was readily oxidized to the corresponding vicinal disulfone **4** in nearly quantitative yield.

**Scheme 4:**
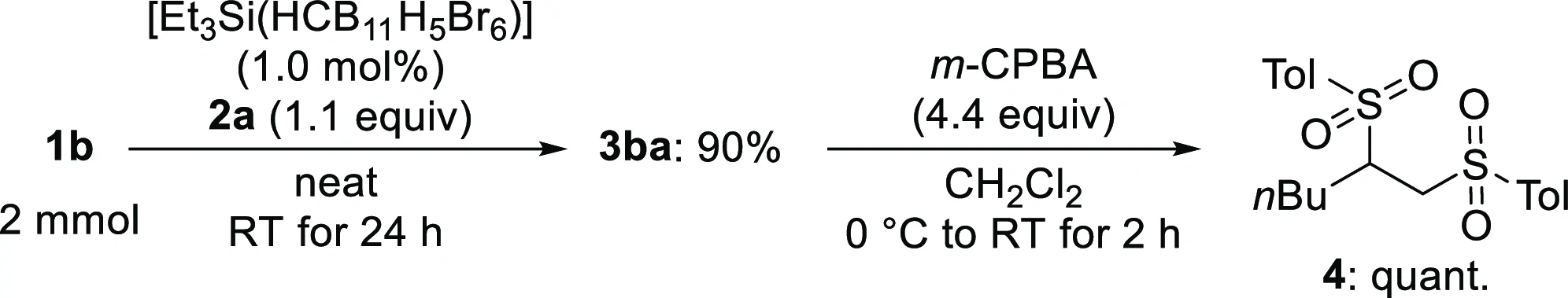
Scale-Up Reaction and Exhaustive Oxidation of a Vicinal Dithioether

Our attempts to obtain the molecular structure of [Et_3_Si­(ArSSAr)]^+^ have been fruitless so far but we obtained the molecular structure of [Et_3_Si­(EtSSEt)]­[HCB_11_H_5_Br_6_] (**5g**) derived from more Lewis basic diethyl disulfide **2g** (Scheme 5; left). We assume that **5g** does not engage in the alkene disulfidation at room temperature (see Scheme 3) because such Lewis pairs of dialkyl disulfides are likely more stable than those of diaryl disulfides.^14,16^ Upon further standing, sulfenium-ion scrambling leads to the formation of the all-sulfur sulfonium ion **6**, and we were able to obtain single crystals for the equilibration with dimethyl disulfide **2h** (Scheme 5; right); the molecular structure of [MeS­(MeSSMe)]­[HCB_11_H_5_Br_6_] (**6h**) is in agreement with previous reports.^17^

**Scheme 5:**
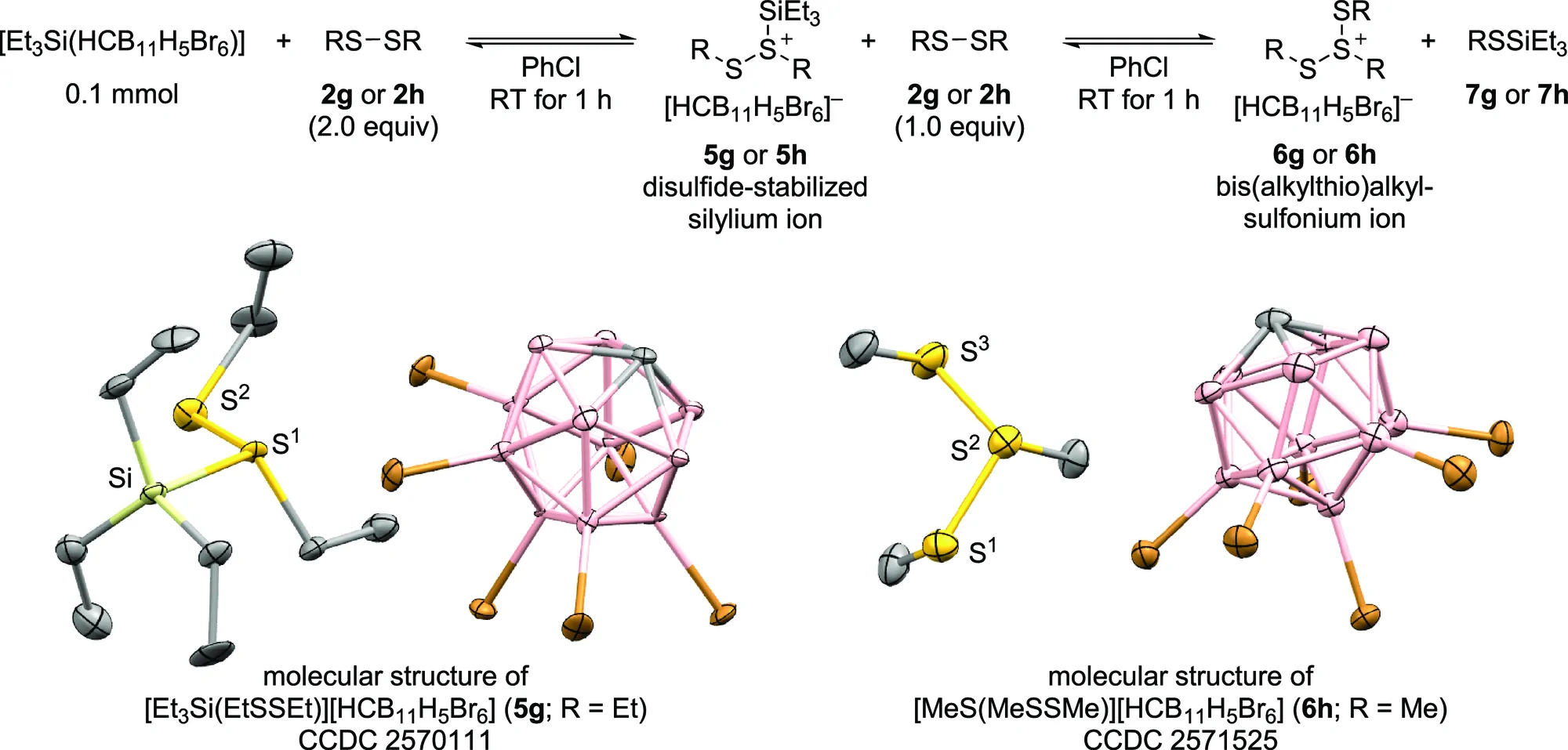
Formation and Isolation of the Sulfenium-Ion Carriers

Based on these results and related silylium ion-initiated transformations,^13^ a plausible reaction mechanism is proposed (Scheme 6). The catalytic cycle is initiated by coordination of the silylium ion to the disulfide **2**, generating the sulfonium ion Et_3_Si­(R^2^SSR^2^)^+^ (**5**). This potential sulfur electrophile undergoes sulfenium-ion exchange with another disulfide molecule to afford intermediate **6** as another sulfenium-ion carrier along with thiosilane byproduct **7**. The isolation of both **5** and **6** are conclusive evidence of their intermediacy in these and related^10^ disulfidation reactions. The all-sulfur sulfonium ion **6** then engages in an electrophilic addition to alkene **1** to furnish episulfonium-ion intermediate **8**. Nucleophilic capture of **8** by another molecule of disulfide **2** then affords intermediate **9**, which subsequently collapses to release the vicinal dithioether product **3**. The thus-released sulfenium ion is transferred to the next alkene molecule **1**, thereby maintaining catalytic turnover without further involvement of the silylium-ion promoter.

**Scheme 6:**
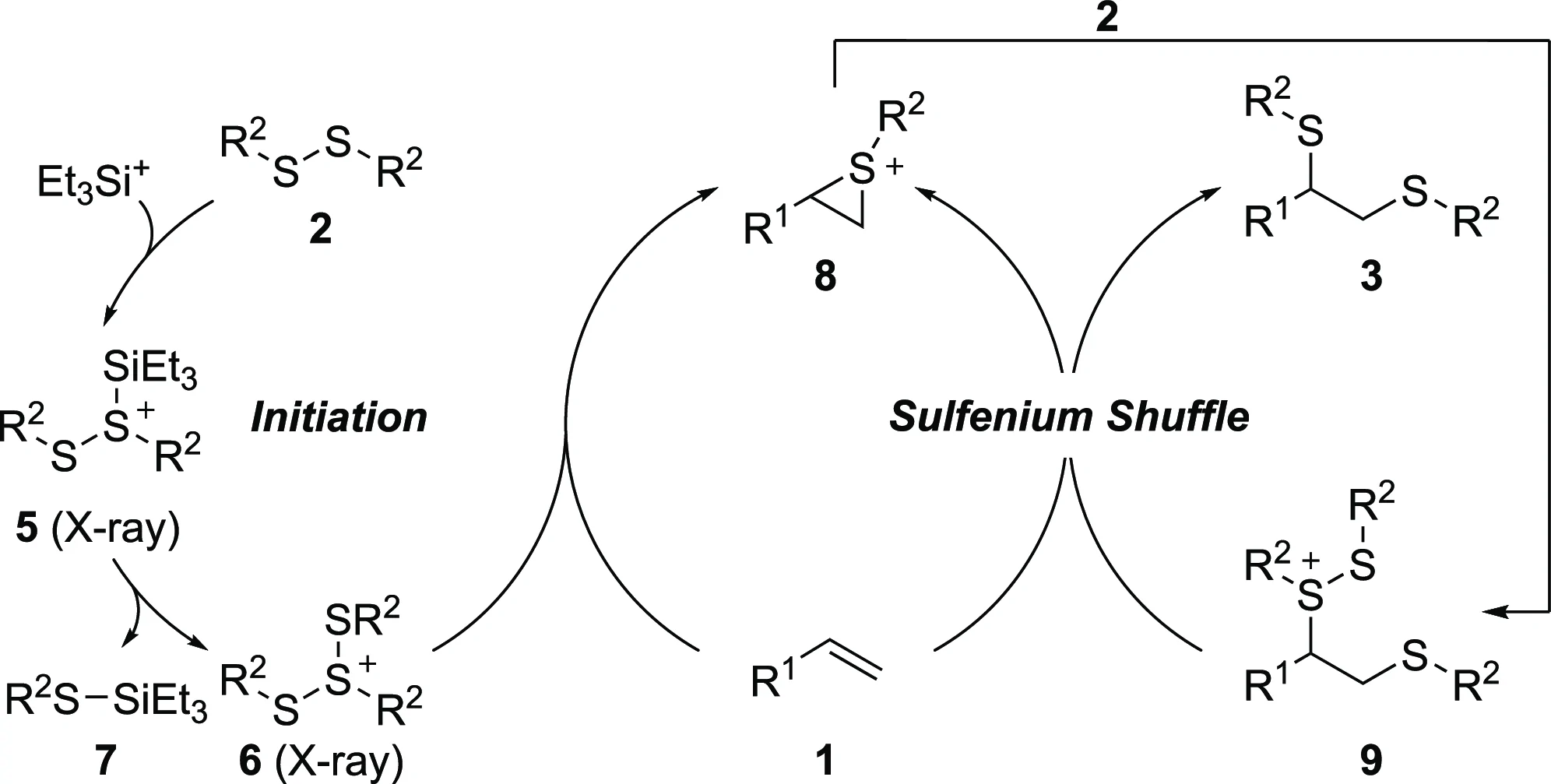
Proposed Reaction Mechanism^a^

a The counteranion [HCB_11_H_5_Br_6_]^−^ has been omitted for clarity.

## Conclusions

In conclusion, we have developed a practical and efficient silylium ion-initiated disulfidation of unactivated alkenes with disulfides, providing direct access to synthetically valuable vicinal dithioethers under metal-free conditions. The protocol features mild reaction conditions, broad substrate scope, excellent functional-group tolerance, operational simplicity, and is applicable to a diverse range of terminal, internal, and cyclic alkenes. The successful isolation and crystallographic characterization of the sulfonium-ion intermediates and sulfenium-ion carriers are rare direct experimental evidence for the involvement of such catalytic manifolds. We hope that this is a starting point for the design of more practical catalytic systems based on the same activation mode.

## Supplementary Material



## Data Availability

The data underlying this study are available in the published article and its Supporting Information.

## Notes

Experimental procedures, including the synthesis of starting materials and characterization data, and NMR spectral data for all new compounds (PDF)
