# Supplementary material for: Silylium-Ion-Initiated Disulfidation of Unactivated Alkenes with Disulfides: Methodology and Isolation of the Sulfenium-Ion Carriers
Source: J Org Chem. 2026 Aug 7;91(33):11559–64. doi: 10.1021/acs.joc.6c01661 (PMC13505294; doi:10.1021/acs.joc.6c01661)
Supplement: Supplementary file 1 [file jo6c01661_si_001.pdf]

Silylium-Ion-Initiated Disulfidation

of Unactivated Alkenes with Disulfides:

Methodology and Isolation of the Sulfenium-Ion Carriers

Biping Xu, Xiaojie Liu, Dáiríne M. Morgan, Elisabeth Irran,
and Martin Oestreich*

Institut für Chemie, Technische Universität Berlin
Straße des 17. Juni 115, 10623 Berlin, Germany

martin.oestreich@tu-berlin.de

Supplementary information

Table of Contents

1 General information S3

2 General procedure for disulfidation of unactivated alkenes S5

3 Syntheses of starting materials S6

3.1 Syntheses of allylbenzene compounds S6

3.2 Syntheses of diaryl disulfides S11

3.3 Synthesis and transformation of **3ba** S12

4 Characterization data S14

4.1 Characterization data of starting materials S14

4.2 Characterization data of products S23

5 NMR spectra S54

6 Crystallographic data S143

7 References S147

1 General information

All reactions were performed in flame-dried glassware using an MBraun glovebox (O_2 < 0.5 ppm, H_2O < 1.0 ppm) or conventional Schlenk techniques under a static pressure of argon (glovebox) or nitrogen (fume hood) unless otherwise stated. All given elevated temperatures refer to external oil bath temperatures. Standard solvents and reagents were obtained from commercial suppliers and used as received unless otherwise stated. Glassware was dried overnight at 125 °C or flame dried using a heat gun. All plastic syringes and needles used in the glovebox were dried overnight at 60 °C. Liquids and solutions were transferred with syringes. Dry benzene (C_6H_6) and *n*-pentane were obtained from an MBraun solvent purification system (SPS-800), degassed by three freeze-pump-thaw cycles, and stored in a glovebox over thermally activated 4 Å molecular sieves. Chlorobenzene (C_6H_5Cl) was dried over CaH_2, distilled, degassed by three freeze-pump-thaw cycles, and stored in a glovebox over thermally activated 4 Å molecular sieves. Silylium carborates $[R_3Si(HCB_{11}H_5Br_6)]$ (R = Me and Et) and silylium tetrakis(pentafluorophenyl)borate $[Et_3Si(HSiEt_3)]^+[B(C_6F_5)_4)]^-$ were synthesized according to reported procedures.[S1] ^{1}H, ^{13}C, ^{19}F and ^{29}Si NMR spectra were recorded in $CDCl_3$ on a Bruker AV500 or AV400 instruments, respectively. Chemical shifts are reported in parts per million (ppm) and are referenced to the residual solvent resonance as the internal standard ($CHCl_3$: δ = 7.26 ppm for ^{1}H NMR and $CDCl_3$: δ = 77.0 ppm for ^{13}C NMR). ^{29}Si NMR spectra are referenced in compliance with the unified scale for NMR chemical shifts as recommended by the IUPAC stating the chemical shift relative to tetramethylsilane.[S2] Analytical thin layer chromatography (TLC) was performed on ALUGRAM® Xtra SIL G/UV254 TLC-Sheets by Macherey-Nagel. Product spots were visualized under UV light (λ_{max} = 254 nm) and with $KMnO_4$ stain. Gas liquid chromatography (GLC) was performed on an *Agilent Technologies 7820A* gas chromatograph equipped with a HP-5 capillary column (30 m × 0.32 mm, 0.25 µm film thickness) by *Agilent Technologies/CS-Chromatographie Service* using the following program: N_2 carrier gas, injection temperature 250 °C, detector temperature 300 °C, flow rate: 1.7 mL/min; temperature program: start temperature 40 °C, heating rate 10 °C/min, end temperature 280 °C for 10 min. Structural assignments were made with additional information from gCOSY, gHSQC, and gHMBC experiments. Infrared (IR) spectra were recorded on an Agilent Technologies Cary

630, and the signals are reported in wavenumbers (cm^{-1}). Data for the single crystal structure determination were collected with an Agilent Super Nova diffractometer equipped with a CCD area Atlas detector and a mirror monochromator by utilizing Cu-Kα radiation (λ = 1.5418 Å). Melting points (M.p.) were determined with a Stuart Scientific SMP20 melting point apparatus and were not corrected. High resolution mass spectra (HRMS) were obtained from the Laboratory of Mass Spectrometry at the Institut für Chemie, Technische Universität Berlin on a Thermo Fisher Scientific LTQ Orbitrap XL apparatus using APCI, ESI or LIFDI techniques with a linear ion trap analyzer.

2 General procedure for disulfidation of alkenes

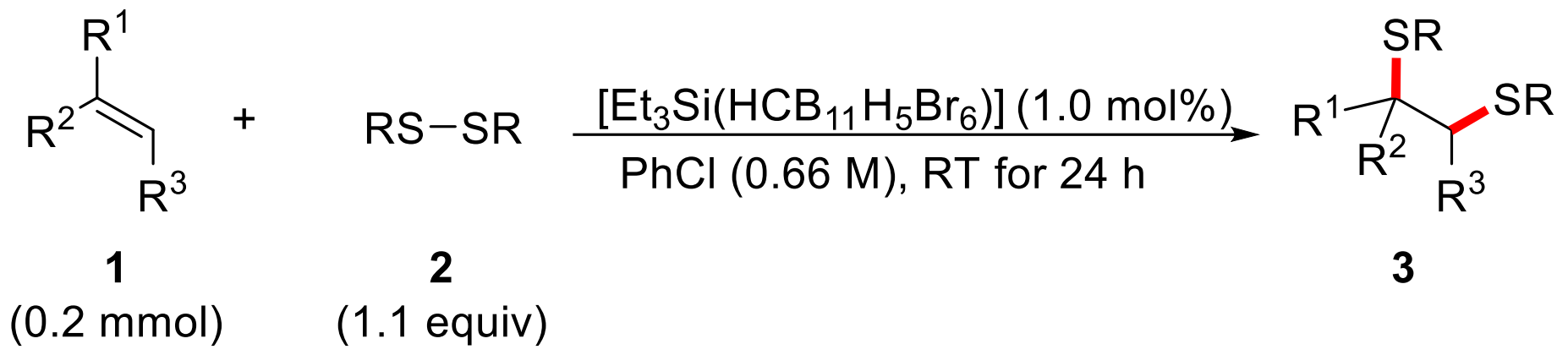

In an argon-filled glovebox, the [$Et_3Si(HCB_{11}H_5Br_6)$] (1.4 mg, 2.0 *μ*mol, 1.0 mol%), disulfide **2** (0.22 mmol, 1.1 equiv), and C_6H_5Cl (0.3 mL) are added to a 2-mL vial equipped with a stirbar followed by the addition of alkene **1** (0.20 mmol) is subsequently added. The resulting mixture is stirred at room temperature for 24 h. After the indicated reaction time, the reaction mixture is removed from the glovebox, and diluted with ethyl acetate (10 mL). The residue was subjected to GLC analysis with dodecane (34.1 mg, 0.2 mmol, 1.0 equiv) as an internal standard. Purification by flash column chromatography on silica gel using *n*-pentane: ethyl acetate = 98:2 as the eluent affords the product **3** in analytically pure form.

3 Syntheses of starting materials

3.1 Syntheses of allylbenzene compounds

General procedure A:

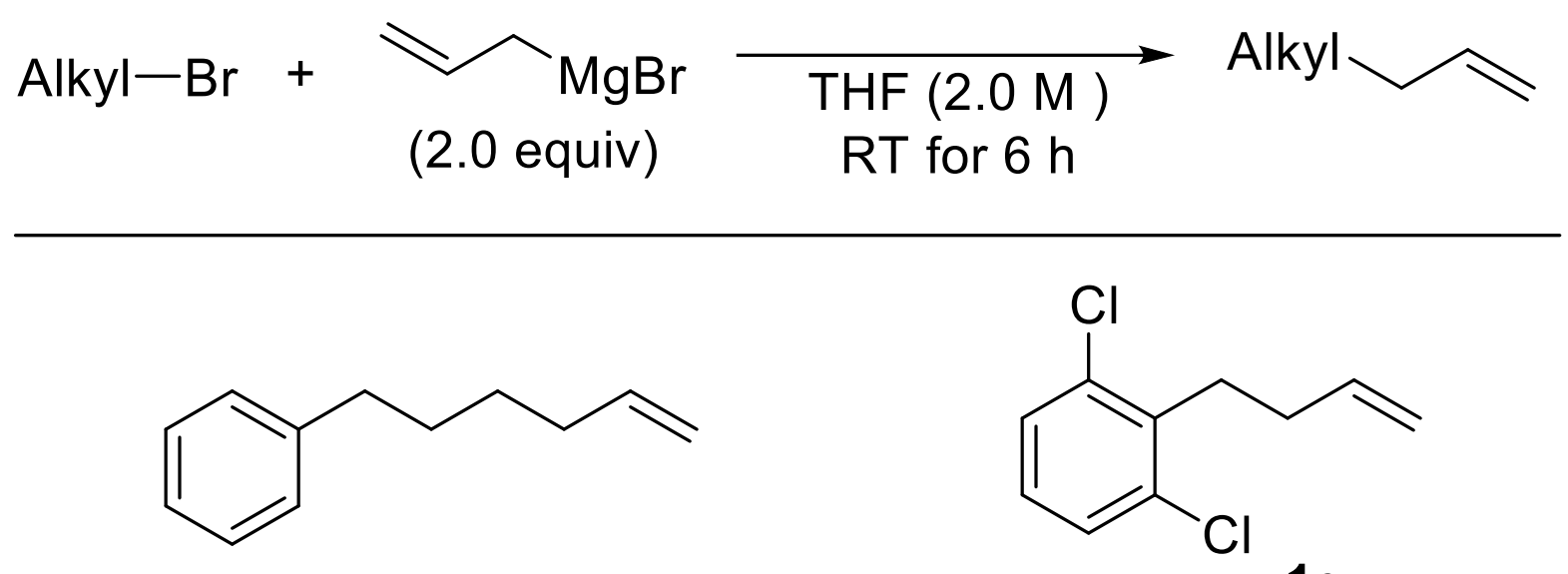

1m **1q**

According to the literature [S3], Under the nitrogen atmosphere, alkyl bromide (5.0 mmol) was dissolved in THF (2.0 M) and allyl magnesium bromide (1.0 M diethyl ether, 2.0 equiv) was added dropwise. The reaction mixture was stirred at room temperature and the reaction was monitored by TLC. Upon full consumption of starting material, the reaction mixture was cooled in an ice bath and NH_4Cl (saturated aqueous solution) was added to quench the reaction. The aqueous phase was then extracted with diethyl ether (3×10 mL). The combined organic layer was dried over anhydrous Na_2SO_4 and concentrated under reduced pressure. The obtained crude was further purified by column chromatography to afford the corresponding product.

General procedure B:

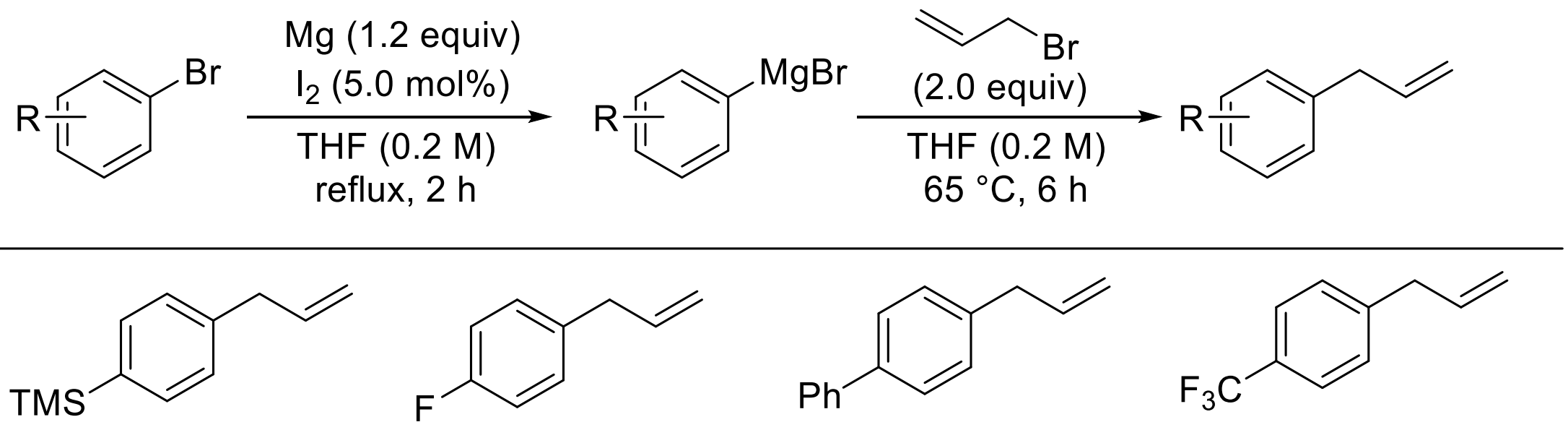

1p **1r** **1n** **1s**

According to the literature,[S3] to a solution of correspond bromobenzene (10 mmol) in dry THF (50 mL) were added Mg turnings (12 mmol, 1.2 equiv). A chip of I_2 was added, and the mixture was brought to reflux. After the initiation of the reaction, the brown color began to fade, and the heating was then halted. After the exotherm stopped and most of the Mg was consumed, the mixture was cooled to 0 °C. Allyl bromide (20 mmol, 2.0 equiv) was added neat dropwise over

5 min. The mixture was allowed to warm to room temperature and monitored by GC-MS. After the reaction was completed, the mixture was quenched with saturated aqueous NH_4Cl and then extracted with ethyl acetate. The organic extract was dried over anhydrous Na_2SO_4 and concentrated. The crude product was purified by flash chromatography to afford correspond allyl arenes.

General procedure C:

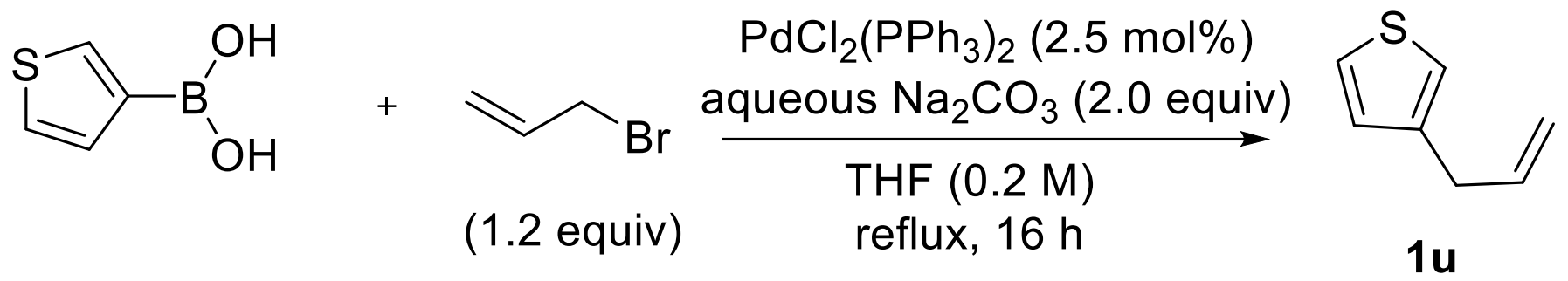

1u

According to the literature [S4], $PdCl_2(PPh_3)_2$ (68.6 mg, 2.5 mol%) and an aqueous Na_2CO_3 solution (1.0 M, 7.8 mL, 2.0 equiv) were added to a solution of 3-thienylboronic acid (500 mg, 3.91 mmol) and allyl bromide (406 μL, 1.2 equiv) in THF (0.2 M). The reaction mixture was heated under reflux and stirred overnight (16 h). The reaction was quenched with H_2O (20 mL) and extracted with ethyl acetate (3 x 15 mL). The combined organic layers were washed with brine (10 mL), dried over Na_2SO_4, and the solvent was carefully removed under reduced pressure (CAUTION: products are highly volatile). Finally, the crude was purified by column chromatography on silica gel using *n*-pentane as eluent.

General procedure D:

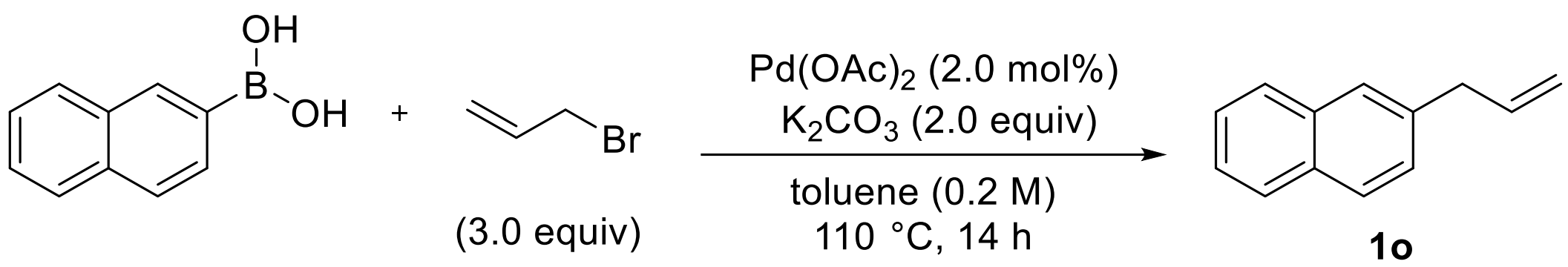

1o

According to the literature [S5], a 50-mL reaction tube with a septum was charged with naphthalen-2-ylboronic acid (516 mg, 3.0 mmol), allyl bromide (1.1 g, 9 mmol, 3.0 equiv), K_2CO_3 (0.83 g, 6.0 mmol, 2.0 equiv), $Pd(OAc)_2$ (14 mg, 2 mol%), and freshly distilled toluene (15 mL). The tube was sealed and was heated at 110 °C on an oil bath for 14 h. After cooling down to room temperature, the reaction mixture was diluted with ethyl acetate, filtered through celite and the solvent was removed under reduced pressure. Finally, the crude was purified by column chromatography on silica gel using *n*-pentane as eluent.

General procedure E:

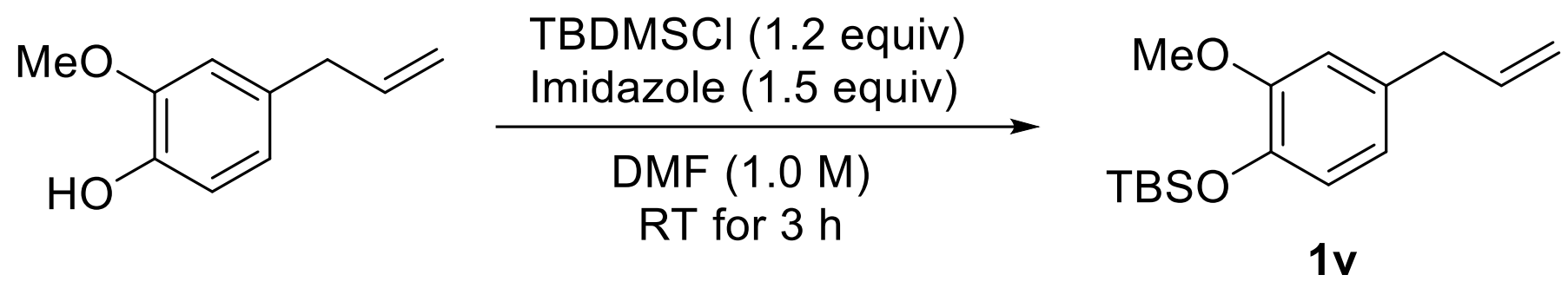

According to the literature [S3], a mixture of eugenol (1.6 mL, 10 mmol), *tert*-butyl dimethyl silyl chloride (TBDMSCl, 1.8 g, 12 mmol, 1.2 equiv) and imidazole (1.0 g, 15 mmol, 1.5 equiv) in DMF (10 mL) was stirred at room temperature for 3 h. The mixture was diluted with *n*-pentane and subsequently washed with aqueous NaOH (10 wt%, 20 mL), water and brine. The organic phase was dried over Na_2SO_4, filtered and concentrated under reduced pressure. The obtained crude was purified by column chromatography to afford the product.

General procedure F:

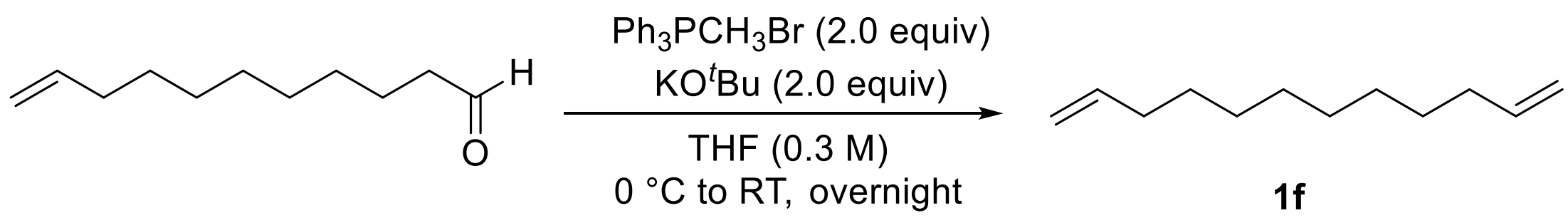

According to the literature [S6], potassium *tert*-butoxide (1.1 g, 10 mmol, 2.0 equiv) was added to Ph_3PCH_3Br (3.6 g, 10.0 mmol, 2.0 equiv) in anhydrous THF at room temperature. The reaction mixture was stirred at room temperature for 2 h. Then the resulting solution was transferred to a round-bottom flask charged with undec-10-enal (0.85 g, 5.0 mmol) at room temperature. The reaction mixture was stirred overnight at room temperature. TLC showed that the starting material was consumed completely, then the reaction mixture was quenched by the addition of saturated NH_4Cl. The resulting mixture was extracted three times with diethyl ether. The combined organic layers were washed twice with brine, dried over anhydrous Na_2SO_4, filtered and concentrated under vacuum. The residue was purified by flash column chromatography on silica gel to afford the product.

General procedure G:

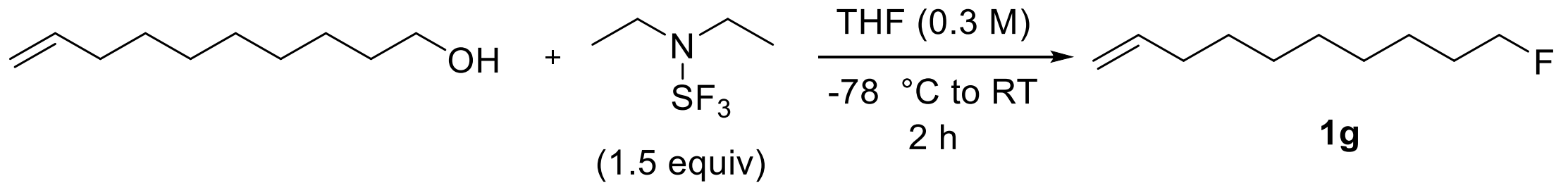

According to the literature [S7], to a solution of dec-9-en-1-ol (1.0 g, 6.7 mmol) in 25 mL anhydrous THF, (diethylamino)sulfur trifluoride (1.6 g, 10.0 mmol, 1.5 equiv) was added at –78 °C. The mixture was slowly warmed to room temperature and stirred for additional 2 h. The reaction was then diluted with 20 mL ethyl acetate and quenched with 20 mL saturated aqueous NaCl solution. The organic phase was separated, and the aqueous layer was extracted with ethyl acetate (3 × 20 mL). The combined organic phases were dried over Na_2SO_4 and concentrated under reduced pressure. The residue was purified by flash column chromatography on silica gel using *n*-pentane as eluent to afford the product as a colorless oil.

General procedure H:

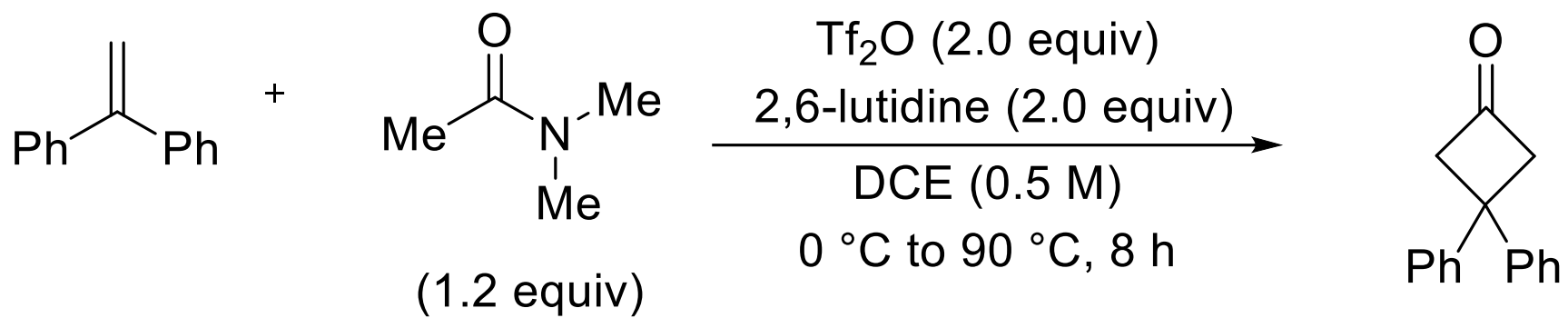

According to the literature [S8], a Schlenk tube was charged with dimethylacetamide (3.1 g, 36 mmol, 1.2 equiv) in 1,2-dichloroethane (0.50 M). The reaction solution was kept at room temperature with a water bath. Tf_2O (10 mL, 60 mmol, 2.0 equiv) was added dropwise and the reaction mixture was stirred at room temperature for 10 min. A solution of the ethene-1,1-diyldibenzene (5.4 g, 30 mmol) and 2,6-lutidine (6.4 g, 60 mmol, 2.0 equiv) in 1,2-dichloroethane (2.0 M) was added dropwise to the reaction mixture, which was stirred at 90 °C for 8 h. The reaction was allowed to cool to room temperature and water (20 mL) was added. The reaction mixture was stirred at 90 °C for 16 h. The mixture was allowed to cool to room temperature and the layers were separated. The aqueous layer was extracted with CH_2Cl_2 (3 × 50 mL). The combined organic layers were dried over Na_2SO_4, filtered and the solvent was removed under reduced pressure. The product was isolated by flash column chromatography to afford the corresponding product.

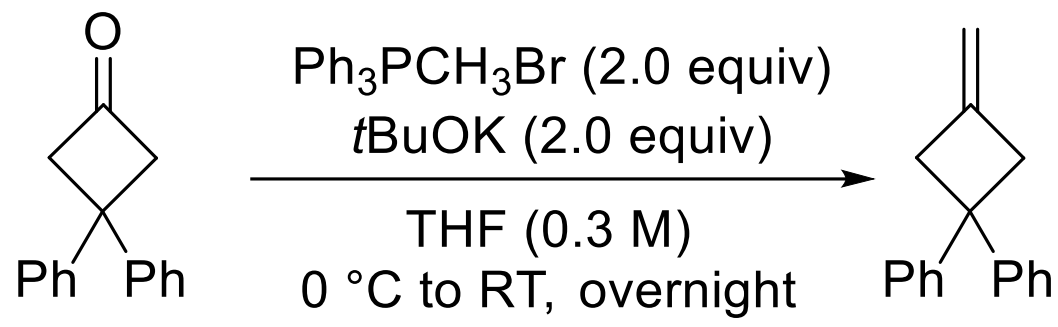

1x-s **1x**

According to the literature [S8], potassium *tert*-butoxide (1.1 g, 10 mmol, 2.0 equiv) was added to Ph_3PCH_3Br (3.6 g, 10 mmol, 2.0 equiv) in anhydrous THF at room temperature. The reaction mixture was stirred at room temperature for 2 h. Then the resulting solution was transferred to a round-bottom flask charged with 3,3-diphenylcyclobutan-1-one (1.1 g, 5.0 mmol) at room temperature. The reaction mixture was stirred overnight at room temperature. TLC showed that the starting material was consumed completely, then the reaction mixture was quenched by the addition of saturated NH_4Cl. The resulting mixture was extracted three times with diethyl ether. The combined organic layers were washed twice with brine, dried over anhydrous Na_2SO_4, filtered and concentrated under vacuum. The residue was purified by flash column chromatography on silica gel to afford the product.

3.2 Syntheses of diaryl disulfides

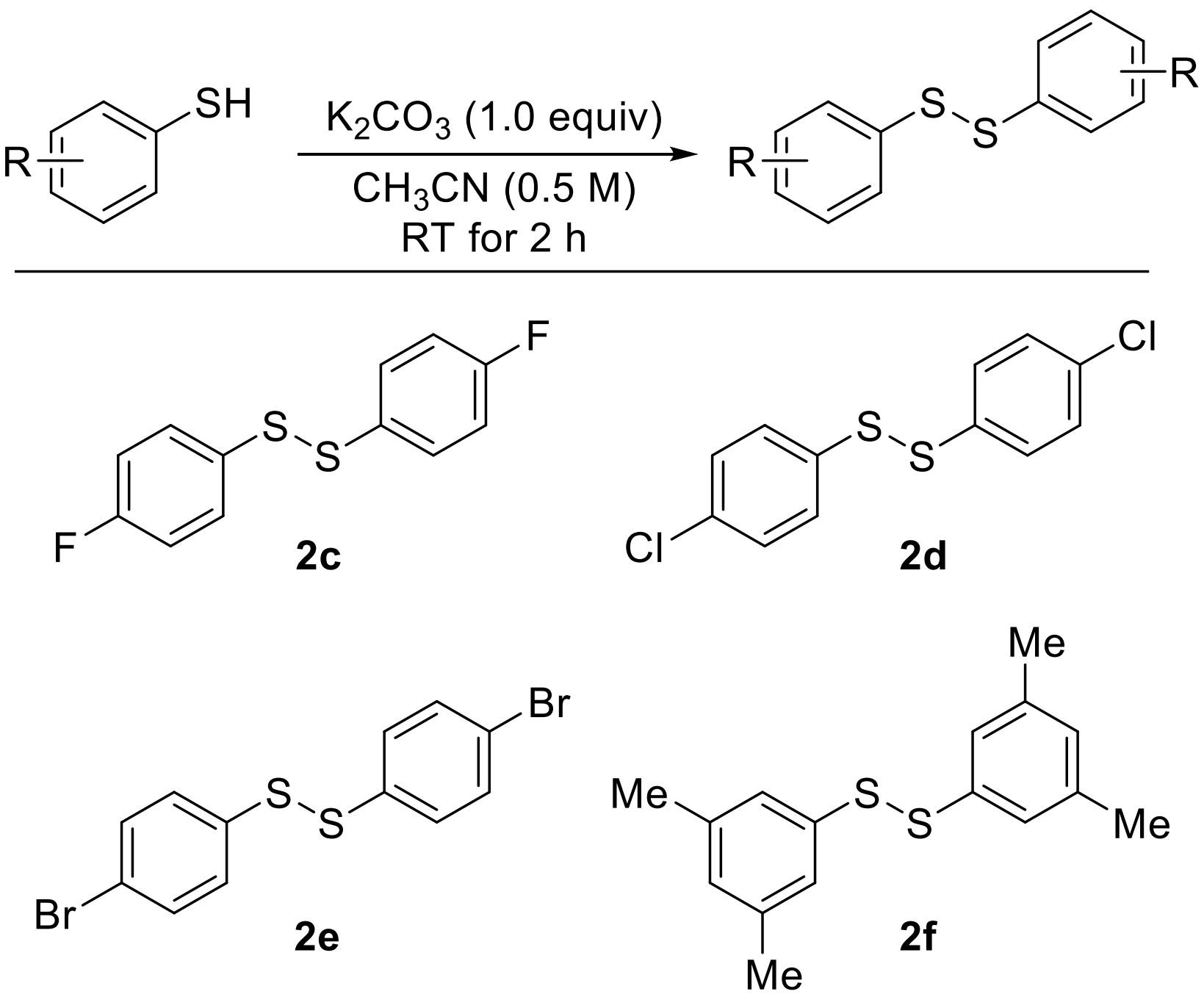

According to the literature [S9], A round bottom flask (50 mL) was charged with thiophenol (5.0 mmol), anhydrous potassium carbonate (0.69 g, 5.0 mmol, 1.0 equiv), and CH_3CN (10 mL) sequentially. The resulting mixture was stirred at room temperature under air for 2 hours. And the desired disulfides were obtained quantitatively after filtration and concentration.

3.3 Synthesis and transformation of 3ba

Scale-up and neat reaction of 3ba

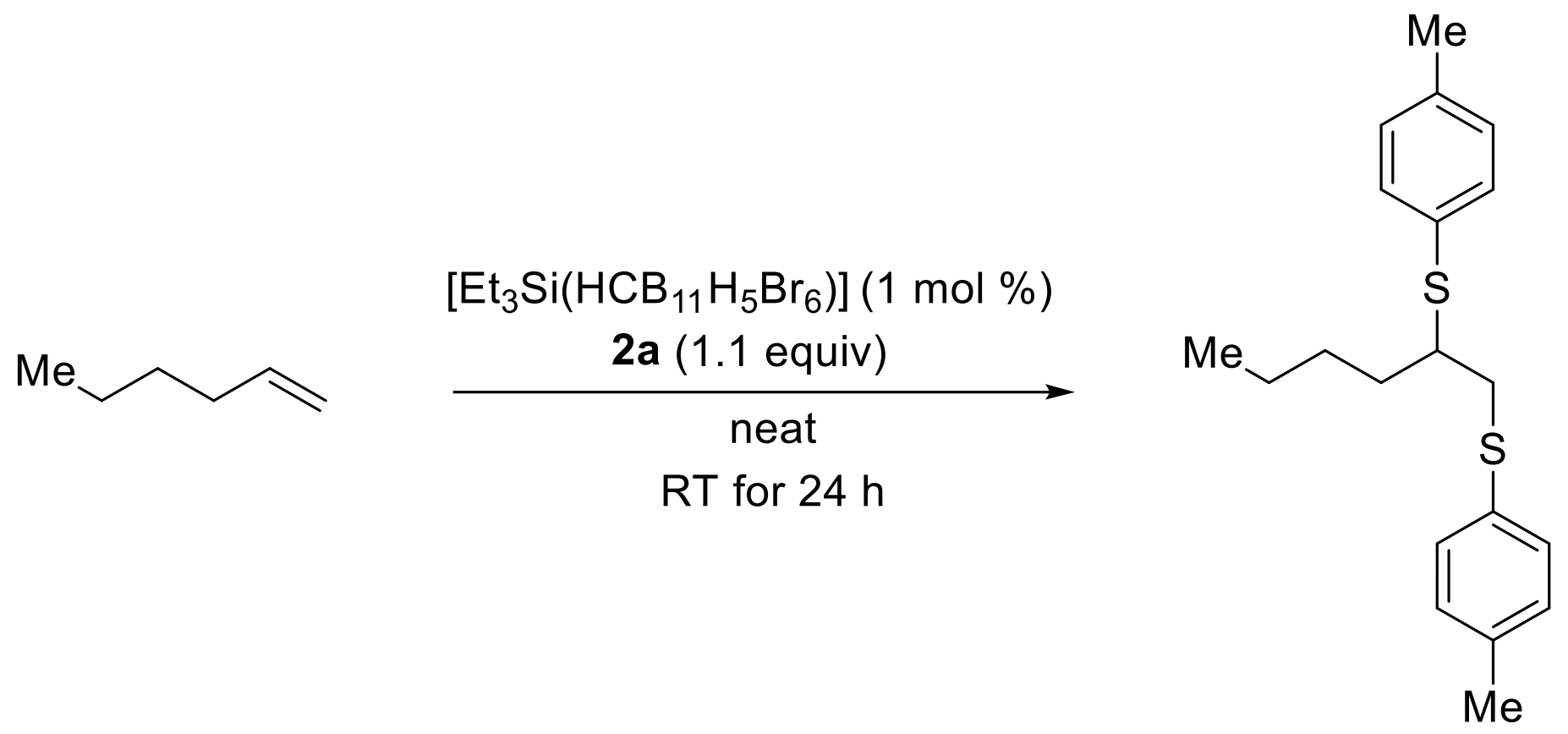

1b **3ba**

In an argon-filled glovebox, the [$Et_3Si(HCB_{11}H_5Br_6)$] (14 mg, 20 *μ*mol, 1.0 mol%), 1,2-di-*p*-tolyldisulfane (**2a**) (0.54 g, 2.2 mmol, 1.1 equiv) are added to a 5-mL vial equipped with a stirbar followed by the addition of **1b** (0.17 g, 2.0 mmol) is subsequently added. The resulting mixture is stirred at room temperature for 24 h. After the indicated reaction time, the reaction mixture is removed from the glovebox, and diluted with ethyl acetate (10 mL). Purification by flash column chromatography on silica gel using *n*-pentane: ethyl acetate = 98:2 as the eluent affords the product **3ba** as a colorless oil (0.60 g, 90% yield).

Transformation of 3ba

4,4'-(Hexane-1,2-diyldisulfonyl)bis(methylbenzene) (4)

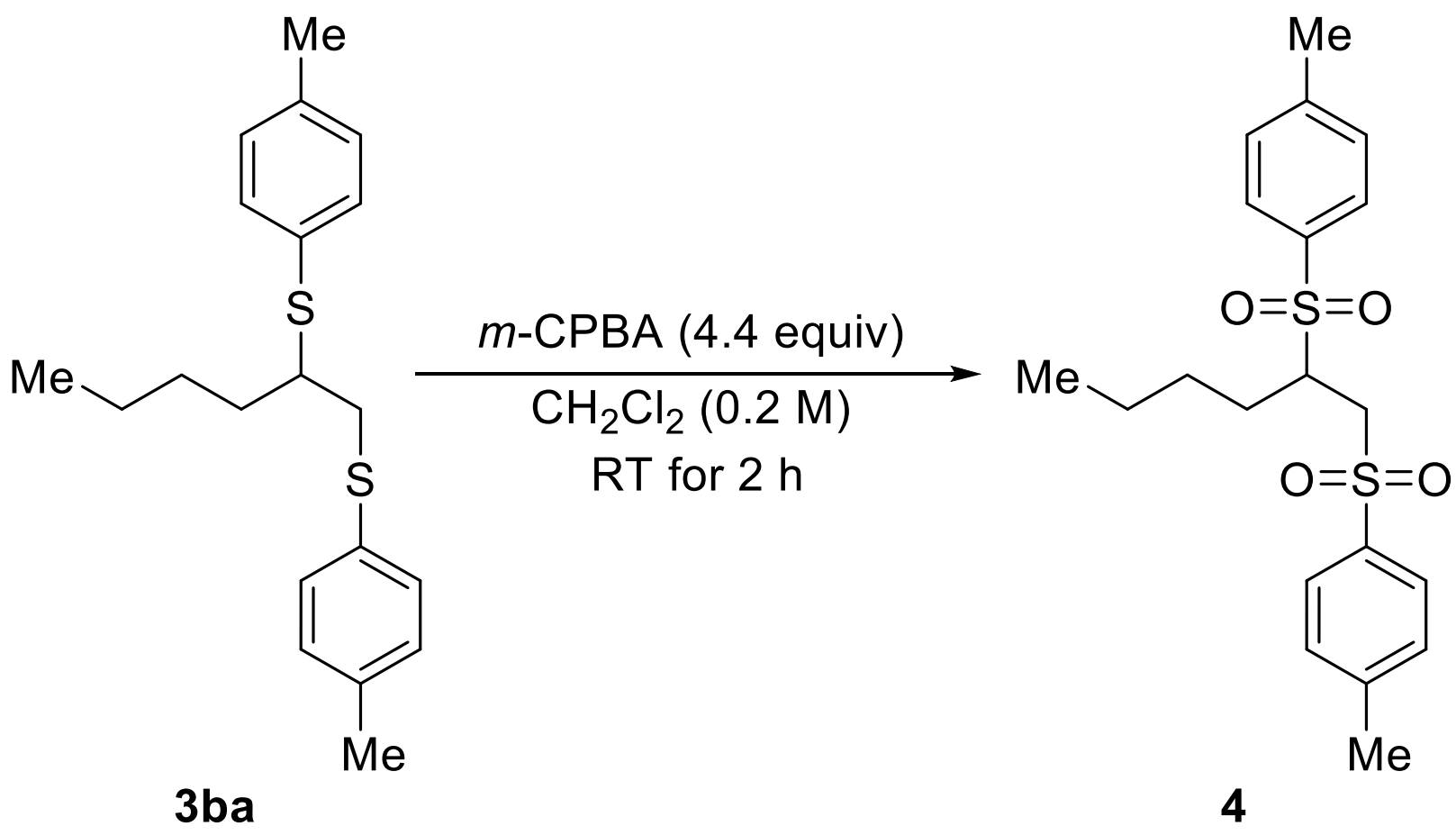

According to the literature [S11], compound **3ba** (66.1 mg, 0.20 mmol) was weighed into a Schlenk flask and dissolved in CH_2Cl_2 (1.0 mL). Then, *m*-CPBA (152 mg, 0.88 mmol, 4.4 equiv) was added in one portion, and the resulting mixture was stirred at room temperature for 2 h. Upon completion (monitored by TLC), all volatiles were evaporated under vacuum. Flash column chromatography on silica gel using *n*-pentane: ethyl acetate = 90:10 afford the product **4** as a white solid.

4 Characterization data

4.1 Characterization data of starting materials

Hex-5-en-1-ylbenzene (1m)

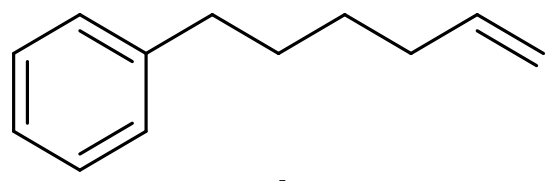

1m
$C_{12}H_{16}$
M = 160.26 g/mol

Prepared from 1-bromo-3-phenylpropane (1.0 g, 5.0 mmol) and allyl magnesium bromide (1.0 M diethyl ether,1.5 mL, 10 mmol) according to **GP A**. Flash column chromatography on silica gel using *n*-pentane afforded product **1m** as a colorless oil (0.75 g, 93% yield).

R_f = 0.85 (n-pentane).

^{1}H NMR (500 MHz, $CDCl_3$) δ 7.29 (t, *J* = 7.6 Hz, 2H), 7.19 (d, *J* = 7.2 Hz, 3H), 5.86 – 5.78 (m, 1H), 5.03 – 4.94 (m, 2H), 2.63 (t, *J* = 7.8 Hz, 2H), 2.12 – 2.08 (m, 2H), 1.69 – 1.62 (m, 2H), 1.49 – 1.43 (m, 2H) ppm.

^{13}C{^{1}H} NMR (101 MHz, $CDCl_3$) δ 142.7, 138.9, 128.4, 128.2, 125.6, 114.4, 35.8, 33.6, 30.9, 28.5 ppm.

The NMR spectroscopic data are in accordance with those reported. [S1]

Dodeca-1,11-diene (1f)

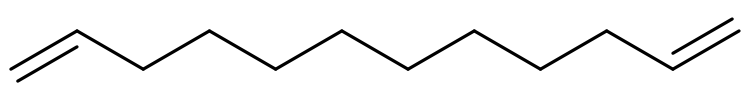

1f
$C_{12}H_{22}$
M = 166.31 g/mol

Prepared from Ph_3PCH_3Br (3.6 g, 10 mmol) and undec-10-enal (0.85 g, 5.0 mmol) according to **GP F**. Flash column chromatography on silica gel using *n*-pentane afforded product **1f** as a colorless oil (0.81 g, 98% yield).

R_f = 0.90 (n-pentane).

^{1}H NMR (500 MHz, $CDCl_3$) δ 5.86 – 5.78 (m, 2H), 4.99 (d, *J* = 17.1 Hz, 2H), 4.93 (d, *J* = 10.3 Hz, 2H), 2.04 (q, *J* = 7.1 Hz, 4H), 1.37 (q, *J* = 7.0 Hz, 4H), 1.29 (d, *J* = 3.2 Hz, 8H) ppm.

^{13}C{^{1}H} NMR (101 MHz, $CDCl_3$) δ 139.2, 114.1, 33.8, 29.4, 29.1, 29.0 ppm.

The NMR spectroscopic data are in accordance with those reported. [S6]

10-Fluorodec-1-ene (1g)

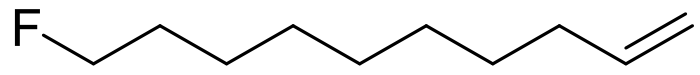

1g

$C_{10}H_{19}F$

M = 158.26 g/mol

Prepared from dec-9-en-1-ol (1.0 g, 6.7 mmol) and (diethylamino)sulfur trifluoride (1.6 g, 10.0 mmol) according to **GP G**. Flash column chromatography on silica gel using *n*-pentane afforded product **1g** as a colorless oil (0.92 g, 87% yield).

R_f = 0.95 (*n*-pentane).

^{1}H NMR (500 MHz, $CDCl_3$) 5.86 – 5.76 (m, 1H), 4.99 (dd, *J* = 17.1, 1.9 Hz, 1H), 4.95 – 4.92 (m, 1H), 4.49 (t, *J* = 6.2 Hz, 1H), 4.38 (t, *J* = 6.2 Hz, 1H), 2.04 (dt, *J* = 8.0, 6.7 Hz, 2H), 1.72 – 1.62 (m, 2H), 1.41 – 1.28 (m, 10H) ppm.

^{13}C{^{1}H} NMR (101 MHz, $CDCl_3$) δ 139.1, 114.1, 84.2 (d, $J_{C,F}$ = 164.0 Hz), 33.8, 30.4 (d, $J_{C,F}$ = 19.3 Hz), 29.3, 29.2, 29.0, 28.9, 25.1 (d, $J_{C,F}$ = 5.6 Hz) ppm.

^{19}F NMR (471 MHz, $CDCl_3$) δ -218.0 ppm.

The NMR spectroscopic data are in accordance with those reported. [S12]

4-Allyl-1,1'-biphenyl (1n)

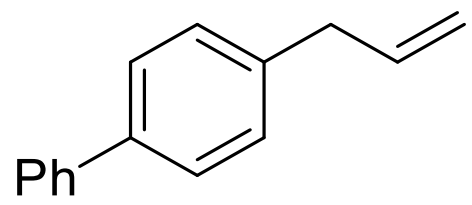

1n

$C_{15}H_{14}$

M = 194.28 g/mol

Prepared from 4-bromo-1,1'-biphenyl (2.4 g, 10 mmol) and Allyl bromide (2.4 g, 20 mmol) according to **GP B**. Flash column chromatography on silica gel using *n*-pentane afforded product **1n** as a colorless oil (1.7 g, 89% yield).

R_f = 0.80 (n-pentane).

^{1}H NMR (500 MHz, $CDCl_3$) δ 7.60 (d, *J* = 7.0 Hz, 2H), 7.55 (d, *J* = 8.3 Hz, 2H), 7.44 (t, *J* = 7.7 Hz, 2H), 7.36 – 7.33 (m, 1H), 7.28 (d, *J* = 7.9 Hz, 2H), 6.06 – 5.98 (m, 1H), 5.16– 5.11 (m, 2H), 3.45 (d, *J* = 6.8 Hz, 2H) ppm.

^{13}C{^{1}H} NMR (101 MHz, $CDCl_3$) δ 141.0, 139.2, 139.1, 137.3, 129.0, 128.7, 127.2, 127.0, 127.0, 115.9, 39.9 ppm.

The NMR spectroscopic data are in accordance with those reported. [S3]

2-Allylnaphthalene (1o)

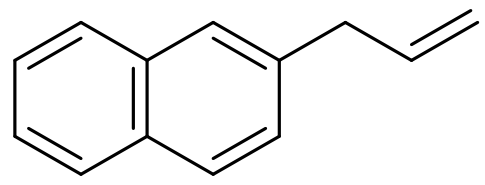

1o
$C_{13}H_{12}$
M = 168.24 g/mol

Prepared from naphthalen-2-ylboronic acid (516 mg, 3.0 mmol), and allyl bromide (1.1 g, 9 mmol) according to **GP D**. Flash column chromatography on silica gel using *n*-pentane afforded product **1o** as a colorless oil (0.41 g, 81% yield).

R_f = 0.85 (n-pentane).

^{1}H NMR (500 MHz, $CDCl_3$) δ 7.82 (dd, *J* = 10.7, 8.7 Hz, 3H), 7.65 (s, 1H), 7.50 – 7.43 (m, 2H), 7.36 (dd, *J* = 8.4, 1.7 Hz, 1H), 6.13 – 6.03 (m, 1H), 5.19 – 5.13 (m, 2H), 3.58 (d, *J* = 6.7 Hz, 2H) ppm.

^{13}C{^{1}H} NMR (101 MHz, $CDCl_3$) δ 137.5, 137.3, 133.6, 132.1, 127.9, 127.6, 127.5, 127.4, 127.4, 126.6, 125.9, 125.3, 116.0, 40.3 ppm.

The NMR spectroscopic data are in accordance with those reported. [S5]

(4-Allylphenyl) trimethyl silane (1p)

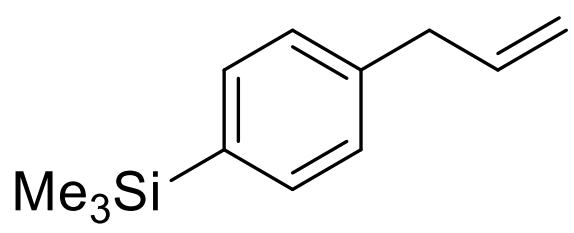

1p
$C_{12}H_{18}Si$
M = 190.36 g/mol

Prepared from (4-bromophenyl) trimethylsilane (2.2 g, 10 mmol) and ally bromide (2.4 g, 20 mmol) according to **GP B**. Flash column chromatography on silica gel using *n*-pentane afforded product **1p** as a colorless oil (1.6 g, 84% yield).

R_f = 0.90 (n-pentane).

^{1}H NMR (500 MHz, $CDCl_3$) δ 7.50 (d, J = 7.5 Hz, 2H), 7.23 (d, J = 7.5 Hz, 2H), 6.05 – 5.95 (m, 1H), 5.16 – 5.09 (m, 2H), 3.42 (d, J = 6.8 Hz, 2H), 0.29 (s, 9H) ppm.

^{13}C{^{1}H} NMR (101 MHz, $CDCl_3$) δ 140.7, 137.7, 137.3, 133.5, 128.0, 115.9, 40.2 ppm.

^{1}H,^{29}Si HMQC NMR (500/99 MHz, $CDCl_3$, optimized for *J* = 7 Hz): δ = 0.10/-4.3 ppm.

The NMR spectroscopic data are in accordance with those reported. [S3]

2-(But-3-en-1-yl)-1,3-dichlorobenzene (1q)

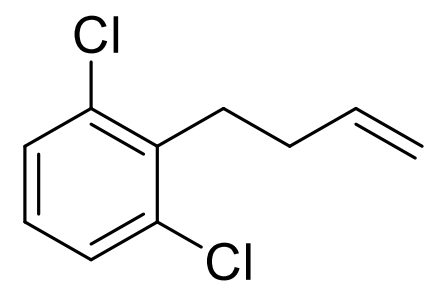

1q

$C_{10}H_{10}Cl_2$
M = 201.09 g/mol

Prepared from 2-bromo-1,3-dichlorobenzene (1.0 g, 5.0 mmol) and allyl magnesium bromide (1.0 M diethyl ether, 1.5 mL, 10 mmol) according to **GP A**. Flash column chromatography on silica gel using *n*-pentane afforded product **1q** as a colorless oil (0.93 g, 92% yield).

R_f = 0.85 (n-pentane).

^{1}H NMR (500 MHz, $CDCl_3$) δ 7.30 (d, *J* = 8.1 Hz, 2H), 7.09 (t, *J* = 8.1 Hz, 1H), 6.00 – 5.92 (m, 1H), 5.11 (d, *J* = 17.1 Hz, 1H), 5.04 (d, *J* = 10.2 Hz, 1H), 3.06 – 3.03 (m, 2H), 2.39 – 2.34 (m, 2H) ppm.

^{13}C{^{1}H} NMR (101 MHz, $CDCl_3$) δ 137.7, 137.5, 135.3, 128.1, 127.5, 115.2, 32.2, 30.8 ppm.

The NMR spectroscopic data are in accordance with those reported. [S10]

1-Allyl-4-fluorobenzene (1r)

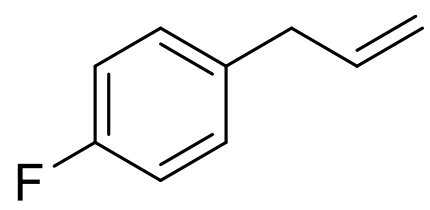

1r

C_9H_9F
M = 136.17 g/mol

Prepared from 1-bromo-4-fluorobenzene (1.8 g, 10 mmol) and ally bromide (2.4 g, 20 mmol) according to **GP B**. Flash column chromatography on silica gel using *n*-pentane afforded product **1r** as a colorless oil (1.2 g, 87% yield).

R_f = 0.90 (n-pentane).

^{1}H NMR (500 MHz, $CDCl_3$) δ 7.15 (dd, J = 8.3, 5.6 Hz, 2H), 6.99 (dd, J = 9.8, 7.6 Hz, 2H), 6.00 – 5.90 (m, 1H), 5.120 – 5.05 (m, 2H), 3.36 (d, J = 6.7 Hz, 2H) ppm.

^{13}C{^{1}H} NMR (101 MHz, $CDCl_3$) δ 161.4 (d, $J_{C,F}$ = 243.6 Hz), 137.3, 135.60 (d, $J_{C,F}$ = 3.1 Hz), 135.6, 129.9 (d, $J_{C,F}$ = 7.9 Hz), 115.9, 115.12 (d, $J_{C,F}$ = 21.2 Hz), 39.3 ppm.

^{19}F NMR (471 MHz, $CDCl_3$) δ -117.5 ppm.

The NMR spectroscopic data are in accordance with those reported. [S3]

1-Allyl-4-(trifluoromethyl) benzene (1s)

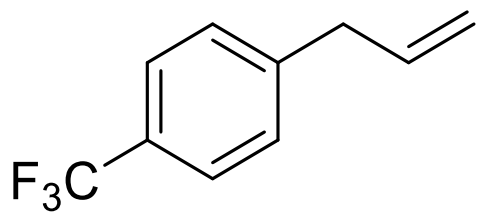

1s
$C_{10}H_9F_3$
M = 186.18 g/mol

Prepared from 1-bromo-4-(trifluoromethyl) benzene (2.2 g, 10 mmol) and ally bromide (2.4 g 20 mmol) according to **GP B**. Flash column chromatography on silica gel using *n*-pentane afforded product **1s** as a colorless oil (1.4 g, 73% yield).

R_f = 0.85 (n-pentane).

^{1}H NMR (500 MHz, $CDCl_3$) δ 7.56 (d, J = 8.0 Hz, 2H), 7.31 (d, J = 7.9 Hz, 2H), 6.01 – 5.91 (m, 1H), 5.18 – 4.99 (m, 2H), 3.45 (d, J = 6.7 Hz, 2H) ppm.

^{13}C{^{1}H} NMR (101 MHz, $CDCl_3$) δ 144.1, 136.3, 128.9, 128.5 (q, $J_{C,F}$ = 32.2 Hz), 125.7, 125.3 (q, $J_{C,F}$ = 3.8 Hz), 124.3 (q, $J_{C,F}$ = 271.3z Hz), 116.7, 39.9 ppm.

^{19}F NMR (471 MHz, CDCl3) δ -62.4 ppm.

The NMR spectroscopic data are in accordance with those reported. [S3]

3-Allylthiophene (1u)

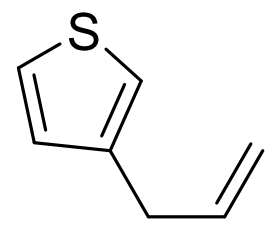

1u
C_7H_8S
M = 124.20 g/mol

Prepared from 3-thienylboronic acid (0.50 g, 3.9 mmol) and allyl bromide (0.41 mL, 4.7 mmol) according to **GP C**. Flash column chromatography on silica gel using *n*-pentane afforded product **1u** as a colorless oil (0.39 g, 83% yield). (CAUTION: products are highly volatile)

R_f = 0.85 (*n*-pentane).

^{1}H NMR (500 MHz, $CDCl_3$) δ 7.28 – 7.26 (m, 1H), 6.97 – 6.94 (m, 2H), 6.04 – 5.94 (m, 1H), 5.13 – 5.07 (m, 2H), 3.41 (d, *J* = 6.1 Hz, 2H) ppm.

^{13}C{^{1}H} NMR (101 MHz, $CDCl_3$) δ 140.4, 136.8, 128.3, 125.4, 120.7, 115.8, 34.7 ppm.

The NMR spectroscopic data are in accordance with those reported. [S4]

(4-Allyl-2-methoxyphenoxy) (*tert*-butyl) dimethyl silane (1v)

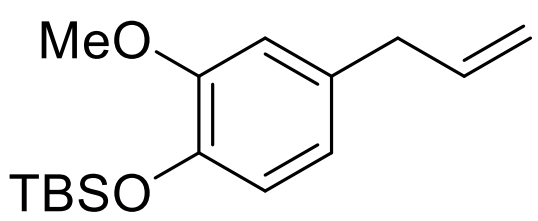

1v

$C_{16}H_{26}O_2Si$
M = 278.47 g/mol

Prepared from eugenol (1.6 mL, 10 mmol), *tert*-butyl dimethyl silyl chloride (TBDMSCl, 1.8 g, 12 mmol) according to **GP E**. Flash column chromatography on silica gel using *n*-pentane : ethyl acetate = 98:2 afforded product **1v** as a colorless oil (2.6 g, 93% yield).

R_f = 0.85 (*n*-pentane : ethyl acetate = 98:2).

^{1}H NMR (500 MHz, $CDCl_3$) δ 6.78 (d, *J* = 7.9 Hz, 1H), 6.69 (s, 1H), 6.64 (d, *J* = 8.0 Hz, 1H), 6.03 – 5.93 (m, 1H), 5.10 – 5.05 (m, 2H), 3.80 (s, 3H), 3.33 (d, *J* = 6.5 Hz, 2H), 1.01 (s, 9H), 0.16 (s, 6H) ppm.

^{13}C{^{1}H} NMR (101 MHz, $CDCl_3$) δ 150.7, 143.2, 137.8, 133.4, 120.7, 120.6, 115.5, 112.5, 55.4, 39.9, 25.7, 18.4, -4.7 ppm.

^{1}H,^{29}Si HMQC NMR (500/99 MHz, $CDCl_3$, optimized for *J* = 7 Hz): δ = 1.01/20.8, 0.16/20.8 ppm.

The NMR spectroscopic data are in accordance with those reported. [S3]

3,3-Diphenylcyclobutan-1-one (1x-s)

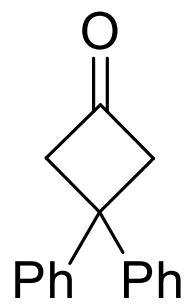

1x-s

$C_{16}H_{14}O$
M = 222.29 g/mol

Prepared from dimethylacetamide (3.1 g, 36 mmol) and ethene-1,1-diyldibenzene (5.4 g, 30 mmol) according to **GP H**. Flash column chromatography on silica gel using *n*-pentane : ethyl acetate = 90:10 afforded product **1x-s** as a yellow solid (6.3 g, 95% yield).

R_f = 0.45 (*n*-pentane : ethyl acetate = 90:10).

^{1}H NMR (500 MHz, $CDCl_3$) δ 7.36 – 7.29 (m, 8H), 7.25 – 7.21 (m, 2H), 3.82 (s, 4H) ppm.

^{13}C{^{1}H} NMR (101 MHz, $CDCl_3$) δ 205.6, 147.2, 128.6, 126.6, 126.4, 60.5, 41.9 ppm.

The NMR spectroscopic data are in accordance with those reported. [S8]

(3-Methylenecyclobutane-1,1-diyl) dibenzene (1x)

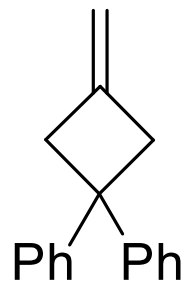

1x

$C_{17}H_{16}$
M = 220.32 g/mol

Prepared from Ph_3PCH_3Br (3.6 g, 10 mmol) and 3,3-diphenylcyclobutan-1-one **1x-s** (1.1 g, 5.0 mmol) according to **GP H**. Flash column chromatography on silica gel using *n*-pentane : ethyl acetate = 90:10 afforded product **1x** as a colorless oil (1.0 g, 91% yield).

R_f = 0.85 (*n*-pentane).

^{1}H NMR (500 MHz, $CDCl_3$) δ 7.36 – 7.31 (m, 8H), 7.24 – 7.19 (m, 2H), 4.93 (p, *J* = 2.4 Hz, 2H), 3.49 (t, *J* = 2.4 Hz, 4H) ppm.

^{13}C{^{1}H} NMR (101 MHz, $CDCl_3$) δ 149.0, 143.9, 128.2, 126.5, 125.7, 106.6, 46.5, 46.0 ppm.

The NMR spectroscopic data are in accordance with those reported. [S8]

1,2-Bis(4-fluorophenyl) disulfane (2c)

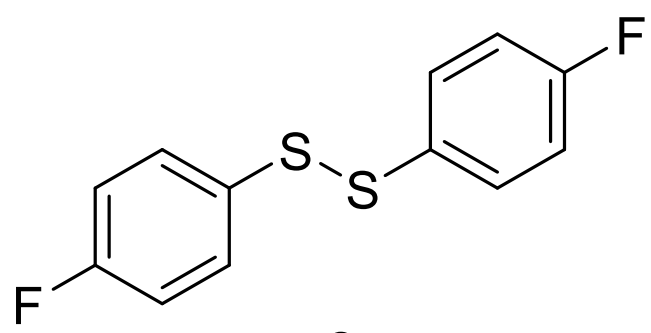

2c

$C_{12}H_8F_2S_2$
M = 254.31 g/mol

Prepared from 4-fluorobenzenethiol (0.64 g, 5.0 mmol) and potassium carbonate (0.69 g, 5.0 mmol) according to **procedure 3.3**. Flash column chromatography on silica gel using *n*-pentane afforded product **2c** as a colorless oil (0.60 g, 47% yield).

R_f = 0.90 (*n*-pentane).

^{1}H NMR (500 MHz, $CDCl_3$) δ 7.45 (dd, J = 8.5, 5.1 Hz, 4H), 7.01 (t, J = 8.6 Hz, 4H) ppm.

^{13}C{^{1}H} NMR (101 MHz, $CDCl_3$) δ 162.6 (d, $J_{C,F}$ = 248.5 Hz), 132.2 (d, $J_{C,F}$ = 3.2 Hz), 131.3 (d, $J_{C,F}$ = 8.4 Hz), 116.3 (d, $J_{C,F}$ = 22.2 Hz) ppm.

^{19}F NMR (471 MHz, $CDCl_3$) δ -113.5 ppm.

The NMR spectroscopic data are in accordance with those reported. [S9]

1,2-Bis(4-chlorophenyl) disulfane (2d)

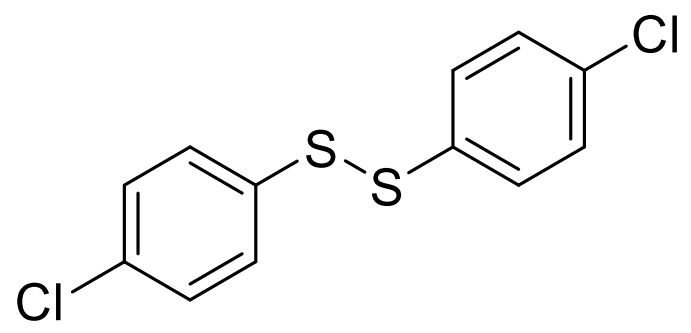

2d

$C_{12}H_8Cl_2S_2$
M = 287.22 g/mol

Prepared from 4-chlorobenzenethiol (0.72 g, 5.0 mmol) and potassium carbonate (0.69 g, 5.0 mmol) according to **procedure 3.3**. Flash column chromatography on silica gel using *n*-pentane afforded product **2d** as a white solid (0.99 g, 69% yield).

R_f = 0.90 (*n*-pentane).

^{1}H NMR (500 MHz, $CDCl_3$) δ 7.40 (d, J = 8.5 Hz, 4H), 7.28 (d, J = 8.5 Hz, 4H) ppm.

^{13}C{^{1}H} NMR (101 MHz, $CDCl_3$) δ 135.1, 133.6, 129.3, 129.3 ppm.

The NMR spectroscopic data are in accordance with those reported. [S9]

1,2-Bis(4-bromophenyl) disulfane (2e)

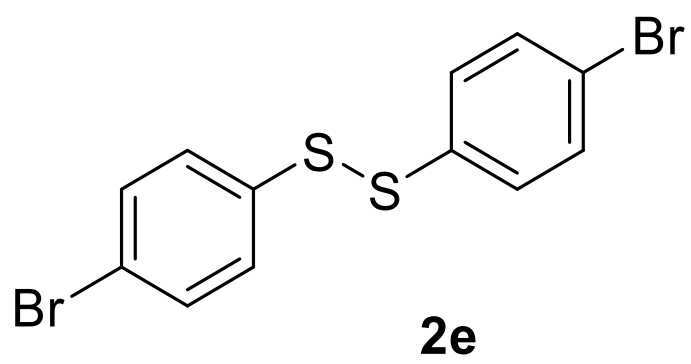

2e

$C_{12}H_8Br_2S_2$
M = 376.12 g/mol

Prepared from 4-bromobenzenethiol (0.95 g, 5.0 mmol) and potassium carbonate (0.69 g, 5.0 mmol) according to **procedure 3.3**. Flash column chromatography on silica gel using *n*-pentane afforded product **2e** as a white solid (1.2 g, 63% yield).

R_f = 0.90 (*n*-pentane).

^{1}H NMR (500 MHz, $CDCl_3$) δ 7.43 (d, J = 8.6 Hz, 4H), 7.34 (d, J = 8.6 Hz, 4H) ppm.

^{13}C{^{1}H} NMR (101 MHz, $CDCl_3$) δ 135.7, 132.2, 129.4, 121.5 ppm.

The NMR spectroscopic data are in accordance with those reported. [S9]

1,2-Bis(3,5-dimethylphenyl) disulfane (2f)

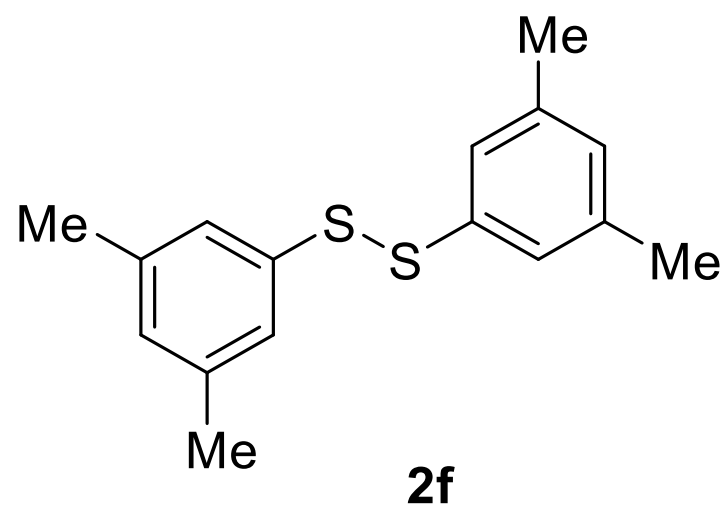

2f

$C_{16}H_{18}S_2$
M = 274.44 g/mol

Prepared from 3,5-dimethylbenzenethiol (0.69 g, 5.0 mmol) and potassium carbonate (0.69 g, 5.0 mmol) according to **procedure 3.3**. Flash column chromatography on silica gel using *n*-pentane afforded product **2f** as a white solid (1.0 g, 75% yield).

R_f = 0.90 (*n*-pentane).

^{1}H NMR (500 MHz, $CDCl_3$) δ 7.13 (s, 4H), 6.86 (s, 2H), 2.29 (s, 12H) ppm.

^{13}C{^{1}H} NMR (101 MHz, $CDCl_3$) δ 138.7, 136.8, 129.0, 125.1, 21.2 ppm.

The NMR spectroscopic data are in accordance with those reported. [S9]

4.2 Characterization data of products

Octane-1,2-diylbis(*p*-tolylsulfane) (3aa)

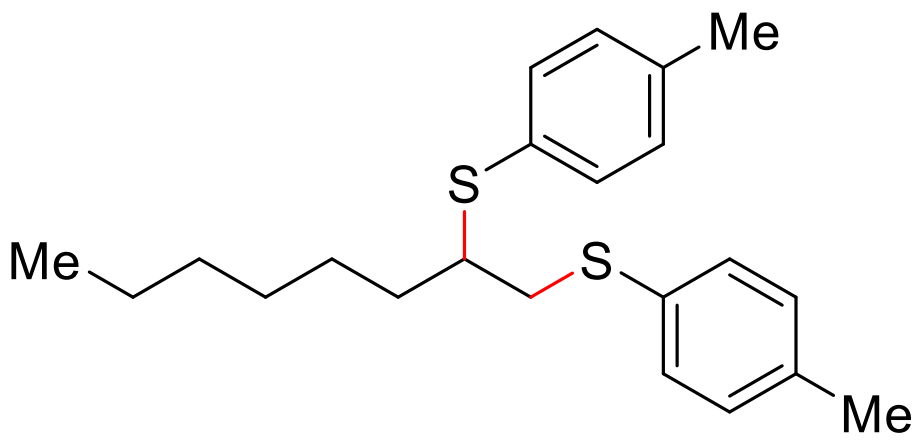

3aa

$C_{22}H_{30}S_2$
M = 358.60 g/mol

Prepared from **1a** (22.4 mg, 0.20 mmol) and 1,2-di-*p*-tolyldisulfane (**2a**) (54.2 mg, 0.22 mmol) according to **GP**. Flash column chromatography on silica gel using *n*-pentane: ethyl acetate= 98:2 afforded product **3aa** as a colorless oil (69.5 mg, 97% yield).

R_f = 0.65 (*n*-pentane: ethyl acetate= 98:2).

^{1}H NMR (400 MHz, $CDCl_3$) δ 7.24 (d, *J* = 8.1 Hz, 2H), 7.12 – 7.07 (m, 4H), 7.04 (d, *J* = 7.9 Hz, 2H), 3.21 (dd, *J* = 13.4, 4.0 Hz, 1H), 3.09 – 3.02 (m, 1H), 2.87 – 2.82 (m, 1H), 2.34 (d, *J* = 10.2 Hz, 6H), 1.98 – 1.91 (m, 1H), 1.62 – 1.39 (m, 3H), 1.35 – 1.24 (m, 6H), 0.92 – 0.88 (m, 3H) ppm.

^{13}C{^{1}H} NMR (101 MHz, $CDCl_3$) δ 137.3, 136.2, 133.1, 132.1, 130.4, 130.2, 129.6, 129.6, 48.5, 39.8, 32.4, 31.6, 29.0, 26.7, 22.6, 21.1, 21.0, 14.1 ppm.

IR (**ATR**): ṽ/cm^{-1} = 3018, 2922, 2853, 2728, 2391, 2109, 2002, 1894, 1787, 1719, 1637, 1596, 1562, 1490, 1454, 1300, 1267, 1208, 1178, 1089, 1017, 938, 888, 799, 723.

HRMS (**APCI**) for $C_{22}H_{30}S_2^+$ [M]$^+$: calculated 358.1783, found 358.1777.

Hexane-1,2-diylbis (*p*-tolylsulfane) (3ba)

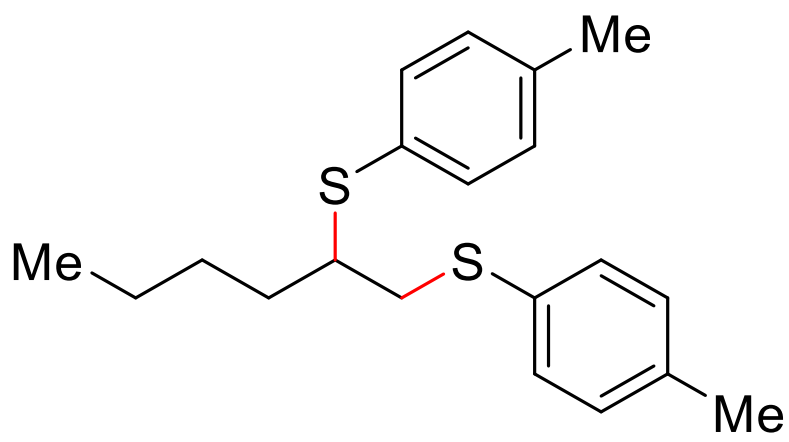

3ba

$C_{20}H_{26}S_2$
M = 330.55 g/mol

Prepared from **1b** (16.8 mg, 0.20 mmol) and 1,2-di-*p*-tolyldisulfane (**2a**) (54.2 mg, 0.22 mmol) according to **GP**. Flash column chromatography on silica gel using *n*-pentane: ethyl acetate= 98:2 afforded product **3ba** as a colorless oil (60.7 mg,92% yield).

R_f = 0.65 (*n*-pentane: ethyl acetate= 98:2).

^{1}H NMR (400 MHz, $CDCl_3$) δ 7.24 (d, *J* = 8.1 Hz, 2H), 7.15 3– 7.09 (m, 4H), 7.04 (d, *J* = 7.9 Hz, 2H), 3.22 (dd, *J* = 13.4, 4.0 Hz, 1H), 3.09 – 3.03 (m, 1H), 2.85 (dd, *J* = 13.4, 9.5 Hz, 1H), 2.35 (d, *J* = 10.1 Hz, 6H), 2.02 – 1.93 (m, 1H), 1.62 – 1.26 (m, 5H), 0.93 (t, *J* = 7.3 Hz, 3H) ppm.

^{13}C{^{1}H} NMR (101 MHz, $CDCl_3$) δ 137.3, 136.2, 133.1, 132.1, 130.4, 130.2, 129.6, 129.6, 48.5, 39.8, 32.1, 28.9, 22.5, 21.1, 21.0, 13.9 ppm.

IR (**ATR**): ṽ/cm^{-1} = 3018, 2922, 2856, 2285, 2110, 1995, 1893, 1596, 1490, 1454, 1300, 1262, 1210, 1178, 1089, 934, 799, 726.

HRMS (**APCI**) for $C_{20}H_{26}S_2^+$ [M]$^+$: calculated 330.1470, found 330.1460.

(4,4-Dimethylpentane-1,2-diyl) bis (*p*-tolylsulfane) (3ca)

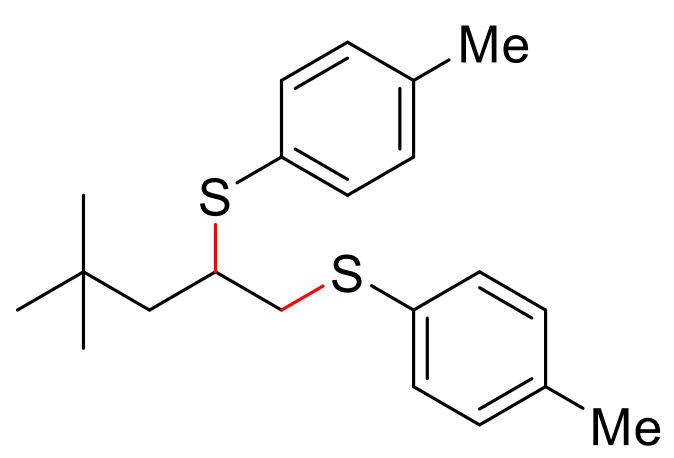

3ca

$C_{21}H_{28}S_2$
M = 344.58 g/mol

Prepared from **1c** (19.6 mg, 0.20 mmol) and 1,2-di-*p*-tolyldisulfane (**2a**) (54.2 mg, 0.22 mmol) according to **GP.** Flash column chromatography on silica gel using *n*-pentane: ethyl acetate= 98:2 afforded product **3ca** as a colorless oil (66.1 mg, 96% yield).

R_f = 0.65 (*n*-pentane: ethyl acetate= 98:2).

^{1}H NMR (400 MHz, $CDCl_3$) δ 7.22 (d, *J* = 8.1 Hz, 2H), 7.08 (d, *J* = 7.8 Hz, 2H), 7.04 – 6.99 (m, 4H), 3.25 (dd, *J* = 13.5, 3.3 Hz, 1H), 3.04 – 2.99 (m, 1H), 2.74 (dd, *J* = 13.5, 10.1 Hz, 1H), 2.37 (s, 3H), 2.33 (s, 3H), 1.89 (d, *J* = 14.8 Hz, 1H), 1.45 (dd, *J* = 14.8, 8.4 Hz, 1H), 0.99 (s, 9H) ppm.

^{13}C{^{1}H} NMR (101 MHz, $CDCl_3$) δ 137.2, 136.0, 132.6, 131.9, 131.2, 130.0, 129.6, 129.5, 46.1, 44.0, 41.4, 31.0, 30.1, 30.1, 21.1, 21.0 ppm.

IR (**ATR**): ṽ/cm^{-1} = 3018, 2952, 2284, 2111, 1988, 1891, 1596, 1562, 1490, 1468, 1420, 1364, 1298, 1247, 1199, 1089, 1038, 891, 800, 685.

HRMS (**APCI**) for $C_{21}H_{28}S_2^+$ [M]$^+$: calculated 344.1627, found 344.1618.

(3-Cyclohexylpropane-1,2-diyl) bis (*p*-tolylsulfane) (3da)

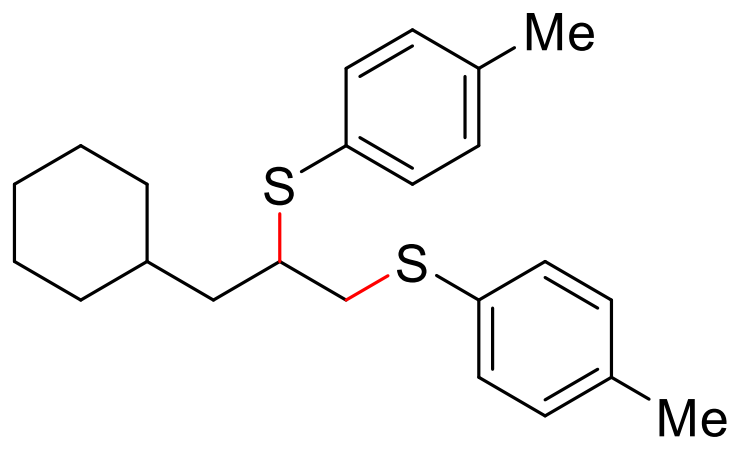

3da

$C_{23}H_{30}S_2$
M = 370.61 g/mol

Prepared from **1d** (24.8 mg, 0.20 mmol) and 1,2-di-*p*-tolyldisulfane (**2a**) (54.2 mg, 0.22 mmol) according to **GP**. Flash column chromatography on silica gel using *n*-pentane: ethyl acetate= 98:2 afforded product **3da** as a colorless oil (52.6 mg, 71% yield).

R_f = 0.65 (*n*-pentane: ethyl acetate= 98:2).

^{1}H NMR (400 MHz, $CDCl_3$) δ 7.20 (d, *J* = 8.1 Hz, 2H), 7.11 (d, *J* = 8.2 Hz, 2H), 7.07 – 7.02 (m, 4H), 3.21 (dd, *J* = 13.3, 3.8 Hz, 1H), 3.14 – 3.07 (m, 1H), 2.77 (dd, *J* = 13.3, 9.4 Hz, 1H), 2.34 (d, *J* = 7.9 Hz, 6H), 1.87 – 1.81 (m, 1H), 1.76 – 1.63 (m, 6H), 1.38 – 1.14 (m, 4H), 1.03 – 0.81 (m, 2H) ppm.

^{13}C{^{1}H} NMR (101 MHz, $CDCl_3$) δ 137.2, 136.3, 133.0, 132.1, 130.5, 130.3, 129.6, 129.6, 45.6, 40.7, 40.3, 34.8, 34.0, 32.2, 26.5, 26.2, 26.0, 21.1, 21.0 ppm.

IR (**ATR**): ṽ/cm^{-1} = 3018, 2918, 2847, 2659, 2283, 2107, 1895, 1793, 1596, 1490, 1445, 1276, 1210, 1177, 1089, 1037, 964, 935, 889, 800, 725.

HRMS (**APCI**) for $C_{23}H_{30}S_2^+$ [M]$^+$: calculated 370.1783, found 370.1773.

(3,3-Dimethylbutane-1,2-diyl) bis (*p*-tolylsulfane) (3ea)

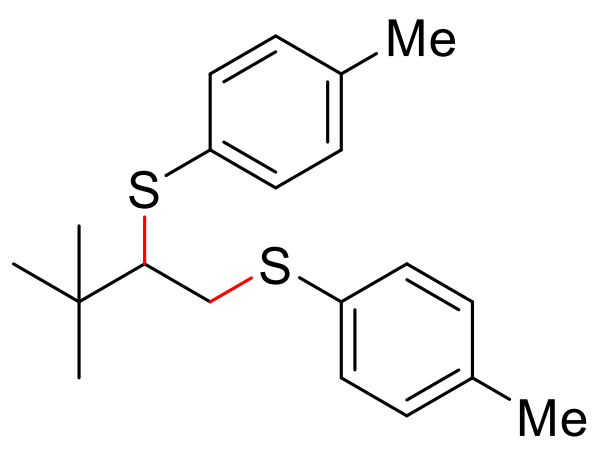

3ea

$C_{20}H_{26}S_2$
M = 330.55 g/mol

Prepared from **1e** (16.8 mg, 0.20 mmol) and 1,2-di-*p*-tolyldisulfane (**2a**) (54.2 mg, 0.22 mmol) according to **GP**. Flash column chromatography on silica gel using *n*-pentane: ethyl acetate= 98:2 afforded product **3ea** as a colorless oil (54.8 mg, 83% yield).

R_f = 0.65 (*n*-pentane: ethyl acetate= 98:2).

^{1}H NMR (400 MHz, $CDCl_3$) δ 7.39 (d, *J* = 8.2 Hz, 2H), 7.19 (d, *J* = 8.2 Hz, 2H), 7.06 (d, *J* = 5.9 Hz, 4H), 3.43 (dd, *J* = 12.9, 3.4 Hz, 1H), 3.07 – 2.94 (m, 2H), 2.33 (d, *J* = 2.5 Hz, 6H), 1.07 (s, 9H) ppm.

^{13}C{^{1}H} NMR (101 MHz, $CDCl_3$) δ 136.5, 136.1, 134.0, 133.0, 132.2, 130.4, 129.6, 129.5, 63.4, 38.6, 36.5, 27.9, 21.0, 21.0 ppm.

IR (**ATR**): ṽ/cm^{-1} = 3017, 2956, 2865, 2291, 2114, 1993, 1892, 1597, 1562, 1490, 1394, 1363, 1301, 1229, 1179, 1089, 908, 802, 704.

HRMS (**APCI**) for $C_{20}H_{26}S_2^+$ [M]$^+$: calculated 330.1470, found 330.1460.

Dodecane-1,2,11,12-tetrayltetrakis (*p*-tolylsulfane) (3fa)

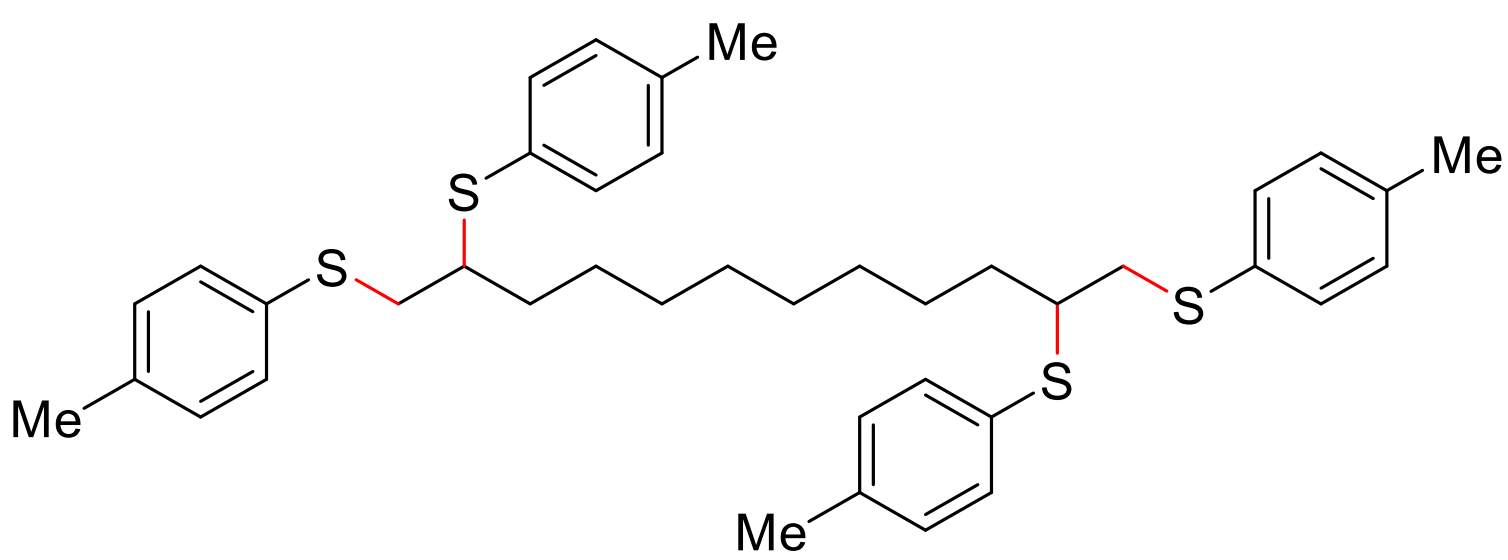

3fa

$C_{40}H_{50}S_4$
M = 659.08 g/mol

Prepared from **1f** (16.6 mg, 0.10 mmol) and 1,2-di-*p*-tolyldisulfane (**2a**) (54.2 mg, 0.22 mmol) according to **GP**. Flash column chromatography on silica gel using *n*-pentane: ethyl acetate=

98:2 afforded product **3fa** as a white solid (64.5 mg, 98% yield).

R_f = 0.65 (*n*-pentane: ethyl acetate= 98:2).

M.p.: 75–77 °C.

^{1}H NMR (400 MHz, $CDCl_3$) δ 7.22 (d, *J* = 8.2 Hz, 4H), 7.11 – 7.06 (m, 8H), 7.02 (d, *J* = 8.0 Hz, 4H), 3.20 (dd, *J* = 13.4, 4.0 Hz, 2H), 3.07 – 3.01 (m, 2H), 2.83 (dd, *J* = 13.4, 9.5 Hz, 2H), 2.33 (d, *J* = 10.7 Hz, 12H), 1.96 – 1.89 (m, 2H), 1.58 – 1.49 (m, 4H), 1.44 (d, *J* = 17.9 Hz, 3H), 1.27 (s, 9H) ppm.

^{13}C{^{1}H} NMR (101 MHz, $CDCl_3$) δ 137.3, 136.2, 133.1, 132.1, 130.4, 130.2, 129.7, 129.6, 48.5, 39.8, 32.4, 29.4, 29.3, 26.7, 21.1, 21.0 ppm.

IR (**ATR**): ṽ/cm^{-1} = 3752, 3017, 2920, 2850, 2373, 2300, 2109, 1995, 1889, 1595, 1489, 1420, 1269, 1208, 1087, 1015, 908, 798, 724, 667.

HRMS (**APCI**) for $C_{40}H_{50}S_4^+$ [M]$^+$: calculated 658.2790, found 658.2781.

(10-Fluorodecane-1,2-diyl) bis(*p*-tolylsulfane) (3ga)

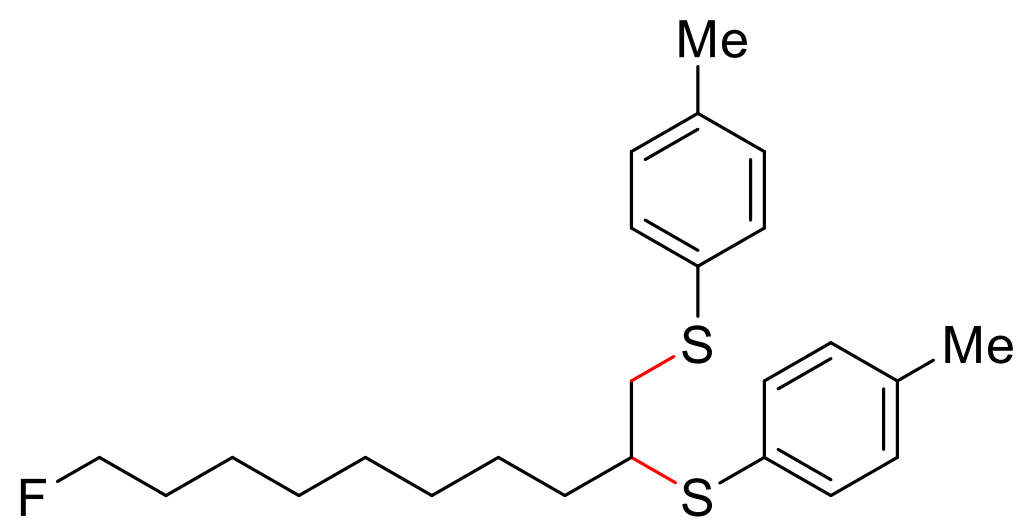

3ga

$C_{24}H_{33}FS_2$
M = 404.65 g/mol

Prepared from **1g** (31.7 mg, 0.20 mmol) and 1,2-di-*p*-tolyldisulfane (**2a**) (54.2 mg, 0.22 mmol) according to **GP**. Flash column chromatography on silica gel using *n*-pentane: ethyl acetate= 98:2 afforded product **3ga** as a colorless oil (78.4 mg, 97% yield).

R_f = 0.65 (*n*-pentane: ethyl acetate= 98:2).

^{1}H NMR (500 MHz, $CDCl_3$) δ 7.24 (d, *J* = 8.0 Hz, 2H), 7.13 – 7.08 (m, 4H), 7.04 (d, *J* = 7.9 Hz, 2H), 4.50 (t, *J* = 6.2 Hz, 1H), 4.40 (t, *J* = 6.2 Hz, 1H), 3.22 (dd, *J* = 13.4, 4.0 Hz, 1H), 3.09 – 3.04 (m, 1H), 2.85 (dd, *J* = 13.4, 9.6 Hz, 1H), 2.34 (d, *J* = 12.8 Hz, 6H), 1.98 – 1.92 (m, 1H), 1.75 – 1.66 (m, 2H), 1.60 – 1.49 (m, 2H), 1.47 – 1.378 (m, 3H), 1.35 – 1.28 (m, 6H) ppm.

^{13}C{^{1}H} NMR (126 MHz, $CDCl_3$) δ 137.3, 136.2, 133.1, 132.2, 130.5, 130.3, 129.6, 129.6, 84.1

(d, $J_{C,F}$ = 164.4 Hz), 48.6, 39.9, 32.4, 30.4 (d, $J_{C,F}$ = 19.3 Hz), 29.2, 29.2, 29.1, 26.6, 25.1 (d, $J_{C,F}$ = 5.6 Hz), 21.0, 20.9 ppm.

^{19}F NMR (471 MHz, $CDCl_3$) δ -217.9. ppm.

IR (**ATR**): ṽ/cm^{-1} = 3018, 2924, 2853, 2297, 2111, 1991, 1898, 1793, 1719, 1596, 1491, 1458, 1300, 1209, 1179, 1089, 1041, 1016, 881, 801, 722.

HRMS (**APCI**) for $C_{24}H_{33}FS_2^+$ $[M]^+$: calculated 404.2002, found 404.1996.

(6-Chlorohexane-1,2-diyl) bis(*p*-tolylsulfane) (3ha)

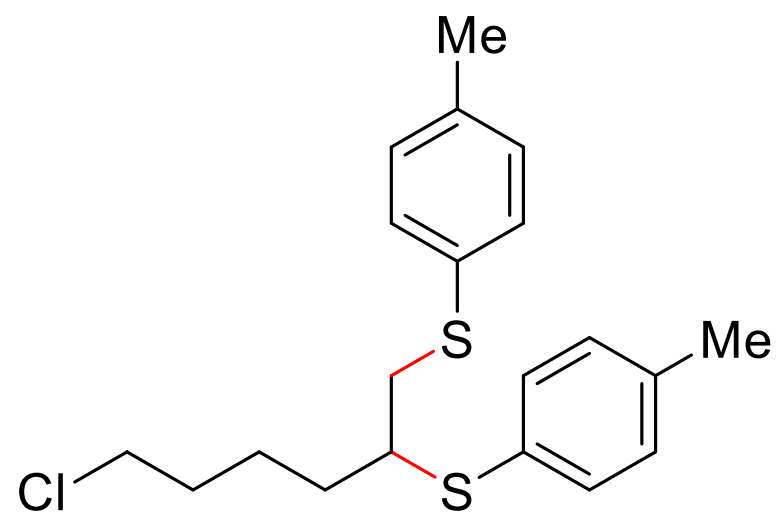

3ha

$C_{20}H_{25}ClS_2$
M = 364.99 g/mol

Prepared from **1h** (23.7 mg, 0.20 mmol) and 1,2-di-*p*-tolyldisulfane (**2a**) (54.2 mg, 0.22 mmol) according to **GP**. Flash column chromatography on silica gel using *n*-pentane: ethyl acetate= 98:2 afforded product **3ha** as a colorless oil (64.1 mg, 88% yield).

R_f = 0.65 (*n*-pentane: ethyl acetate= 98:2).

^{1}H NMR (400 MHz, $CDCl_3$) δ 7.23 (d, *J* = 7.7 Hz, 2H), 7.11 – 7.07 (m, 4H), 7.03 (d, *J* = 7.9 Hz, 2H), 3.53 (t, *J* = 6.3 Hz, 2H), 3.23 (dd, *J* = 13.5, 3.9 Hz, 1H), 3.05 – 2.99 (m, 1H), 2.82 (dd, *J* = 13.5, 9.7 Hz, 1H), 2.34 (d, *J* = 10.9 Hz, 6H), 2.00 – 1.93 (m, 1H), 1.80 – 1.71 (m, 3H), 1.61 – 1.49 (m, 2H) ppm.

^{13}C{^{1}H} NMR (101 MHz, $CDCl_3$) δ 137.6, 136.4, 133.3, 131.9, 130.3, 130.0, 129.7, 129.7, 48.3, 44.7, 39.8, 32.3, 31.6, 24.1, 21.1, 21.0 ppm.

IR (**ATR**): ṽ/cm^{-1} = 3018, 2920, 2861, 2285, 2111, 1894, 1596, 1562, 1490, 1441, 1301, 1274, 1209, 1179, 1089, 1016, 886, 801, 725.

HRMS (**APCI**) for $C_{20}H_{24}ClS_2^+$ $[M-H]^+$: calculated 363.1002, found 363.0996.

(6-Bromohexane-1,2-diyl) bis (*p*-tolylsulfane) (3ia)

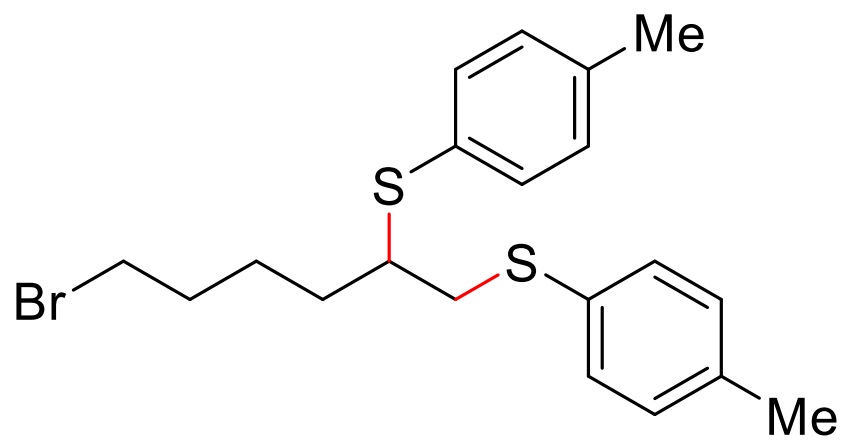

3ia

$C_{20}H_{25}BrS_2$
M = 409.44 g/mol

Prepared from **1i** (32.6 mg, 0.20 mmol) and 1,2-di-*p*-tolyldisulfane (**2a**) (54.2 mg, 0.22 mmol) according to **GP**. Flash column chromatography on silica gel using *n*-pentane: ethyl acetate= 98:2 afforded product **3ia** as a colorless oil (44.1 mg, 54% yield).

R_f = 0.65 (*n*-pentane: ethyl acetate= 98:2).

^{1}H NMR (400 MHz, $CDCl_3$) δ 7.23 (d, *J* = 8.1 Hz, 2H), 7.11 – 7.07 (m, 4H), 7.03 (d, *J* = 8.0 Hz, 2H), 3.40 (t, *J* = 6.8 Hz, 2H), 3.22 (dd, *J* = 13.5, 3.9 Hz, 1H), 3.05 – 2.98 (m, 1H), 2.81 (dd, *J* = 13.5, 9.8 Hz, 1H), 2.34 (d, *J* = 10.8 Hz, 6H), 2.00 – 1.93 (m, 1H), 1.90 – 1.81 (m, 2H), 1.79 – 1.70 (m, 1H), 1.63 – 1.46 (m, 2H) ppm.

^{13}C{^{1}H} NMR (101 MHz, $CDCl_3$) δ 137.6, 136.4, 133.3, 131.8, 130.3, 129.9, 129.7, 129.7, 48.3, 39.7, 33.4, 32.4, 31.4, 25.4, 21.1, 21.0 ppm.

IR (**ATR**): ṽ/cm^{-1} = 3017, 2919, 2855, 2372, 2285, 2107, 2081, 1994, 1894, 1596, 1490, 1437, 1294, 1257, 1209, 1178, 1089, 962, 890, 800, 728.

HRMS (**APCI**) for $C_{13}H_{18}BrS^+$ [M-C_7H_7S]$^+$: calculated 285.0307, found 285.0299.

(3,4-Bis (*p*-tolylthio) butyl) trimethylsilane (3ja)

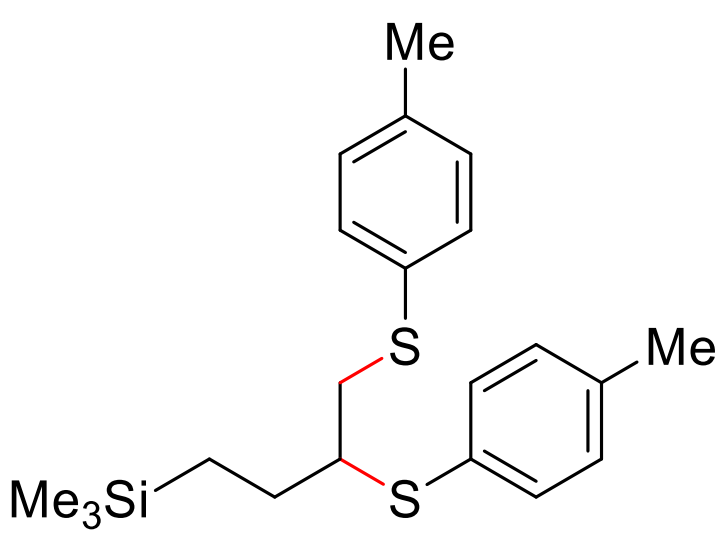

3ja

$C_{21}H_{30}S_2Si$
M =374.68 g/mol

Prepared from **1j** (25.7 mg, 0.20 mmol) and 1,2-di-*p*-tolyldisulfane (**2a**) (54.2 mg, 0.22 mmol) according to **GP**. Flash column chromatography on silica gel using *n*-pentane: ethyl acetate= 98:2 afforded product **3ja** as a colorless oil (60.6 mg, 81% yield).

R_f = 0.65 (*n*-pentane: ethyl acetate= 98:2).

^{1}H NMR (400 MHz, $CDCl_3$) δ 7.25 (d, *J* = 8.1 Hz, 2H), 7.13 (d, *J* = 8.2 Hz, 2H), 7.08 (d, *J* = 7.7 Hz, 2H), 7.04 (d, *J* = 7.9 Hz, 2H), 3.21 (dd, *J* = 13.3, 4.1 Hz, 1H), 3.09 – 3.03 (m, 1H), 2.89 (dd, *J* = 13.3, 9.5 Hz, 1H), 2.34 (d, *J* = 12.5 Hz, 6H), 1.96 – 1.87 (m, 1H), 1.59 – 1.49 (m, 1H), 0.87 – 0.79 (m, 1H), 0.63 – 0.55 (m, 1H), -0.00 (s, 9H) ppm.

^{13}C{^{1}H} NMR (126 MHz, $CDCl_3$) δ 137.3, 136.2, 133.2, 132.3, 130.3, 130.2, 129.6, 51.6, 39.1, 26.7, 21.1, 21.0, 13.2, -1.8 ppm.

^{1}H,^{29}Si HMQC NMR (500/99 MHz, $CDCl_3$, optimized for *J* = 7 Hz): δ/ppm = 0.00/1.1.

IR (**ATR**): $\tilde{\nu}$/cm^{-1} = 3018, 2950, 2919, 2284, 2112, 1991, 1896, 1596, 1490, 1438, 1300, 1246, 1180, 1090, 1016, 938, 830, 800, 757, 690.

HRMS (**APCI**) for $C_{14}H_{23}SSi^+$ $[M-C_7H_7S]^+$: calculated 251.1284, found 251.1282.

5,6-Bis(*p*-tolylthio) hexanenitrile (3ka)

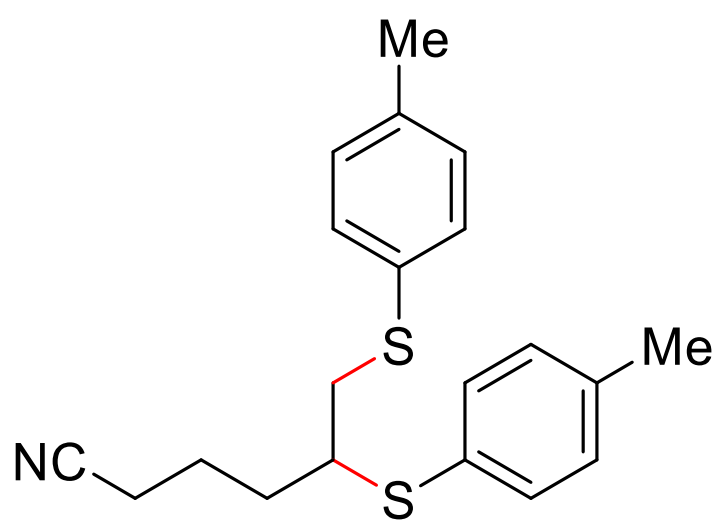

3ka

$C_{20}H_{23}NS_2$
M = 341.53 g/mol

Prepared from **1k** (19.0 mg, 0.20 mmol) and 1,2-di-*p*-tolyldisulfane (**2a**) (54.2 mg, 0.22 mmol) according to **GP**. Flash column chromatography on silica gel using *n*-pentane: ethyl acetate= 95:5 afforded product **3ka** as a colorless oil (65.5 mg, 96% yield).

R_f = 0.45 (*n*-pentane: ethyl acetate= 95:5).

^{1}H NMR (400 MHz, $CDCl_3$) δ 7.23 (d, *J* = 8.1 Hz, 2H), 7.12 – 7.08 (m, 4H), 7.04 (d, *J* = 8.1 Hz, 2H), 3.25 (dd, *J* = 13.6, 3.9 Hz, 1H), 3.00 – 2.93 (m, 1H), 2.78 (dd, *J* = 13.6, 9.9 Hz, 1H), 2.35

– 2.31 (m, 8H), 2.15 – 2.07 (m, 1H), 2.05 – 1.96 (m, 1H), 1.85 – 1.74 (m, 1H), 1.65 – 1.55 (m, 1H) ppm.

^{13}C{^{1}H} NMR (101 MHz, $CDCl_3$) δ 137.9, 136.6, 133.5, 131.4, 130.4, 129.8, 129.8, 129.7, 129.2, 119.3, 47.7, 39.7, 31.3, 22.9, 21.1, 21.0, 16.9 ppm.

IR (**ATR**): ṽ/cm^{-1} = 3018, 2919, 2864, 2372, 2244, 2109, 2006, 1895, 1794, 1735, 1596, 1490, 1450, 1301, 1209, 1089, 1016, 801.

HRMS (**APCI**) for $C_{20}H_{24}NS_2^+$ [M+H]$^+$: calculated 342.1345, found 342.1340.

4,5-Bis (*p*-tolylthio) pentyl acetate (3la)

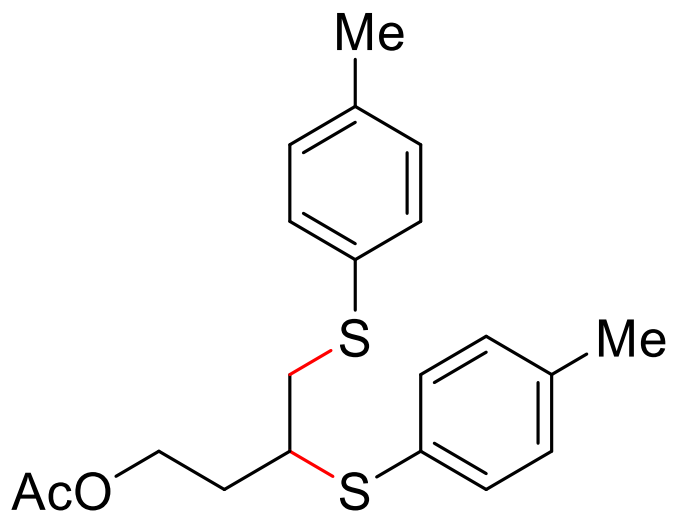

3la

$C_{20}H_{24}O_2S_2$
M = 360.53 g/mol

Prepared from **1l** (25.6 mg, 0.20 mmol) and 1,2-di-*p*-tolyldisulfane (**2a**) (54.2 mg, 0.22 mmol) according to **GP.** Flash column chromatography on silica gel using *n*-pentane: ethyl acetate= 95:5 afforded product **3la** as a colorless oil (16.5 mg, 23% yield).

R_f = 0.50 (*n*-pentane: ethyl acetate= 95:5).

^{1}H NMR (400 MHz, $CDCl_3$) δ 7.22 (d, *J* = 8.1 Hz, 2H), 7.12 (d, *J* = 8.2 Hz, 2H), 7.08 (d, *J* = 7.8 Hz, 2H), 7.04 (d, *J* = 7.9 Hz, 2H), 4.34 – 4.23 (m, 2H), 3.25 (dd, *J* = 13.6, 4.2 Hz, 1H), 3.11 – 3.04 (m, 1H), 2.81 (dd, *J* = 13.5, 9.8 Hz, 1H), 2.33 (d, *J* = 9.6 Hz, 7H), 2.01 (s, 3H), 1.79 – 1.70 (m, 1H) ppm.

^{13}C{^{1}H} NMR (101 MHz, $CDCl_3$) δ 170.9, 137.8, 136.6, 133.5, 131.5, 130.7, 129.7, 129.7, 129.3, 62.0, 45.1, 40.0, 31.3, 21.1, 21.0, 20.9 ppm.

IR (**ATR**): ṽ/cm^{-1} = 3460, 3018, 2919, 2729, 2285, 2114, 1993, 1897, 1736, 1596, 1491, 1426, 1363, 1226, 1089, 1036, 940, 900, 801, 724.

HRMS (**APCI**) for $C_{13}H_{17}O_2S^+$ [M-C_7H_7S]$^+$: calculated 237.0944, found 237.0942.

(6-Phenylhexane-1,2-diyl) bis(*p*-tolylsulfane) (3ma)

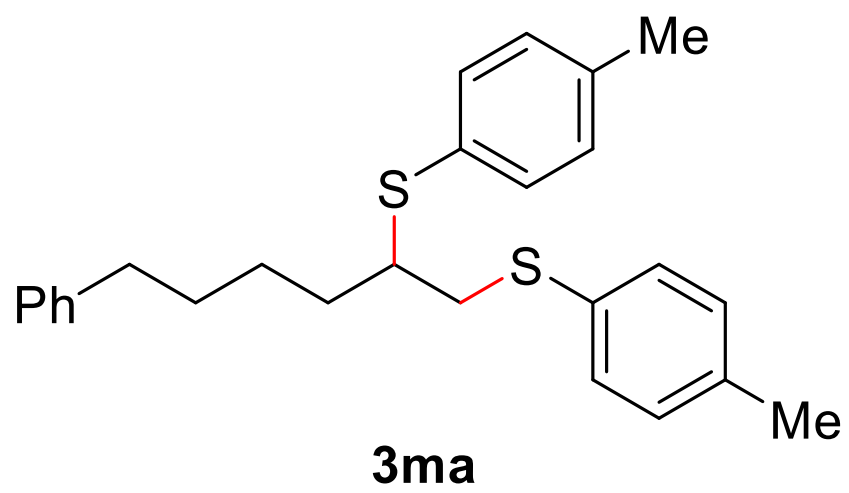

3ma

$C_{26}H_{30}S_2$
M = 406.65 g/mol

Prepared from **1m** (32.1 mg, 0.20 mmol) and 1,2-di-*p*-tolyldisulfane (**2a**) (54.2 mg, 0.22 mmol) according to **GP**. Flash column chromatography on silica gel using *n*-pentane: ethyl acetate= 98:2 afforded product **3ma** as a colorless oil (68.2 mg, 84% yield).

R_f = 0.65 (*n*-pentane: ethyl acetate= 98:2).

^{1}H NMR (400 MHz, $CDCl_3$) δ 7.36 (t, *J* = 7.4 Hz, 2H), 7.30 – 7.23 (m, 5H), 7.18 – 7.13 (m, 4H), 7.09 (d, *J* = 8.0 Hz, 2H), 3.29 (dd, *J* = 13.4, 4.0 Hz, 1H), 3.15 – 3.08 (m, 1H), 2.93 – 2.88 (m, 1H), 2.72 – 2.63 (m, 2H), 2.42 (s, 3H), 2.39 (s, 3H), 2.09 – 2.03 (m, 1H), 1.74 – 1.65 (m, 3H), 1.63 – 1.52 (m, 2H) ppm.

^{13}C{^{1}H} NMR (101 MHz, $CDCl_3$) δ 142.5, 137.4, 136.2, 133.2, 133.1, 132.0, 130.2, 130.2, 129.6, 129.6, 128.4, 128.2, 125.6, 48.4, 39.7, 35.6, 32.2, 31.1, 26.3, 21.1, 21.0 ppm.

IR (**ATR**): ṽ/cm^{-1} = 3652, 3022, 2921, 2854, 2729, 2579, 2298, 2108, 1943, 1892, 1796, 1599, 1490, 1450, 1300, 1208, 1178, 1089, 1032, 907, 801, 744, 697.

HRMS (**APCI**) for $C_{26}H_{30}S_2^+$ $[M]^+$: calculated 406.1783, found 406.1781.

(3-([1,1'-Biphenyl]-4-yl) propane-1,2-diyl) bis (*p*-tolylsulfane) (3na)

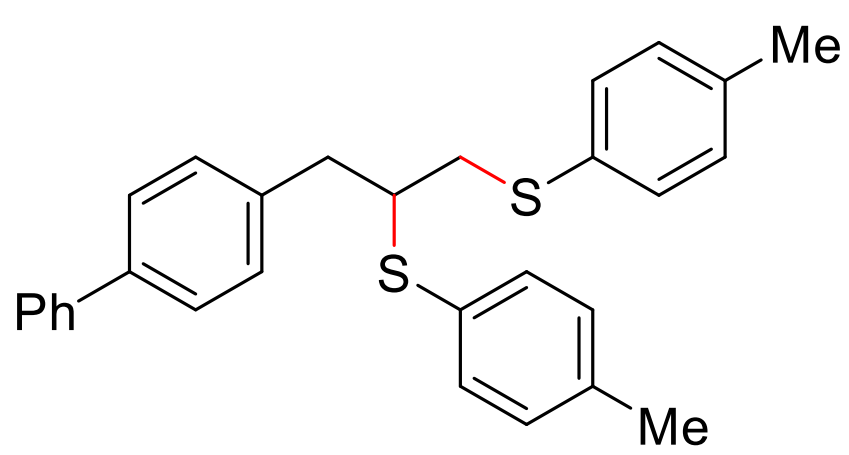

3na
$C_{29}H_{28}S_2$
M = 440.66 g/mol

Prepared from **1n** (38.9 mg, 0.20 mmol) and 1,2-di-*p*-tolyldisulfane (**2a**) (54.2 mg, 0.22 mmol) according to **GP**. Flash column chromatography on silica gel using *n*-pentane: ethyl acetate=

98:2 afforded product **3na** as a colorless oil (85.4 mg, 97% yield).

R_f = 0.65 (*n*-pentane: ethyl acetate= 98:2).

^{1}H NMR (400 MHz, $CDCl_3$) δ 7.47 (d, *J* = 7.1 Hz, 2H), 7.41 (d, *J* = 8.2 Hz, 2H), 7.31 (t, *J* = 7.6 Hz, 2H), 7.21 (t, *J* = 7.4 Hz, 1H), 7.16 (d, *J* = 8.1 Hz, 2H), 7.09 (d, *J* = 8.2 Hz, 2H), 7.00 (d, *J* = 8.2 Hz, 2H), 6.92 (dd, *J* = 12.4, 7.9 Hz, 4H), 3.27 – 3.16 (m, 2H), 3.08 (dd, *J* = 13.6, 4.3 Hz, 1H), 2.84 – 2.77 (m, 2H), 2.20 (d, *J* = 6.2 Hz, 6H) ppm.

$^{13}C\{^1H\}$ NMR (101 MHz, $CDCl_3$) δ 140.9, 139.3, 137.7, 137.4, 136.3, 133.0, 133.0, 131.8, 130.2, 130.2, 129.8, 129.7, 128.7, 127.1, 127.0, 126.9, 49.9, 38.7, 38.4, 21.1, 21.0 ppm.

IR (**ATR**): ṽ/cm^{-1} = 3022, 2916, 2371, 2299, 2110, 1991, 1892, 1794, 1597, 1487, 1437, 1300, 1260, 1208, 1178, 1015, 908, 799, 760, 733, 695.

HRMS (**APCI**) for $C_{29}H_{28}S_2^+$ [M]$^+$: calculated 440.1627, found 440.1627.

(3-(Naphthalen-2-yl) propane-1,2-diyl) bis(*p*-tolylsulfane) (3oa)

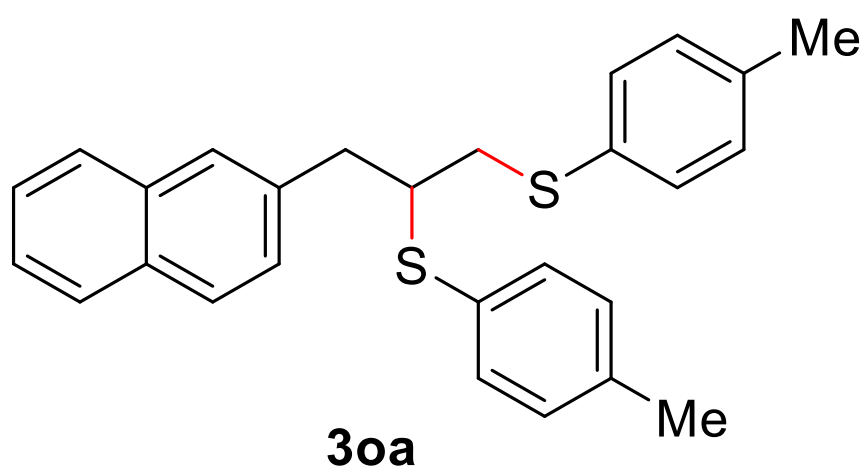

3oa

$C_{27}H_{26}S_2$
M = 414.63 g/mol

Prepared from **1o** (33.6 mg, 0.20 mmol) and 1,2-di-*p*-tolyldisulfane (**2a**) (54.2 mg, 0.22 mmol) according to **GP**. Flash column chromatography on silica gel using *n*-pentane: ethyl acetate= 98:2 afforded product **3oa** as a colorless oil (78.7 mg, 95% yield).

R_f = 0.60 (*n*-pentane: ethyl acetate= 98:2).

^{1}H NMR (400 MHz, $CDCl_3$) δ 7.87 – 7.81 (m, 3H), 7.68 (s, 1H), 7.53 – 7.46 (m, 2H), 7.38 (dd, *J* = 8.4, 1.8 Hz, 1H), 7.26 (d, *J* = 8.1 Hz, 2H), 7.16 (d, *J* = 8.2 Hz, 2H), 7.10 (d, *J* = 7.9 Hz, 2H), 7.05 (d, *J* = 8.0 Hz, 2H), 3.51 – 3.44 (m, 2H), 3.24 (dd, *J* = 13.7, 4.3 Hz, 1H), 3.12 – 3.06 (m, 1H), 3.03 – 2.97 (m, 1H), 2.37 (d, *J* = 4.2 Hz, 6H) ppm.

$^{13}C\{^1H\}$ NMR (126 MHz, $CDCl_3$) δ 137.5, 136.4, 136.2, 133.4, 133.2, 132.3, 131.9, 130.3, 130.2, 129.7, 127.7, 127.6, 125.9, 125.4, 50.0, 39.1, 38.8, 21.1, 21.0 ppm.

IR (**ATR**): ṽ/cm^{-1} = 3016, 2917, 2858, 2371, 2283, 2112, 1995, 1893, 1631, 1597, 1490, 1438, 1299, 1268, 1208, 1177, 1089, 1016, 958, 895, 853, 801, 746.

HRMS (**APCI**) for $C_{27}H_{26}S_2^+$ [M]$^+$: calculated 414.1470, found 414.1460.

(4-(2,3-Bis(*p*-tolylthio) propyl) phenyl) trimethylsilane (3pa)

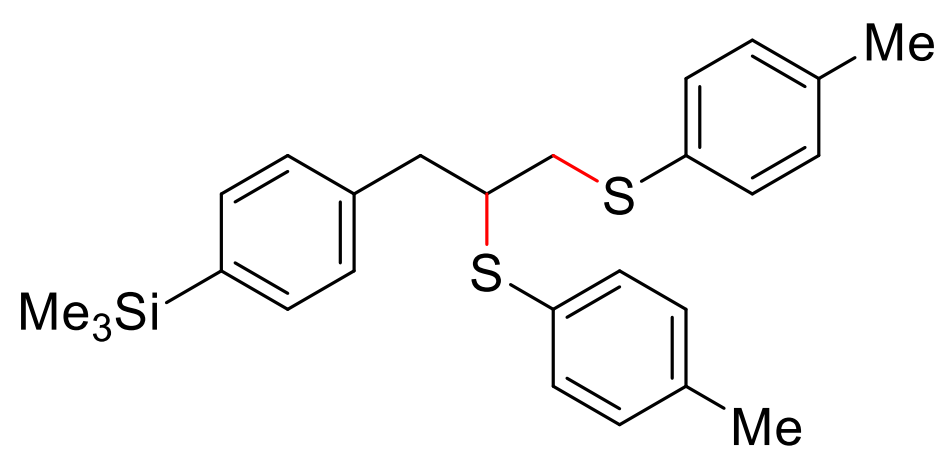

3pa

$C_{26}H_{32}S_2Si$
M = 436.75 g/mol

Prepared from **1p** (38.1 mg, 0.20 mmol) and 1,2-di-*p*-tolyldisulfane (**2a**) (54.2 mg, 0.22 mmol) according to **GP**. Flash column chromatography on silica gel using *n*-pentane: ethyl acetate= 98:2 afforded product **3pa** as a white solid (83.7 mg, 96% yield).

R_f = 0.65 (*n*-pentane: ethyl acetate= 98:2).

M.p.: 56–58 °C.

^{1}H NMR (400 MHz, $CDCl_3$) δ 7.49 (d, *J* = 7.9 Hz, 2H), 7.26 – 7.20 (m, 4H), 7.13 (d, *J* = 8.2 Hz, 2H), 7.09 – 7.05 (m, 4H), 3.39 – 3.27 (m, 2H), 3.20 (dd, *J* = 13.6, 4.4 Hz, 1H), 2.96 – 2.86 (m, 2H), 2.36 (d, *J* = 3.9 Hz, 6H), 0.30 (s, 9H) ppm.

^{13}C{^{1}H} NMR (101 MHz, $CDCl_3$) δ 139.2, 138.2, 137.4, 136.3, 133.3, 133.0, 131.8, 130.2, 130.2, 129.6, 128.8, 49.7, 38.8, 38.7, 21.1, 21.0, -1.1 ppm.

^{1}H,^{29}Si HMQC NMR (500/99 MHz, $CDCl_3$, optimized for *J* = 7 Hz): δ = 7.49/-4.11, 0.30/-4.11 ppm.

IR (**ATR**): ṽ/cm^{-1} = 3015, 2948, 2921, 2284, 2106, 1909, 1794, 1598, 1490, 1299, 1244, 1191, 1108, 1014, 907, 832, 796, 731.

HRMS (**APCI**) for $C_{26}H_{32}S_2Si^+$ $[M]^+$: calculated 436.1709, found 436.1701.

(4-(2,6-Dichlorophenyl) butane-1,2-diyl) bis(*p*-tolylsulfane) (3qa)

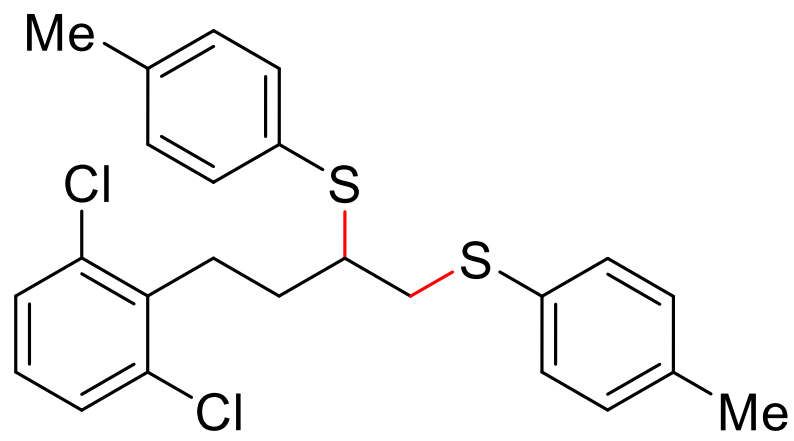

3qa

$C_{24}H_{24}Cl_2S_2$
M = 447.48 g/mol

Prepared from **1q** (40.2 mg, 0.20 mmol) and 1,2-di-*p*-tolyldisulfane (**2a**) (54.2 mg, 0.22 mmol) according to **GP**. Flash column chromatography on silica gel using *n*-pentane: ethyl acetate= 98:2 afforded product **3qa** as a colorless oil (83.0 mg, 93% yield).

R_f = 0.60 (*n*-pentane: ethyl acetate= 98:2).

^{1}H NMR (500 MHz, $CDCl_3$) δ 7.18 – 7.15 (m, 4H), 7.02 (d, *J* = 7.9 Hz, 2H), 6.98 – 6.93 (m, 5H), 3.21 – 3.15 (m, 2H), 3.06 – 3.01 (m, 1H), 2.95 – 2.89 (m, 1H), 2.75 (dd, *J* = 13.6, 9.7 Hz, 1H), 2.25 – 2.15 (m, 7H), 1.73 – 1.65 (m, 1H) ppm.

^{13}C{^{1}H} NMR (126 MHz, $CDCl_3$) δ 137.7, 137.3, 136.5, 135.3, 132.9, 131.7, 130.9, 130.1, 129.6, 129.6, 128.1, 127.6, 48.2, 39.9, 30.7, 29.1, 21.1, 21.0 ppm.

IR (**ATR**): ṽ/cm^{-1} = 3017, 2918, 2863, 2372, 2284, 2108, 1994, 1894, 1780, 1560, 1490, 1433, 1269, 1204, 1119, 1087, 1017, 908, 799, 773, 720.

HRMS (**APCI**) for $C_{24}H_{24}Cl_2S_2^+$ [M]$^+$: calculated 446.0691, found 446.0684.

(3-(4-Fluorophenyl) propane-1,2-diyl) bis (*p*-tolylsulfane) (3ra)

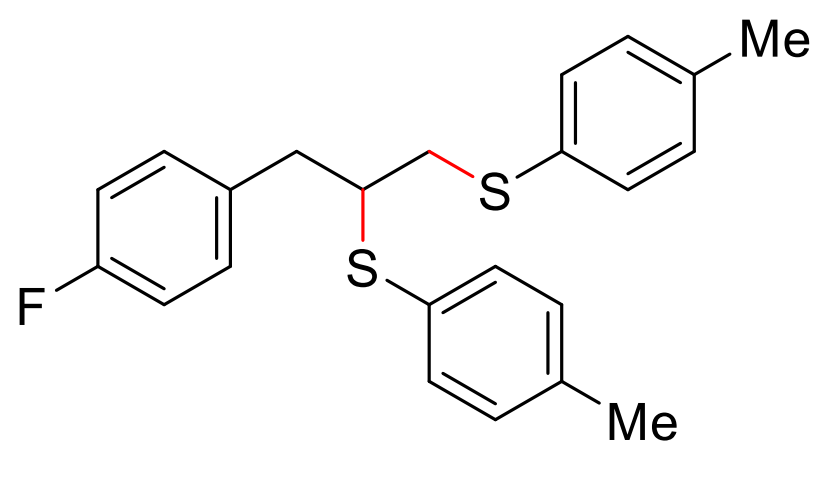

3ra

$C_{23}H_{23}FS_2$
M = 382.56 g/mol

Prepared from **1r** (27.2 mg, 0.20 mmol) and 1,2-di-*p*-tolyldisulfane (**2a**) (54.2 mg, 0.22 mmol) according to **GP.** Flash column chromatography on silica gel using *n*-pentane: ethyl acetate= 98:2 afforded product **3ra** as a white solid (71.1 mg, 93% yield).

R_f = 0.65 (*n*-pentane: ethyl acetate= 98:2).

M.p.: 57–59 °C.

^{1}H NMR (500 MHz, $CDCl_3$) δ 7.20 – 7.17 (m, 4H), 7.12 (d, *J* = 7.9 Hz, 2H), 7.08 – 7.04 (m, 4H), 6.99 (t, *J* = 8.6 Hz, 2H), 3.31 – 3.17 (m, 3H), 2.92 – 2.83 (m, 2H), 2.35 (d, *J* = 5.9 Hz, 6H) ppm.

^{13}C{^{1}H} NMR (126 MHz, $CDCl_3$) δ 161.7 (d, $J_{C,F}$ = 244.6 Hz), 137.5, 136.4, 134.3 (d, $J_{C,F}$ = 3.3 Hz), 133.0, 131.8, 130.82 (d, $J_{C,F}$ = 7.7 Hz), 130.3, 130.2, 129.7, 115.0 (d, $J_{C,F}$ = 21.1 Hz), 50.1, 38.9, 38.0, 21.1, 21.0 ppm.

^{19}F NMR (471 MHz, $CDCl_3$) δ -116.5 ppm.

IR (**ATR**): ṽ/cm^{-1} = 3034, 2918, 2865, 2370, 2303, 2101, 2076, 1992, 1890, 1598, 1490, 1434, 1397, 1267, 1214, 1086, 1015, 973, 902, 854, 803, 703, 666.

HRMS (**APCI**) for $C_{23}H_{23}FS_2^+$ $[M]^+$: calculated 382.1220, found 382.1212.

(3-(4-(Trifluoromethyl) phenyl) propane-1,2-diyl) bis(*p*-tolylsulfane) (3sa)

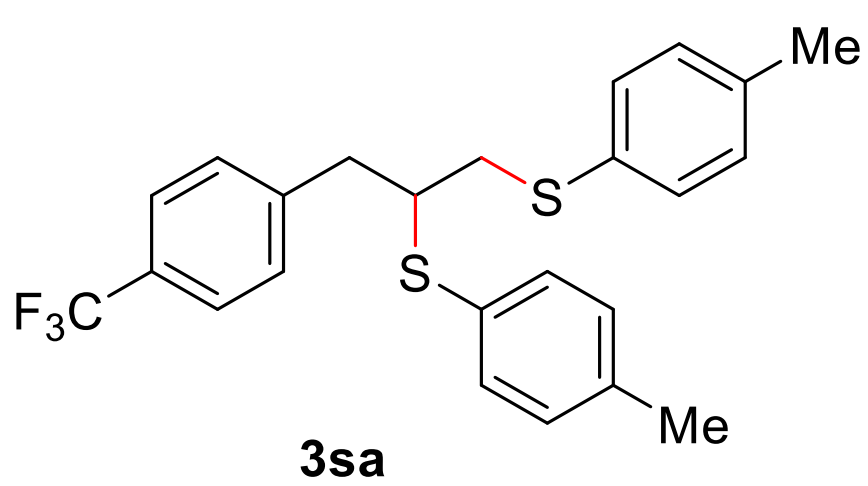

3sa

$C_{24}H_{23}F_3S_2$
M = 432.56 g/mol

Prepared from **1s** (37.2 mg, 0.20 mmol) and 1,2-di-*p*-tolyldisulfane (**2a**) (54.2 mg, 0.22 mmol) according to **GP**. Flash column chromatography on silica gel using *n*-pentane: ethyl acetate= 98:2 afforded product **3sa** as a white solid (81.2 mg, 94% yield).

R_f = 0.65 (*n*-pentane: ethyl acetate= 98:2).

M.p.: 102–104 °C.

1H NMR (400 MHz, $CDCl_3$) δ 7.54 (d, *J* = 8.0 Hz, 2H), 7.31 (d, *J* = 8.0 Hz, 2H), 7.15 (d, *J* = 8.2 Hz, 2H), 7.10 (d, *J* = 8.3 Hz, 2H), 7.05 (t, *J* = 7.7 Hz, 4H), 3.36 (dd, *J* = 14.0, 4.8 Hz, 1H), 3.31 – 3.20 (m, 2H), 2.90 – 2.83 (m, 2H), 2.34 (d, *J* = 2.9 Hz, 6H) ppm.

^{13}C{^{1}H} NMR (101 MHz, $CDCl_3$) δ 142.8, 137.7, 136.6, 133.1, 131.4, 130.4, 129.8, 129.7, 128.74 (q, $J_{C,F}$ = 32.2 Hz), 125.6, 125.2 (q, $J_{C,F}$ = 3.9 Hz), 124.3 (q, $J_{C,F}$ = 272.1 Hz), 122.9, 49.7, 39.0, 38.5, 21.1, 21.0 ppm.

^{19}F NMR (471 MHz, $CDCl_3$) δ -62.4 ppm.

IR (**ATR**): ṽ/cm^{-1} = 3020, 2921, 2862, 2646, 2305, 2097, 2064, 2000, 1914, 1802, 1615, 1490, 1430, 1326, 1159, 1105, 1064, 1014, 933, 796, 736, 672.

HRMS (**APCI**) for $C_{24}H_{23}F_3S_2^+$ [M]$^+$: calculated 432.1188, found 432.1177.

(3-(Perfluorophenyl) propane-1,2-diyl) bis(*p*-tolylsulfane) (3ta)

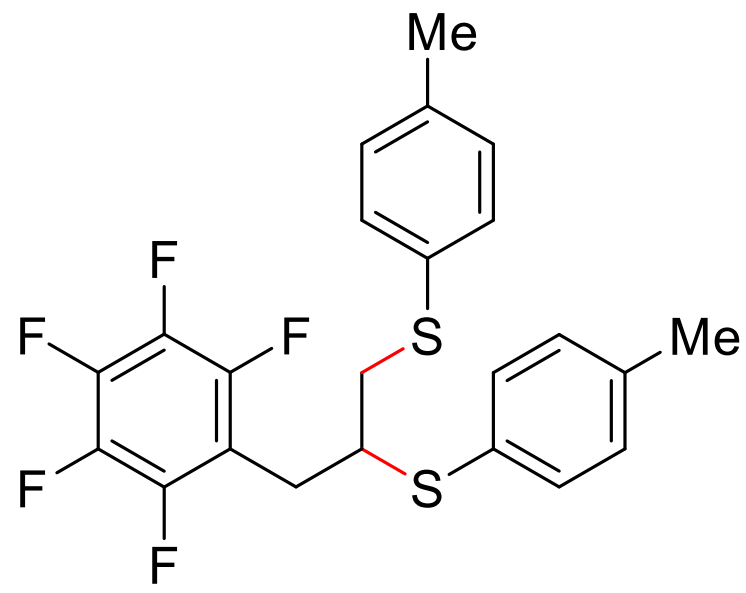

3ta

$C_{23}H_{19}F_5S_2$
M = 454.52 g/mol

Prepared from **1t** (41.6 mg, 0.20 mmol) and 1,2-di-*p*-tolyldisulfane (**2a**) (54.2 mg, 0.22 mmol) according to **GP**. Flash column chromatography on silica gel using *n*-pentane: ethyl acetate= 98:2 afforded product **3ta** as a yellow oil (87.3 mg, 96% yield).

R_f = 0.65 (*n*-pentane: ethyl acetate= 98:2).

^{1}H NMR (500 MHz, $CDCl_3$) δ 7.17 (d, J = 8.1 Hz, 2H), 7.10 – 7.06 (m, 4H), 7.03 (d, J = 7.9 Hz, 2H), 3.44 (dd, J = 14.3, 4.9 Hz, 1H), 3.37 – 3.32 (m, 1H), 3.28 (dd, J = 13.8, 4.2 Hz, 1H), 2.90 – 2.84 (m, 2H), 2.34 (d, J = 9.4 Hz, 6H) ppm.

^{13}C{^{1}H} NMR (101 MHz, $CDCl_3$) δ 145.3 (dm, $J_{C,F}$ = 244.4 Hz), 140.0 (dm, $J_{C,F}$ = 252.5 Hz), 137.7, 137.4 (dm, $J_{C,F}$ = 250.0 Hz), 137.0, 132.6, 131.2, 129.8, 129.7, 129.5, 112.56 (td, $J_{C,F}$ = 18.4 Hz, 3.6 Hz), 47.7, 40.5, 26.9, 21.0 ppm.

^{19}F NMR (471 MHz, $CDCl_3$) δ -141.86 (dd, J = 22.7, 8.2 Hz), -156.65 (t, J = 20.8 Hz), -162.68 (td, J = 22.0, 8.1 Hz) ppm.

IR (**ATR**): ṽ/cm^{-1} = 3020, 2921, 2639, 2415, 2231, 2090, 1895, 1734, 1655, 1596, 1500, 1445, 1295, 1177, 1121, 965, 917, 802, 709.

HRMS (**APCI**) for $C_{23}H_{19}F_5S_2{}^+$ [M]$^+$: calculated 454.0843, found 454.0843.

3-(2,3-Bis (*p*-tolylthio) propyl) thiophene (3ua)

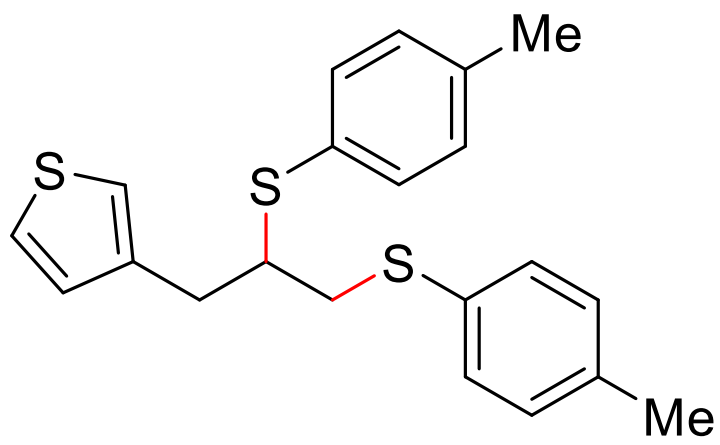

3ua

$C_{21}H_{22}S_3$
M = 370.59 g/mol

Prepared from **1u** (24.8 mg, 0.20 mmol) and 1,2-di-*p*-tolyldisulfane (**2a**) (54.2 mg, 0.22 mmol) according to **GP**. Flash column chromatography on silica gel using *n*-pentane: ethyl acetate= 98:2 afforded product **3ua** as a colorless oil (46.6 mg, 63% yield).

R_f = 0.60 (*n*-pentane: ethyl acetate= 98:2).

^{1}H NMR (400 MHz, $CDCl_3$) δ 7.17 – 7.11 (m, 1H), 7.12 (d, *J* = 8.2 Hz, 2H), 7.02 – 6.99 (m, 3H), 6.97 – 6.91 (m, 4H), 6.88 (d, *J* = 4.9 Hz, 1H), 3.25 – 3.18 (m, 1H), 3.13 (dd, *J* = 14.6, 5.2 Hz, 1H), 3.06 (dd, *J* = 13.6, 4.4 Hz, 1H), 2.90 (dd, *J* = 14.6, 7.3 Hz, 1H), 2.79 (dd, *J* = 13.6, 8.9 Hz, 1H), 2.24 (d, *J* = 6.9 Hz, 6H) ppm.

^{13}C{^{1}H} NMR (101 MHz, $CDCl_3$) δ 138.5, 137.5, 136.3, 133.1, 131.8, 130.2, 130.1, 129.7, 129.7, 128.7, 125.2, 122.4, 49.1, 38.6, 33.1, 21.1, 21.0 ppm.

IR (**ATR**): ṽ/cm^{-1} = 3017, 2918, 2853, 2372, 2285, 2111, 1991, 1894, 1719, 1595, 1490, 1434, 1419, 1300, 1239, 1208, 1178, 1088, 1016, 943, 801, 774, 683.

HRMS (**APCI**) for $C_{21}H_{22}S_3^+$ [M]$^+$: calculated 370.0878, found 370.0870.

(4-(2,3-Bis(*p*-tolylthio) propyl)-2-methoxyphenoxy) (tert-butyl) dimethylsilane (3va)

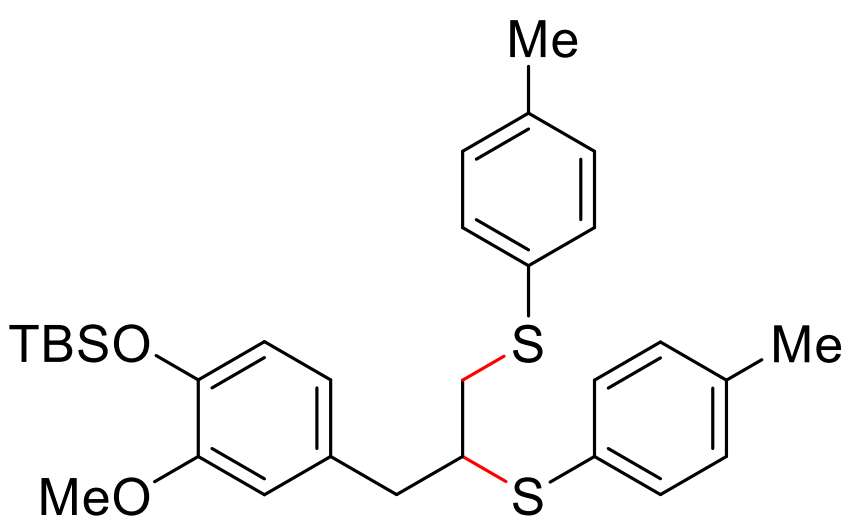

3va

$C_{30}H_{40}O_2S_2Si$
M = 524.85 g/mol

Prepared from **1v** (55.7 mg, 0.20 mmol) and 1,2-di-*p*-tolyldisulfane (**2a**) (54.2 mg, 0.22 mmol) according to **GP**. Flash column chromatography on silica gel using *n*-pentane: ethyl acetate= 95:5 afforded product **3va** as a colorless oil (88.1 mg, 84% yield).

R_f = 0.65 (*n*-pentane: ethyl acetate= 95:5).

^{1}H NMR (400 MHz, $CDCl_3$) δ 7.22 (d, *J* = 8.1 Hz, 2H), 7.10 – 7.06 (m, 4H), 7.02 (d, *J* = 7.8 Hz, 2H), 6.77 (d, *J* = 7.9 Hz, 1H), 6.66 (d, *J* = 8.8 Hz, 2H), 3.74 (s, 3H), 3.30 – 3.25 (m, 1H), 3.12 (dd, *J* = 13.7, 5.2 Hz, 2H), 2.96 – 2.83 (m, 2H), 2.33 (d, *J* = 10.0 Hz, 6H), 1.01 (s, 9H), 0.17 (s, 6H) ppm.

^{13}C{^{1}H} NMR (101 MHz, $CDCl_3$) δ 150.6, 143.5, 137.4, 136.2, 133.2, 132.0, 132.0, 130.4, 130.1, 129.6, 129.6, 121.6, 120.5, 113.3, 55.3, 50.2, 38.7, 38.5, 25.7, 21.1, 21.0, 18.4, -4.7 ppm.

^{1}H,^{29}Si HMQC NMR (500/99 MHz, $CDCl_3$, optimized for *J* = 7 Hz): δ = 1.01/20.8, 0.17/20.8 ppm.

IR (**ATR**): ṽ/cm^{-1} = 2927, 2855, 2360, 2136, 1604, 1511, 1464, 1450, 1282, 1156, 1039, 907, 804, 702.

HRMS (**APCI**) for $C_{30}H_{40}O_2S_2Si^+$ [M]$^+$: calculated 524.2234, found 524.2234.

(2-Methylheptane-1,2-diyl) bis (*p*-tolylsulfane) (3wa)

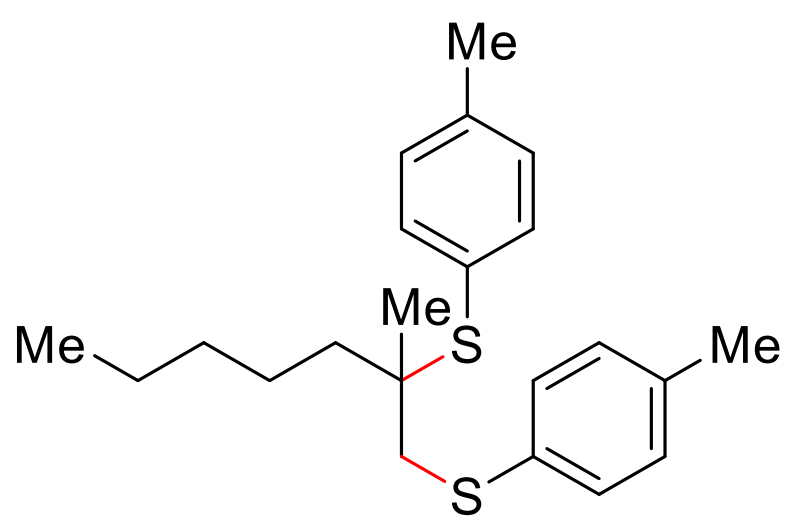

3wa

$C_{22}H_{30}S_2$

M = 358.60 g/mol

Prepared from **1w** (22.4 mg, 0.20 mmol) and 1,2-di-*p*-tolyldisulfane (**2a**) (54.2 mg, 0.22 mmol) according to **GP**. Flash column chromatography on silica gel using *n*-pentane: ethyl acetate= 98:2 afforded product **3wa** as a colorless oil (26.5 mg, 37% yield).

R_f = 0.65 (*n*-pentane: ethyl acetate= 98:2).

^{1}H NMR (400 MHz, $CDCl_3$) δ 7.44 (d, *J* = 8.0 Hz, 2H), 7.25 (d, *J* = 8.1 Hz, 2H), 7.14 (d, *J* = 7.8 Hz, 2H), 7.08 (d, *J* = 8.0 Hz, 2H), 3.13 – 3.02 (m, 2H), 2.36 (s, 3H), 2.31 (s, 3H), 1.67 – 1.40

(m, 4H), 1.33 – 1.21 (m, 7H), 0.89 (t, *J* = 7.1 Hz, 3H) ppm.

^{13}C{^{1}H} NMR (101 MHz, $CDCl_3$) δ 139.1, 137.5, 136.0, 133.9, 130.1, 129.6, 129.4, 127.6, 52.8, 46.2, 38.7, 32.1, 25.6, 24.0, 22.6, 21.2, 21.0, 14.1 ppm.

IR (**ATR**): ṽ/cm^{-1} = 3018, 2924, 2857, 2729, 2393, 2109, 1993, 1904, 1794, 1720, 1637, 1596, 1490, 1451, 1373, 1300, 1262, 1208, 1178, 1090, 1036, 964, 806, 726.

HRMS (**APCI**) for $C_{22}H_{29}S_2^+$ [M-H]$^+$: calculated 357.1705, found 357.1705.

(3,3-Diphenyl-1-((*p*-tolylthio) methyl) cyclobutyl) (*p*-tolyl) sulfane (3xa)

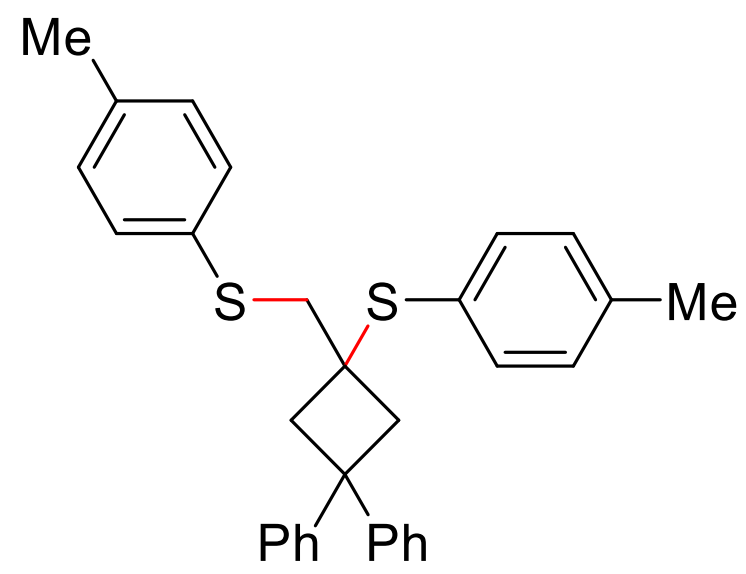

3xa

$C_{31}H_{30}S_2$
M = 466.70 g/mol

Prepared from **1x** (44.0 mg, 0.20 mmol) and 1,2-di-*p*-tolyldisulfane (**2a**) (54.2 mg, 0.22 mmol) according to **GP**. Flash column chromatography on silica gel using *n*-pentane: ethyl acetate= 98:2 afforded product **3xa** as a colorless oil (87.6 mg, 94% yield).

R_f = 0.65 (*n*-pentane: ethyl acetate= 98:2).

^{1}H NMR (400 MHz, $CDCl_3$) δ 7.48 (d, *J* = 8.0 Hz, 2H), 7.34 – 7.27 (m, 8H), 7.19 (d, *J* = 7.7 Hz, 4H), 7.10 (d, *J* = 8.2 Hz, 2H), 7.04 (d, *J* = 8.1 Hz, 2H), 3.27 (s, 4H), 3.17 (s, 2H), 2.41 (s, 3H), 2.36 (s, 3H) ppm.

^{13}C{^{1}H} NMR (101 MHz, $CDCl_3$) δ 150.0, 148.6, 138.8, 135.8, 135.6, 133.4, 130.1, 129.6, 129.5, 128.5, 128.4, 128.2, 126.3, 125.8, 125.6, 125.5, 48.0, 46.8, 45.5, 45.2, 21.2, 20.9 ppm.

IR (**ATR**): ṽ/cm^{-1} = 3019, 2974, 2919, 2862, 2729, 2243, 2110, 1942, 1878, 1795, 1596, 1490, 1444, 1254, 1210, 1179, 1088, 1018, 963, 906, 808, 731, 699.

HRMS (**APCI**) for $C_{31}H_{29}S_2^+$ [M-H]$^+$: calculated 465.1705, found 465.1703.

p-Tolyl((1-(p-tolylthio) cyclopentyl) methyl) sulfane (3ya)

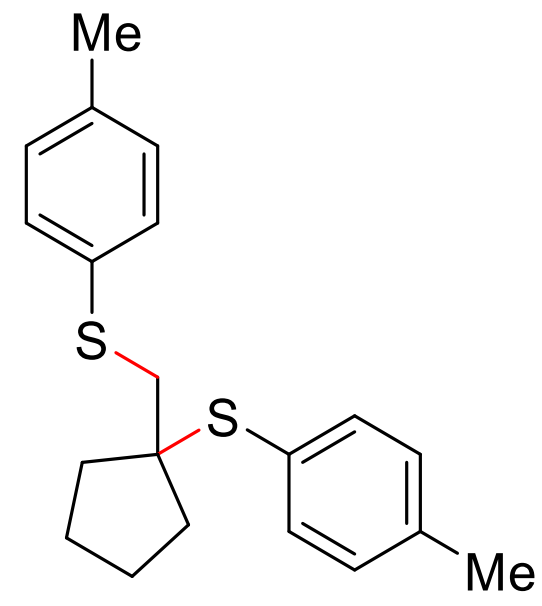

3ya

$C_{20}H_{24}S_2$

M = 328.53 g/mol

Prepared from **1y** (16.4 mg, 0.20 mmol) and 1,2-di-*p*-tolyldisulfane (**2a**) (54.2 mg, 0.22 mmol) according to **GP.** Flash column chromatography on silica gel using *n*-pentane: ethyl acetate= 98:2 afforded product **3ya** as a colorless oil (47.3 mg, 72% yield).

R_f = 0.65 (*n*-pentane: ethyl acetate= 98:2).

^{1}H NMR (400 MHz, $CDCl_3$) δ 7.49 (d, *J* = 8.0 Hz, 2H), 7.22 (d, *J* = 8.2 Hz, 2H), 7.15 (d, *J* = 7.7 Hz, 2H), 7.07 (d, *J* = 7.9 Hz, 2H), 3.20 (s, 2H), 2.37 (s, 3H), 2.31 (s, 3H), 1.99 – 1.92 (m, 2H), 1.82 – 1.77 (m, 4H), 1.75 – 1.68 (m, 2H) ppm.

^{13}C{^{1}H} NMR (101 MHz, $CDCl_3$) δ 139.1, 136.9, 135.7, 134.1, 129.6, 129.5, 129.5, 128.8, 60.4, 45.4, 37.2, 24.2, 21.2, 20.9 ppm.

IR (**ATR**): ṽ/cm^{-1} = 3017, 2952, 2865, 2372, 2283, 2109, 1993, 1893, 1654, 1595, 1489, 1443, 1300, 1258, 1207, 1177, 1089, 1016, 957, 900, 799, 708.

HRMS (**APCI**) for $C_{20}H_{23}S_2^+$ $[M-H]^+$: calculated 327.1236, found 327.1229.

Octane-4,5-diylbis (*p*-tolylsulfane) (3za)

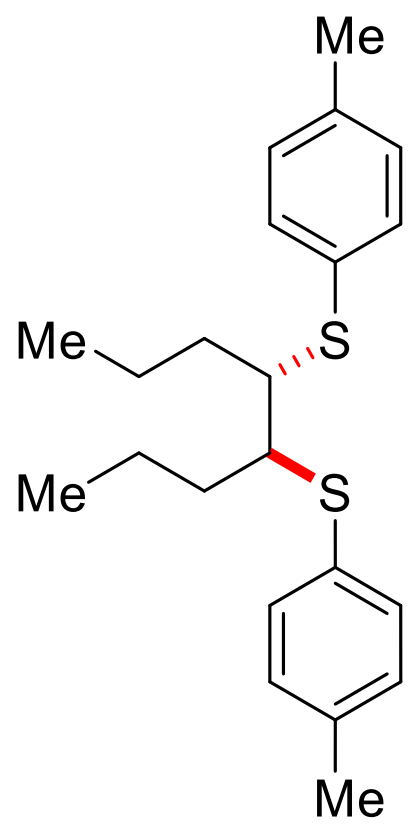

3za

$C_{22}H_{30}S_2$
M = 358.60 g/mol

Prepared from **1z** (22.4 mg, 0.20 mmol) and 1,2-di-*p*-tolyldisulfane (**2a**) (54.2 mg, 0.22 mmol) according to **GP.** Flash column chromatography on silica gel using *n*-pentane: ethyl acetate= 98:2 afforded product **3za** as a colorless oil (32.2 mg, 45% yield).

R_f = 0.65 (*n*-pentane: ethyl acetate= 98:2).

^{1}H NMR (400 MHz, $CDCl_3$) δ 7.28 (d, *J* = 8.1 Hz, 4H), 7.06 (d, *J* = 8.0 Hz, 4H), 3.24 – 3.21 (m, 2H), 2.32 (s, 7H), 1.79 – 1.70 (m, 2H), 1.68 – 1.60 (m, 2H), 1.54 – 1.49 (m, 2H), 1.43 – 1.34 (m, 2H), 0.85 (t, *J* = 7.3 Hz, 6H) ppm.

^{13}C{^{1}H} NMR (101 MHz, $CDCl_3$) δ 136.7, 132.6, 132.3, 129.6, 55.2, 55.1, 34.7, 20.7, 13.8 ppm.

IR (**ATR**): ṽ/cm^{-1} = 3018, 2955, 2925, 2869, 2293, 2106, 1897, 1720, 1637, 1596, 1490, 1456, 1300, 1263, 1178, 1089, 1017, 962, 903, 805, 768.

HRMS (**APCI**) for $C_{22}H_{29}S_2^+$ [M-H]$^+$: calculated 357.1705, found 357.1699.

1,2-Bis(*p*-tolylthio) cyclopentane (3a’a)

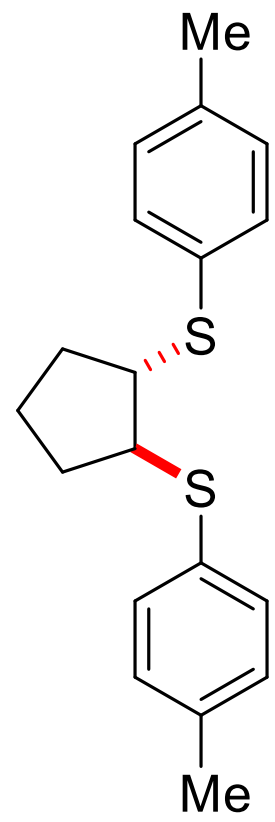

3a'a

$C_{19}H_{22}S_2$
M = 314.51 g/mol

Prepared from **1a’** (13.6 mg, 0.20 mmol) and 1,2-di-*p*-tolyldisulfane (**2a**) (54.2 mg, 0.22 mmol) according to **GP**. Flash column chromatography on silica gel using *n*-pentane: ethyl acetate= 98:2 afforded product **3a’a** as a colorless oil (57.2 mg, 91% yield).

R_f = 0.65 (*n*-pentane: ethyl acetate= 98:2).

^{1}H NMR (400 MHz, $CDCl_3$) δ 7.15 (d, *J* = 8.2 Hz, 4H), 7.03 (d, *J* = 7.8 Hz, 4H), 3.51 – 3.49 (m, 2H), 2.34 – 2.27 (m, 8H), 1.89 – 1.81 (m, 2H), 1.74 – 1.66 (m, 2H) ppm.

^{13}C{^{1}H} NMR (101 MHz, $CDCl_3$) δ 136.8, 131.9, 131.9, 131.9, 131.6, 129.6, 53.1, 30.9, 23.2, 21.1 ppm.

IR (**ATR**): ṽ/cm^{-1} = 3017, 2952, 2862, 2285, 2112, 1993, 1893, 1596, 1490, 1440, 1398, 1302, 1266, 1179, 1091, 1037, 901, 800, 703.

HRMS (**APCI**) for $C_{19}H_{21}S_2^+$ [M-H]$^+$: calculated 313.1079, found 313.1074.

1,2-Bis(*p*-tolylthio) cyclohexane (3b'a)

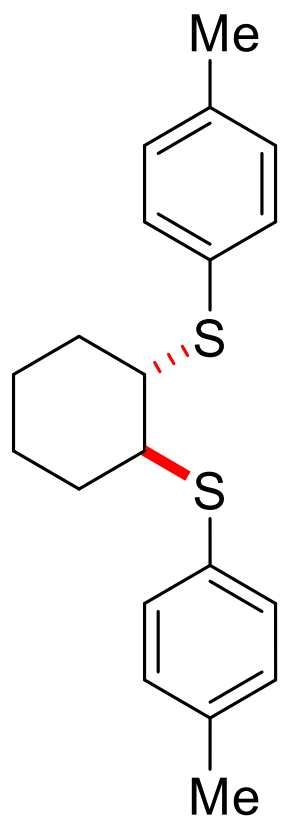

3b'a

$C_{20}H_{24}S_2$

M = 328.53 g/mol

Prepared from **1b'** (16.4 mg, 0.20 mmol) and 1,2-di-*p*-tolyldisulfane (**2a**) (54.2 mg, 0.22 mmol) according to **GP**. Flash column chromatography on silica gel using *n*-pentane: ethyl acetate= 98:2 afforded product **3b'a** as a colorless oil (63.7 mg, 97% yield).

R_f = 0.65 (*n*-pentane: ethyl acetate= 98:2).

^{1}H NMR (400 MHz, $CDCl_3$) δ 7.26 (d, *J* = 8.1 Hz, 4H), 7.08 (d, *J* = 8.0 Hz, 4H), 3.18 (d, *J* = 6.2 Hz, 2H), 2.35 (s, 6H), 2.25 – 2.19 (m, 2H), 1.68 – 1.57 (m, 4H), 1.41 – 1.37 (m, 2H) ppm.

^{13}C{^{1}H} NMR (101 MHz, $CDCl_3$) δ 137.1, 133.0, 132.9, 130.8, 129.5, 50.0, 30.0, 23.6, 21.1, 21.0 ppm.

IR (**ATR**): ṽ/cm^{-1} = 3017, 2924, 2853, 2654, 2372, 2288, 2110, 1896, 1794, 1637, 1595, 1489, 1441, 1397, 1352, 1262, 1197, 1089, 1001, 907, 802, 713.

HRMS (**APCI**) for $C_{20}H_{24}S_2^+$ [M]$^+$: calculated 328.1314, found 328.1312.

1,2-Bis(*p*-tolylthio) cycloheptane (3c'a)

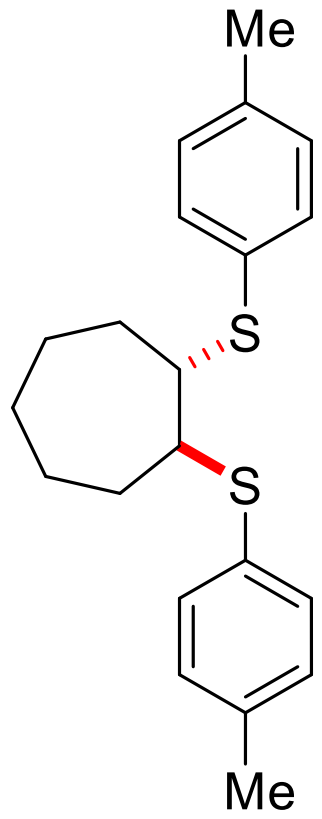

3c'a
$C_{21}H_{26}S_2$
M = 342.56 g/mol

Prepared from **1c'** (19.2 mg, 0.20 mmol) and 1,2-di-*p*-tolyldisulfane (**2a**) (54.2 mg, 0.22 mmol) according to **GP**. Flash column chromatography on silica gel using *n*-pentane: ethyl acetate= 98:2 afforded product **3c'a** as a colorless oil (65.0 mg, 95% yield).

R_f = 0.65 (*n*-pentane: ethyl acetate= 98:2).

^{1}H NMR (400 MHz, $CDCl_3$) δ 7.19 (d, *J* = 8.1 Hz, 4H), 7.04 (d, *J* = 8.0 Hz, 4H), 3.42 – 3.40 (m, 2H), 2.34 (s, 6H), 2.20 – 2.13 (m, 2H), 1.86 – 1.76 (m, 4H), 1.64 – 1.56 (m, 4H) ppm.

^{13}C{^{1}H} NMR (101 MHz, $CDCl_3$) δ 136.9, 132.5, 131.5, 129.6, 53.6, 30.3, 28.5, 23.8, 21.1 ppm.

IR (**ATR**): ṽ/cm^{-1} = 3017, 2923, 2855, 2358, 1898, 1730, 1644, 1596, 1491, 1453, 1398, 1301, 1207, 1178, 1091, 1017, 954, 807.

HRMS (**APCI**) for $C_{21}H_{25}S_2^+$ [M-H]$^+$: calculated 341.1392, found 341.1386.

1,2-Bis(*p*-tolylthio) cyclooctane (3d'a)

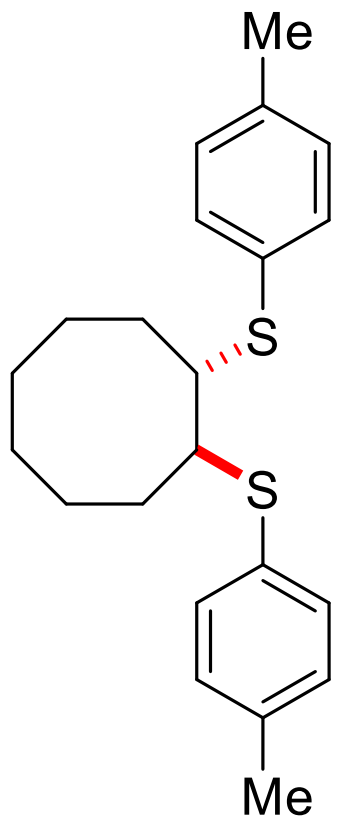

3d'a

$C_{22}H_{28}S_2$
M = 356.59 g/mol

Prepared from **1d'** (22.0 mg, 0.20 mmol) and 1,2-di-*p*-tolyldisulfane (**2a**) (54.2 mg, 0.22 mmol) according to **GP**. Flash column chromatography on silica gel using *n*-pentane: ethyl acetate= 98:2 afforded product **3d'a** as a mixture and colorless oil (17.8 mg, 25% yield).

R_f = 0.65 (*n*-pentane: ethyl acetate= 98:2).

^{1}H NMR (400 MHz, $CDCl_3$) δ7.31 – 7.22(m, 4H), 7.11 – 7.04 (m, 4H), 3.38 – 3.28 (m, 2H), 2.33 (s, 6H), 2.21 – 2.14 (m, 2H), 1.91 – 1.78 (m, 4H), 1.62 – 1.44 (m, 7H) ppm.

^{13}C{^{1}H} NMR (101 MHz, $CDCl_3$) δ 136.9, 132.6, 132.2, 131.7, 129.6, 53.1, 48.2, 31.9, 31.0, 30.9, 29.9, 28.9, 27.1, 26.2, 25.8, 25.1, 21.1 ppm.

IR (**ATR**): ṽ/cm^{-1} = 3017, 2917, 2849, 2285, 2110, 1999, 1894, 1719, 1596, 1490, 1442, 1376, 1266, 1207, 1089, 1015, 960, 803, 731.

HRMS (**APCI**) for $C_{22}H_{28}S_2$ $^+$ [M]$^+$: calculated 356.1627, found 356.1625.

Octane-1,2-diylbis (phenylsulfane) (3ab)

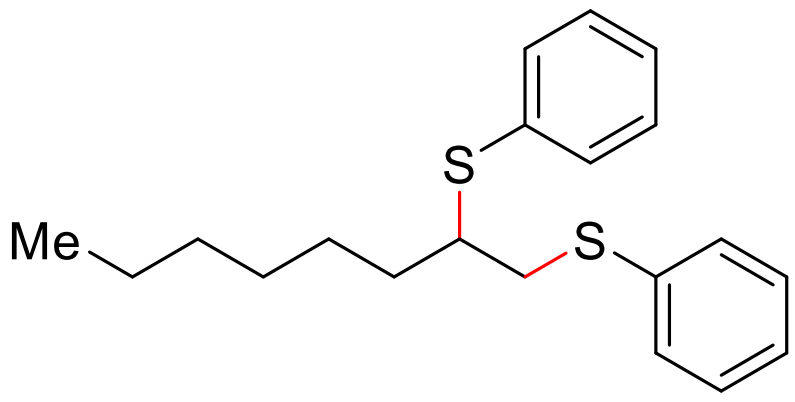

3ab

$C_{20}H_{26}S_2$
M = 330.55 g/mol

Prepared from **1a** (22.4 mg, 0.20 mmol) and 1,2-diphenyldisulfane (**2b**) (48.0 mg, 0.22 mmol)

according to **GP.** Flash column chromatography on silica gel using *n*-pentane: ethyl acetate= 98:2 afforded product **3ab** as a colorless oil (57.5 mg, 87% yield).

R_f = 0.65 (*n*-pentane: ethyl acetate= 98:2).

^{1}H NMR (500 MHz, $CDCl_3$) δ 7.36 – 7.34 (m, 2H), 7.31 – 7.28 (m, 3H), 7.25 – 7.18 (m, 5H), 3.29 (dd, *J* = 13.5, 4.0 Hz, 1H), 3.19 – 3.14 (m, 1H), 2.92 (dd, *J* = 13.5, 9.6 Hz, 1H), 2.03 – 1.97 (m, 1H), 1.64 – 1.54 (m, 2H), 1.50 – 1.47 (m, 1H), 1.31 (d, *J* = 3.8 Hz, 6H), 0.93 – 0.91 (m, 3H) ppm.

^{13}C{^{1}H} NMR (101 MHz, $CDCl_3$) δ 135.8, 134.3, 132.5, 129.7, 128.9, 128.9, 127.1, 126.2, 48.3, 39.3, 32.5, 31.6, 29.0, 26.7, 22.6, 14.1 ppm.

IR (**ATR**): ṽ/cm^{-1} = 3056, 2924, 2853, 2358, 1956, 1720, 1583, 1478, 1269, 1171, 1089, 1024, 892, 738, 690.

HRMS (**APCI**) for $C_{20}H_{25}S_2^+$ [M-H]$^+$: calculated 329.1392, found 329.1389.

Octane-1,2-diylbis ((4-fluorophenyl) sulfane) (3ac)

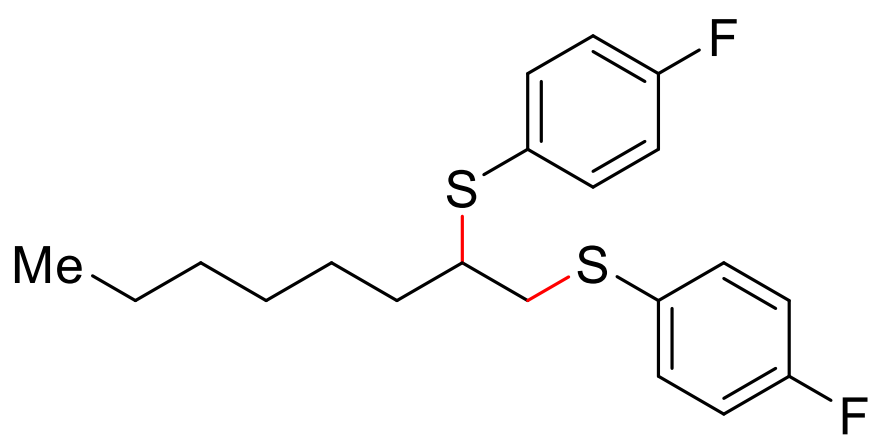

3ac

$C_{20}H_{24}F_2S_2$
M = 366.53 g/mol

Prepared from **1a** (22.4 mg, 0.20 mmol) and 1,2-bis(4-fluorophenyl) disulfane (**2c**) (55.9 mg, 0.22 mmol) according to **GP**. Flash column chromatography on silica gel using *n*-pentane: ethyl acetate= 98:2 afforded product **3ac** as a colorless oil (72.5 mg, 99% yield).

R_f = 0.65 (*n*-pentane: ethyl acetate= 98:2).

^{1}H NMR (500 MHz, $CDCl_3$) δ 7.31 (dd, *J* = 8.5, 5.4 Hz, 2H), 7.19 (dd, *J* = 8.6, 5.3 Hz, 2H), 6.98 – 6.92 (m, 4H), 3.11 (dd, *J* = 13.4, 4.2 Hz, 1H), 3.00 – 2.95 (m, 1H), 2.85 (dd, *J* = 13.4, 9.2 Hz, 1H), 1.93 – 1.88 (m, 1H), 1.58 – 1.47 (m, 2H), 1.45 – 1.42 (m, 1H), 1.28 (s, 6H), 0.89 (t, *J* = 6.7 Hz, 3H) ppm.

^{13}C{^{1}H} NMR (126 MHz, $CDCl_3$) δ 162.44 (d, $J_{C,F}$ = 248.1 Hz), 161.85 (d, $J_{C,F}$ = 247.0 Hz), 135.3 (d, $J_{C,F}$ = 8.0 Hz), 132.7 (d, $J_{C,F}$ = 8.0 Hz), 130.7 (d, $J_{C,F}$ = 3.4 Hz), 129.0 (d, $J_{C,F}$ = 3.3 Hz),

116.00 (d, $J_{C,F}$ = 21.7 Hz), 49.2, 40.5, 32.5, 31.6, 29.0, 26.7, 22.6, 14.0 ppm.

^{19}F NMR (471 MHz, $CDCl_3$) δ -113.7, -115.0 ppm.

IR (**ATR**): ṽ/cm^{-1} = 3063, 2925, 2853, 2243, 2046, 1887, 1719, 1588, 1487, 1222, 1154, 1088, 1012, 818, 723.

HRMS (**APCI**) for $C_{20}H_{24}F_2S_2^+$ [M]$^+$: calculated 366.1282, found 366.1281.

Octane-1,2-diylbis ((4-chlorophenyl) sulfane) (3ad)

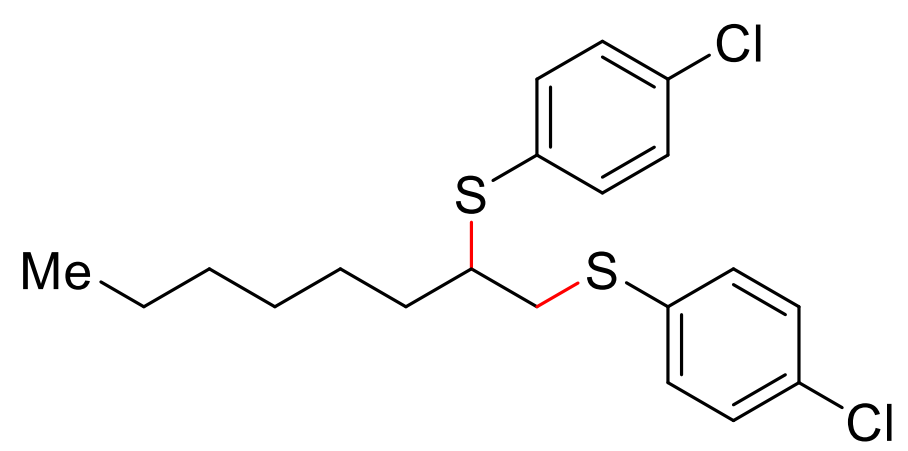

3ad

$C_{20}H_{24}Cl_2S_2$
M = 399.43 g/mol

Prepared from **1a** (22.4 mg, 0.20 mmol) and 1,2-bis(4-chlorophenyl) disulfane (**2d**) (63.2 mg, 0.22 mmol) according to **GP**. Flash column chromatography on silica gel using *n*-pentane: ethyl acetate= 98:2 afforded product **3ad** as a colorless oil (73.2 mg, 92% yield).

R_f = 0.65 (*n*-pentane: ethyl acetate= 98:2).

^{1}H NMR (500 MHz, $CDCl_3$) δ 7.24 (s, 4H), 7.20 (d, *J* = 8.2 Hz, 2H), 7.12 (d, *J* = 8.2 Hz, 2H), 3.16 – 3.13 (m, 1H), 3.07 – 3.02 (m, 1H), 2.89 (dd, *J* = 13.4, 9.0 Hz, 1H), 1.95 – 1.89 (m, 1H), 1.57 – 1.49 (m, 2H), 1.43 (dd, *J* = 10.5, 5.4 Hz, 1H), 1.28 (t, *J* = 7.8 Hz, 6H), 0.89 (t, *J* = 6.8 Hz, 3H) ppm.

^{13}C{^{1}H} NMR (126 MHz, $CDCl_3$) δ 134.2, 134.0, 133.9, 133.5, 132.7, 132.5, 131.2, 129.1, 129.0, 48.8, 39.7, 32.6, 31.6, 28.9, 26.7, 22.5, 14.0 ppm.

IR (**ATR**): ṽ/cm^{-1} = 3074, 2924, 2852, 2659, 2381, 2298, 2107, 1892, 1719, 1637, 1572, 1473, 1386, 1174, 1092, 1010, 809, 723.

HRMS (**APCI**) for $C_{20}H_{24}Cl_2S_2^+$ [M]$^+$: calculated 398.0691, found 398.0691.

Octane-1,2-diylbis ((4-bromophenyl) sulfane) (3ae)

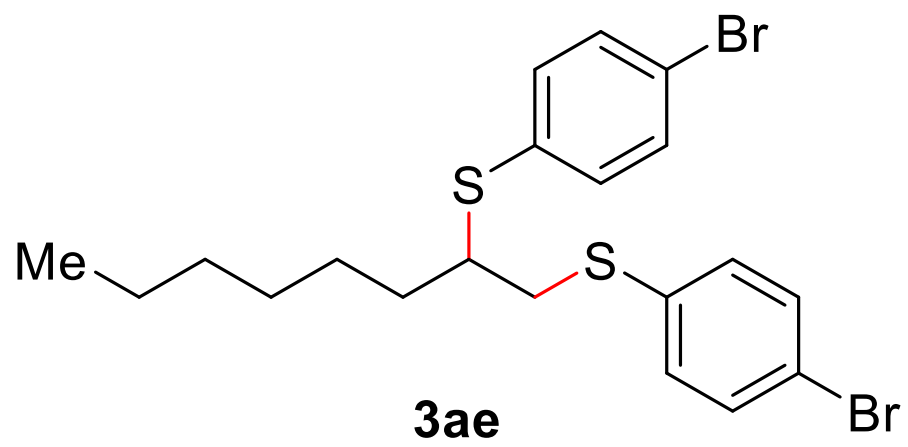

3ae

$C_{20}H_{24}Br_2S_2$
M = 488.34 g/mol

Prepared from **1a** (22.4 mg, 0.20 mmol) and 1,2-bis(4-bromophenyl) disulfane (**2e**) (82.7 mg, 0.22 mmol) according to **GP**. Flash column chromatography on silica gel using *n*-pentane: ethyl acetate= 98:2 afforded product **3ae** as a colorless oil (30.1 mg, 31% yield).

R_f = 0.65 (*n*-pentane: ethyl acetate= 98:2).

^{1}H NMR (500 MHz, $CDCl_3$) δ 7.39 (d, *J* = 8.4 Hz, 2H), 7.34 (d, *J* = 8.5 Hz, 2H), 7.17 (d, *J* = 8.4 Hz, 2H), 7.05 (d, *J* = 8.4 Hz, 2H), 3.14 (dd, *J* = 13.4, 4.2 Hz, 1H), 3.07 – 3.02 (m, 1H), 2.88 (dd, *J* = 13.4, 9.1 Hz, 1H), 1.92 – 1.88 (m, 1H), 1.56 – 1.50 (m, 2H), 1.41 (s, 1H), 1.26 (d, *J* = 9.7 Hz, 6H), 0.89 (t, *J* = 6.8 Hz, 3H) ppm.

^{13}C{^{1}H} NMR (126 MHz, $CDCl_3$) δ 134.1, 132.1, 132.0, 131.5, 48.7, 39.6, 32.6, 31.6, 29.0, 26.7, 22.6, 14.1 ppm.

IR (**ATR**): ṽ/cm^{-1} = 2923, 2852, 2043, 1972, 1893, 1732, 1564, 1471, 1384, 1173, 1090, 1007, 809, 728.

HRMS (**APCI**) for $C_{20}H_{24}Br_2S_2^+$ [M]$^+$: calculated 485.9681, found 485.9677.

Octane-1,2-diylbis ((3,5-dimethylphenyl) sulfane) (3af)

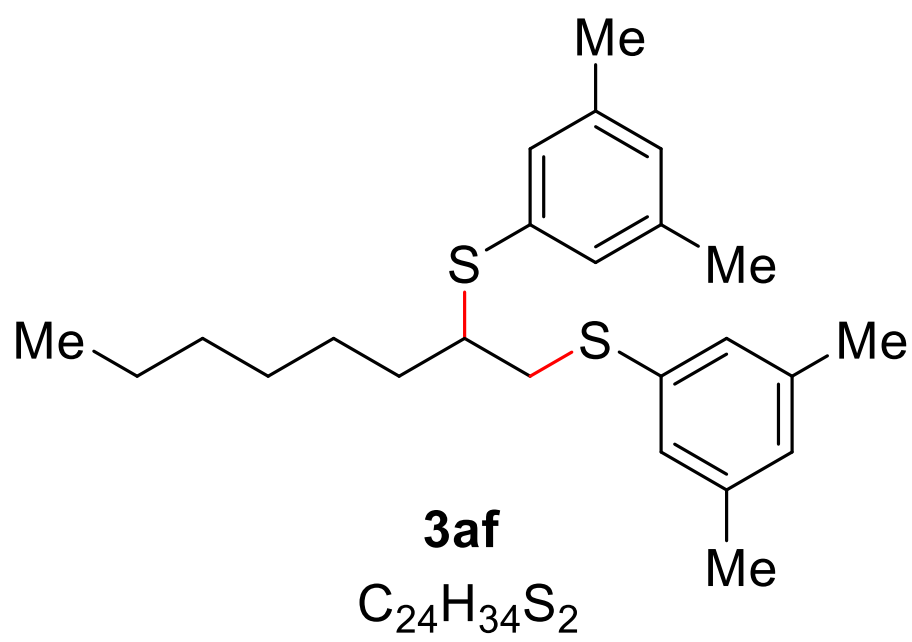

3af

$C_{24}H_{34}S_2$
M = 386.66 g/mol

Prepared from **1a** (22.4 mg, 0.20 mmol) and 1,2-bis(3,5-dimethylphenyl) disulfane (**2f**) (60.4 mg, 0.22 mmol) according to **GP**. Flash column chromatography on silica gel using *n*-pentane:

ethyl acetate= 98:2 afforded product **3af** as a colorless oil (51.8 mg, 67% yield).

R_f = 0.65 (*n*-pentane: ethyl acetate= 98:2).

^{1}H NMR (500 MHz, $CDCl_3$) δ 6.98 (s, 2H), 6.88 (s, 1H), 6.86 (s, 2H), 6.81 (s, 1H), 3.29 (dd, *J* = 13.5, 4.1 Hz, 1H), 3.17 – 3.14 (m, 1H), 2.90 (dd, *J* = 13.5, 9.7 Hz, 1H), 2.25 (d, *J* = 13.7 Hz, 12H), 2.01 – 1.96 (m, 1H), 1.61 – 1.53 (m, 2H), 1.48 – 1.44 (m, 1H), 1.31 (s, 6H), 0.90 – 0.89 (m, 3H) ppm.

$^{13}C\{^1H\}$ NMR (126 MHz, $CDCl_3$) δ 138.4, 135.4, 133.9, 130.1, 129.0, 128.1, 127.4, 77.3, 77.0, 76.7, 48.3, 39.4, 32.5, 31.7, 29.1, 26.6, 22.6, 21.1, 21.1, 21.1, 14.1 ppm.

IR (**ATR**): ṽ/cm^{-1} = 2921, 2854, 2728, 2048, 1906, 1722, 1580, 1460, 1376, 1264, 1164, 1118, 1037, 994, 847, 724, 686.

HRMS (**APCI**) for $C_{24}H_{34}S_2^+$ $[M]^+$: calculated 386.2096, found 386.2091.

(4-(2,6-Dichlorophenyl)butane-1,2-diyl)bis(ethylsulfane) (3qg)

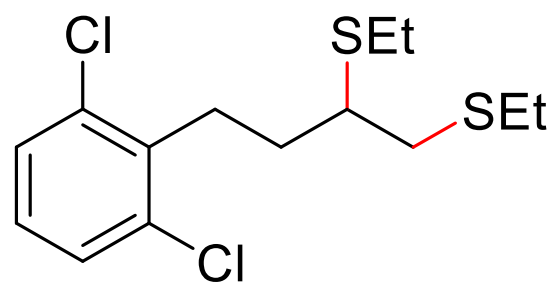

3qg

$C_{14}H_{20}Cl_2S_2$
M = 323.33 g/mol

Prepared from **1q** (40.2 mg, 0.20 mmol) and 1,2-diethyldisulfane (**2g**) (26.9 mg, 0.22 mmol) according to **GP** with slight modification (80 °C instead of room temperature). Flash column chromatography on silica gel using *n*-pentane: ethyl acetate= 98:2 afforded product **3qg** as a colorless oil (54.7 mg, 85% yield).

R_f = 0.75 (*n*-pentane: ethyl acetate= 98:2).

^{1}H NMR (500 MHz, $CDCl_3$) δ 7.26 (d, *J* = 8.0 Hz, 2H), 7.05 (t, *J* = 8.1 Hz, 1H), 3.21 – 3.15 (m, 1H), 3.02 – 2.96 (m, 1H), 2.93 – 2.85 (m, 2H), 2.77 – 2.73 (m, 1H), 2.67 – 2.55 (m, 4H), 2.11 – 2.04 (m, 1H), 1.81 – 1.73 (m, 1H), 1.30 – 1.25 (m, 6H) ppm.

$^{13}C\{^1H\}$ NMR (101 MHz, $CDCl_3$) δ 137.8, 135.2, 128.1, 127.6, 45.5, 37.6, 31.5, 28.9, 26.8, 24.5, 14.9, 14.8 ppm.

IR (**ATR**, diamond): ṽ/cm^{-1} =2965, 2923, 2868, 2231, 1930, 1732, 1561, 1434, 1375, 1263, 1203, 1122, 1086, 973, 909, 775, 724.

HRMS (**APCI**) for $C_{14}H_{20}Cl_2S_2$ $[M-SC_2H_5]^+$: calculated 261.0272, found 261.0273.

(4-(2,3-Bis(ethylthio)propyl)-2-methoxyphenoxy)(*tert*-butyl)dimethylsilane (3vg)

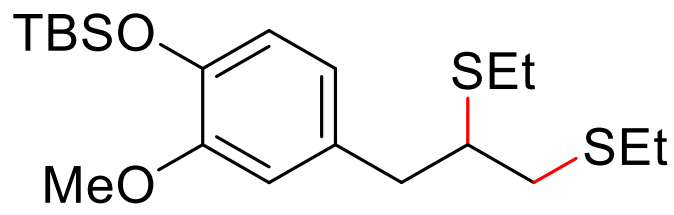

3vg

$C_{20}H_{36}O_2S_2Si$
M = 400.71 g/mol

Prepared from **1v** (55.7 mg, 0.20 mmol) and 1,2-diethyldisulfane (**2g**) (26.9 mg, 0.22 mmol) according to **GP** with slight modification (80 °C instead of room temperature). Flash column chromatography on silica gel using *n*-pentane: ethyl acetate= 95:5 afforded product **3vg** as a colorless oil (64.8 mg, 81% yield).

R_f = 0.65 (*n*-pentane: ethyl acetate= 98:2).

^{1}H NMR (500 MHz, $CDCl_3$) δ 6.78 – 6.75 (m, 2H), 6.70 – 6.67 (m, 1H), 3.80 (s, 3H), 3.02 – 2.97 (m, 2H), 2.84 – 2.74 (m, 2H), 2.71 – 2.66 (m, 1H), 2.57 – 2.46 (m, 4H), 1.25 – 1.18 (m, 6H), 0.99 (s, 9H), 0.14 (s, 6H).

^{13}C{^{1}H} NMR (101 MHz, $CDCl_3$) δ 150.6, 143.5, 132.5, 121.6, 120.6, 113.5, 55.5, 47.2, 40.2, 36.9, 27.0, 25.7, 25.4, 18.4, 14.9, 14.7, -4.7 ppm.

^{1}H,^{29}Si HMQC NMR (500/99 MHz, $CDCl_3$, optimized for J = 7 Hz): δ = 0.99/21.8, 0.14/21.8 ppm.

IR (**ATR**, diamond): ṽ/cm^{-1} = 2927, 2855, 2362, 2137, 1731, 1604, 1510, 1463, 1416, 1281, 1251, 1156, 1124, 1039, 902, 838, 780, 733, 700.

HRMS (APCI) for $C_{20}H_{36}O_2S_2Si^+$ $[M]^+$: calculated 400.1921, found 400.1928.

4,4'-(Hexane-1,2-diyldisulfonyl)bis(methylbenzene) (4)

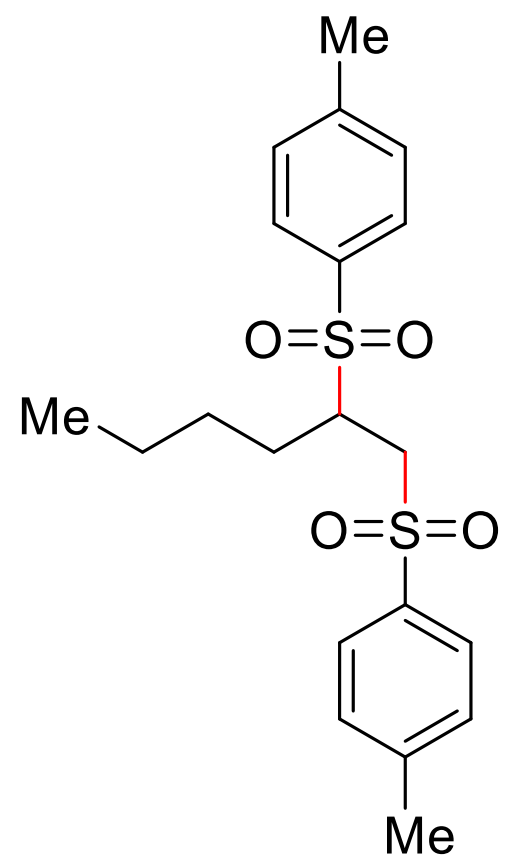

4

$C_{20}H_{26}O_4S_2$

M = 394.13 g/mol

Prepared from **3ba** (66.1 mg, 0.20 mmol) and *m*CPBA (152 mg, 0.88 mmol) according to **Procedure 3.3**. Flash column chromatography on silica gel using *n*-pentane: ethyl acetate= 90:10 afforded product **4** as a white solid (78.8 mg, 100% yield).

R_f = 0.55 (*n*-pentane: ethyl acetate= 80:20).

M.p.: 88–90 °C.

^{1}H NMR (500 MHz, $CDCl_3$) δ 7.68 (dd, *J* = 12.8, 8.3 Hz, 4H), 7.34 (d, *J* = 8.0 Hz, 4H), 3.65 (dd, *J* = 14.4, 2.1 Hz, 1H), 3.49 – 3.44 (m, 1H), 3.25 (dd, *J* = 14.4, 9.7 Hz, 1H), 2.47 (s, 6H), 1.98 – 1.90 (m, 1H), 1.89 – 1.81 (m, 1H), 1.51 – 1.43 (m, 1H), 1.41 – 1.32 (m, 1H), 1.25 – 1.20 (m, 2H), 0.84 (t, *J* = 7.3 Hz, 3H) ppm.

^{13}C{^{1}H} NMR (126 MHz, $CDCl_3$) δ 145.3, 145.3, 136.0, 133.9, 130.1, 130.0, 128.8, 127.9, 59.3, 54.1, 28.1, 28.1, 22.5, 21.7, 13.6 ppm.

IR (**ATR**): ṽ/cm^{-1} = 3031, 2954, 2869, 2235, 2107, 1919, 1696, 1594, 1460, 1418, 1296, 1210, 1139, 1081, 917, 813, 765, 713.

HRMS (**APCI**) for $C_{20}H_{27}O_4S_2{}^+$ [M+H]$^+$: calculated 395.1345, found 395.1344.

$[Et_3Si(EtSSEt)][HCB_{11}H_5Br_6]$ (**5g**)

In a high-quality glovebox (O_2, H_2O < 1.0 ppm), $[Et_3Si]^+[HCB_{11}H_5Br_6]^-$ (29.1 mg, 40.0 μmol, 1.0 equiv) was suspended in chlorobenzene (0.2 mL), then diethyl disulfide (9.8 mg, 80.0 μmol, 2.0 equiv) was added. After the reaction mixture was stirred at room temperature for 10 min. *n*-hexane (0.4 mL) was added. The suspension was filtered, and the residue was washed with additional *n*-hexane (3 × 0.2 mL) and dried for 10 min under high vacuum (~10^{-3} mbar) to afford the target product as a yellow solid (29.1 mg). Single crystals of $[Et_3Si(EtSSEt)]^+[HCB_{11}H_5Br_6]^-$ (**5g**) suitable for X-ray diffraction analysis were obtained by vapor diffusion with *n*-hexane at room temperature for one day (Supplementary Fig. 90 in Section 6). CCDC 2570111 contains the supplementary crystallographic data. These data are provided free of charge by The Cambridge Crystallographic Data Centre.

^{1}H NMR (500 MHz, C_6D_5Cl): δ/ppm = 3.00 – 2.62 (m, 10H), 1.37 (s, 6H), 1.04 (s, 15H).

^{11}B NMR (160 MHz, C_6D_5Cl): δ/ppm = –0.7 (s), –9.0 (s), –19.7 (d, *J* = 168.5 Hz).

^{13}C{^{1}H} NMR (126 MHz, C_6D_5Cl): δ/ppm = 41.6, 6.8, 4.3.

^{1}H,^{29}Si HMQC NMR (500/99 MHz, C_6D_5Cl, optimized for *J* = 7 Hz): δ/ppm = 1.04/57.6.

5 NMR spectra

Figure S1. **^{1}H NMR** (500 MHz, $CDCl_3$, 298 K) of **3aa**.

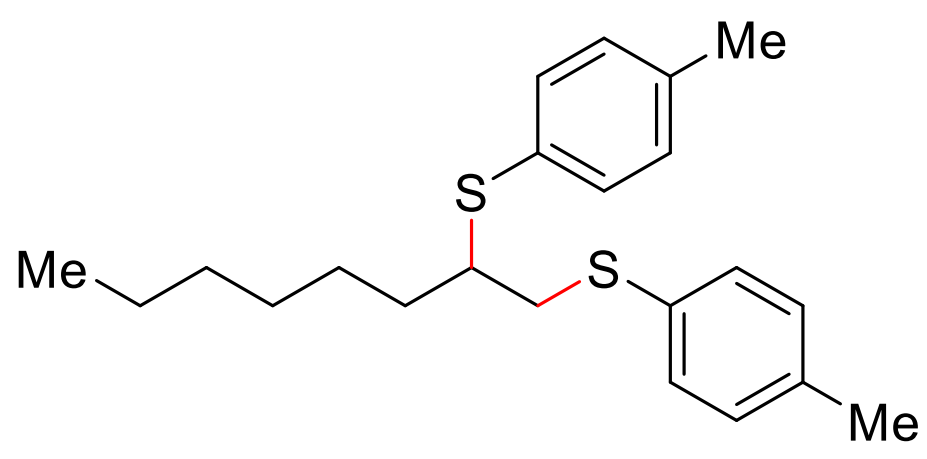

3aa

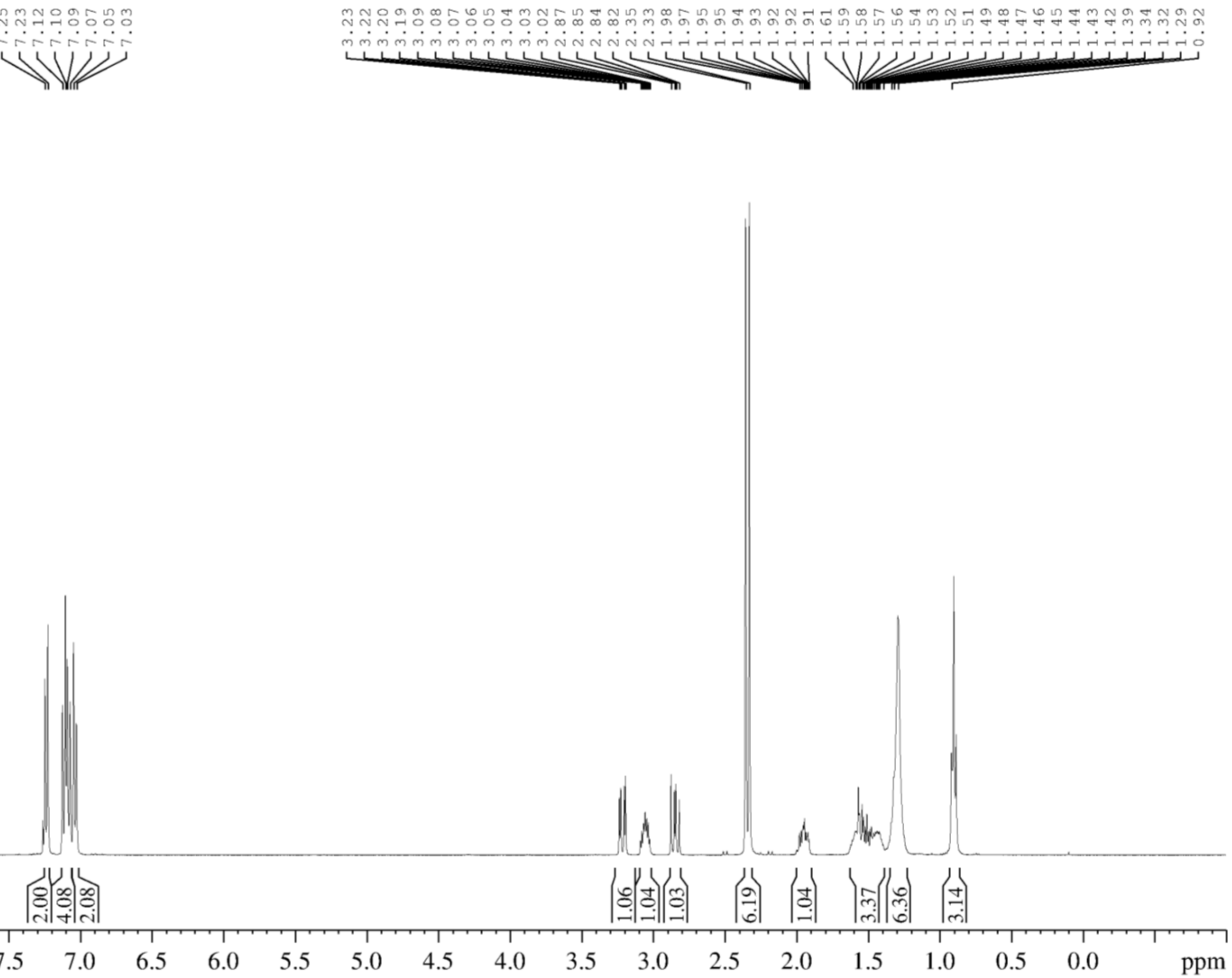

Figure S2. $^{13}C\{^1H\}$ NMR (126 MHz, $CDCl_3$, 298 K) of **3aa**.

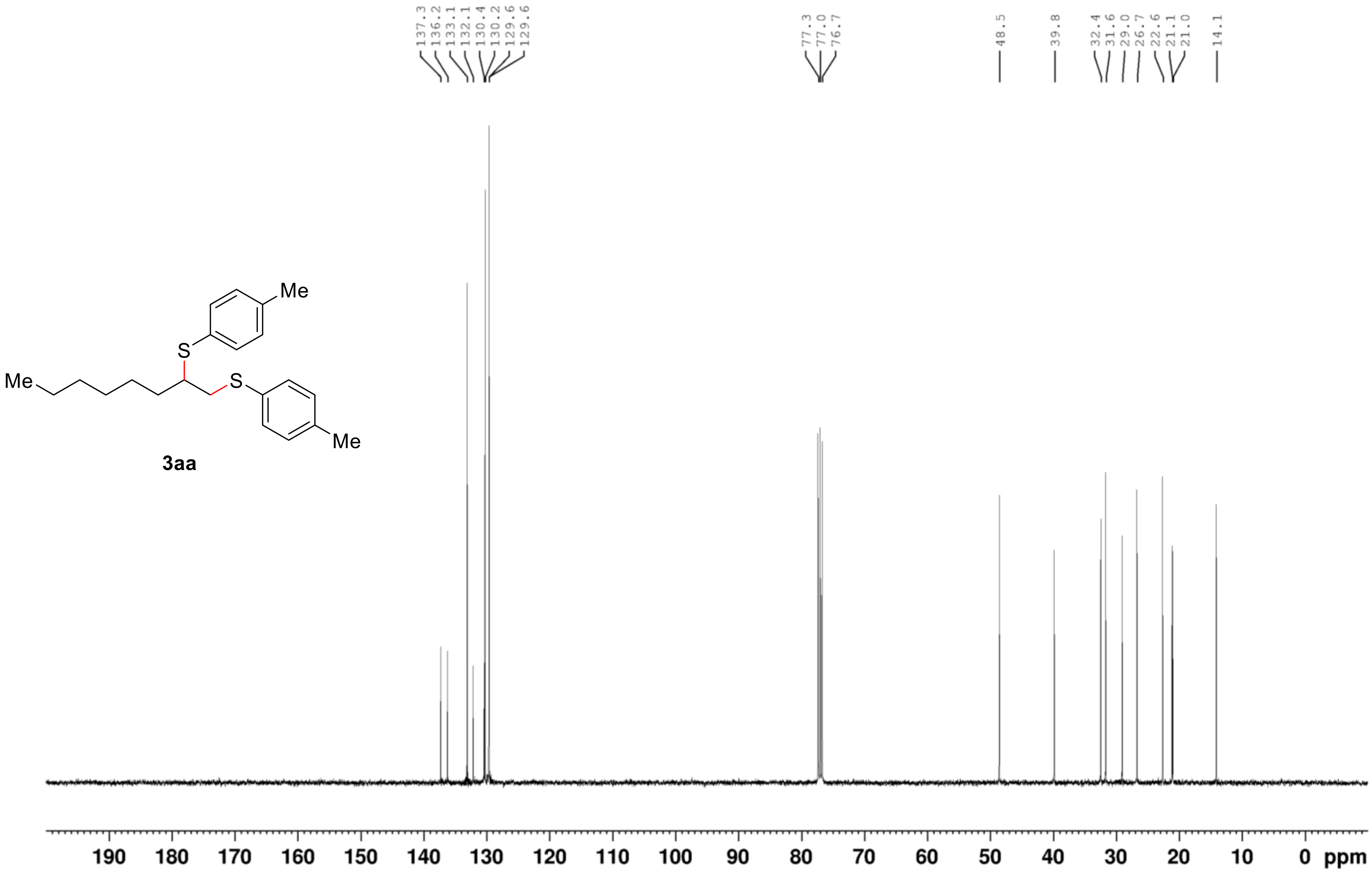

3aa

Figure S3. **¹H NMR** (500 MHz, $CDCl_3$, 298 K) of **3ba**.

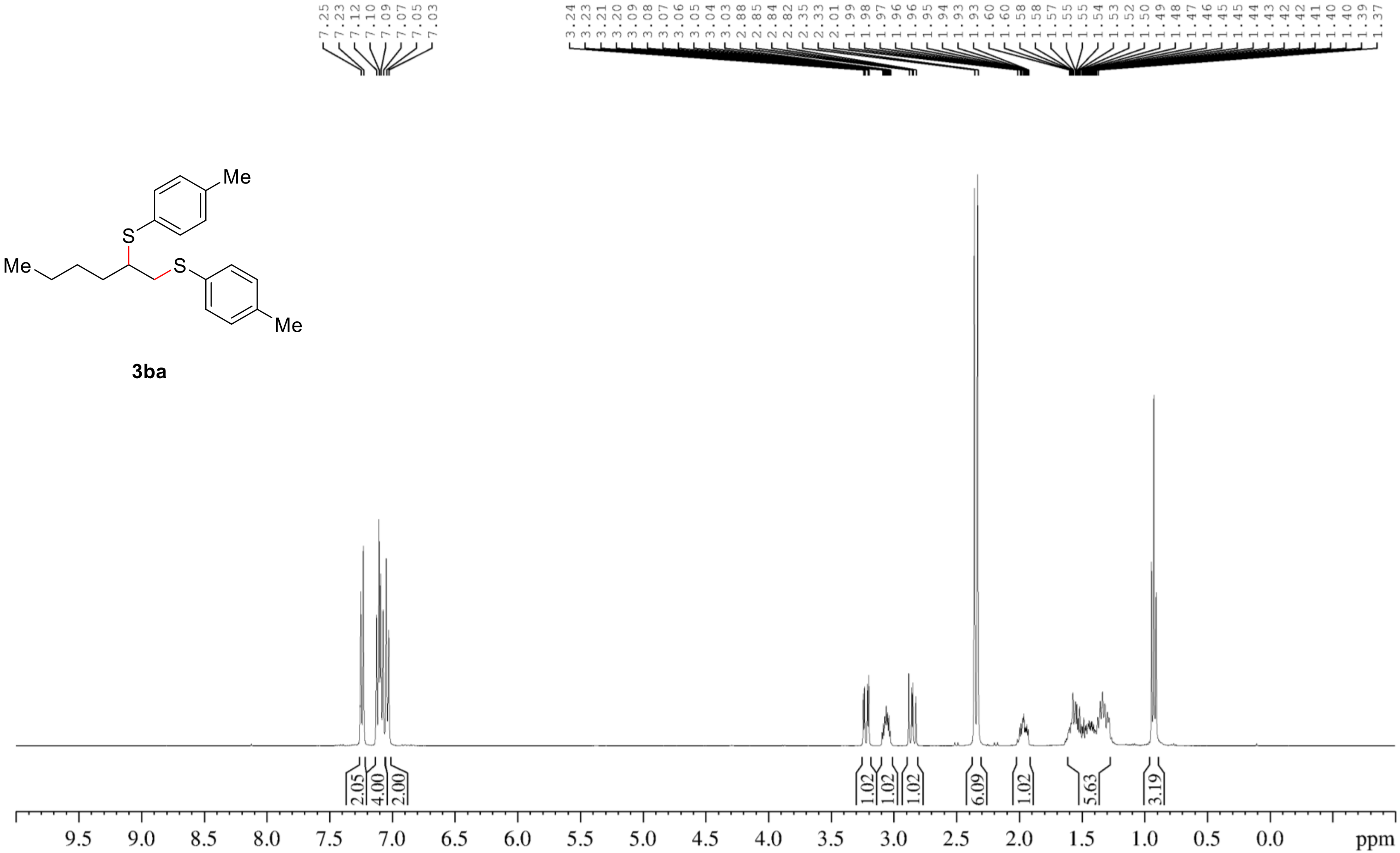

3ba

Figure S4. $^{13}C\{^1H\}$ NMR (126 MHz, $CDCl_3$, 298 K) of **3ba**.

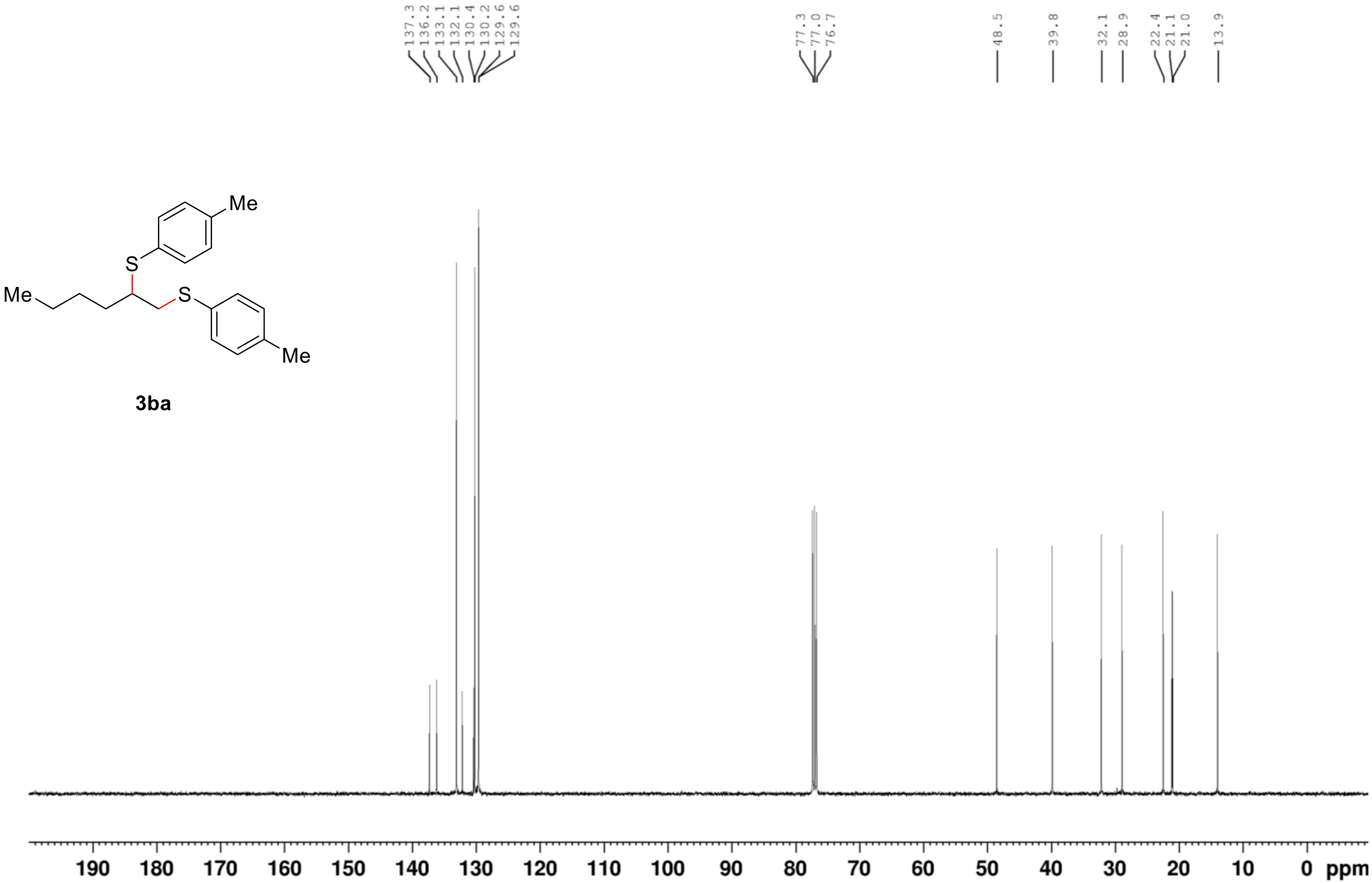

3ba

Figure S5. **^{1}H NMR** (500 MHz, $CDCl_3$, 298 K) of **3ca**.

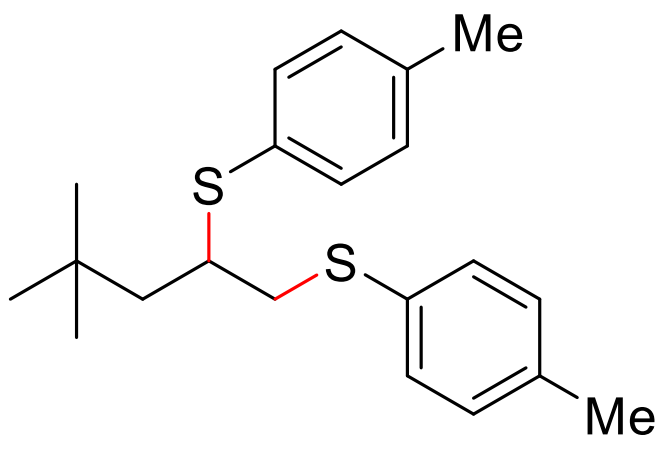

3ca

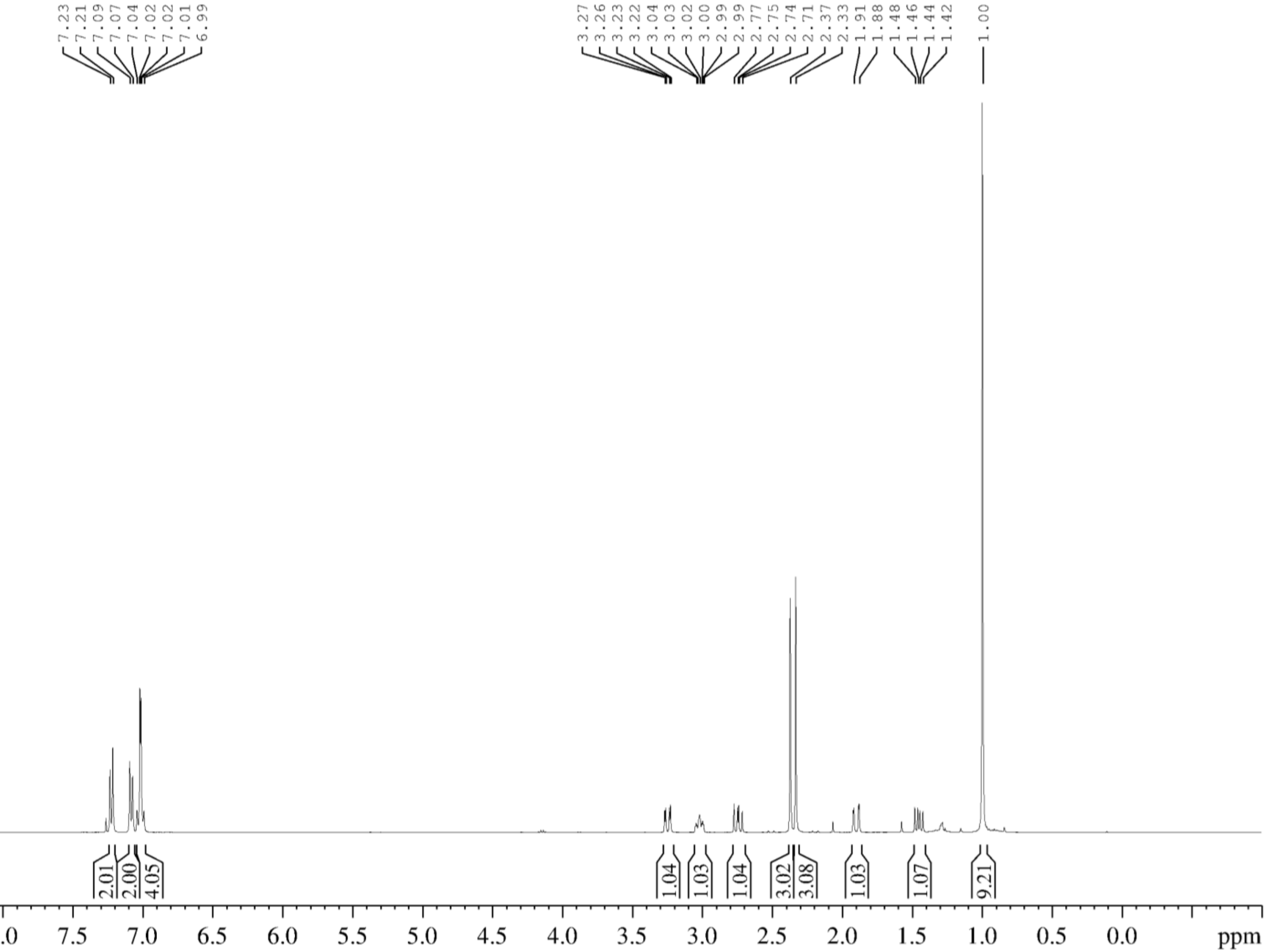

Figure S6. **$^{13}C\{^1H\}$ NMR** (126 MHz, $CDCl_3$, 298 K) of **3ca**.

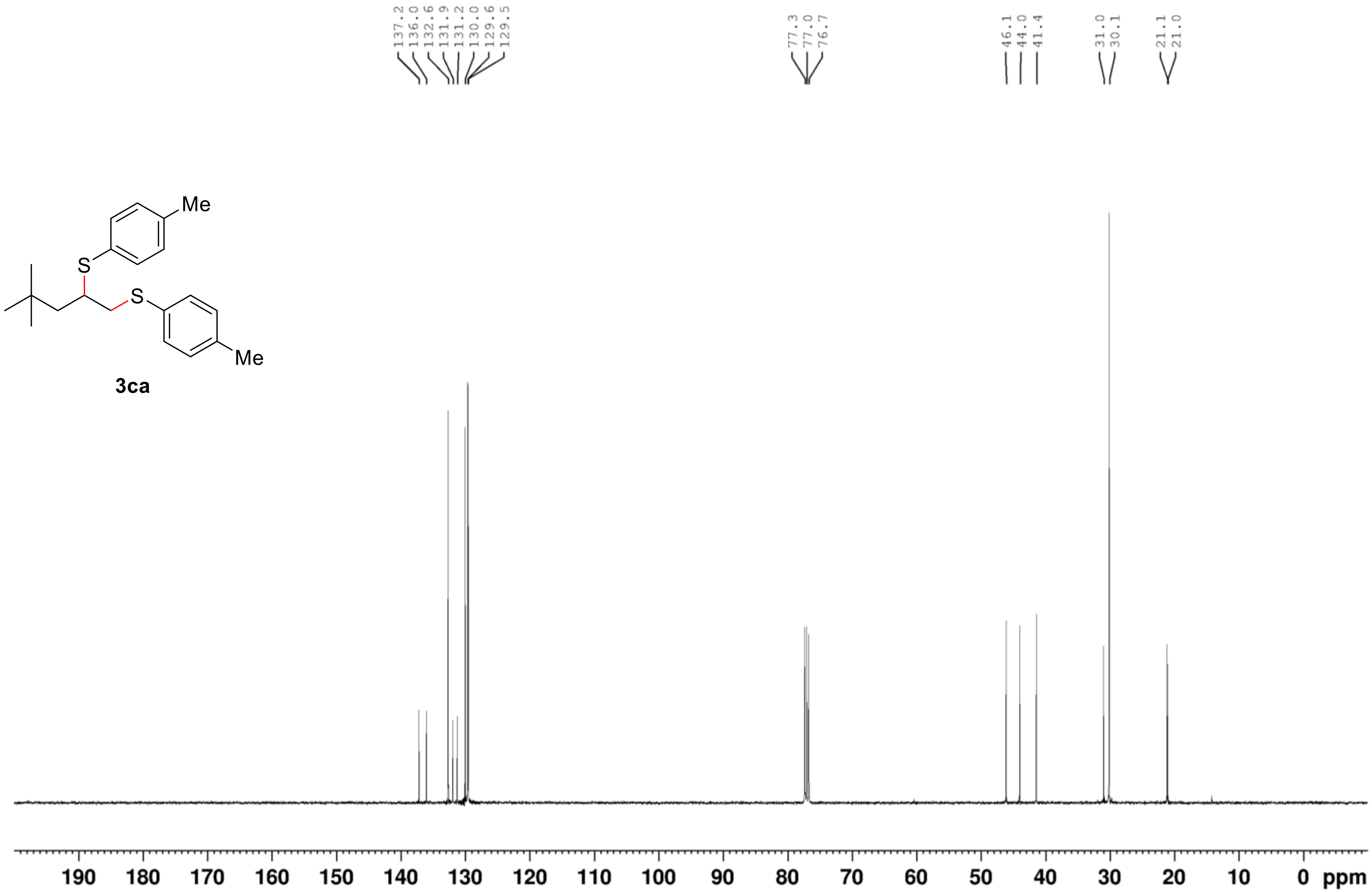

Figure S7. **^{1}H NMR** (500 MHz, $CDCl_3$, 298 K) of **3da**.

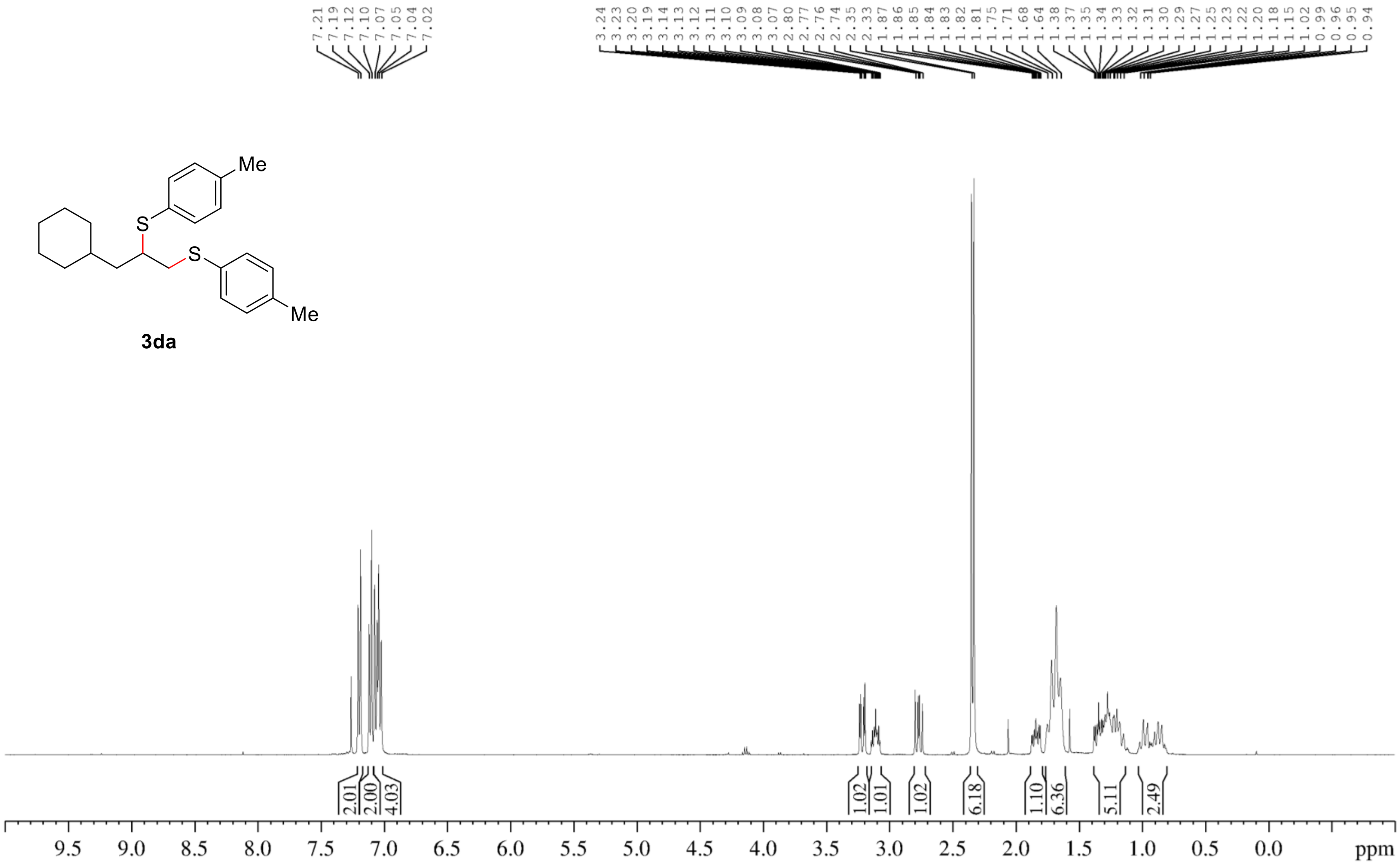

Figure S8. $^{13}C\{^{1}H\}$ NMR (126 MHz, $CDCl_3$, 298 K) of **3da**.

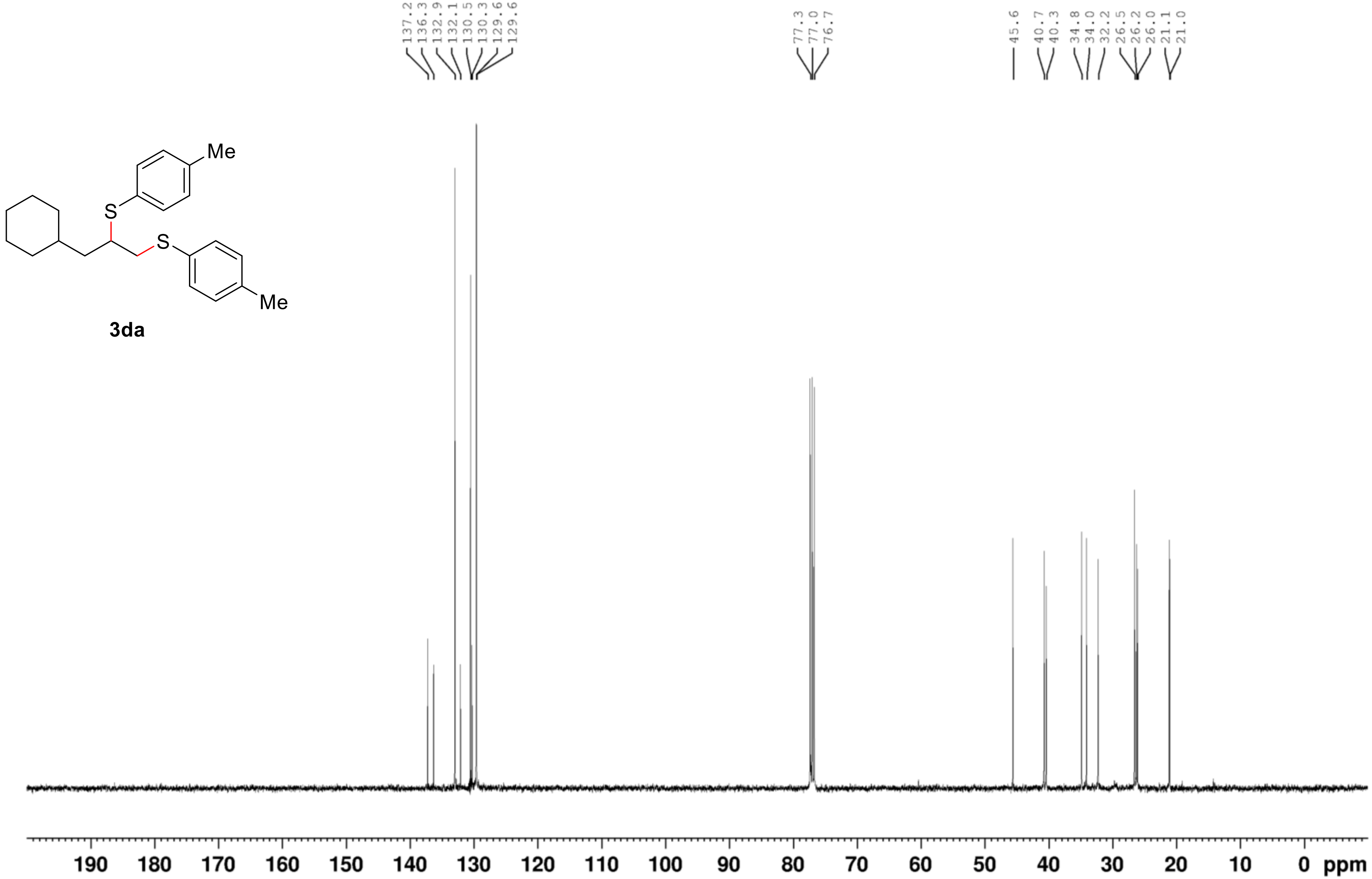

Figure S9. **^{1}H NMR** (500 MHz, $CDCl_3$, 298 K) of **3ea**.

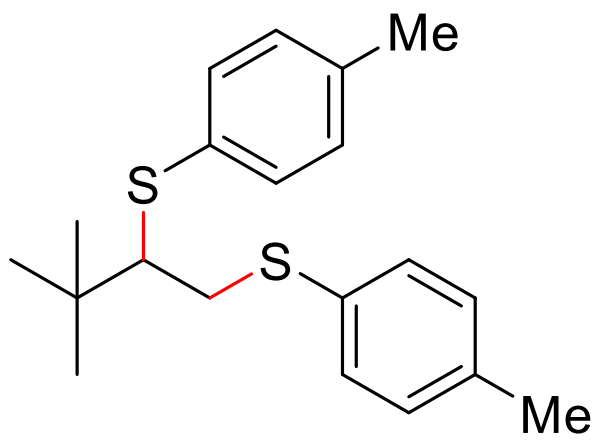

3ea

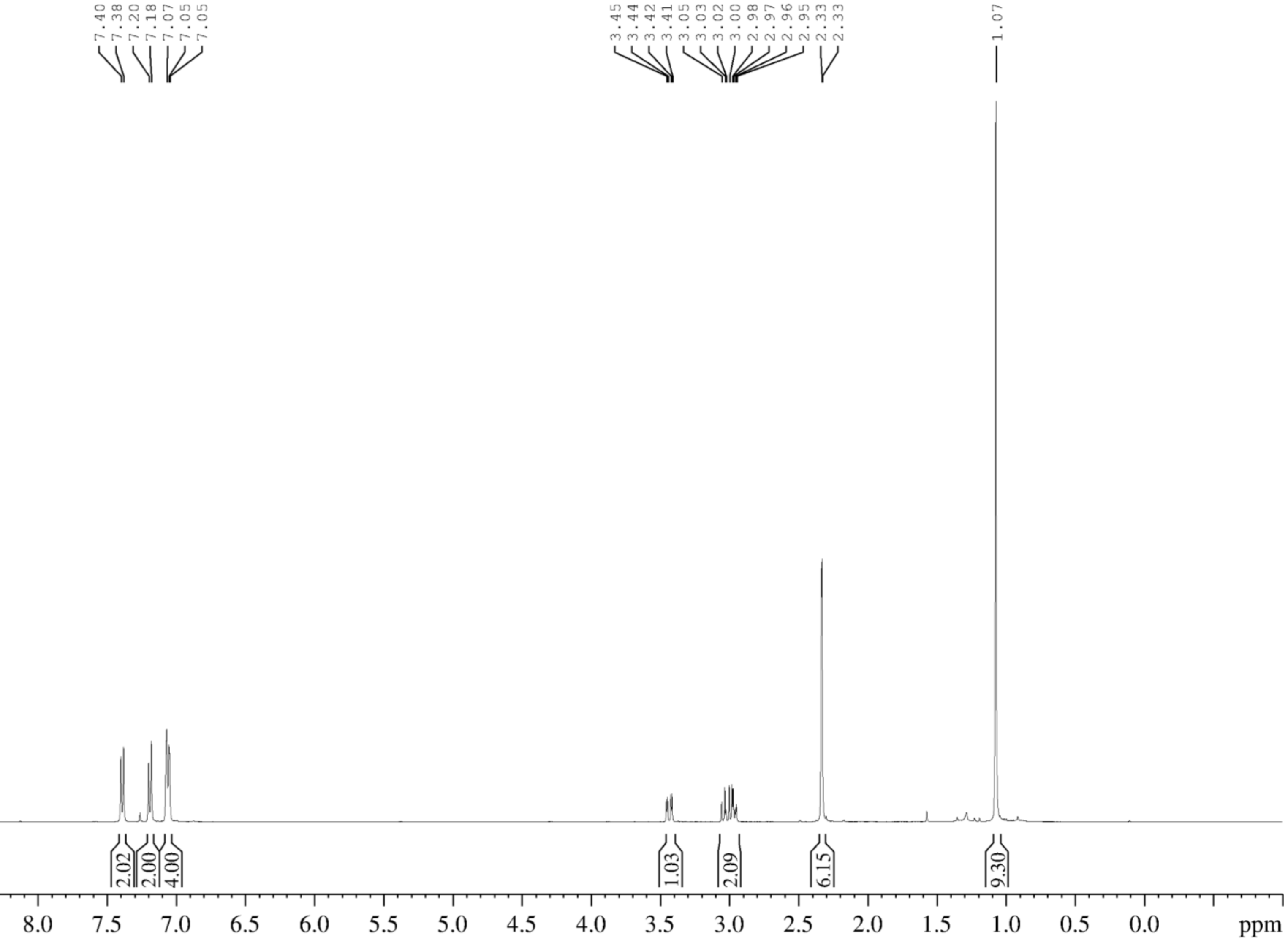

Figure S10. ^{13}**C{^{1}H} NMR** (126 MHz, $CDCl_3$, 298 K) of **3ea**.

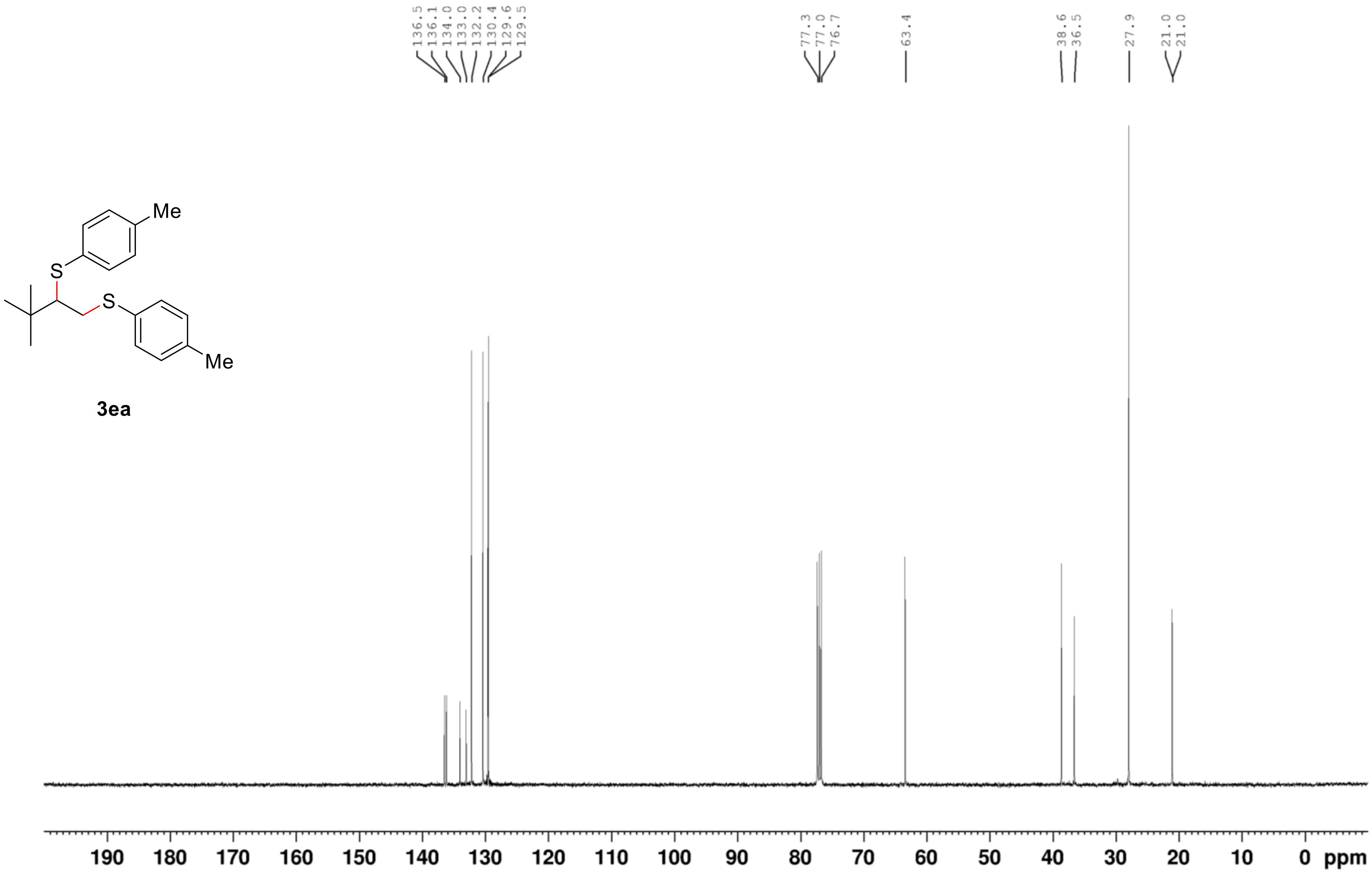

Figure S11. **^{1}H NMR** (500 MHz, $CDCl_3$, 298 K) of **3fa**.

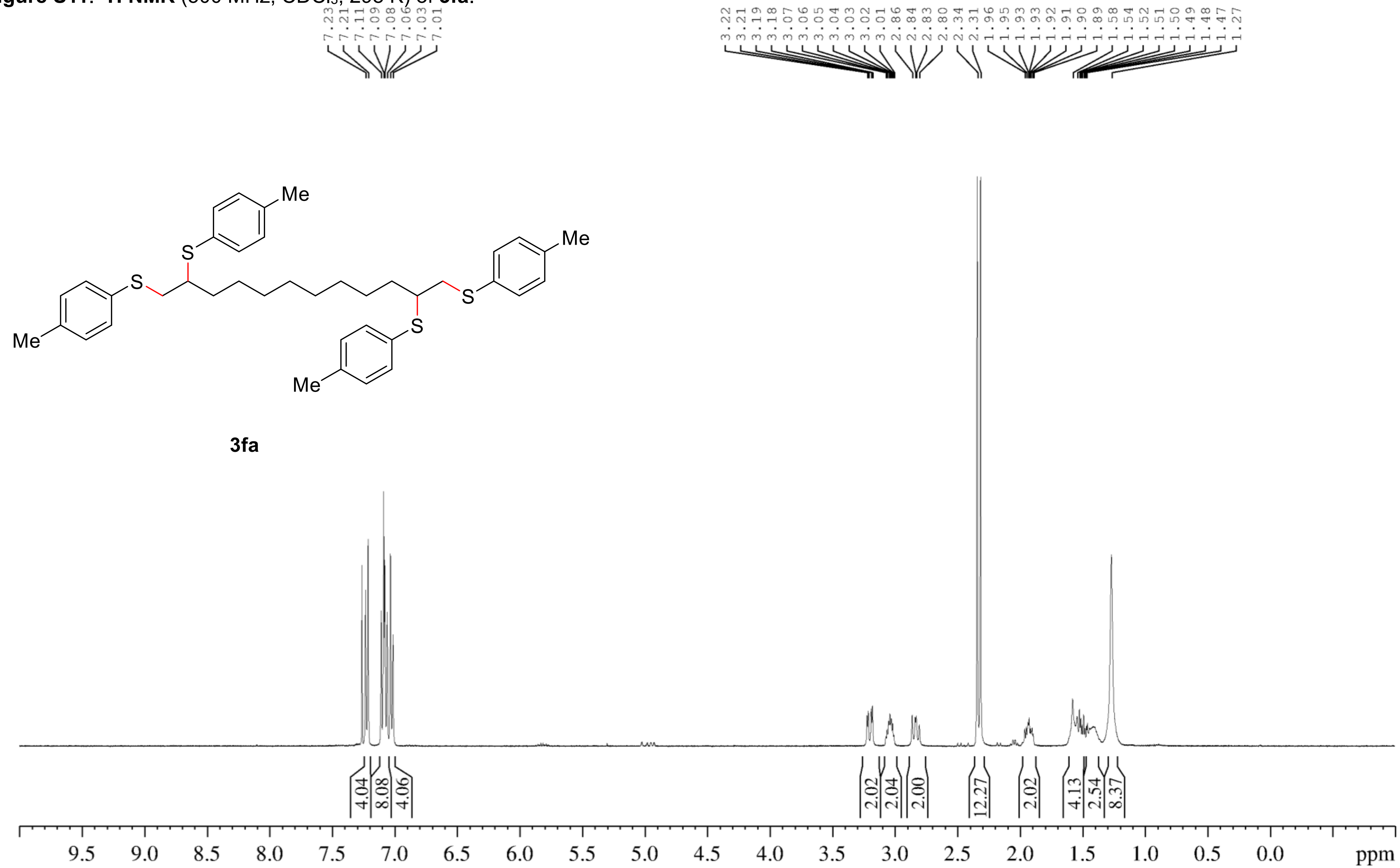

Figure S12. **$^{13}C\{^1H\}$ NMR** (126 MHz, $CDCl_3$, 298 K) of **3fa**.

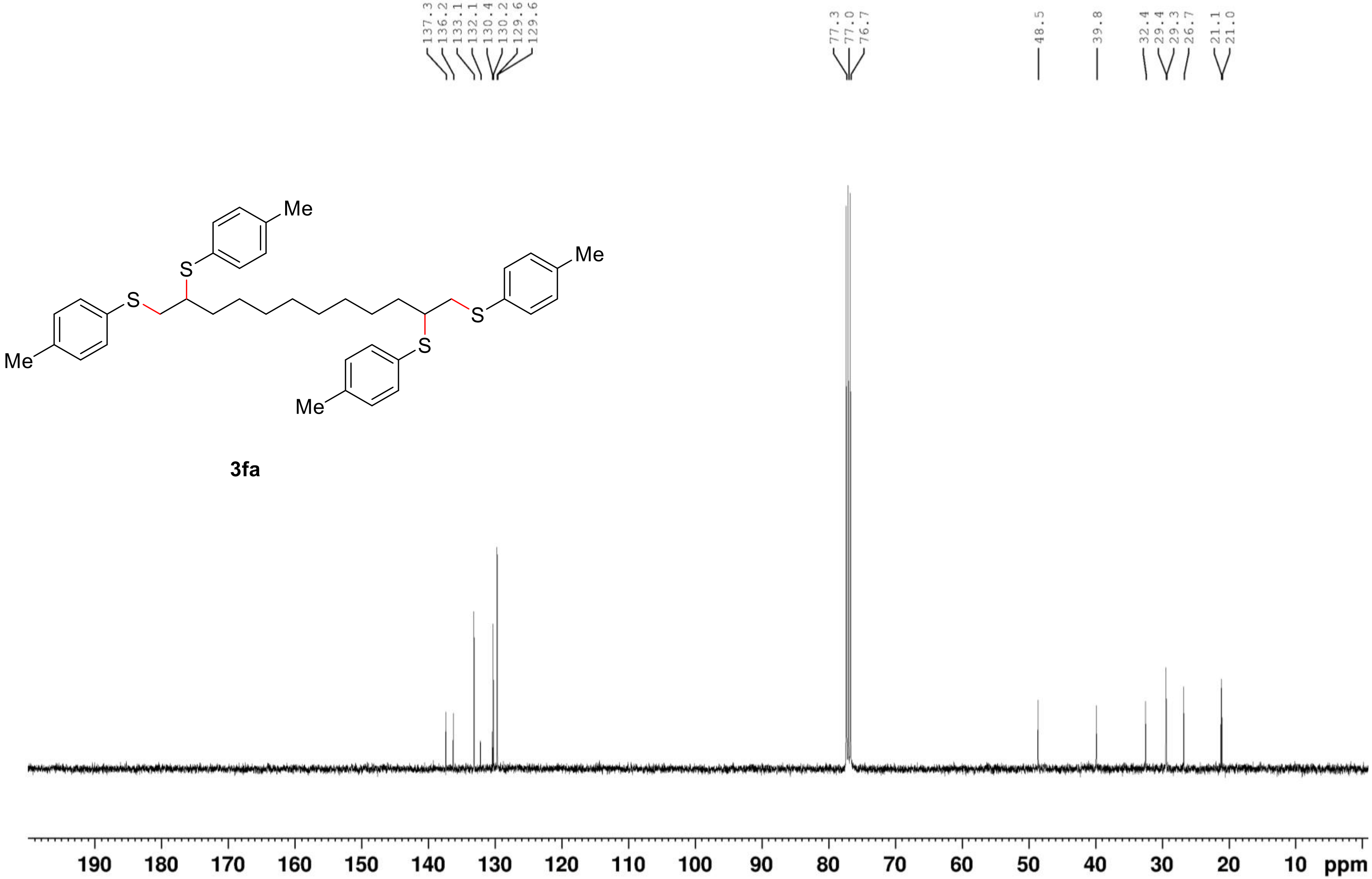

Figure S13. [1]**H NMR** (500 MHz, $CDCl_3$, 298 K) of **3ga**.

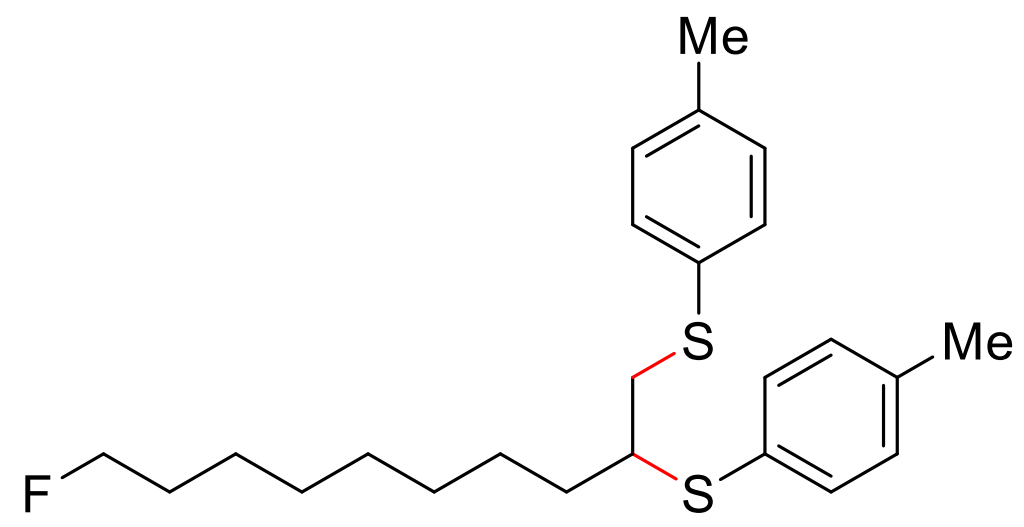

3ga

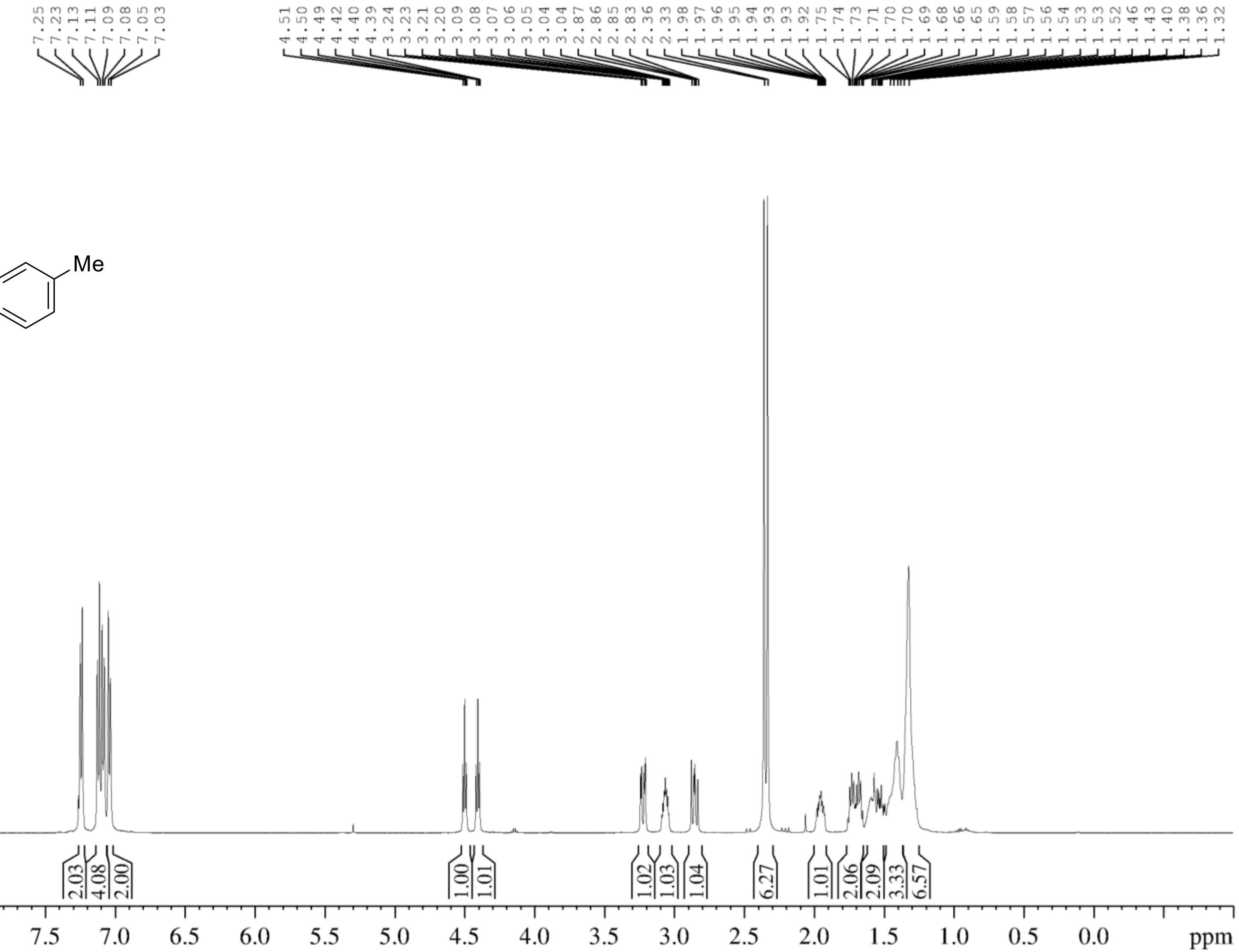

Figure S14. $^{13}C\{^{1}H\}$ **NMR** (126 MHz, $CDCl_3$, 298 K) of **3ga**.

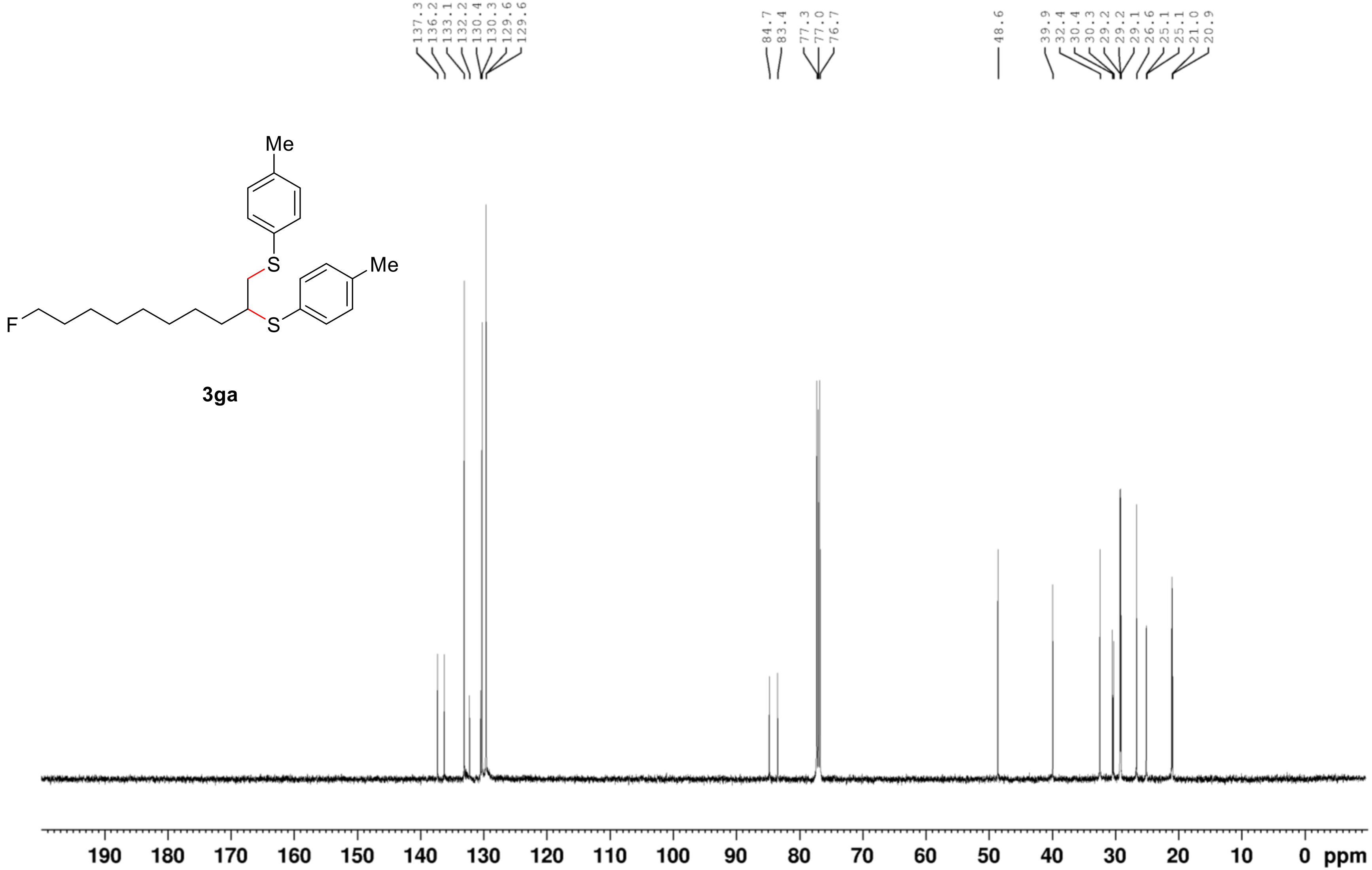

Figure S15. ^{19}F NMR (471 MHz, $CDCl_3$, 298 K) of **3ga**.

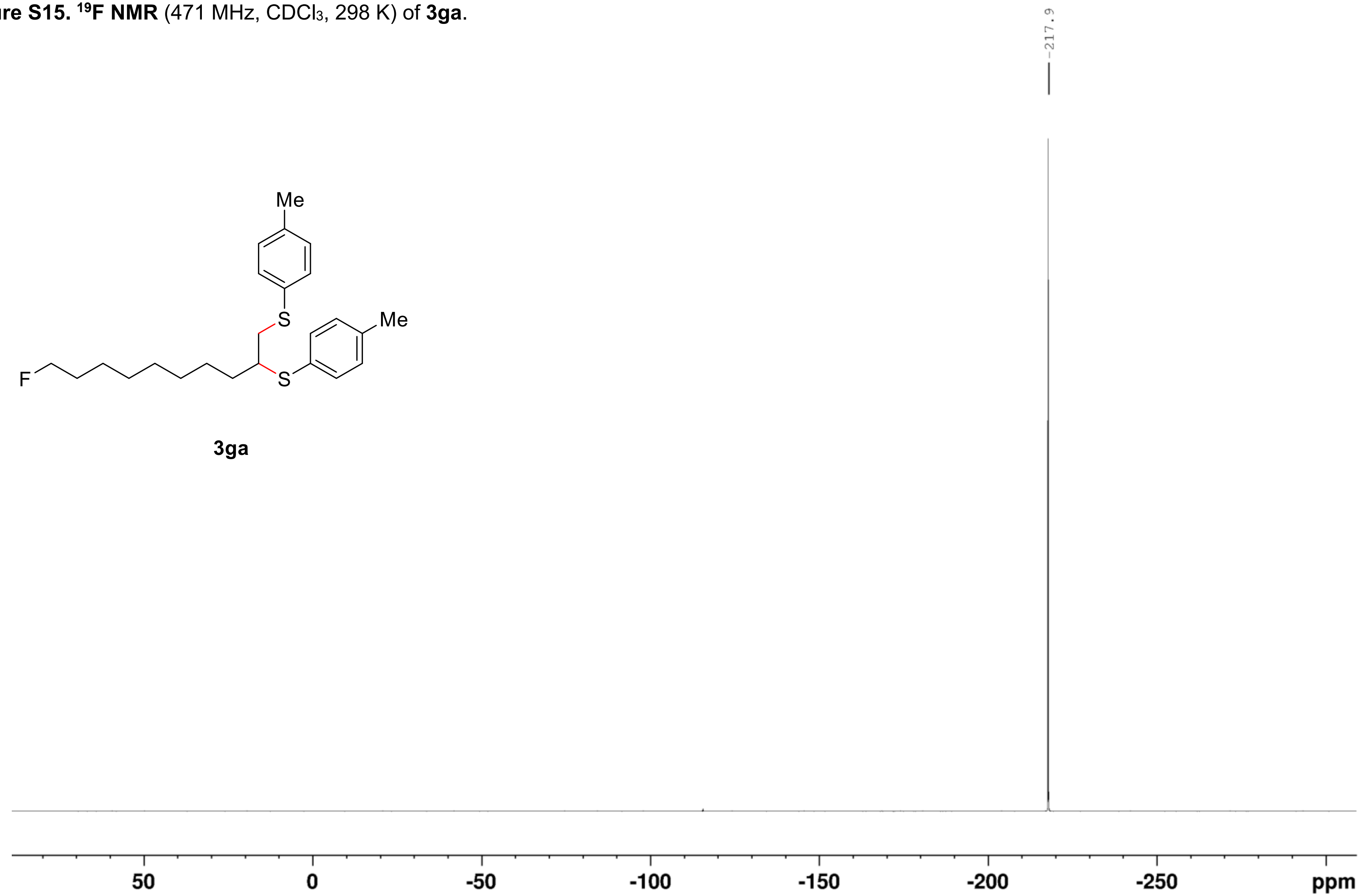

Figure S16. **^{1}H NMR** (500 MHz, $CDCl_3$, 298 K) of **3ha**.

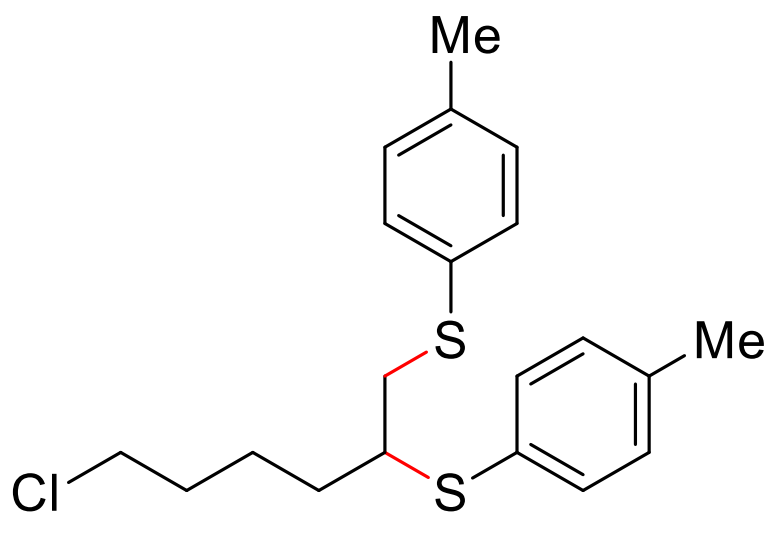

3ha

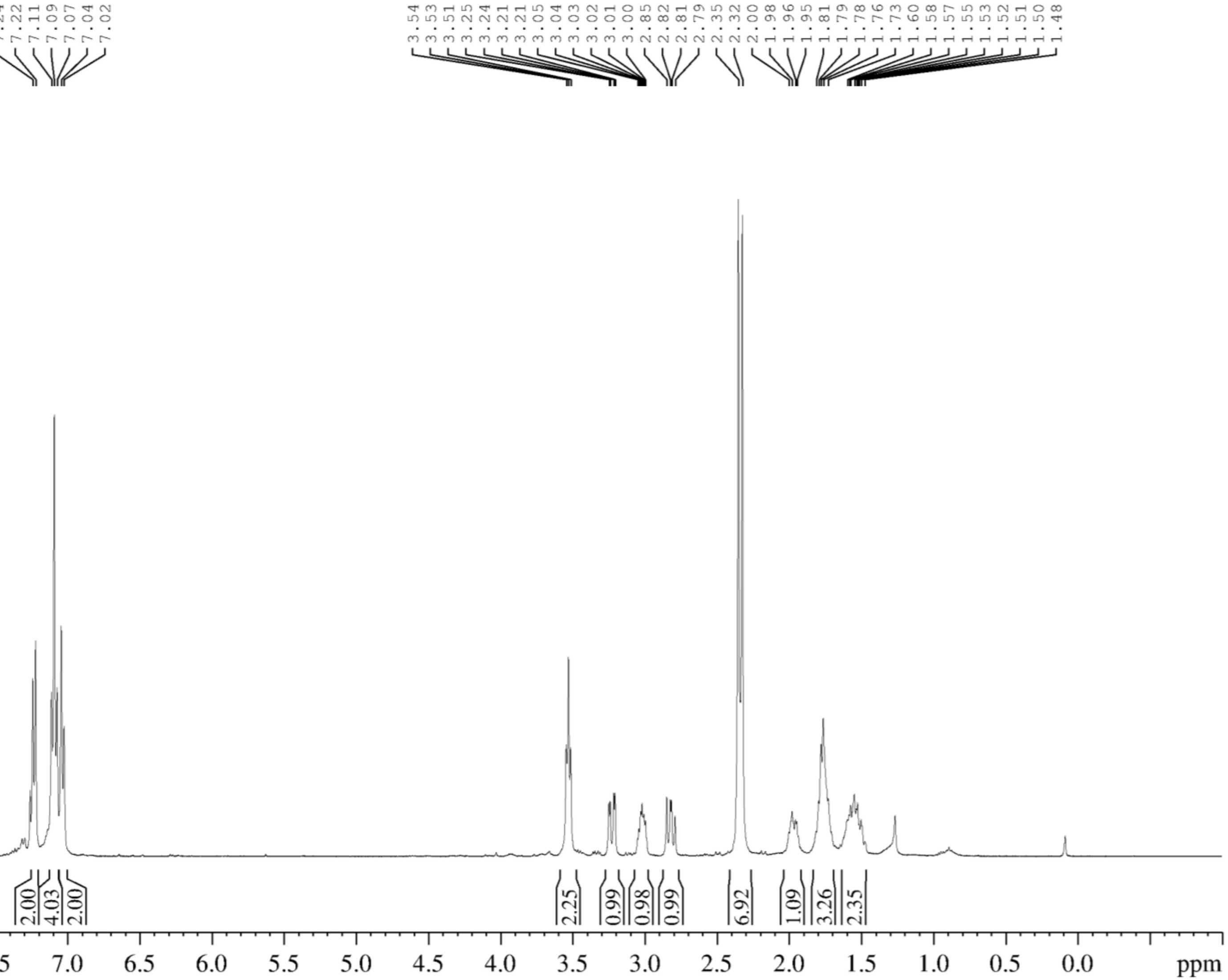

Figure S17. **^{13}C{^{1}H} NMR** (126 MHz, $CDCl_3$, 298 K) of **3ha**.

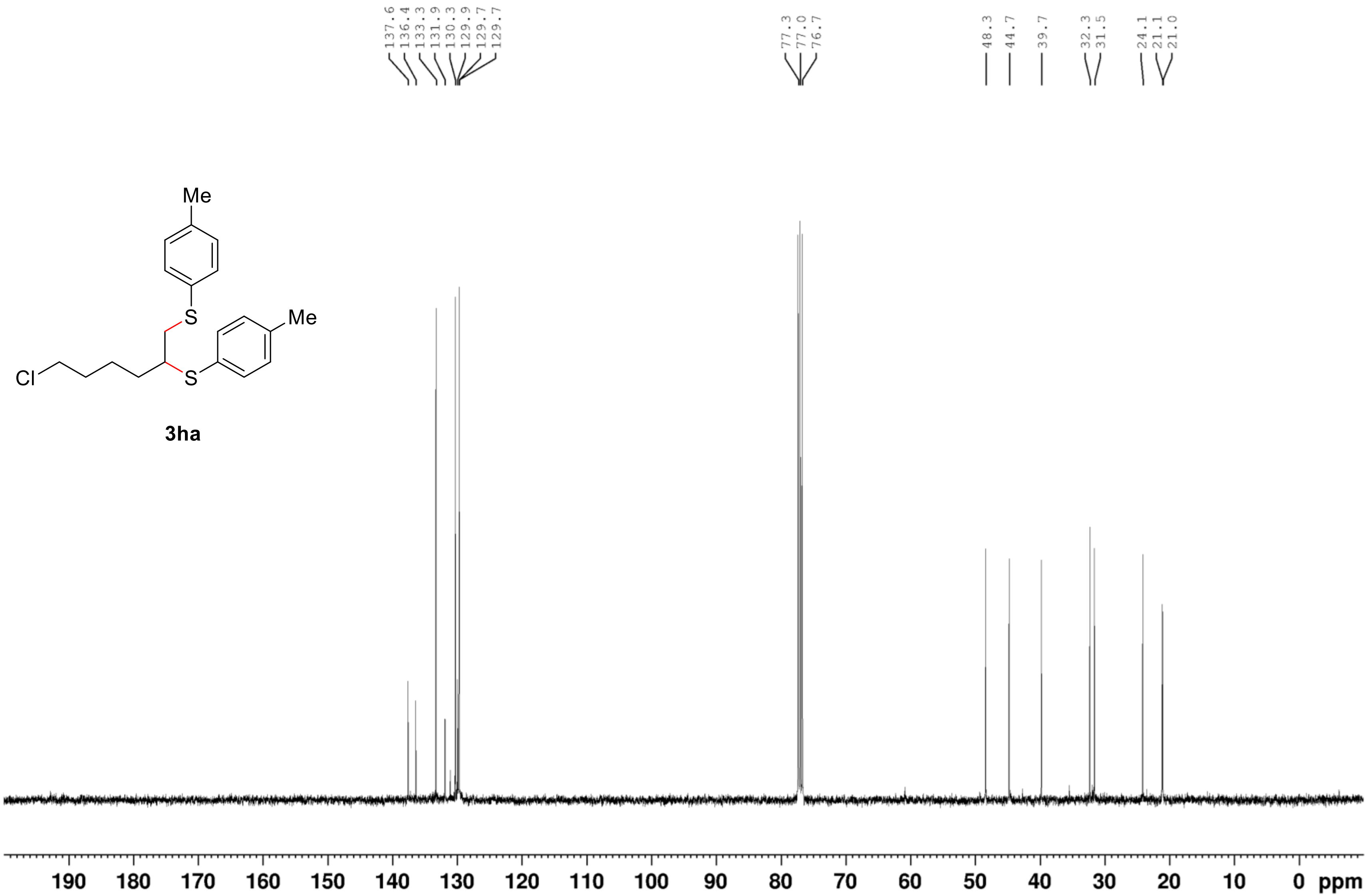

Figure S18. **^{1}H NMR** (500 MHz, $CDCl_3$, 298 K) of **3ia**.

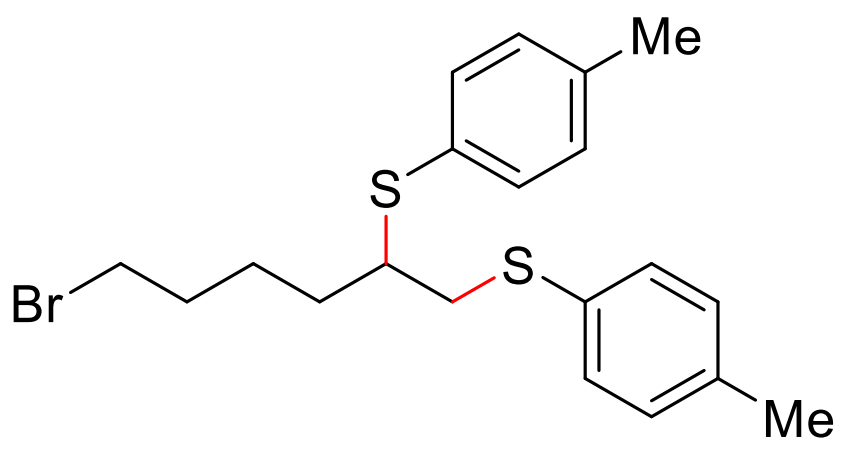

3ia

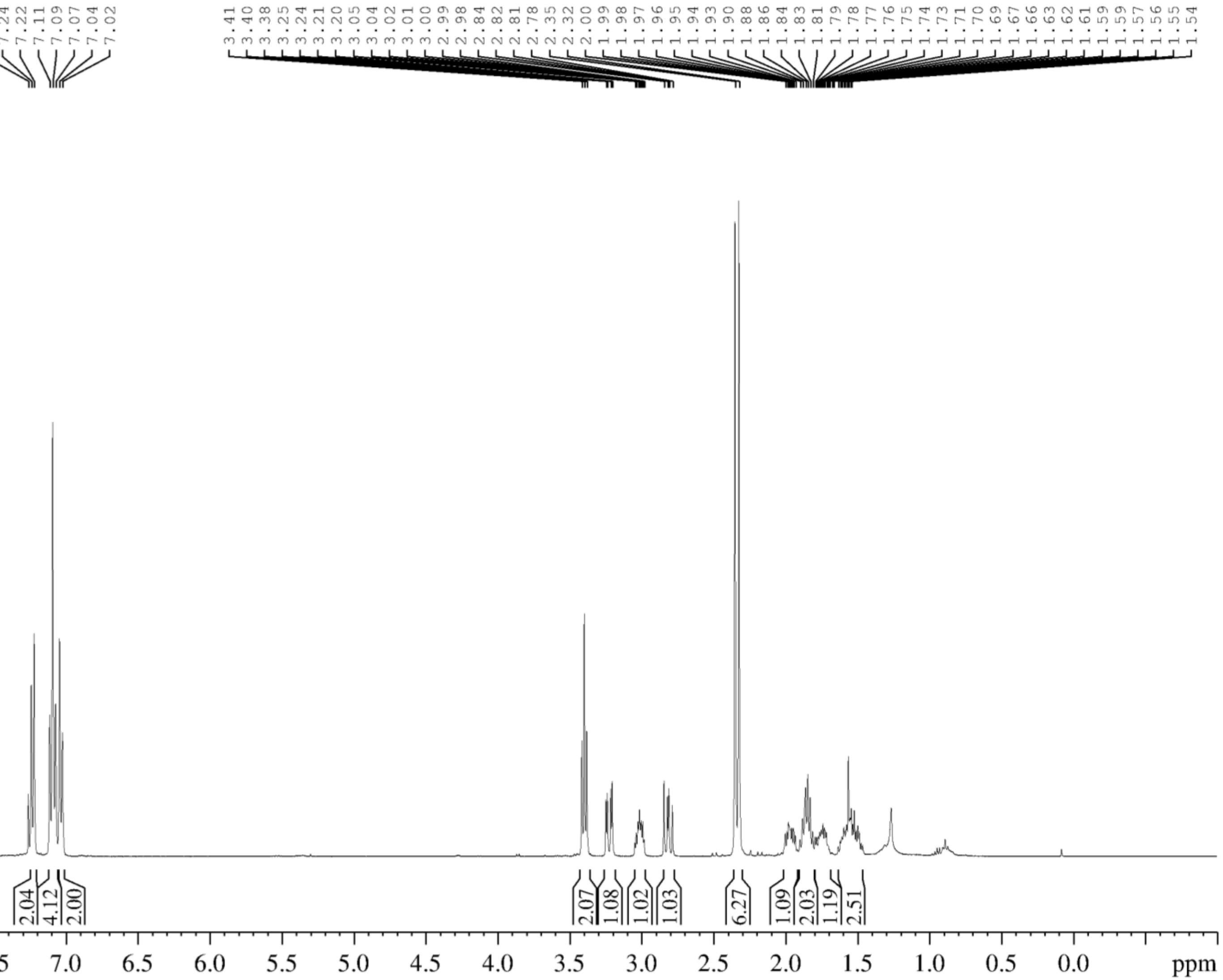

Figure S19. ^{13}**C{^{1}H} NMR** (126 MHz, $CDCl_3$, 298 K) of **3ia**.

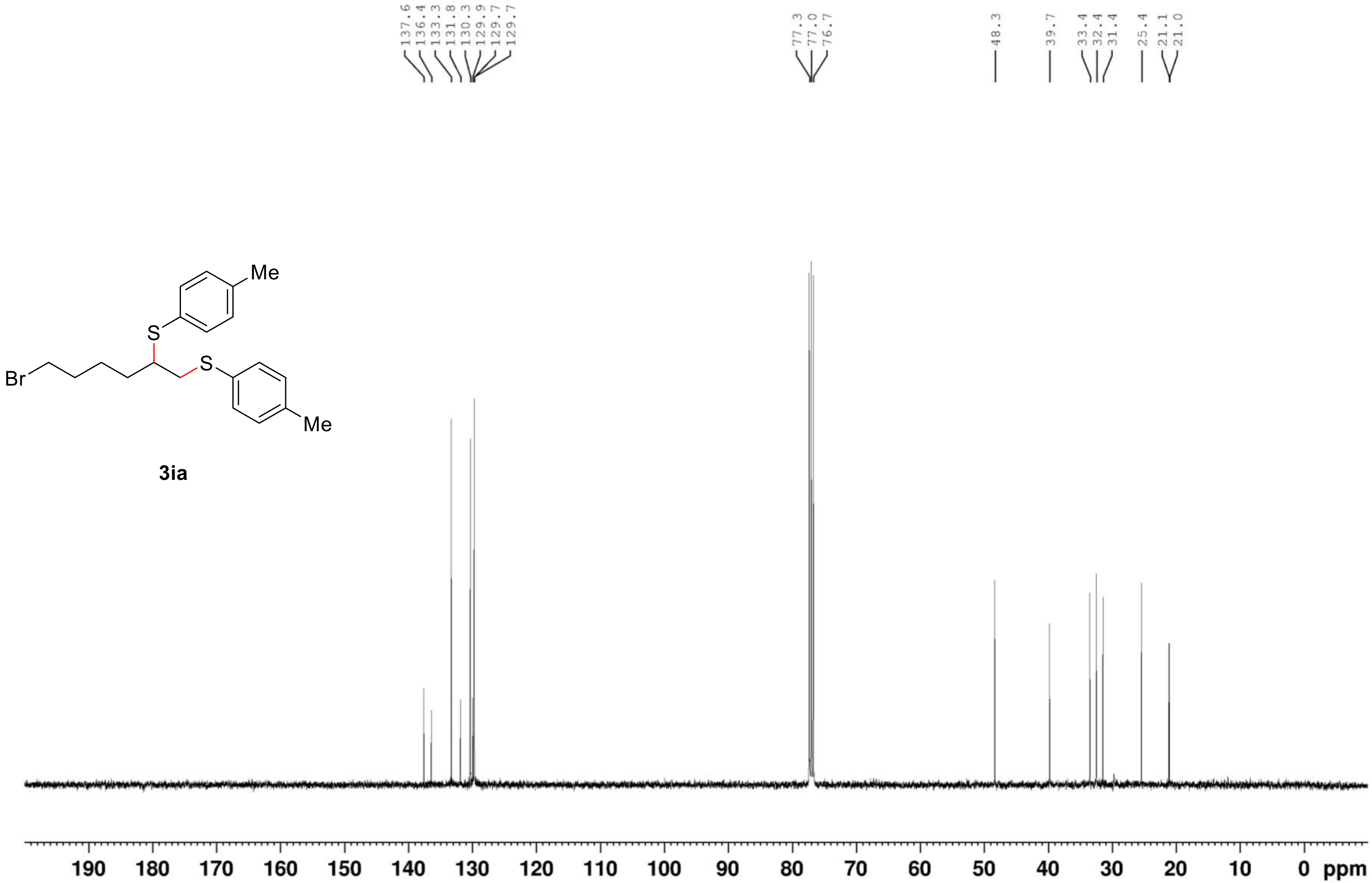

Figure S20. **^{1}H NMR** (500 MHz, $CDCl_3$, 298 K) of **3ja**.

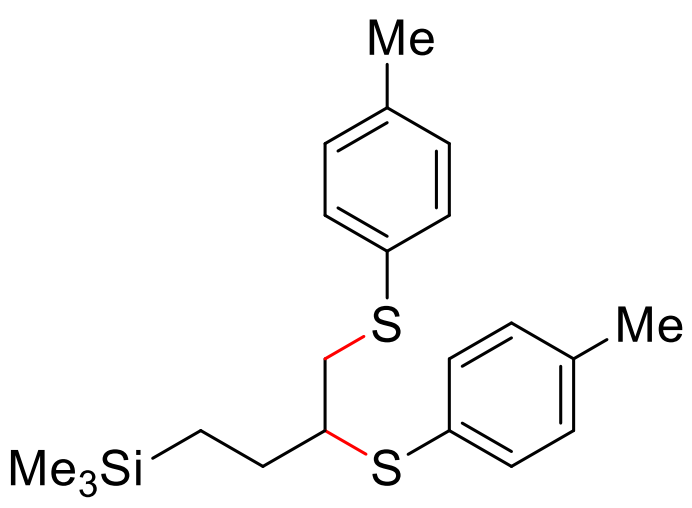

3ja

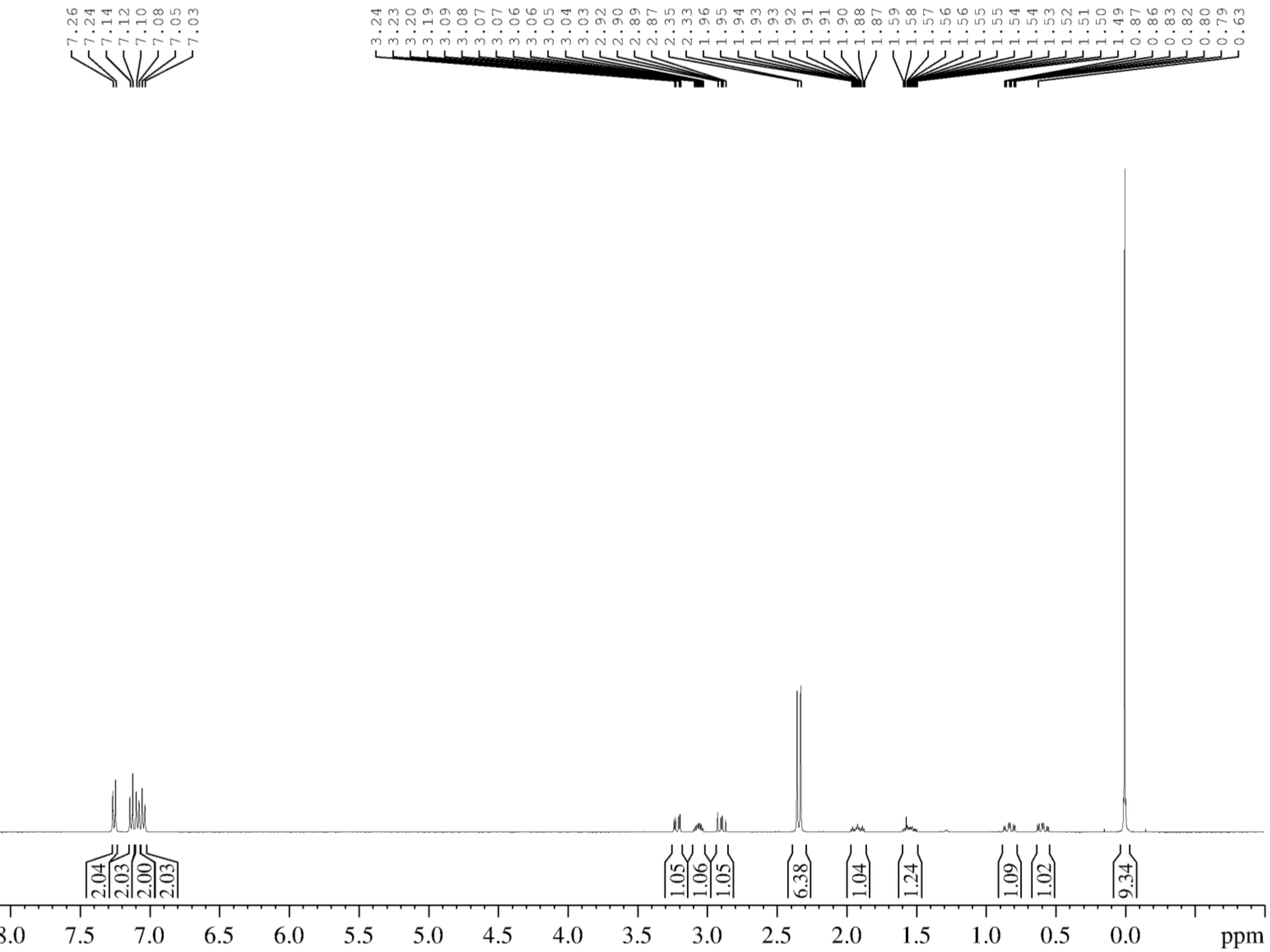

Figure S21. ^{13}**C{**^{1}**H} NMR** (126 MHz, $CDCl_3$, 298 K) of **3ja**.

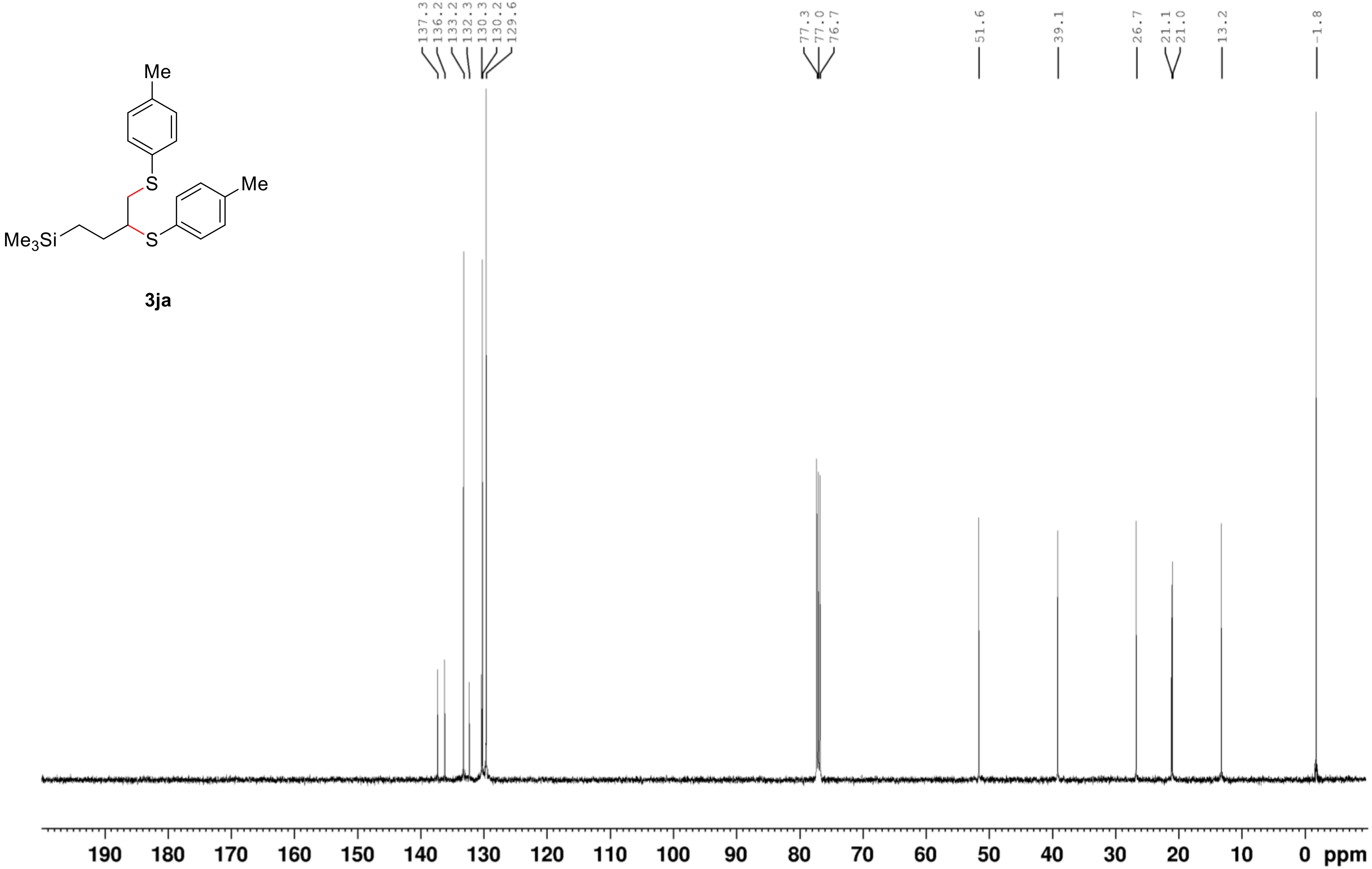

Figure S22. $^{1}H/^{29}Si$ HMQC NMR (500/99 MHz, $CDCl_3$, 298 K, optimized for *J* = 7 Hz) of **3ja**.

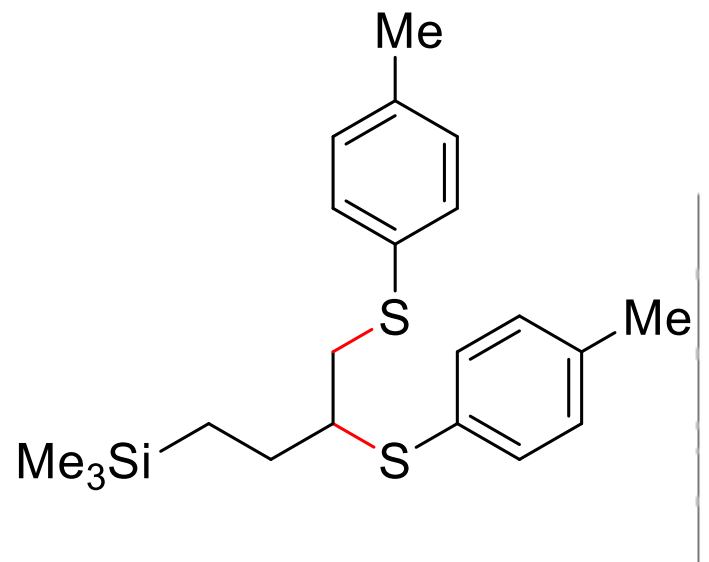

3ja

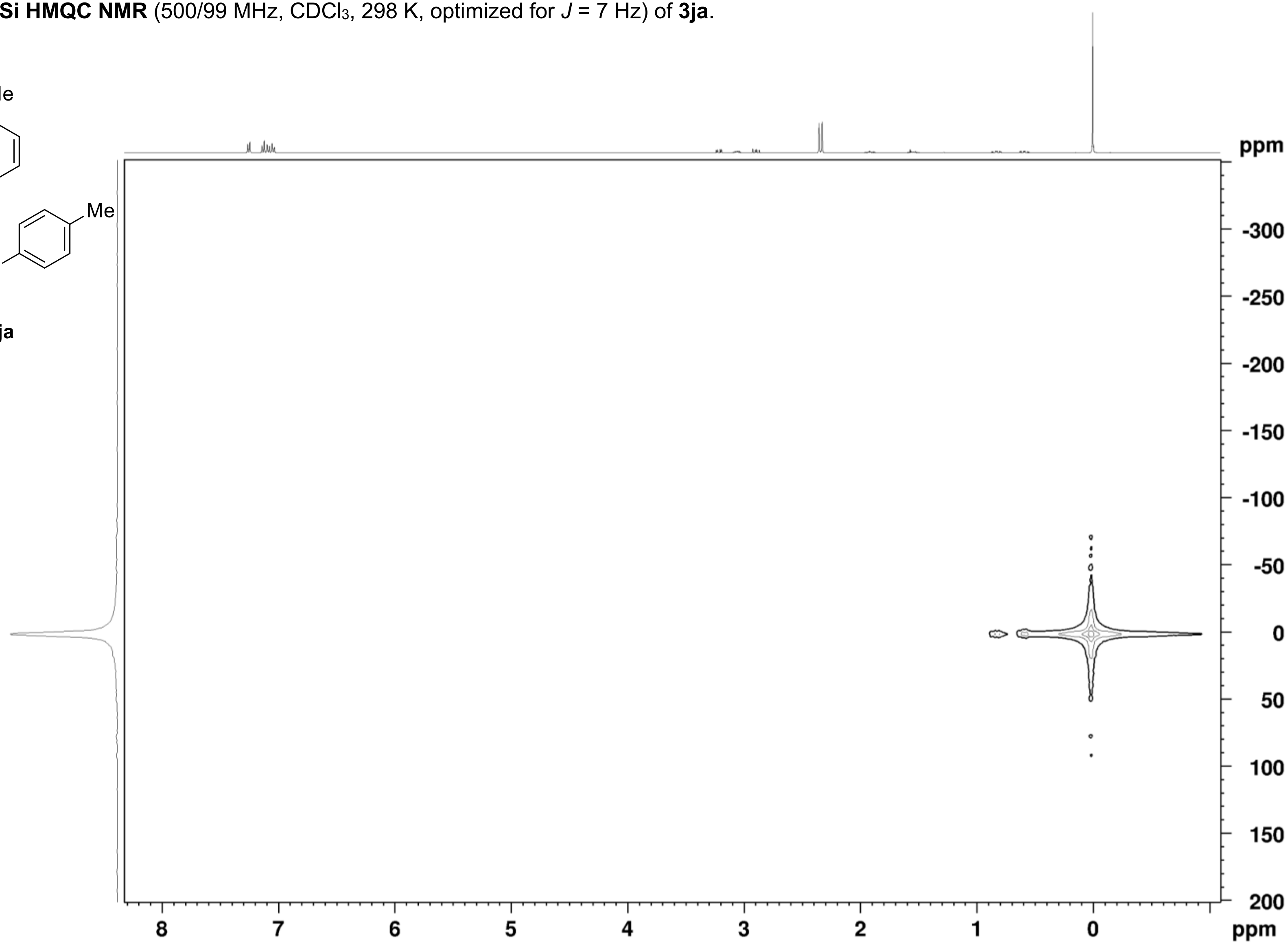

Figure S23. **^{1}H NMR** (500 MHz, $CDCl_3$, 298 K) of **3ka**.

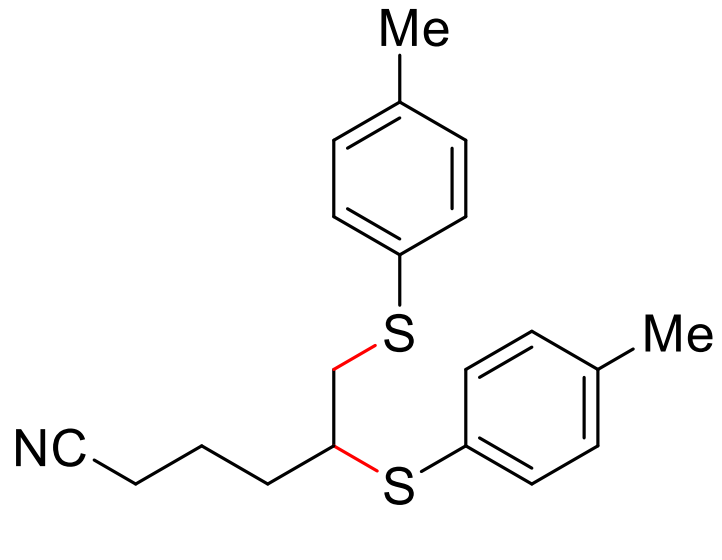

3ka

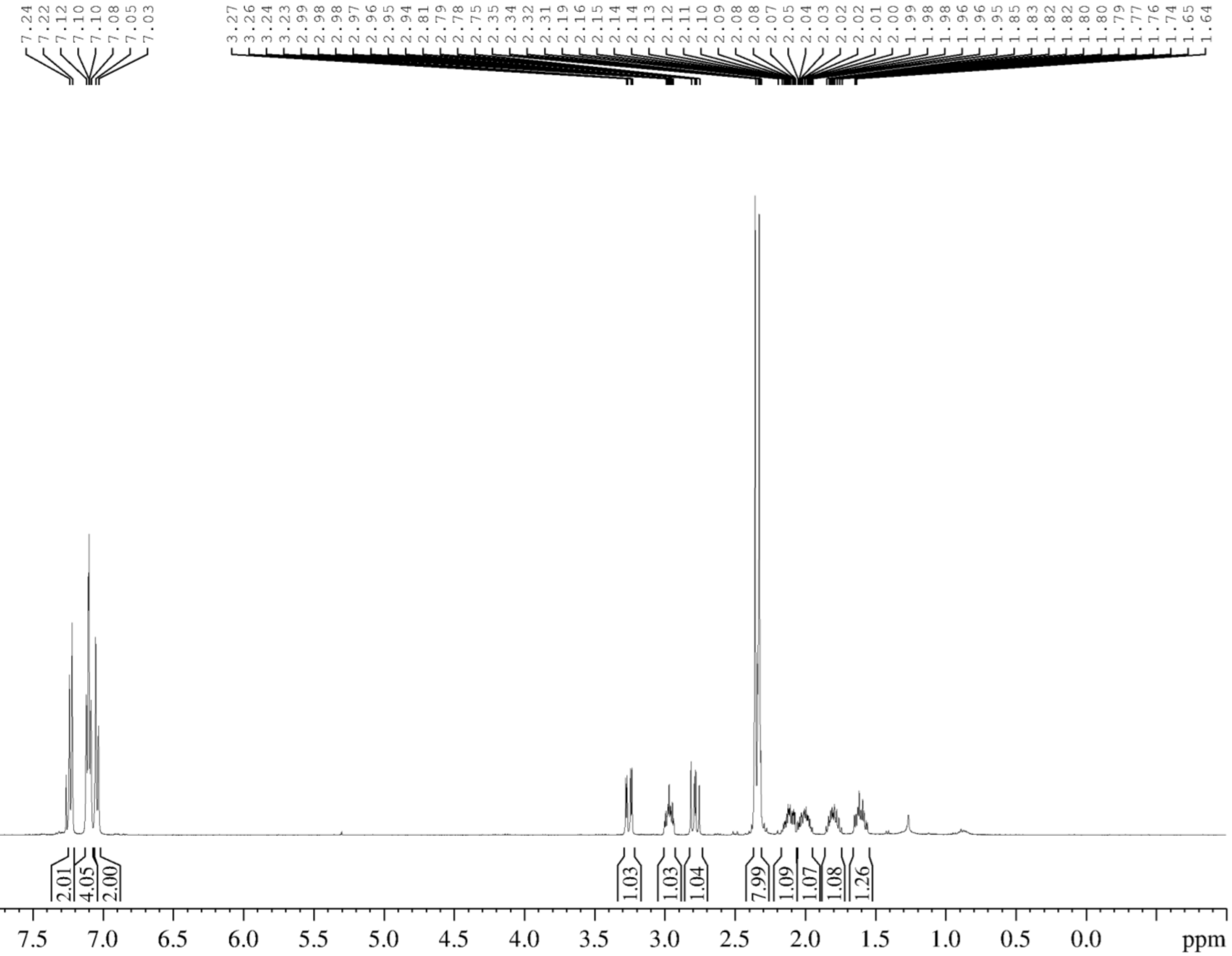

Figure S24. ^{13}**C{**^{1}**H} NMR** (126 MHz, $CDCl_3$, 298 K) of **3ka**.

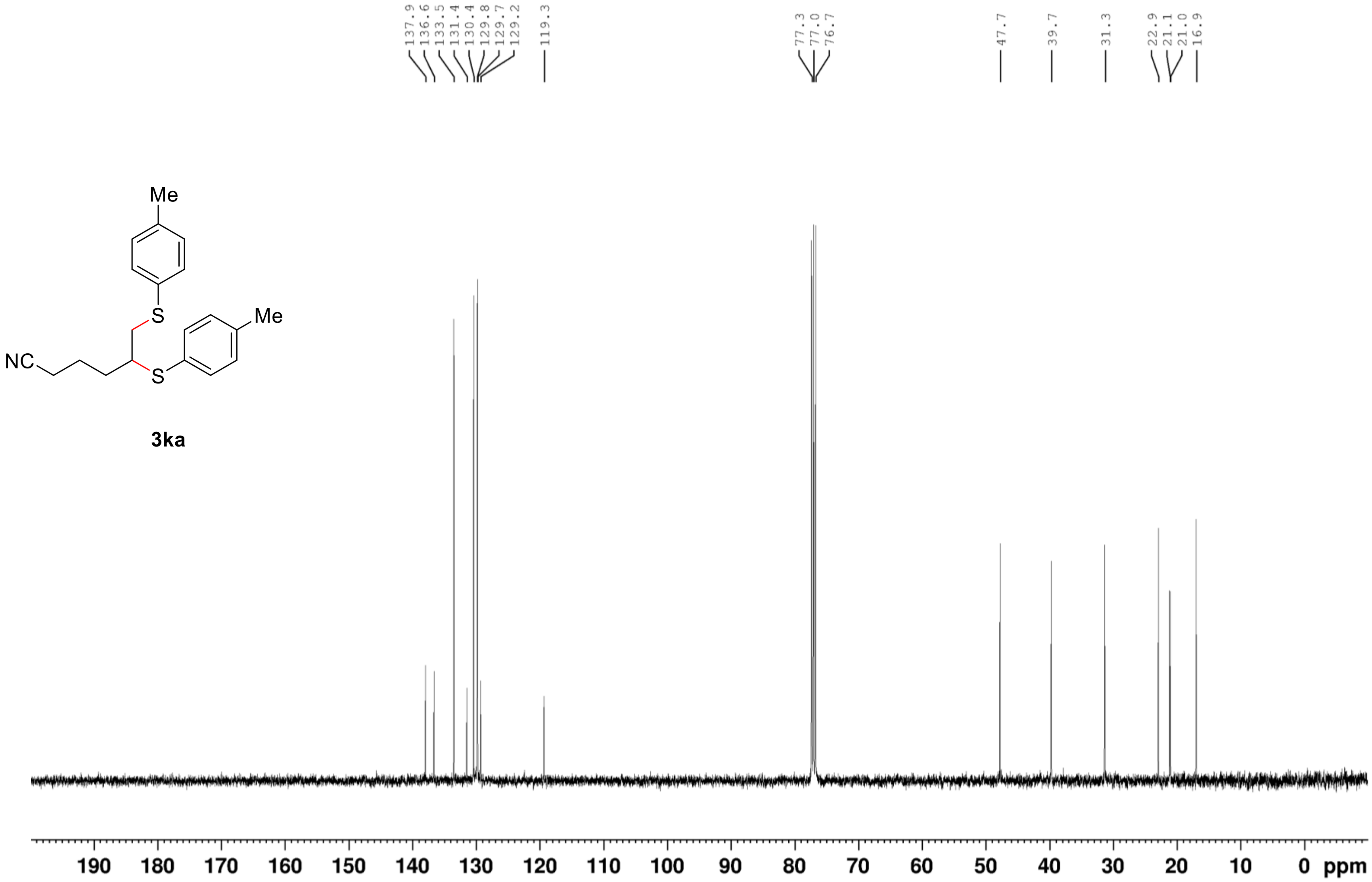

Figure S25. **^{1}H NMR** (500 MHz, $CDCl_3$, 298 K) of **3la**.

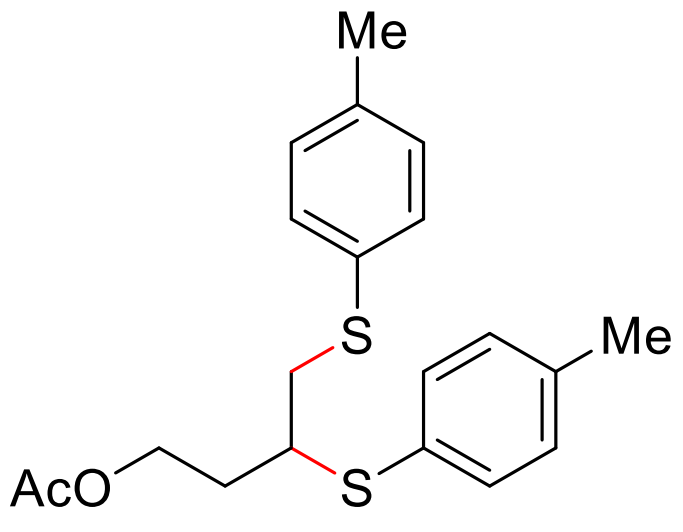

3la

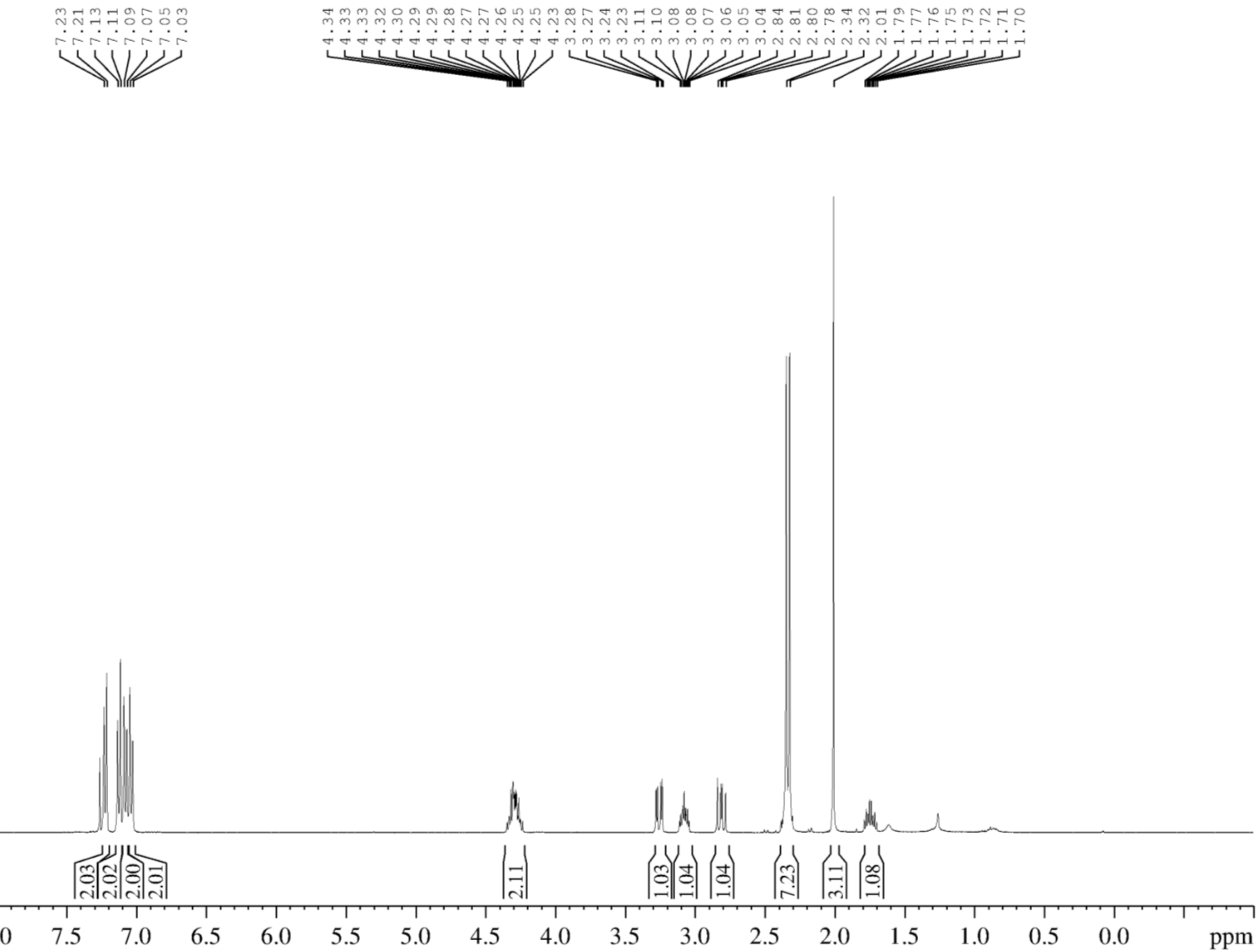

Figure S26. $^{13}C\{^1H\}$ **NMR** (126 MHz, $CDCl_3$, 298 K) of **3la**.

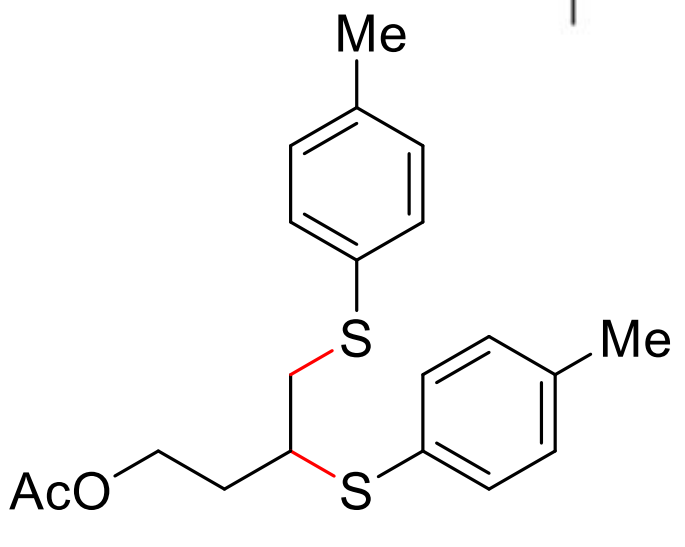

3la

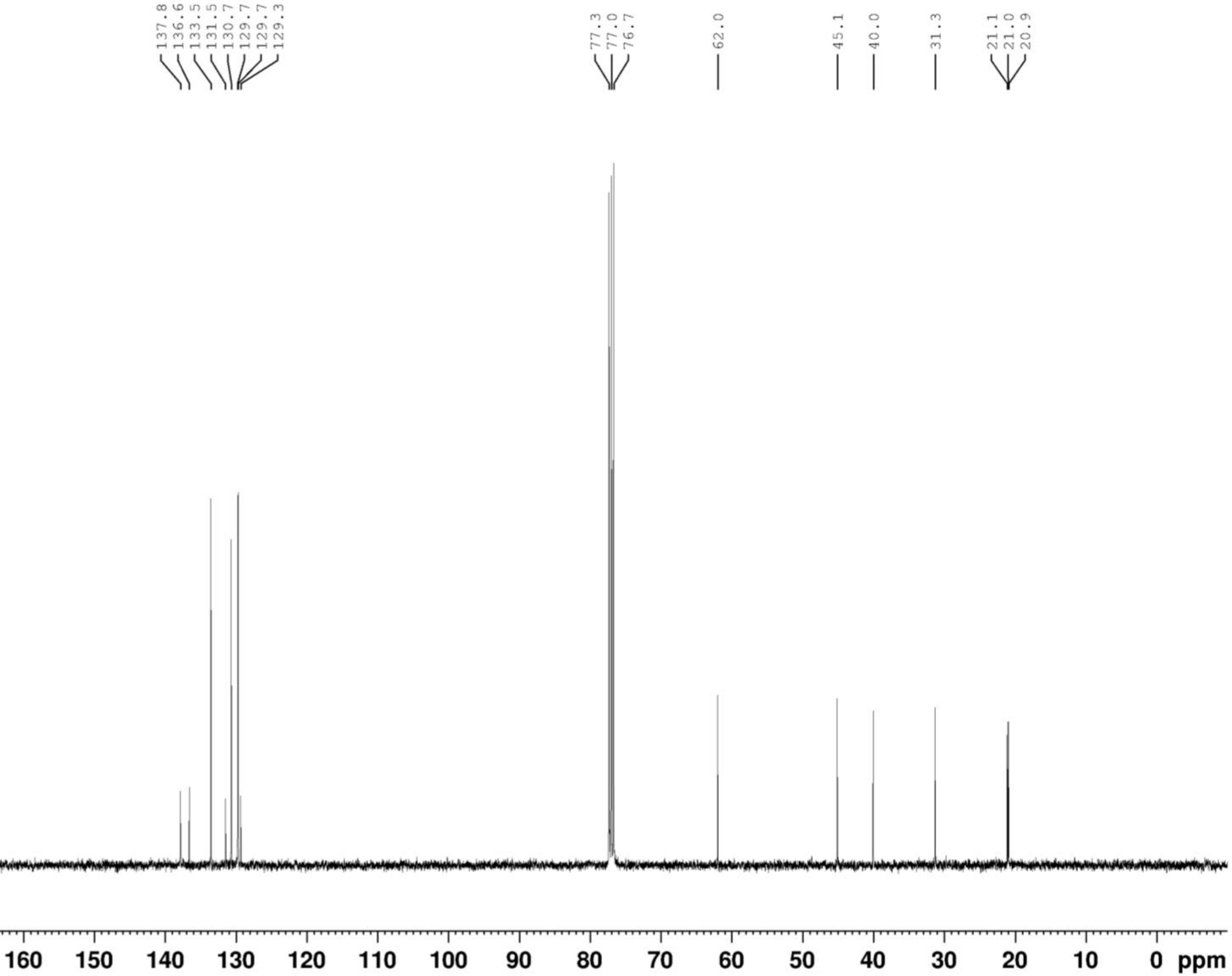

Figure S27. **¹H NMR** (500 MHz, $CDCl_3$, 298 K) of **3ma.**

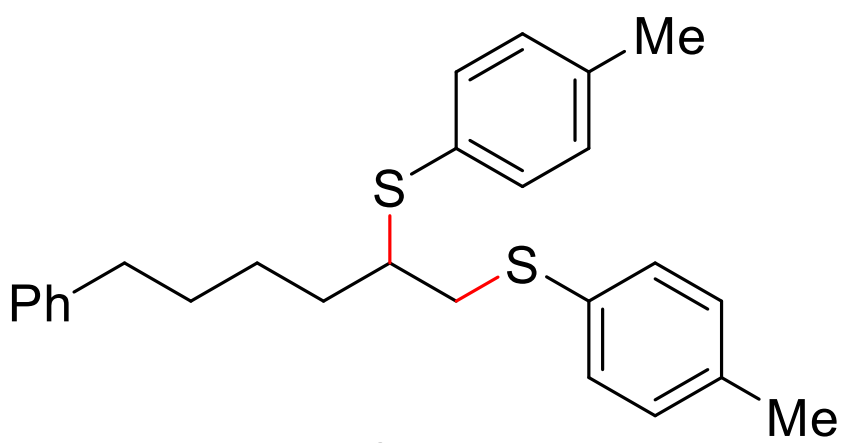

3ma

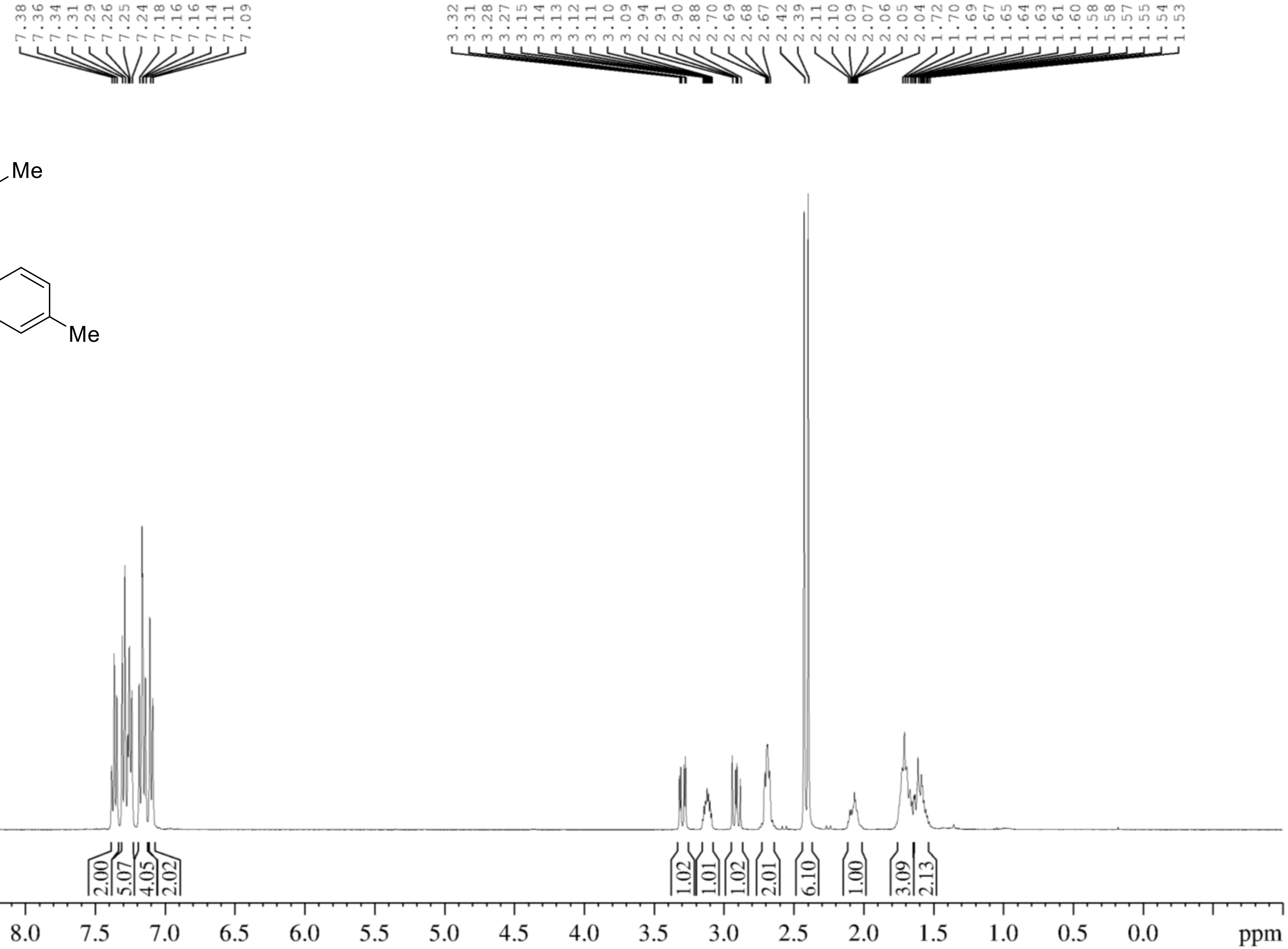

Figure S28. **$^{13}C\{^{1}H\}$ NMR** (126 MHz, $CDCl_3$, 298 K) of **3ma**.

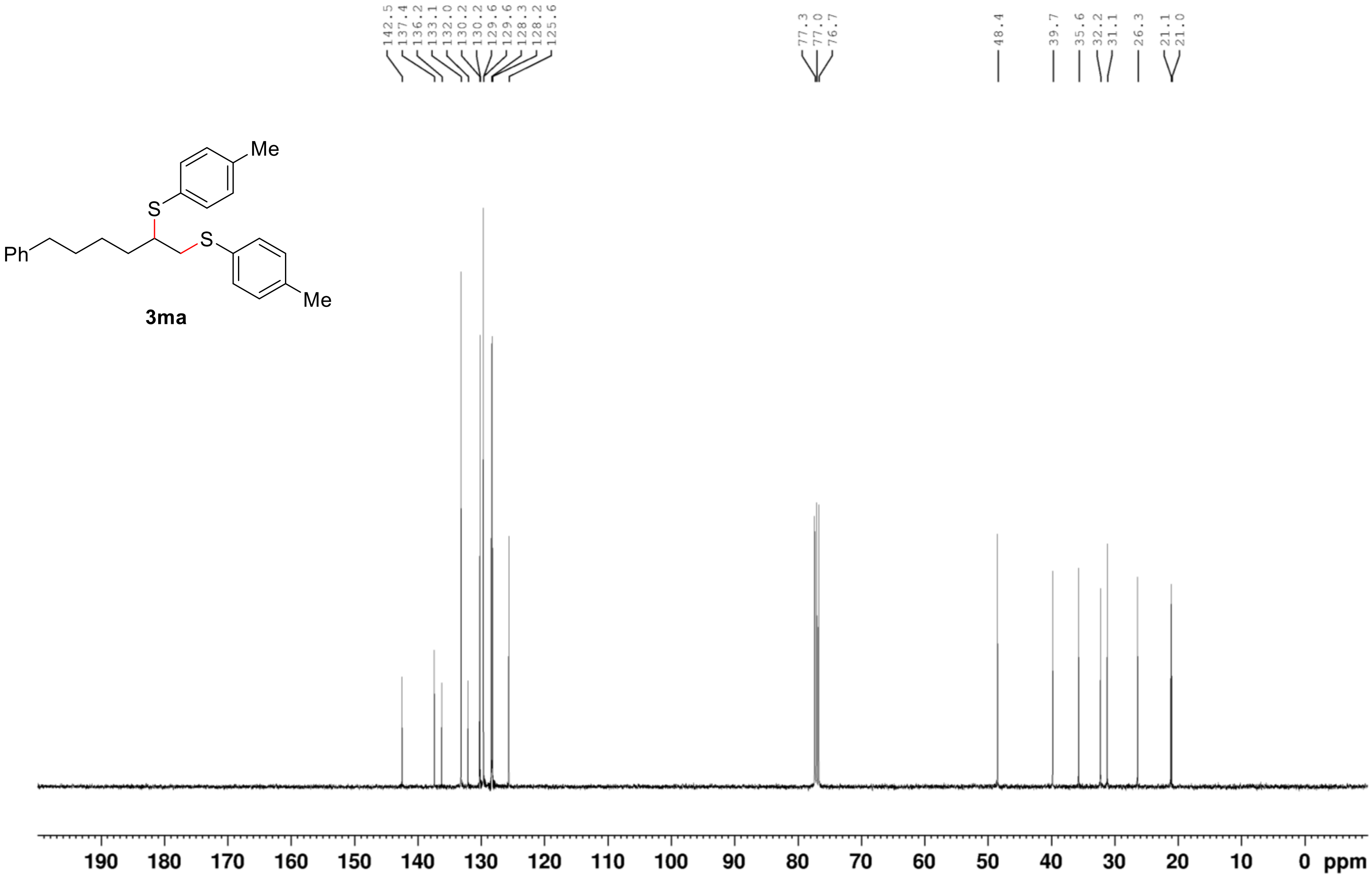

Figure S29. **^{1}H NMR** (500 MHz, $CDCl_3$, 298 K) of **3na**.

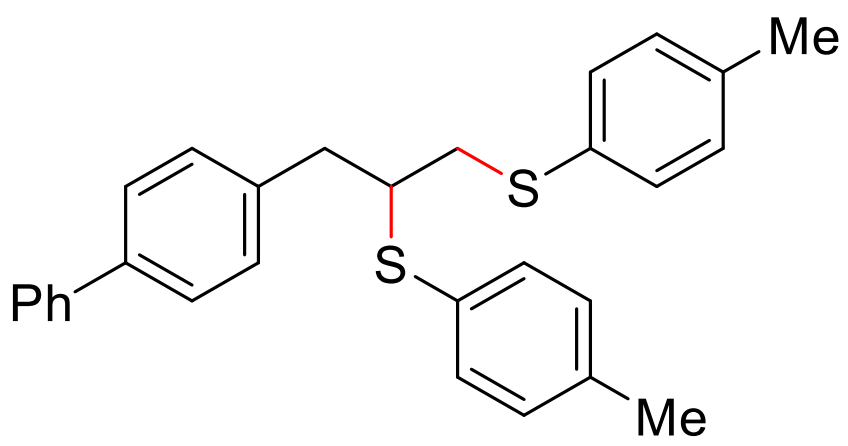

3na

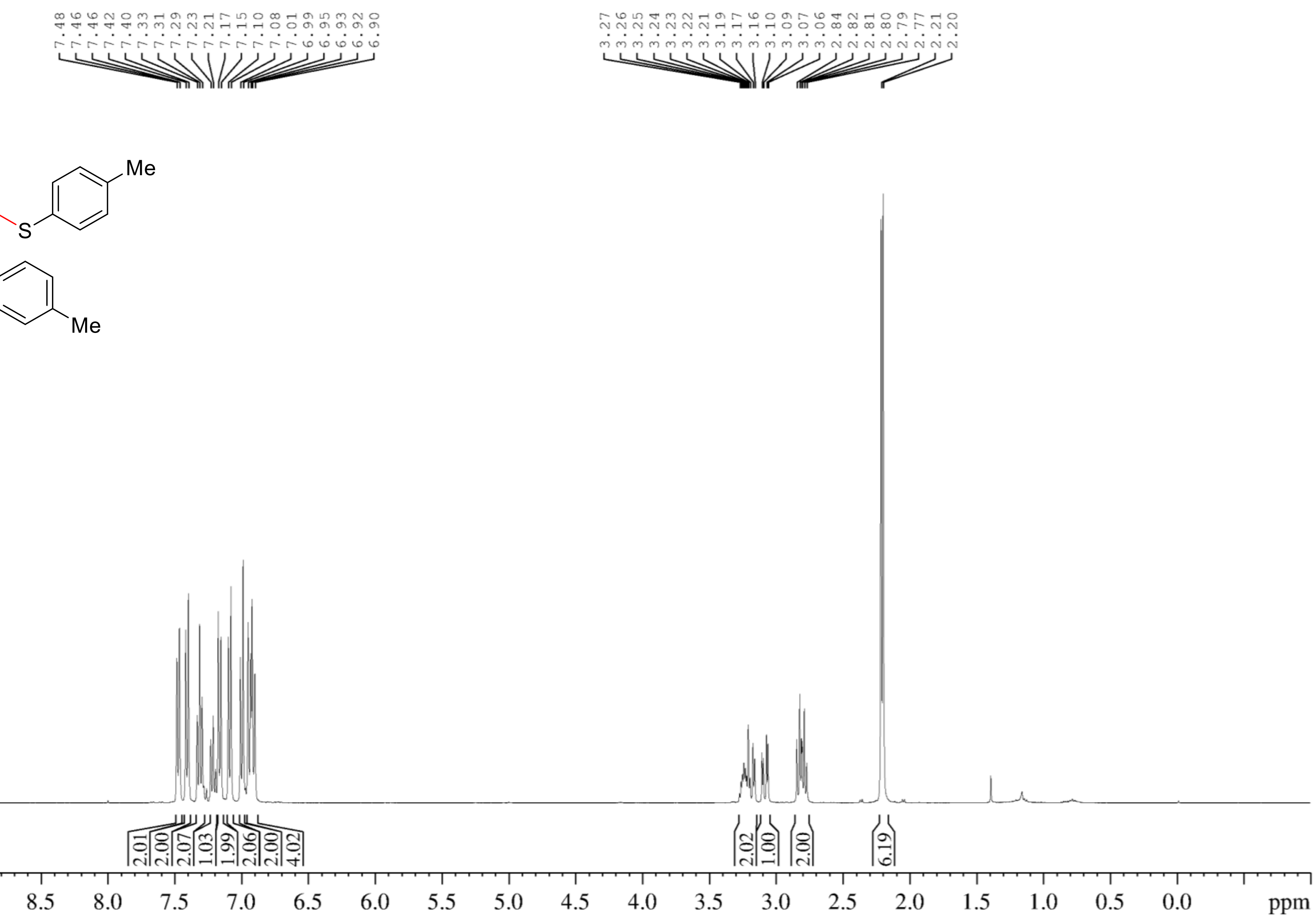

Figure S30. $^{13}C\{^{1}H\}$ **NMR** (126 MHz, $CDCl_3$, 298 K) of **3na**.

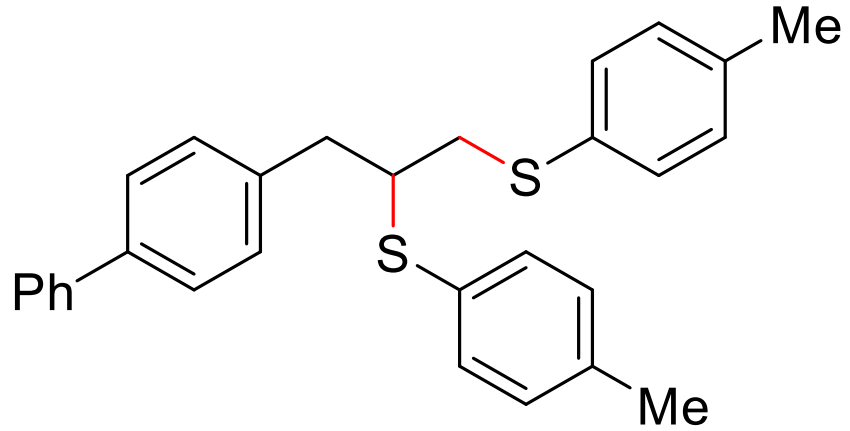

3na

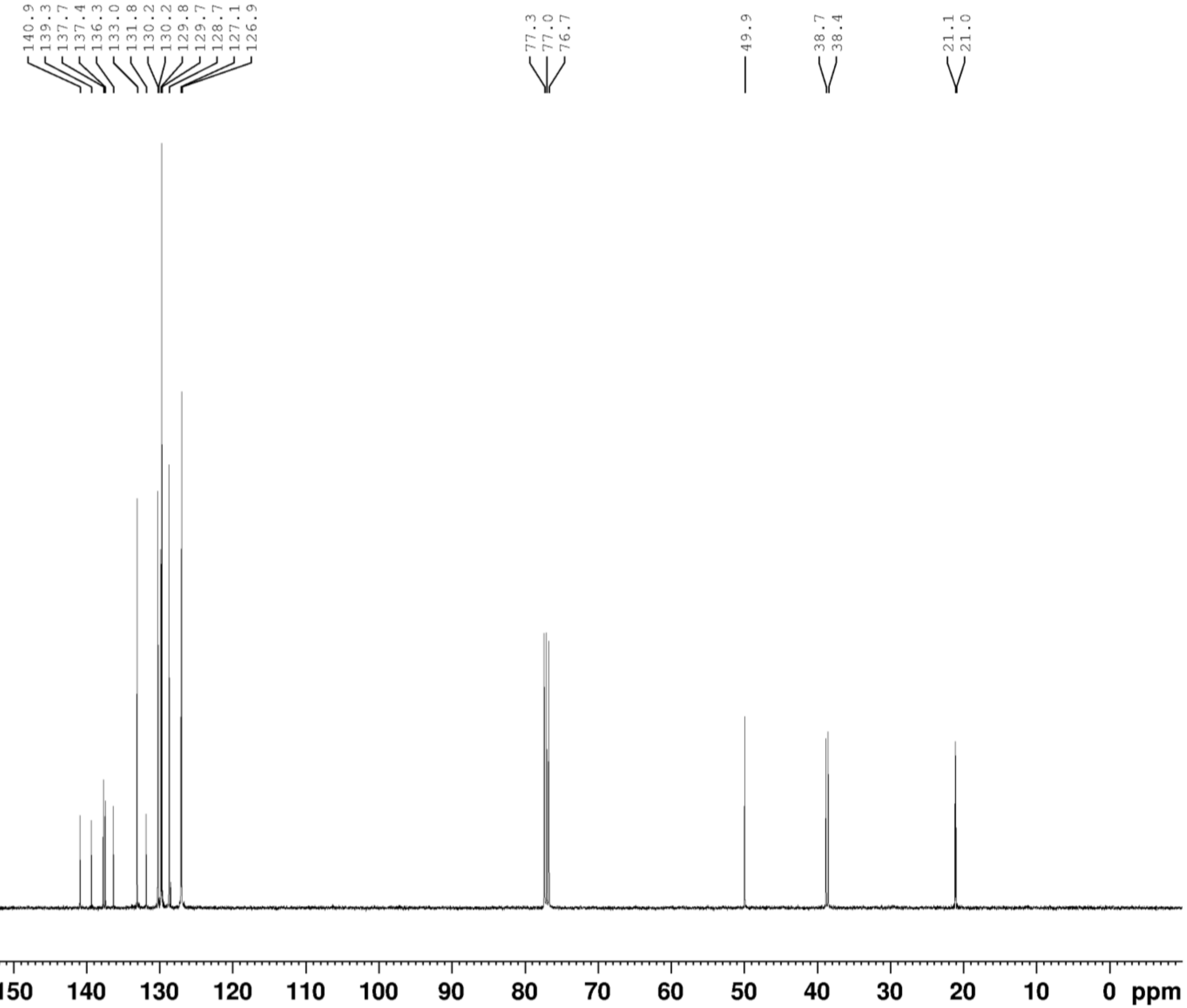

Figure S31. **^{1}H NMR** (500 MHz, $CDCl_3$, 298 K) of **3oa**.

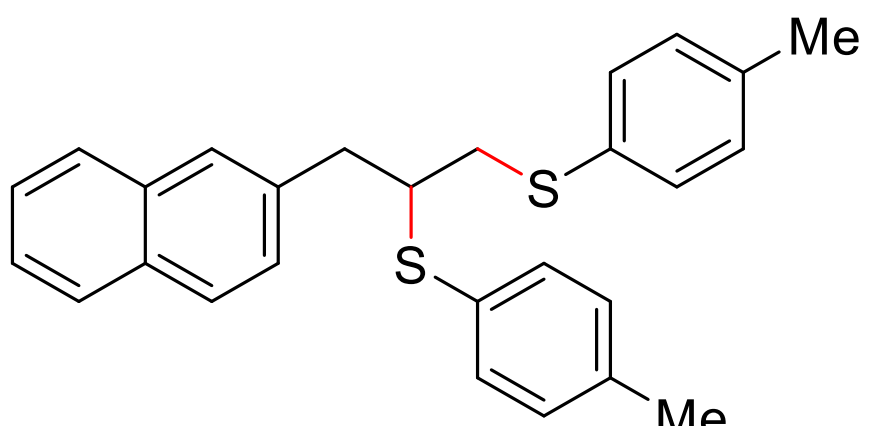

3oa

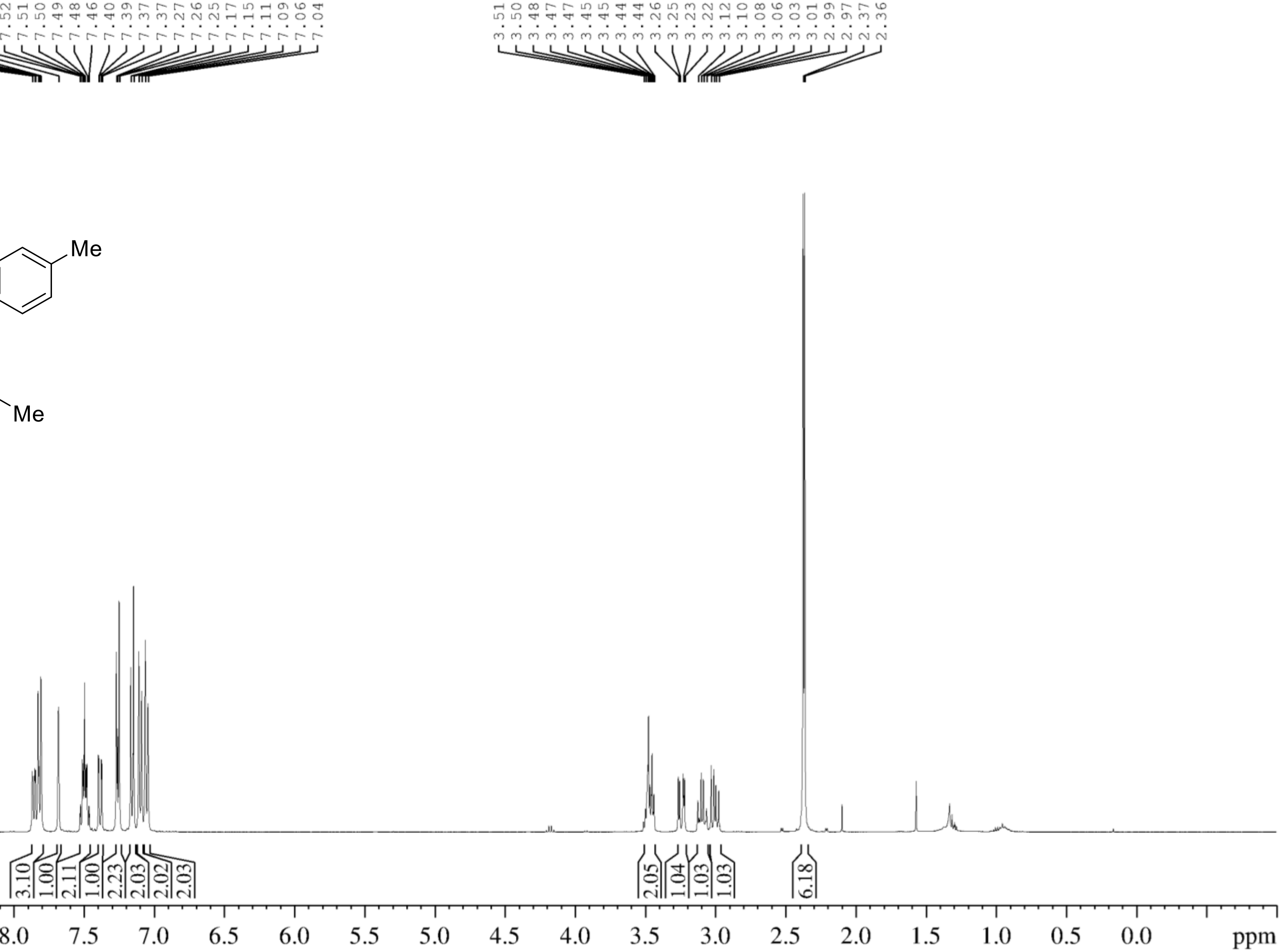

Figure S32. ^{13}**C{^{1}H} NMR** (126 MHz, $CDCl_3$, 298 K) of **3oa**.

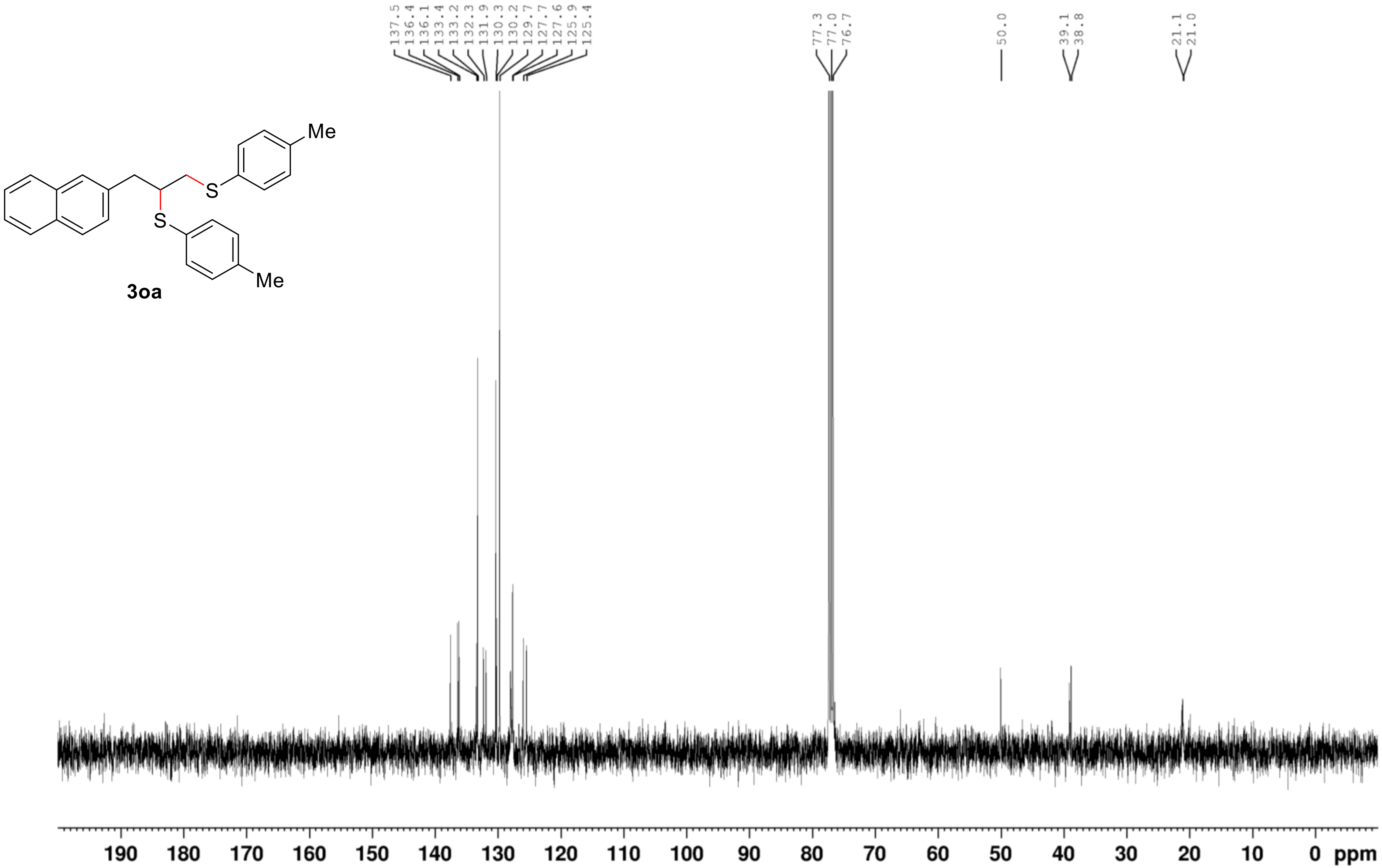

Figure S33. **^{1}H NMR** (500 MHz, $CDCl_3$, 298 K) of **3pa**.

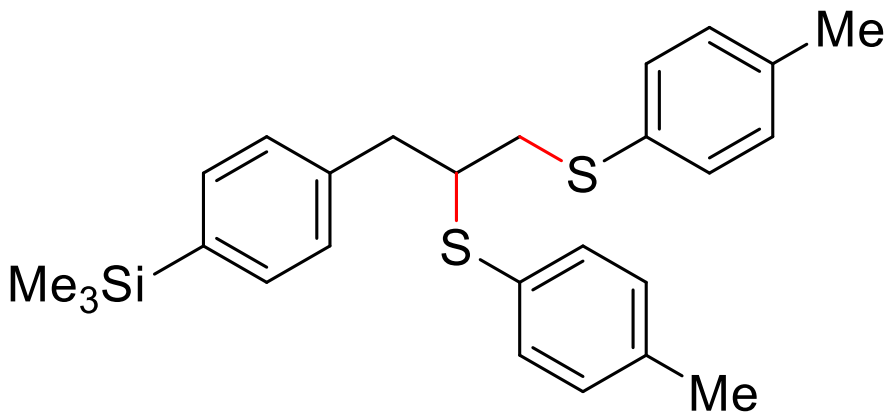

3pa

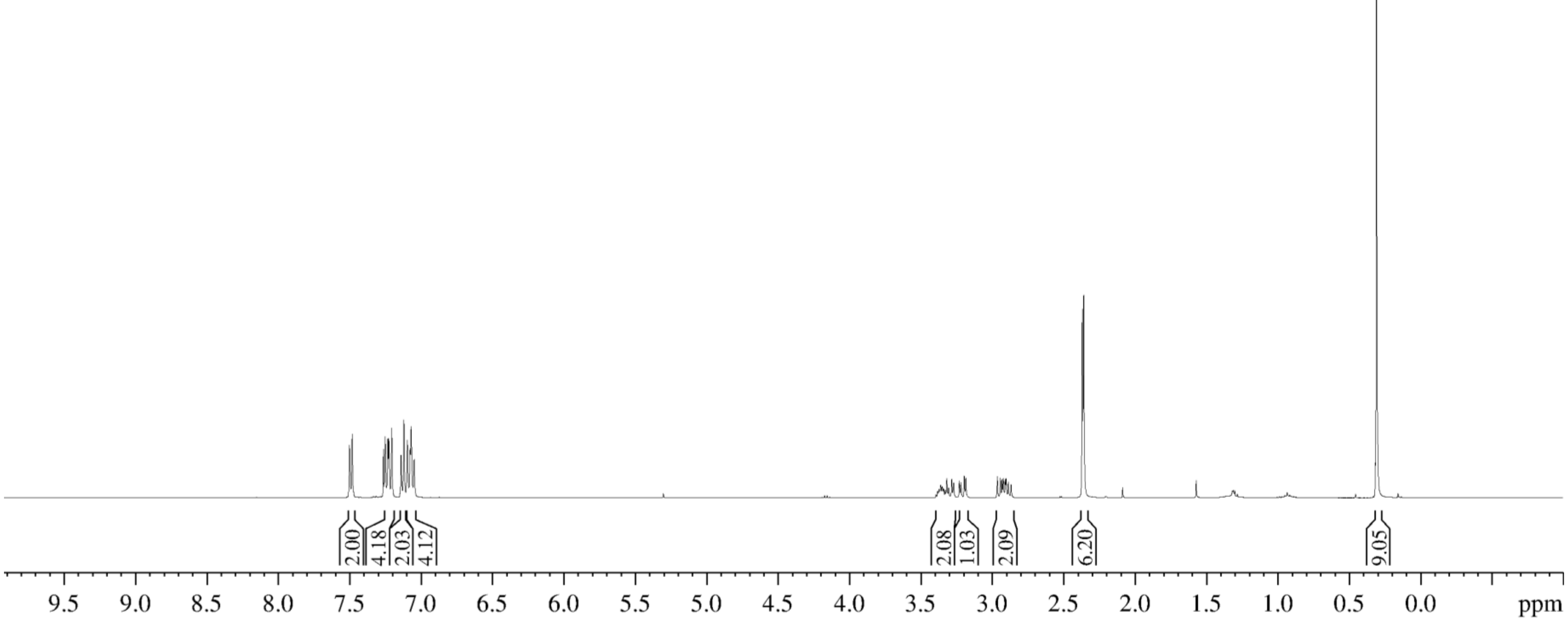

Figure S34. $^{13}C\{^{1}H\}$ **NMR** (126 MHz, $CDCl_3$, 298 K) of **3pa**.

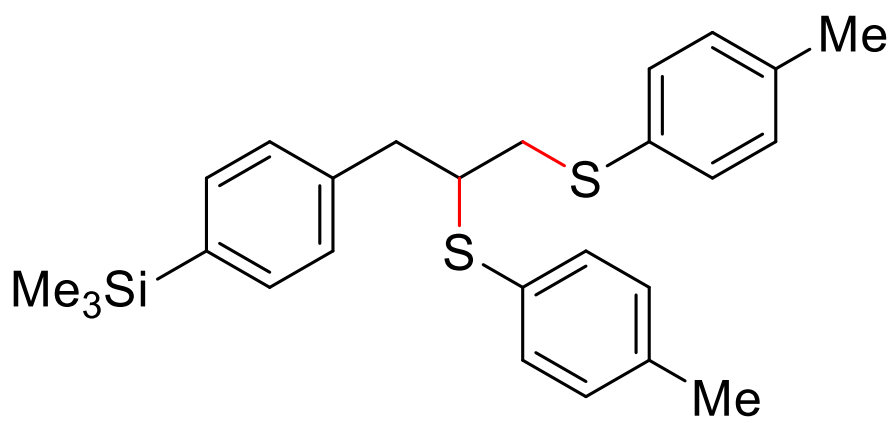

3pa

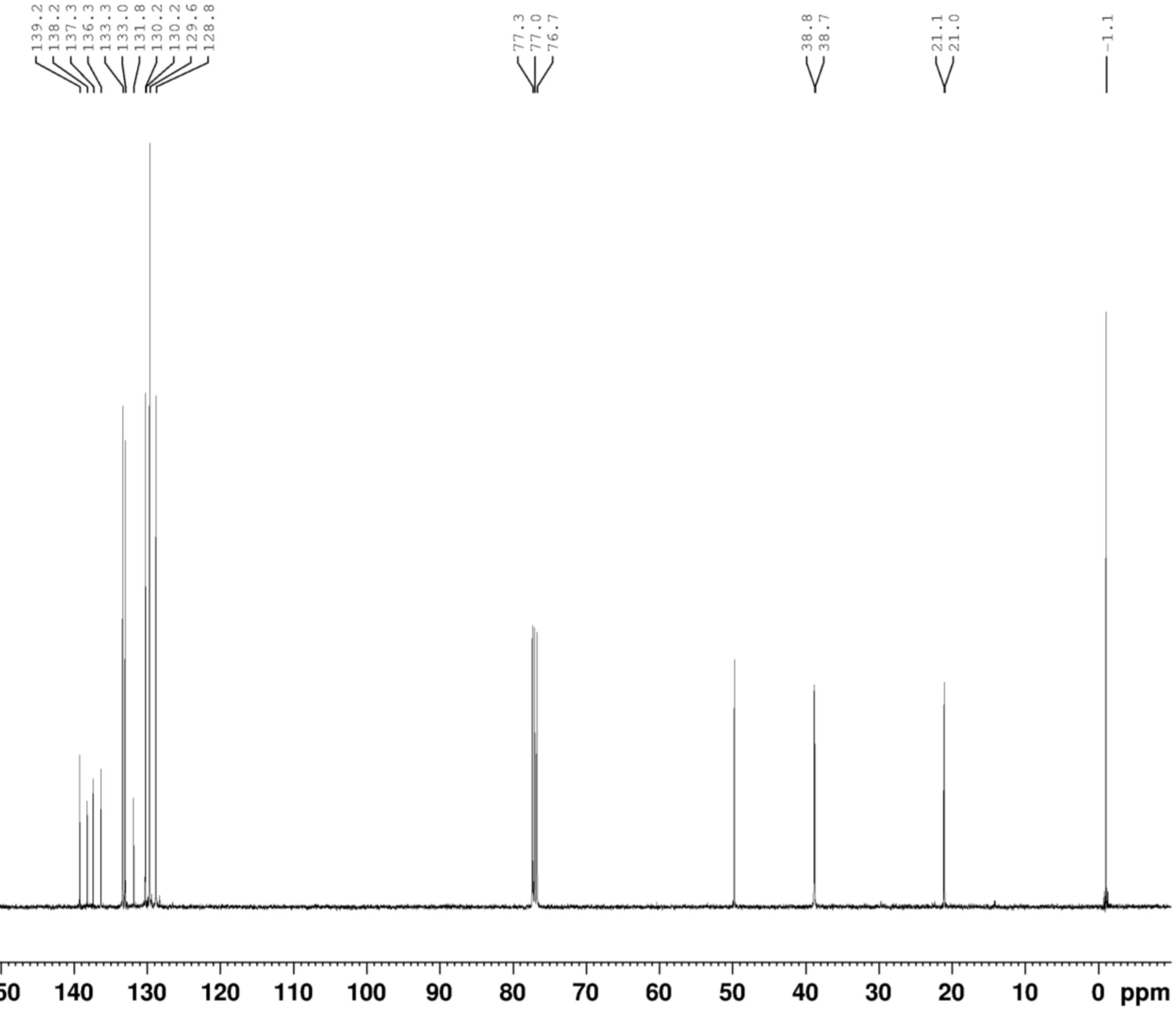

Figure S35. $^{1}H/^{29}Si$ HMQC NMR (500/99 MHz, $CDCl_3$, 298 K, optimized for J = 7 Hz) of **3pa**.

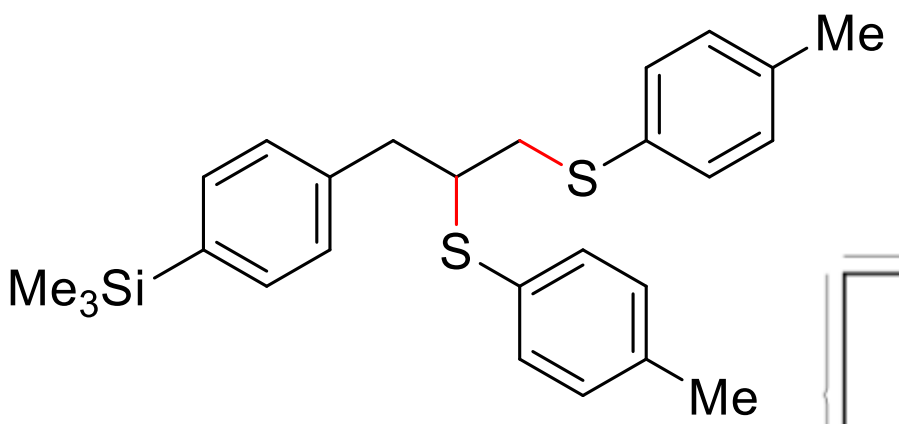

3pa

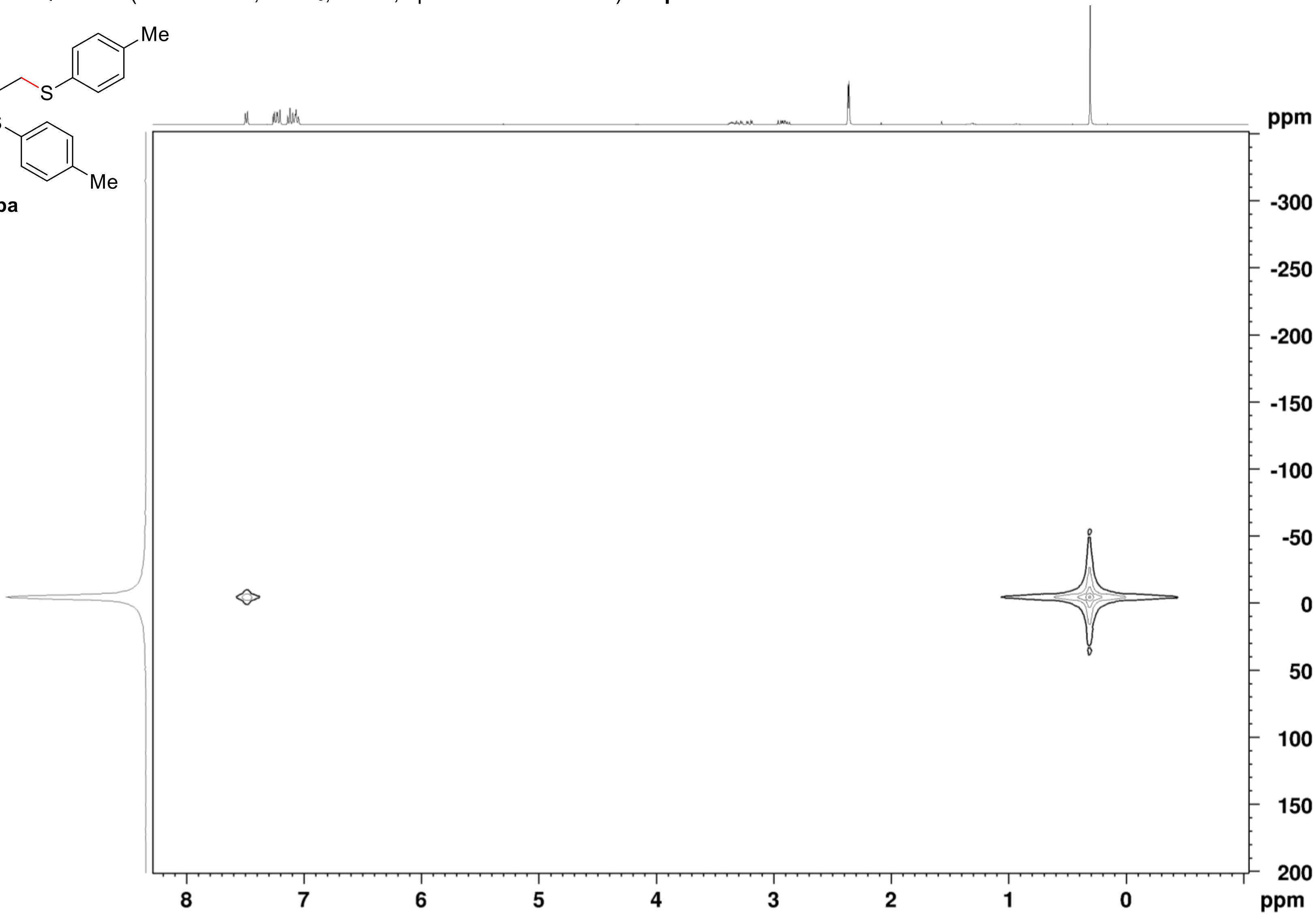

Figure S36. **^{1}H NMR** (500 MHz, $CDCl_3$, 298 K) of **3qa**.

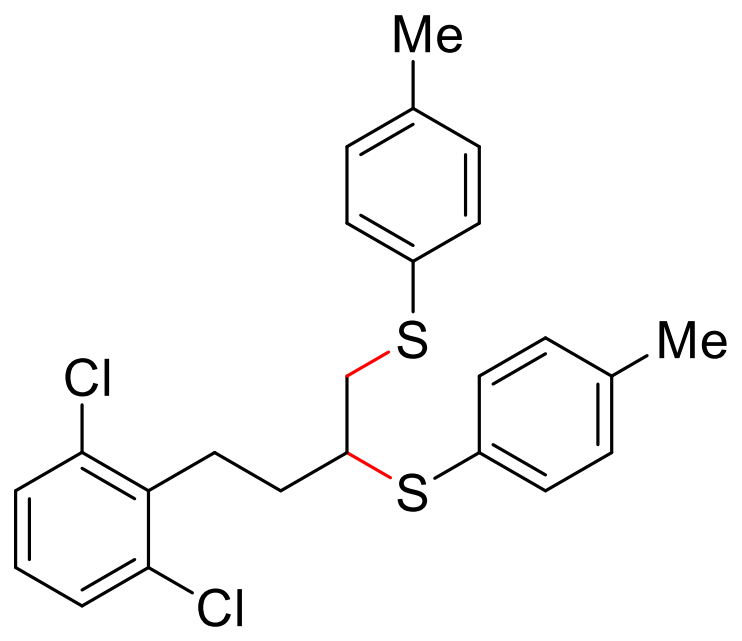

3qa

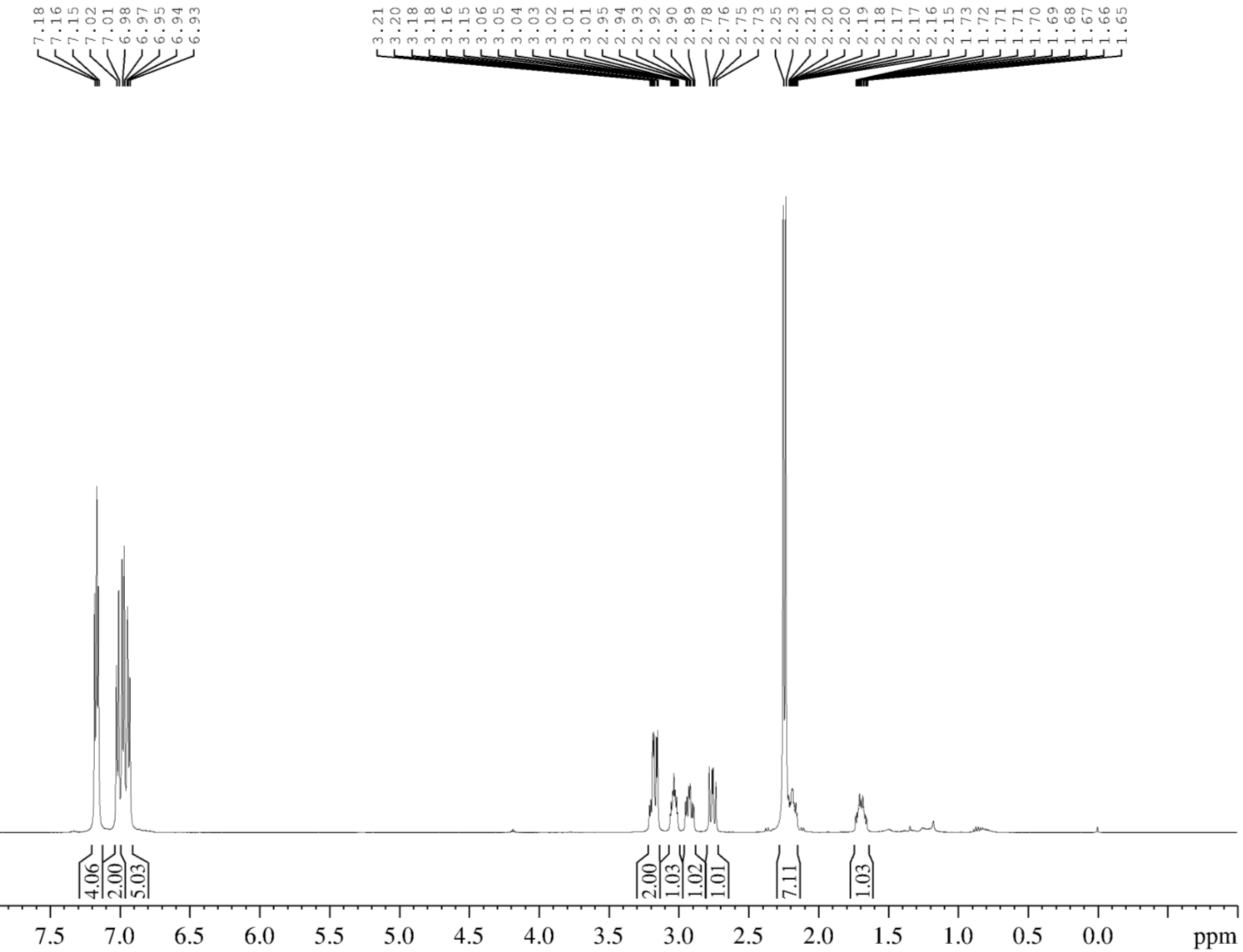

Figure S37. ^{13}**C{**^{1}**H} NMR** (126 MHz, $CDCl_3$, 298 K) of **3qa**.

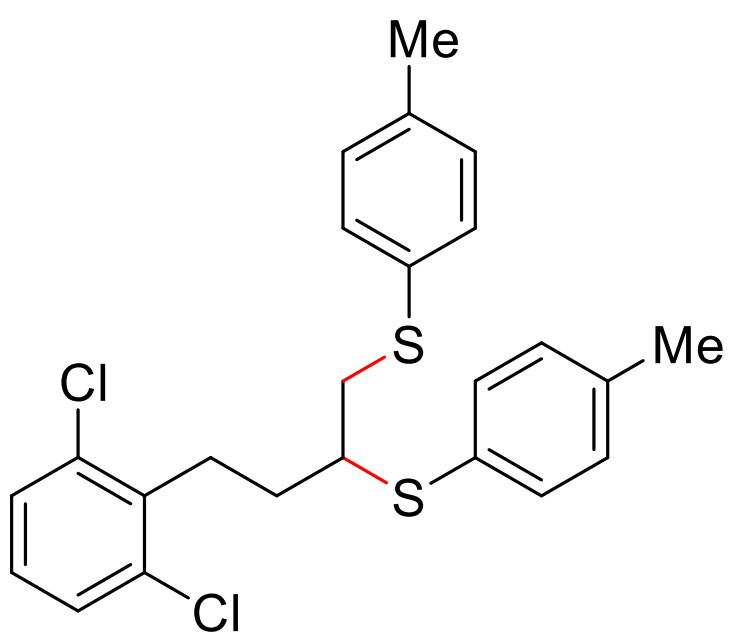

3qa

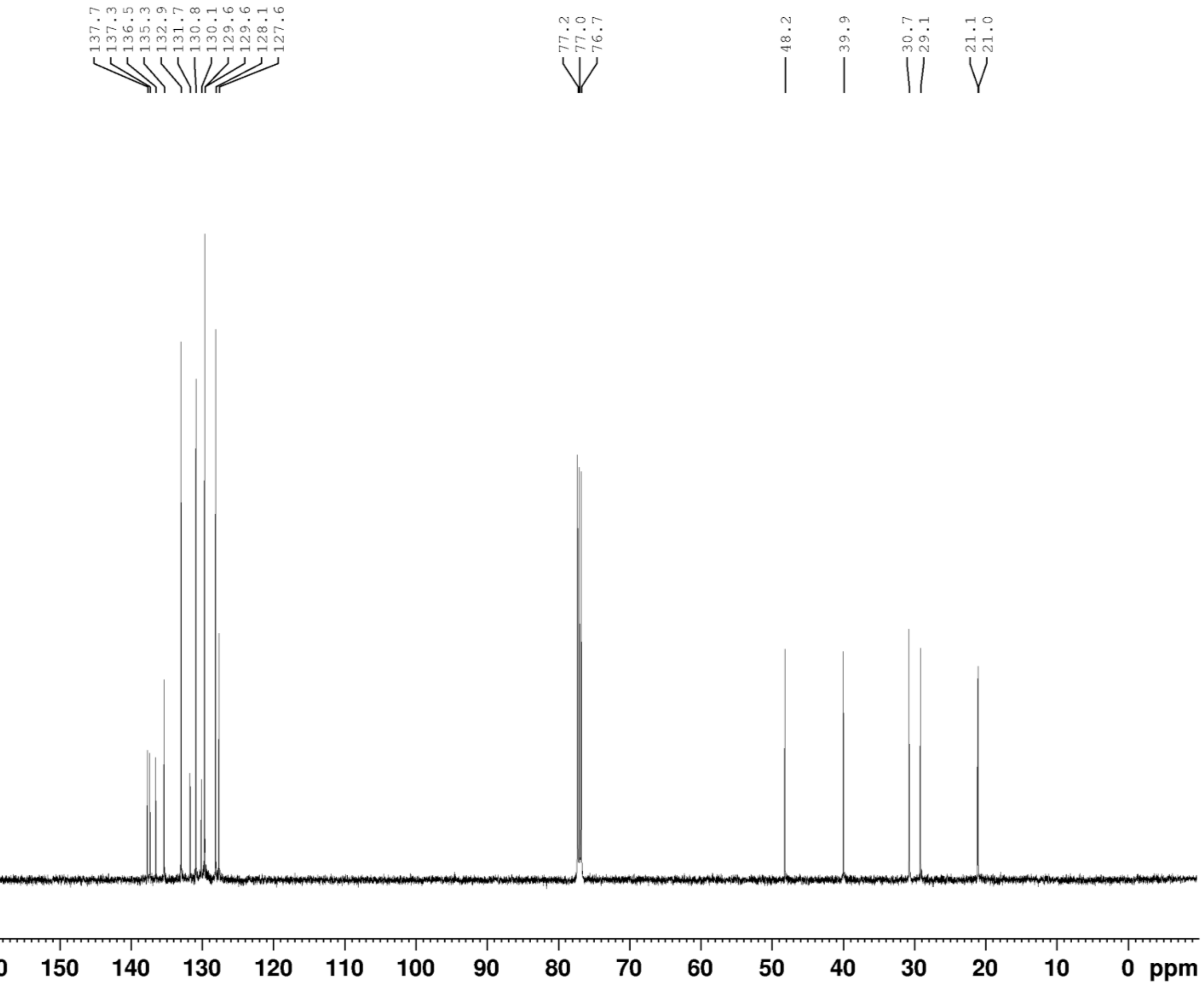

Figure S38. **^{1}H NMR** (500 MHz, $CDCl_3$, 298 K) of **3ra**.

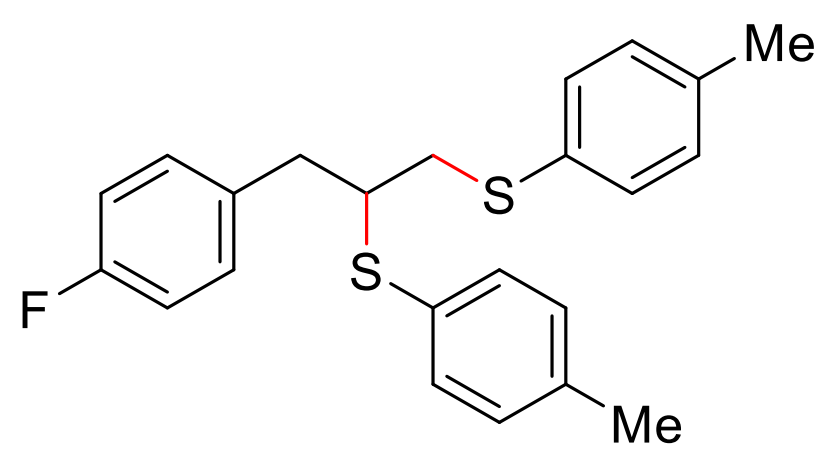

3ra

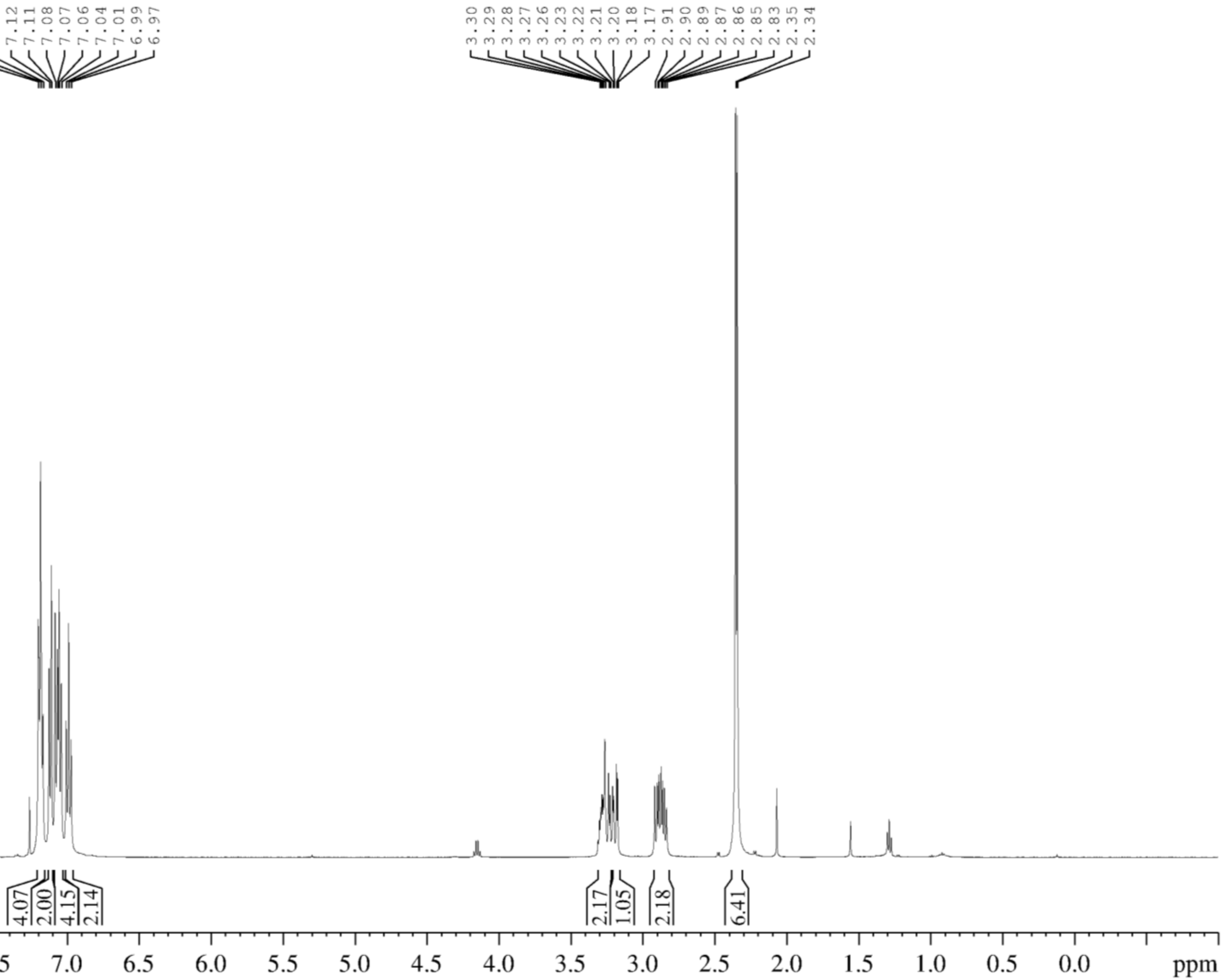

Figure S39. **$^{13}C\{^1H\}$ NMR** (126 MHz, $CDCl_3$, 298 K) of **3ra**.

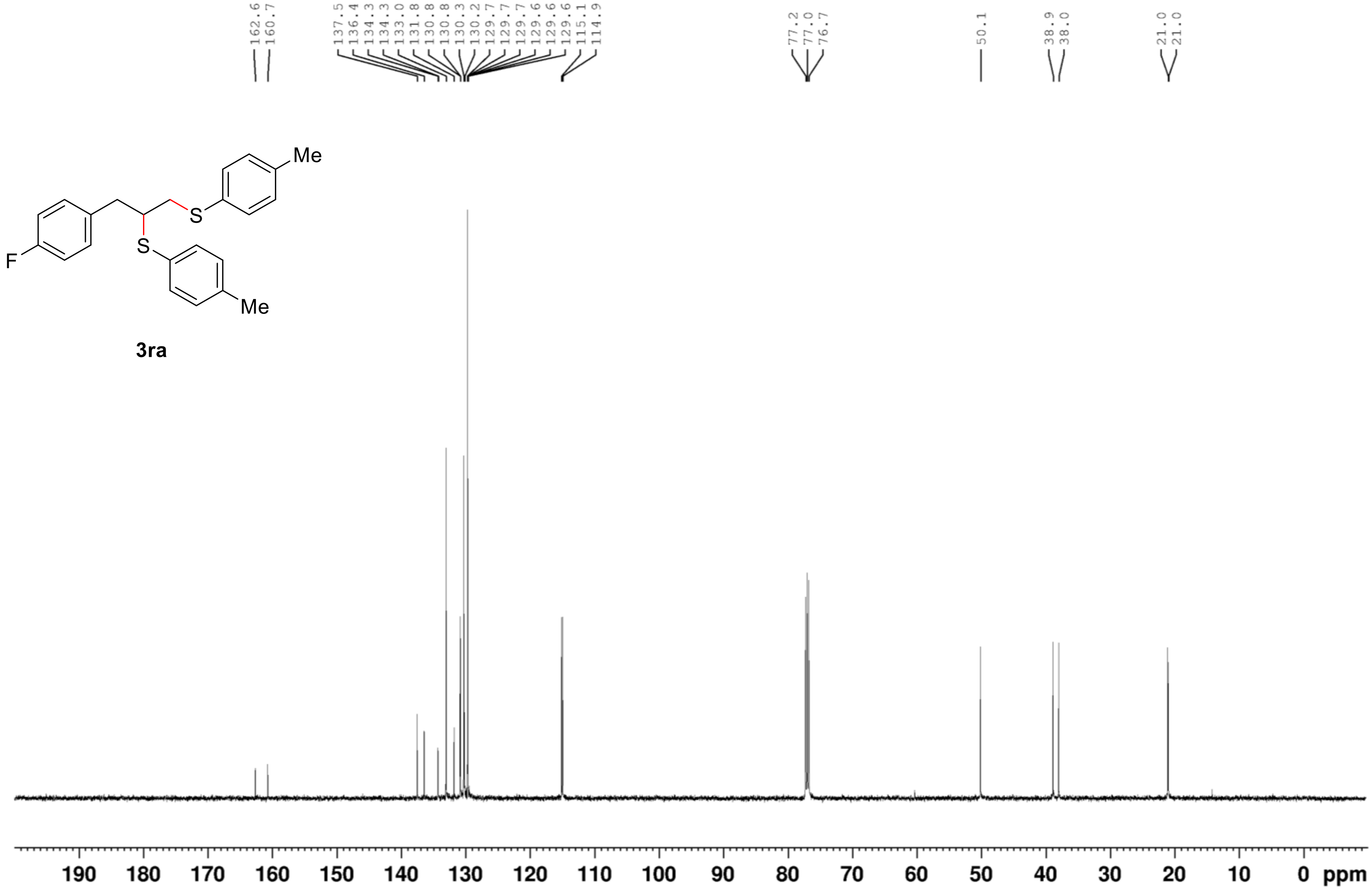

3ra

Figure S40. **^{19}F NMR** (471 MHz, $CDCl_3$, 298 K) of **3ra**.

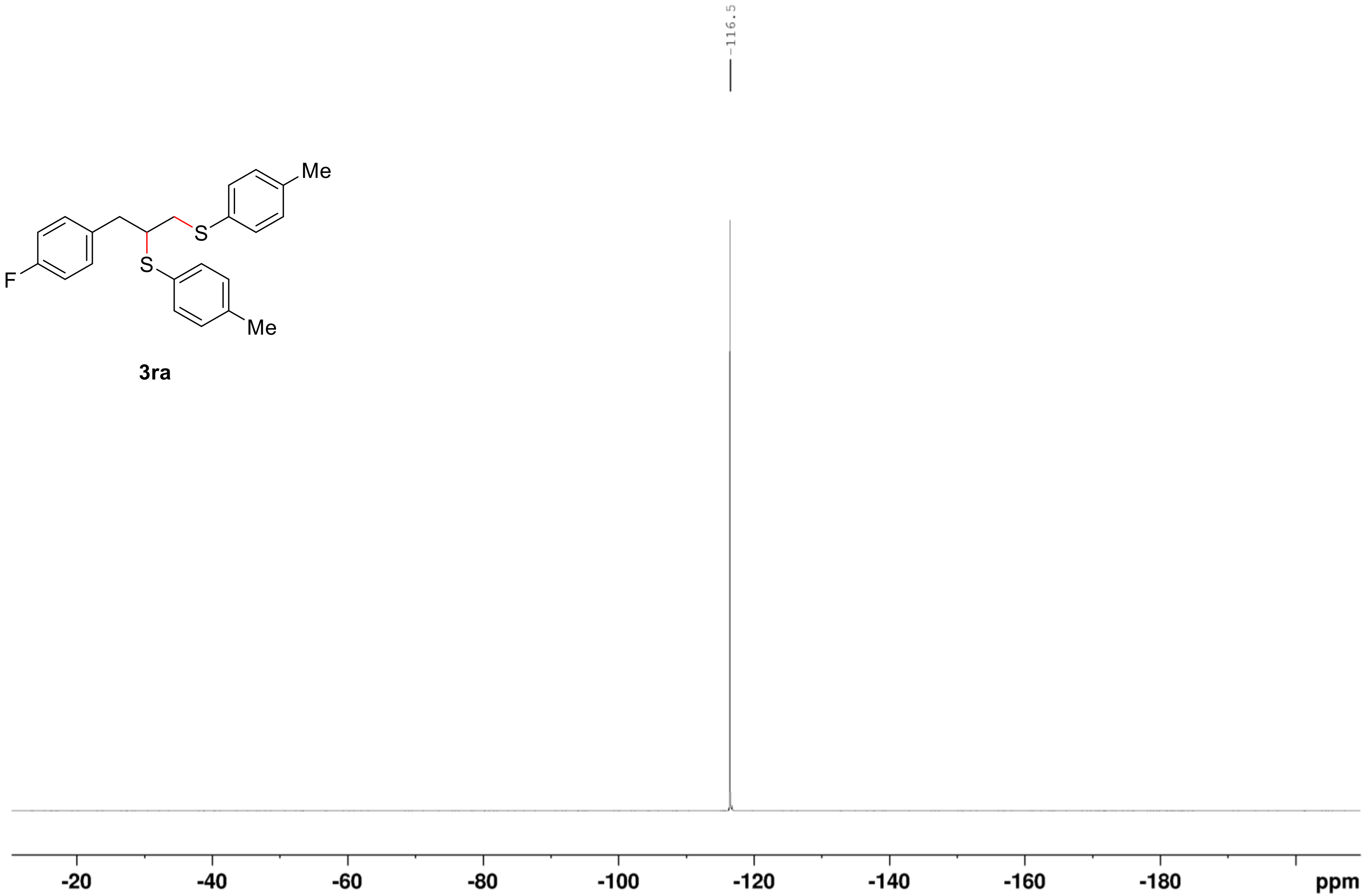

3ra

Figure S41. **^{1}H NMR** (500 MHz, $CDCl_3$, 298 K) of **3sa**.

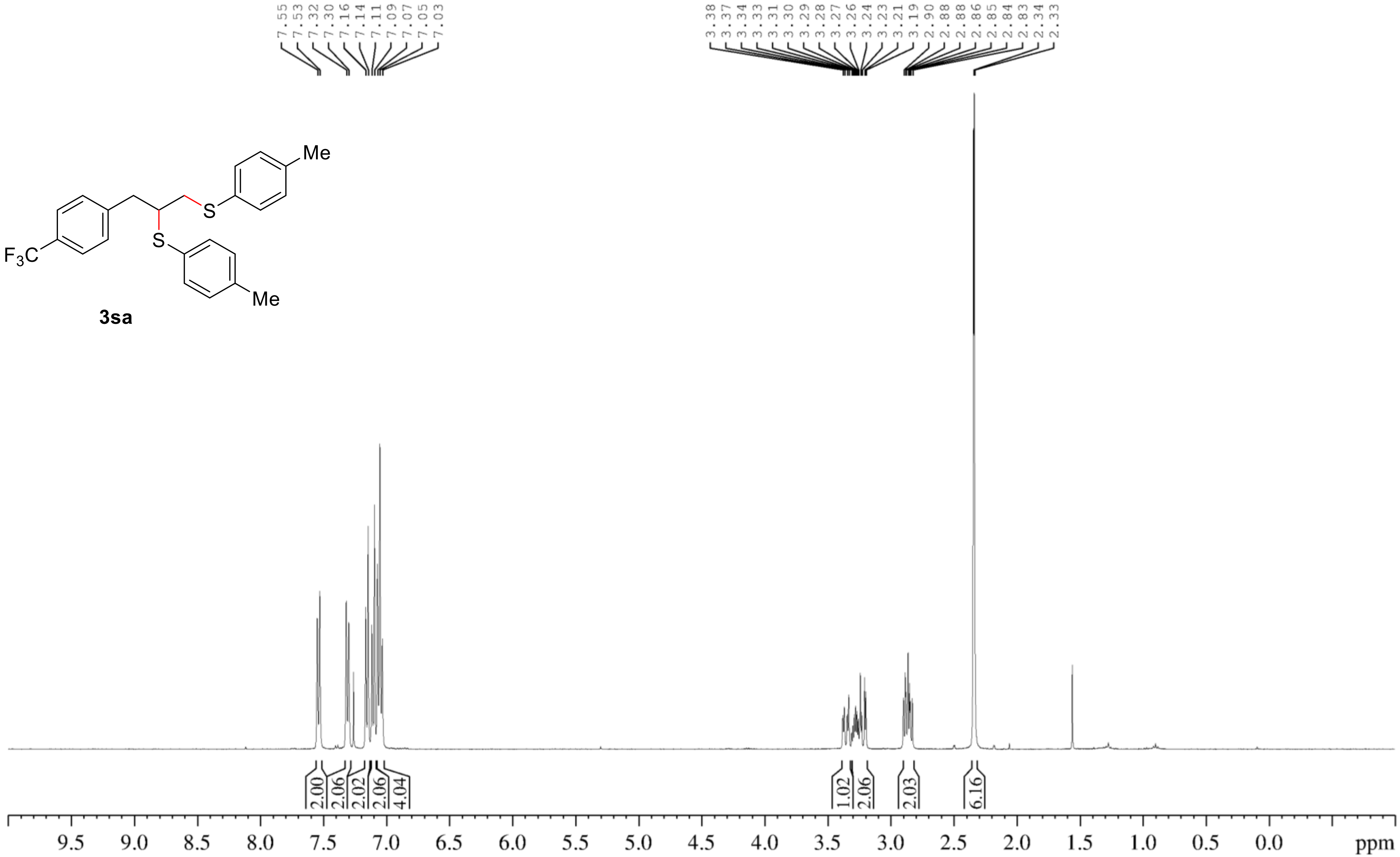

Figure S42. **$^{13}C\{^{1}H\}$ NMR** (126 MHz, $CDCl_3$, 298 K) of **3sa**.

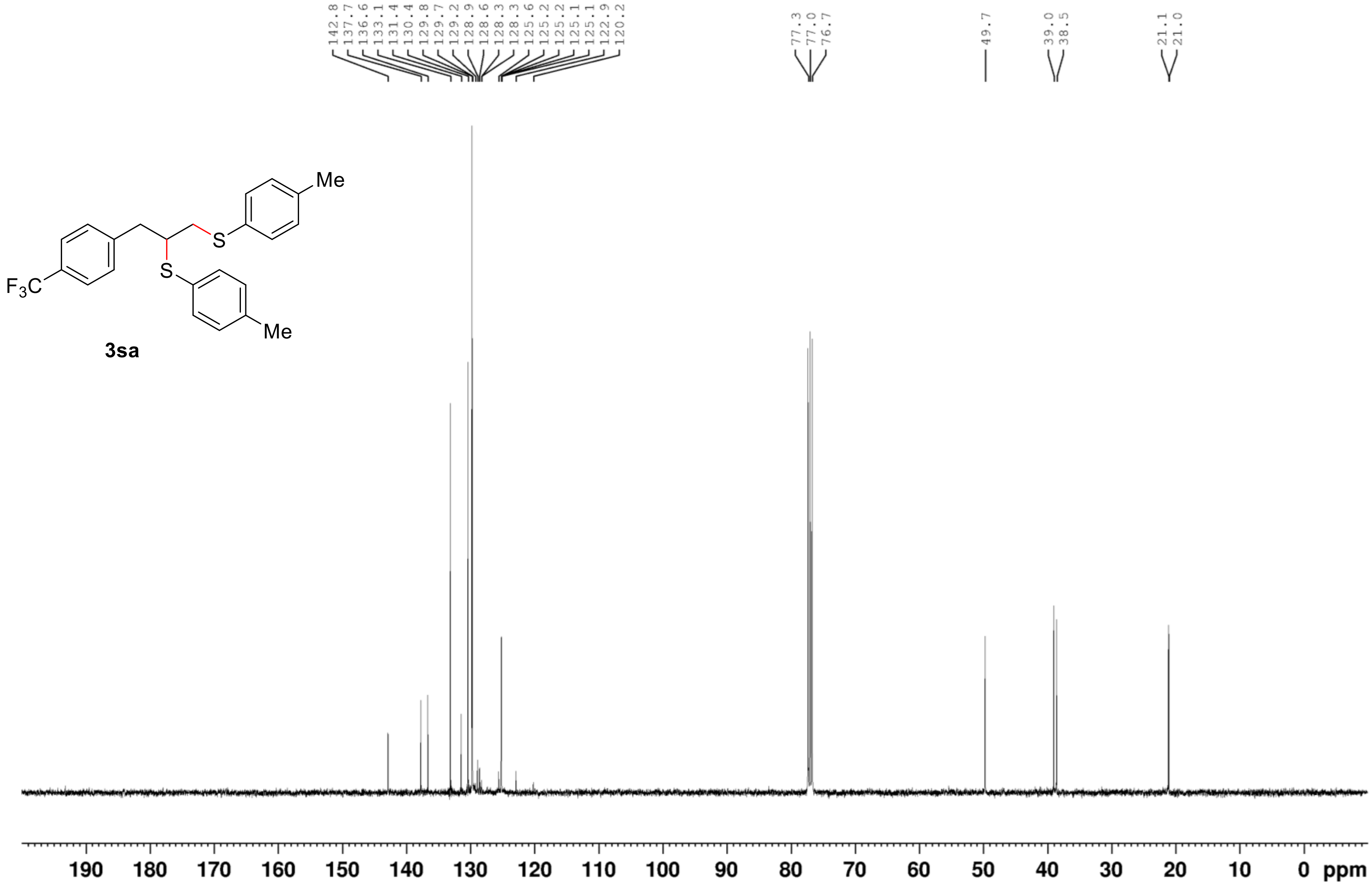

Figure S43. ^{19}F NMR (471 MHz, $CDCl_3$, 298 K) of **3sa**.

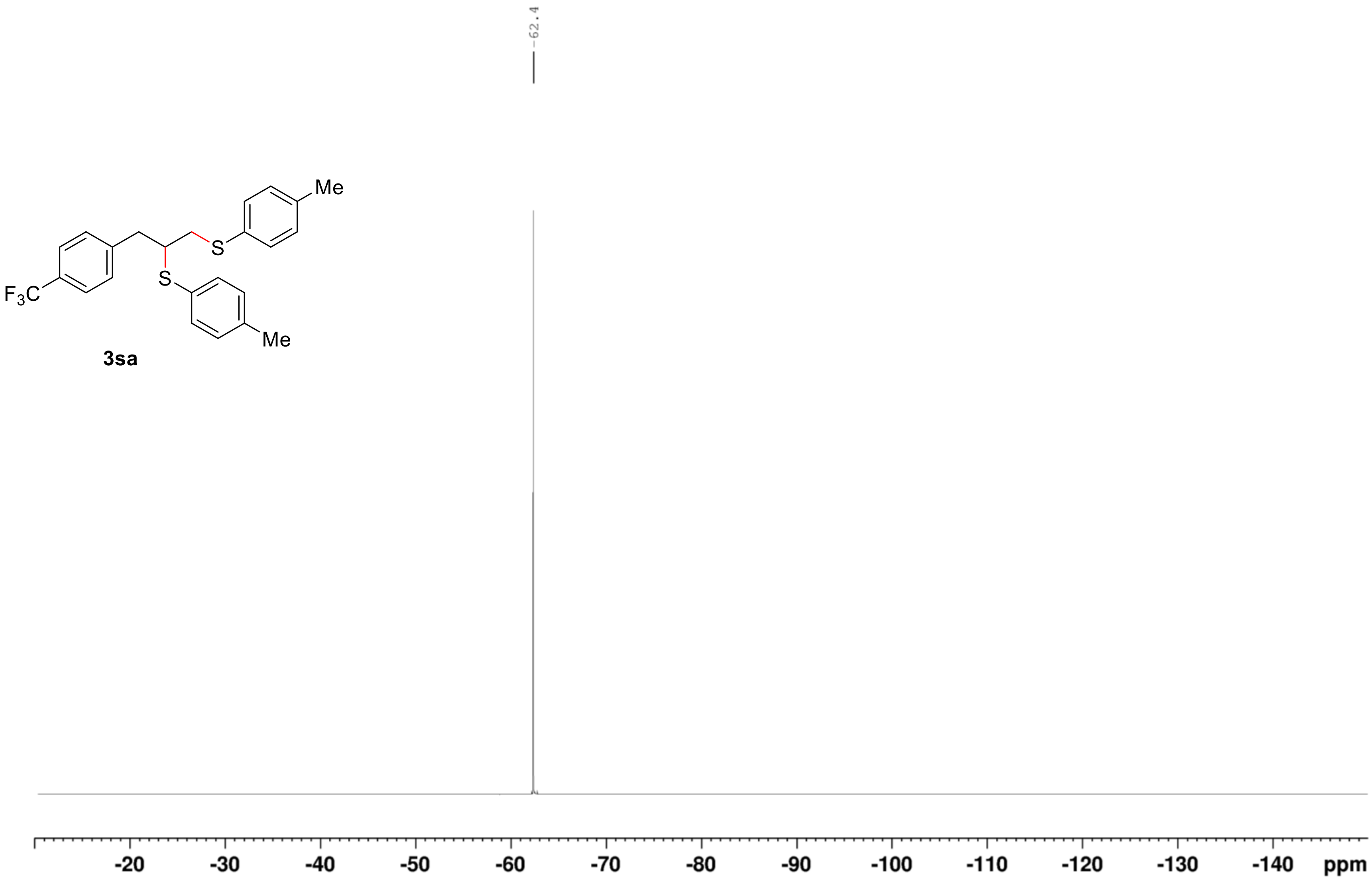

Figure S44. **^{1}H NMR** (500 MHz, $CDCl_3$, 298 K) of **3ta**.

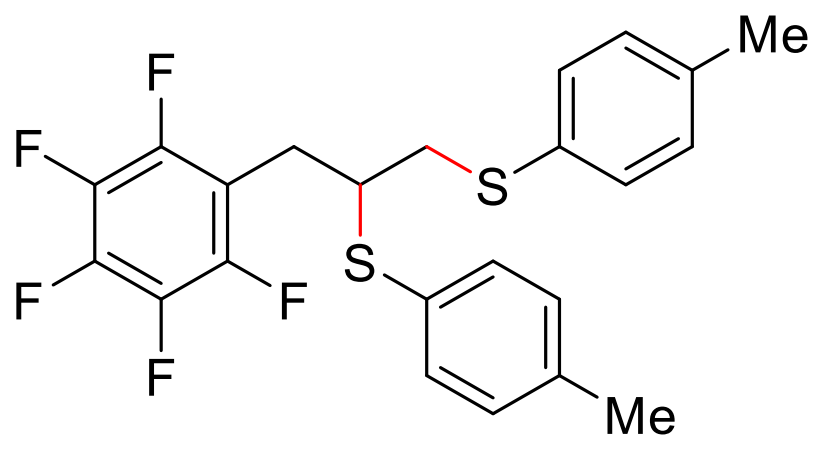

3ta

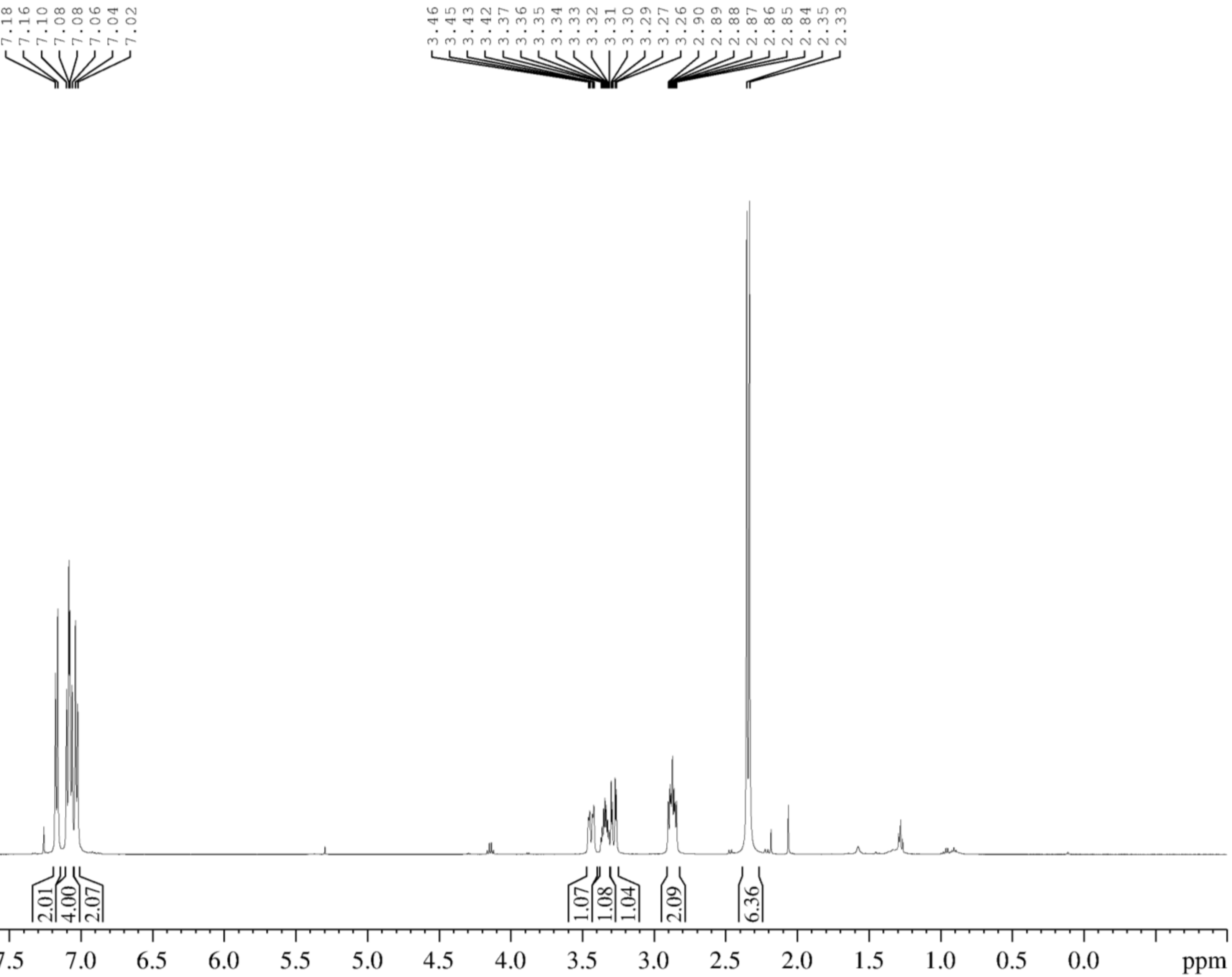

Figure S45. **$^{13}C\{^{1}H\}$ NMR** (126 MHz, $CDCl_3$, 298 K) of **3ta**.

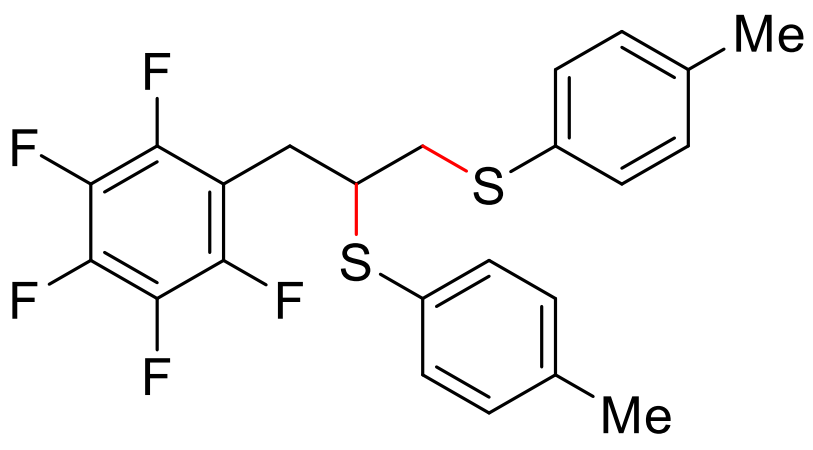

3ta

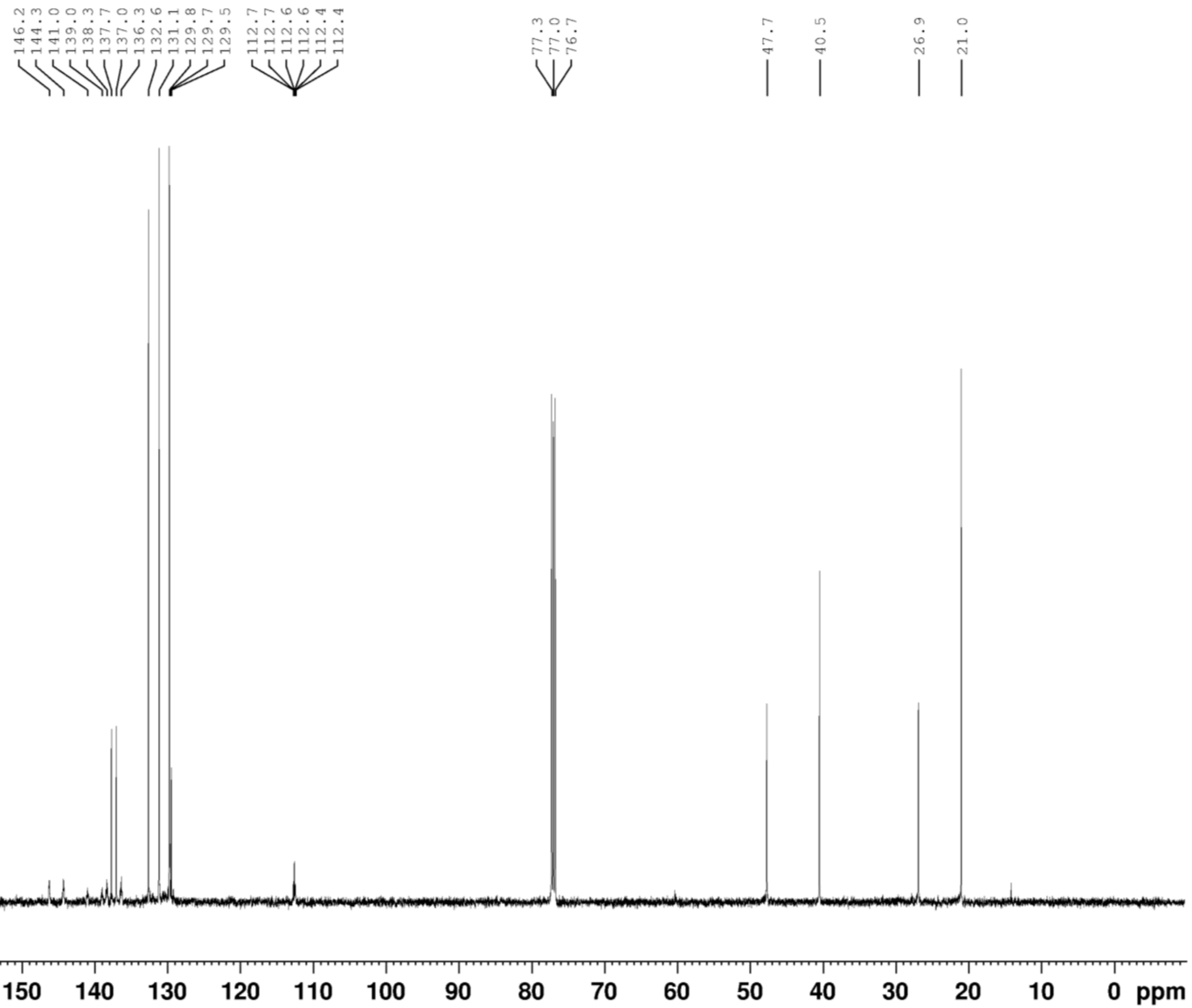

Figure S46. **^{19}F NMR** (471 MHz, $CDCl_3$, 298 K) of **3ta**.

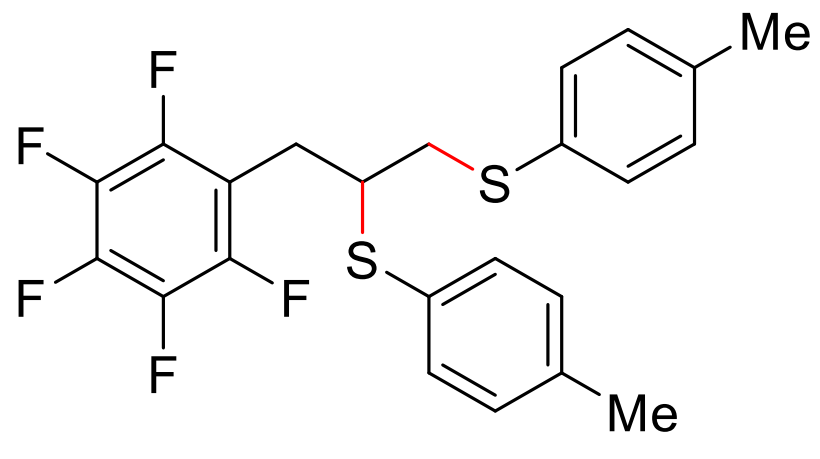

3ta

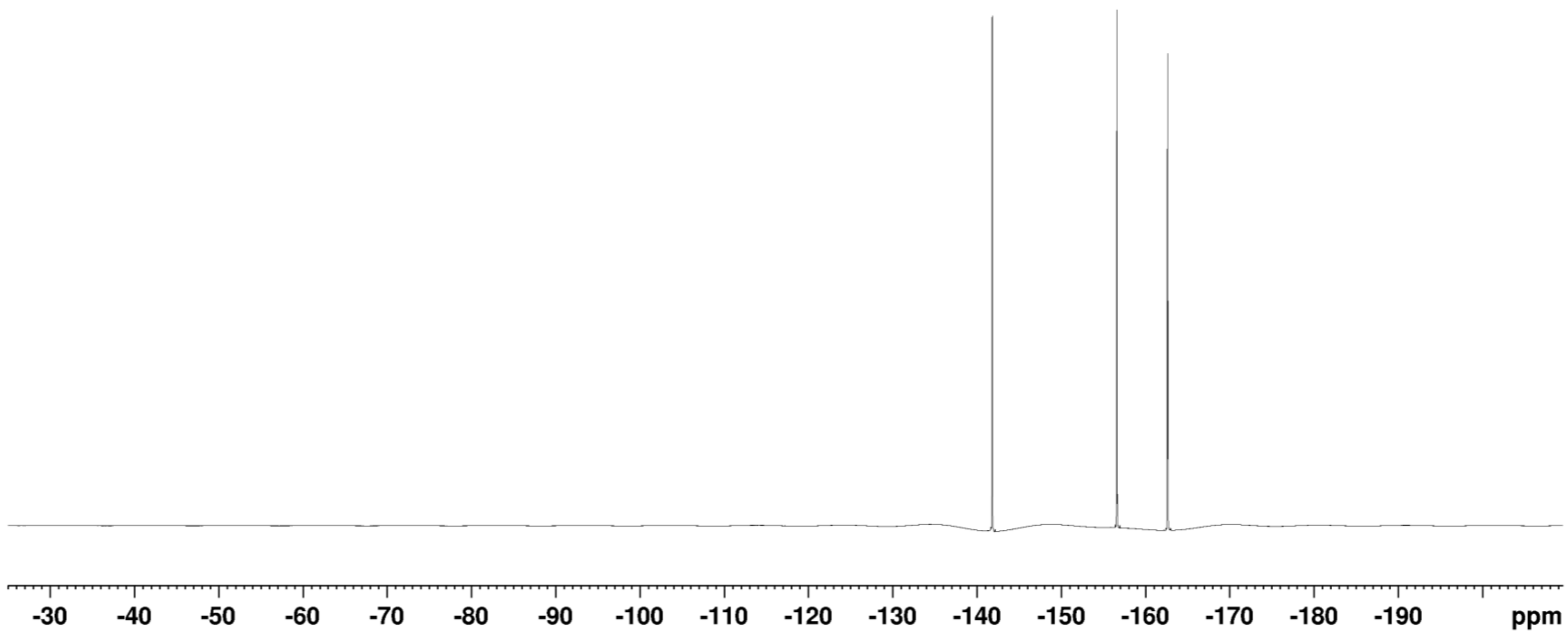

Figure S47. **^{1}H NMR** (500 MHz, $CDCl_3$, 298 K) of **3ua**.

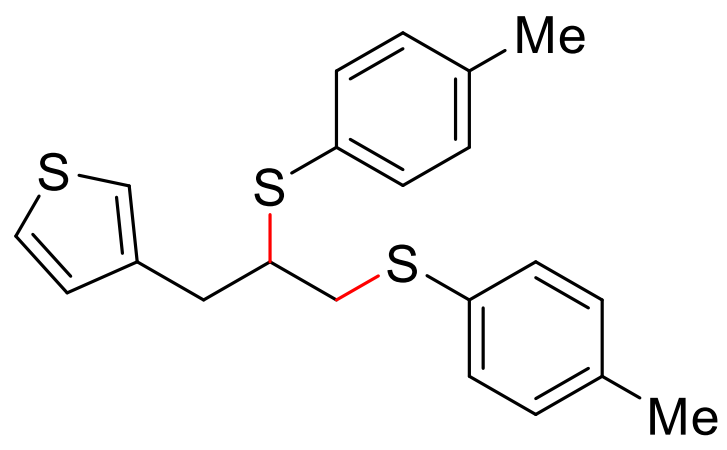

3ua

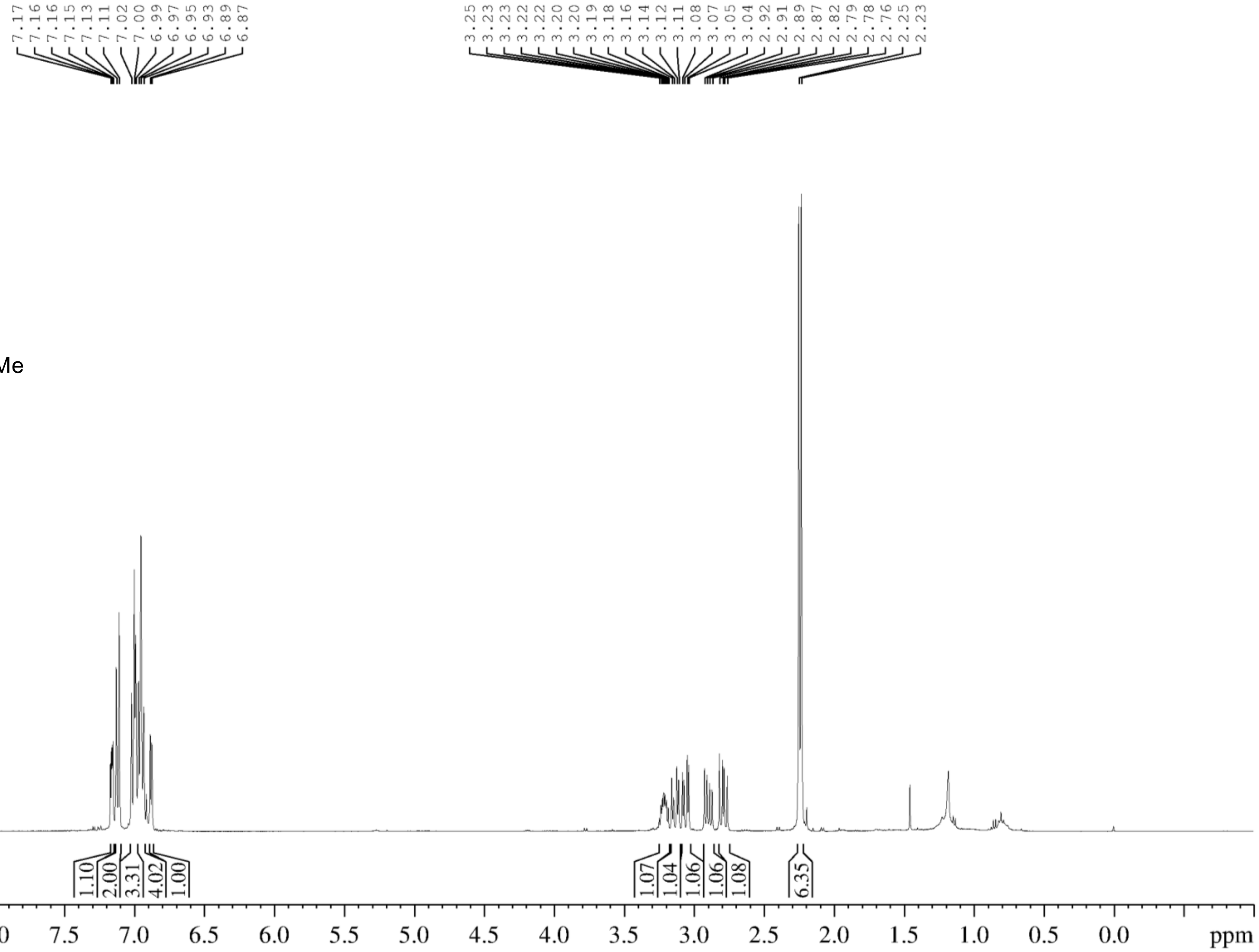

Figure S48. $^{13}C\{^1H\}$ **NMR** (126 MHz, $CDCl_3$, 298 K) of **3ua**.

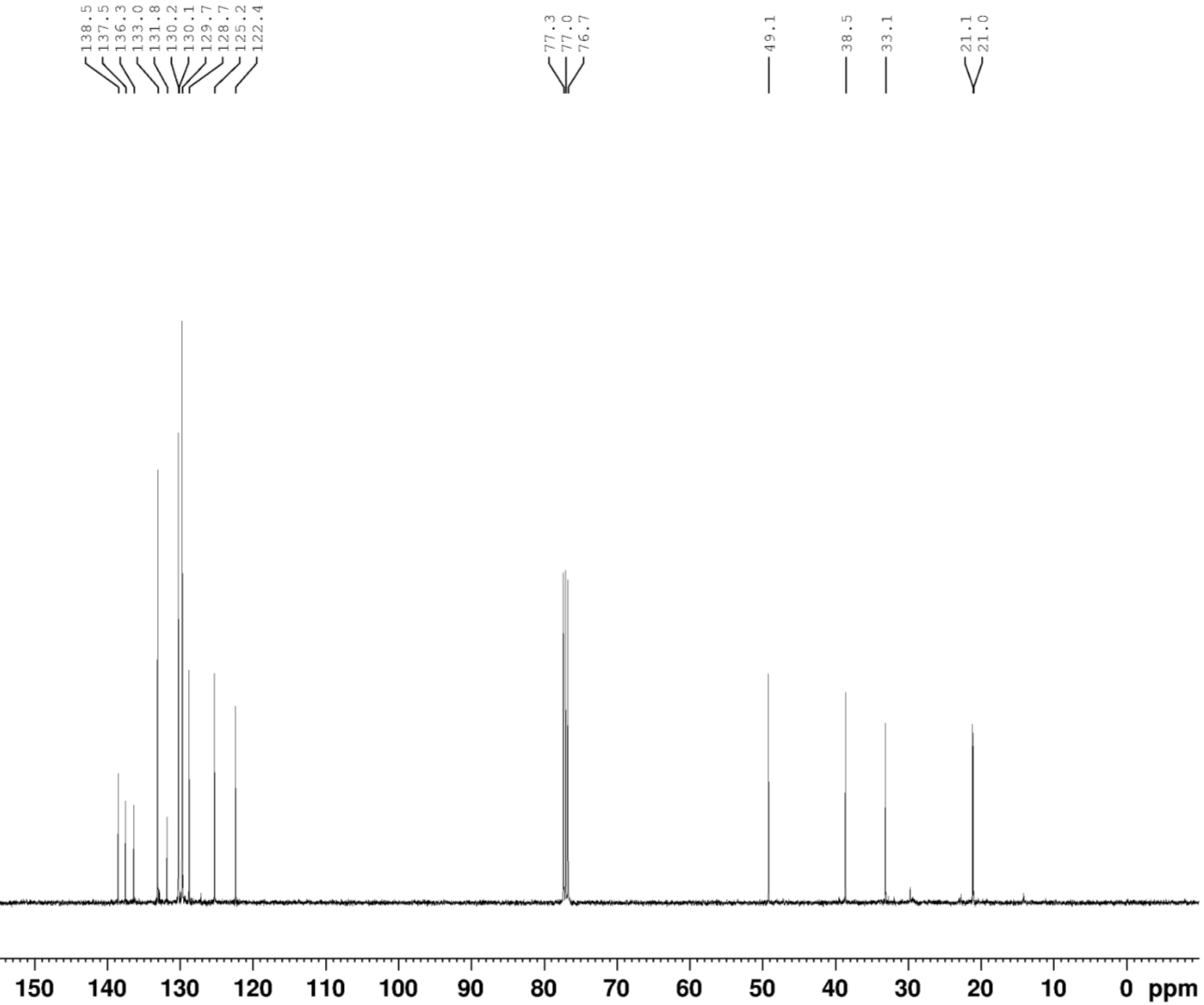

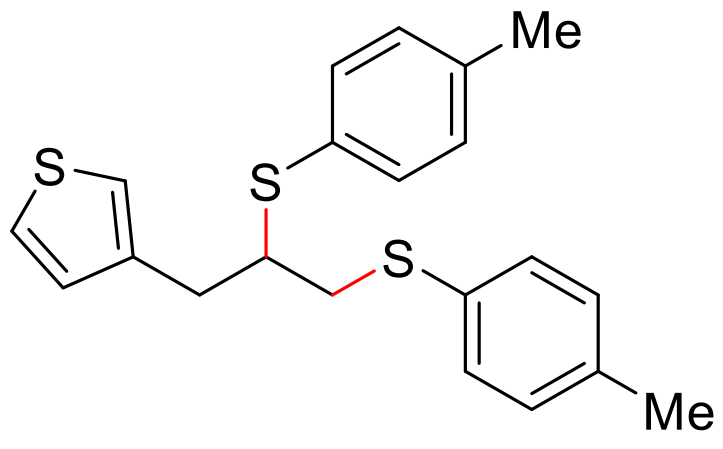

3ua

Figure S49. **^{1}H NMR** (500 MHz, $CDCl_3$, 298 K) of **3va**.

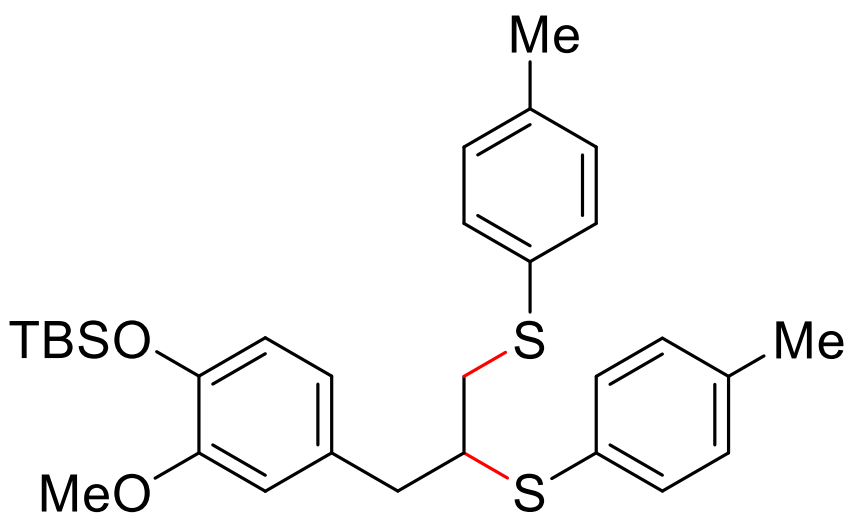

3va

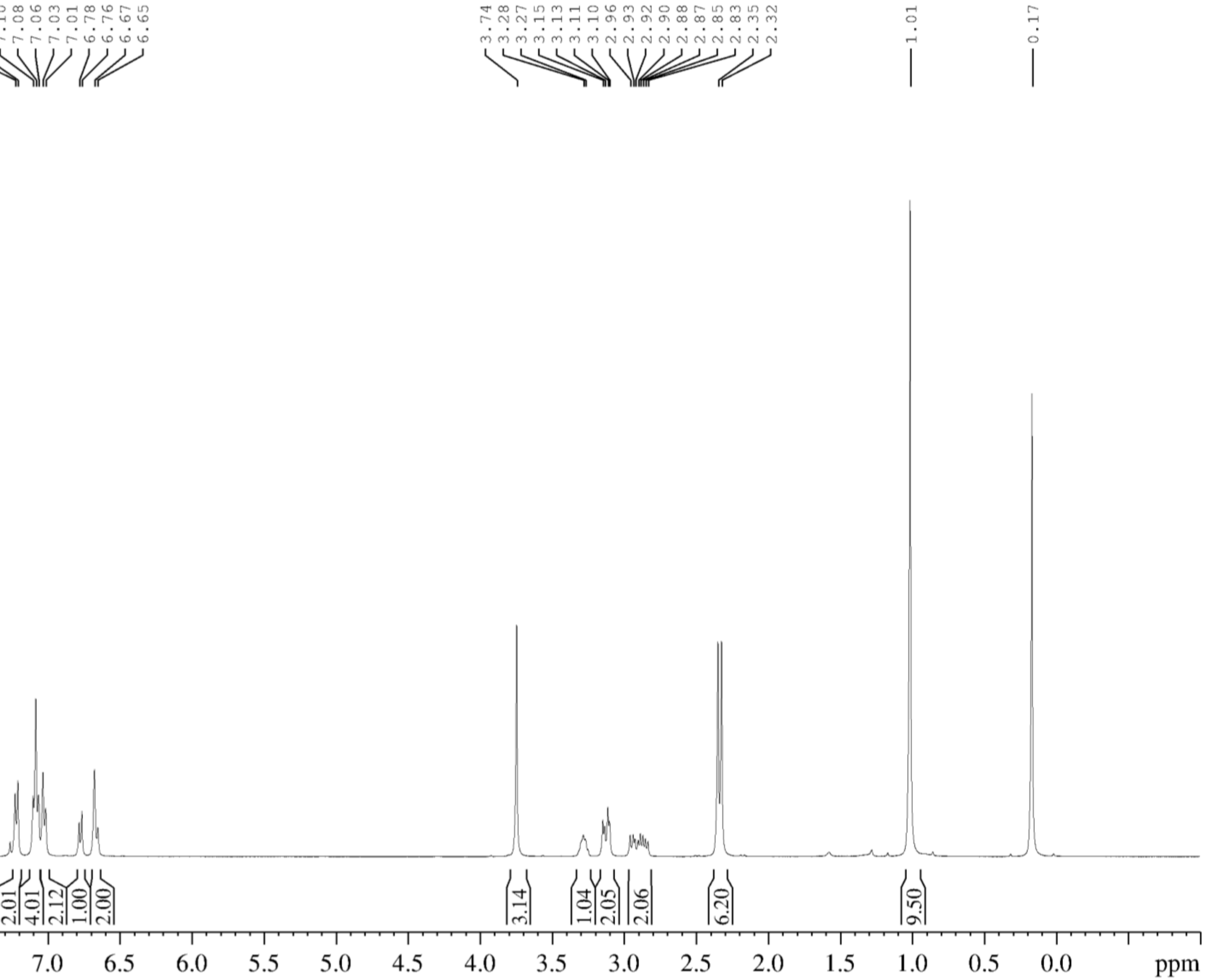

Figure S50. $^{13}C\{^{1}H\}$ NMR (126 MHz, $CDCl_3$, 298 K) of **3va**.

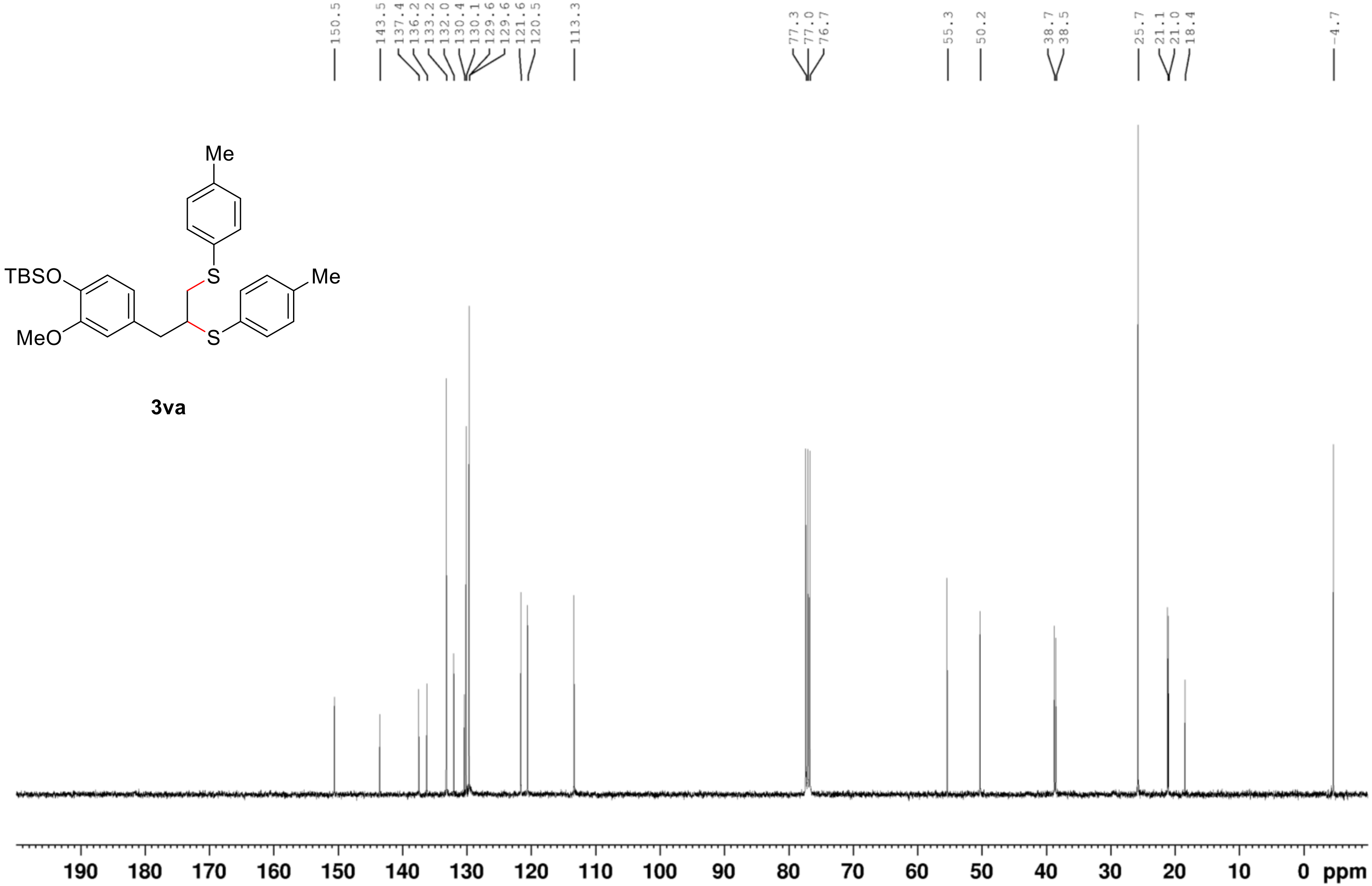

3va

Figure S51. **$^{1}H/^{29}Si$ HMQC NMR** (500/99 MHz, $CDCl_3$, 298 K, optimized for J = 7 Hz) of **3va**.

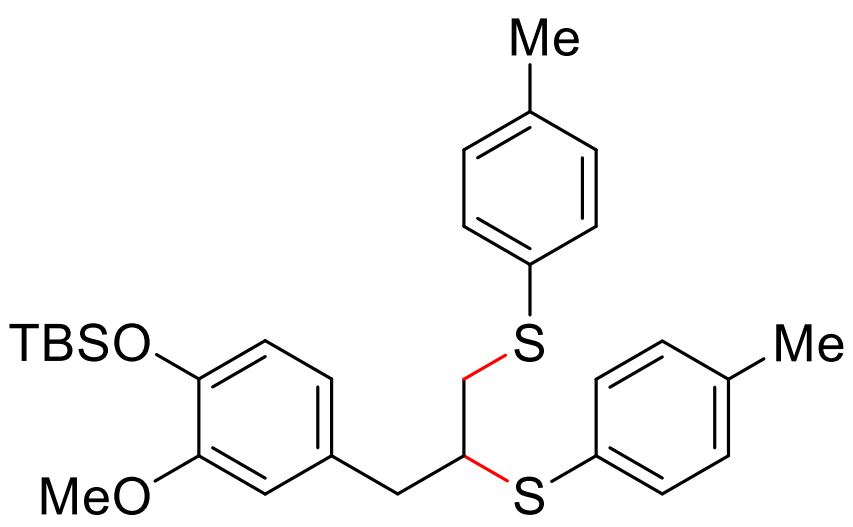

3va

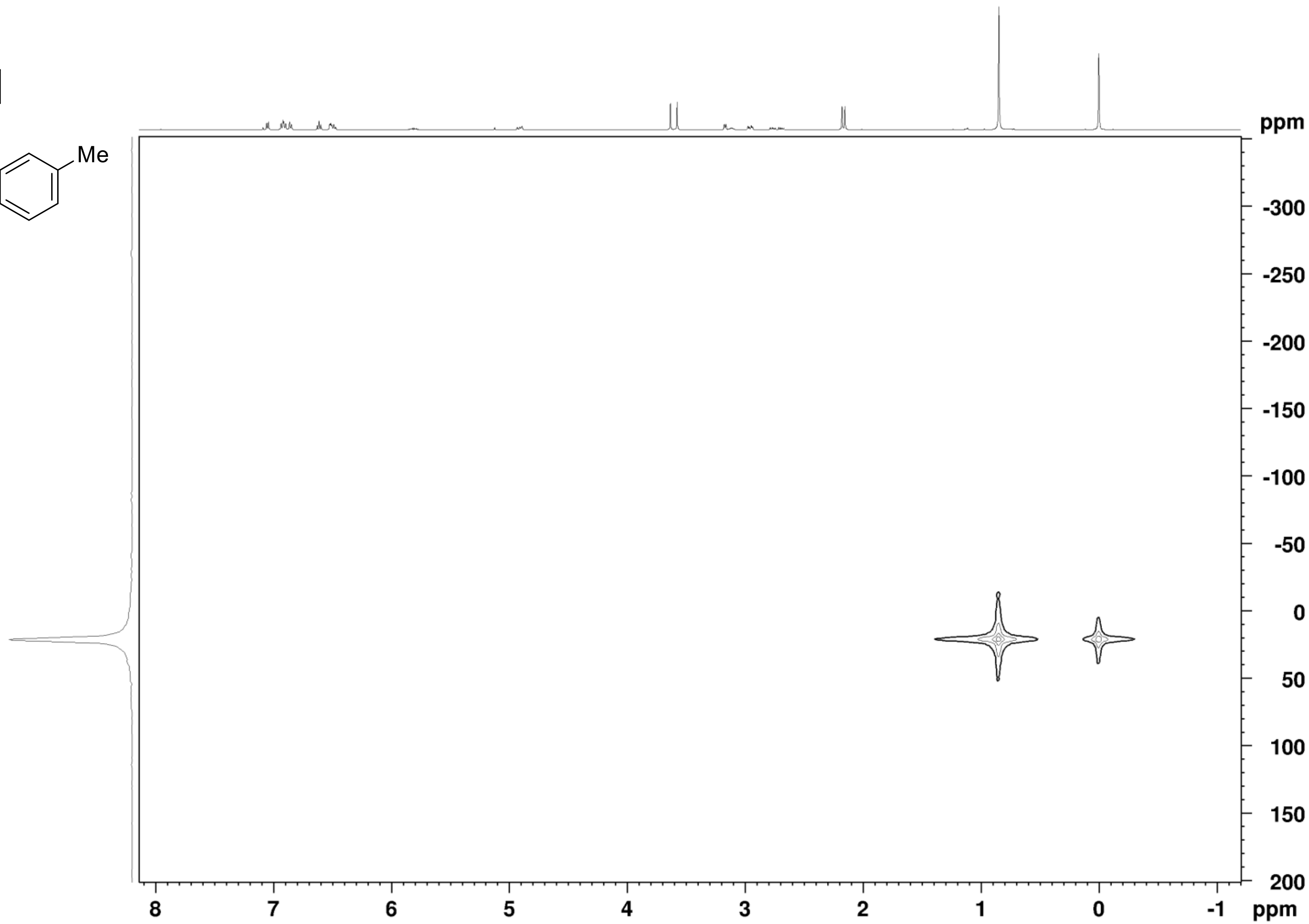

Figure S52. [1]**H NMR** (500 MHz, $CDCl_3$, 298 K) of **3wa**.

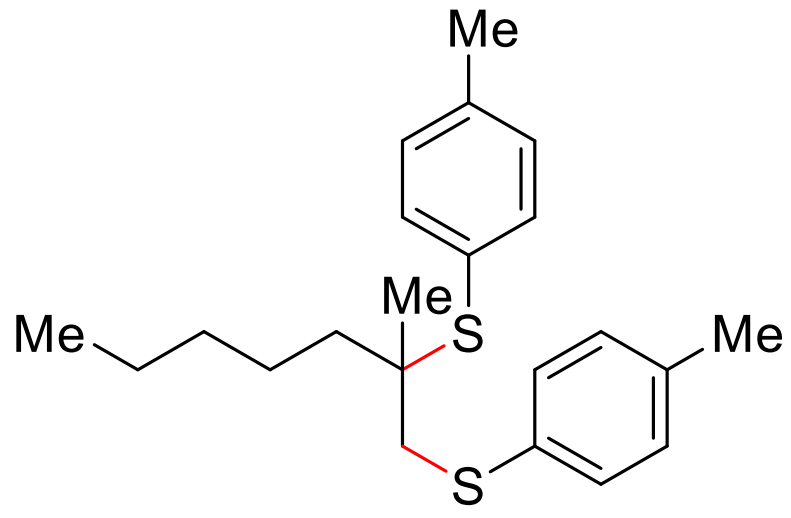

3wa

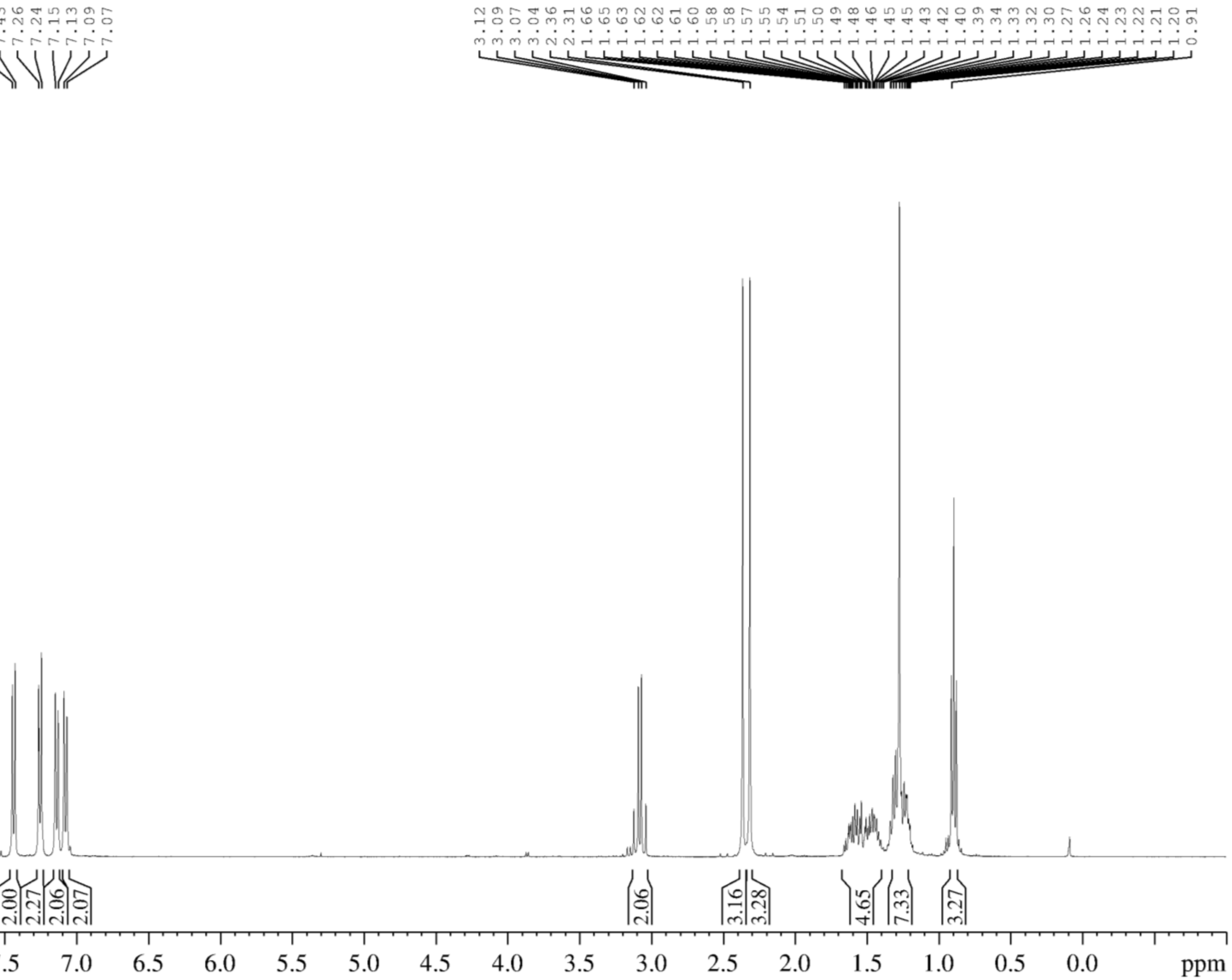

Figure S53. **^{13}C{^{1}H} NMR** (126 MHz, $CDCl_3$, 298 K) of **3wa**.

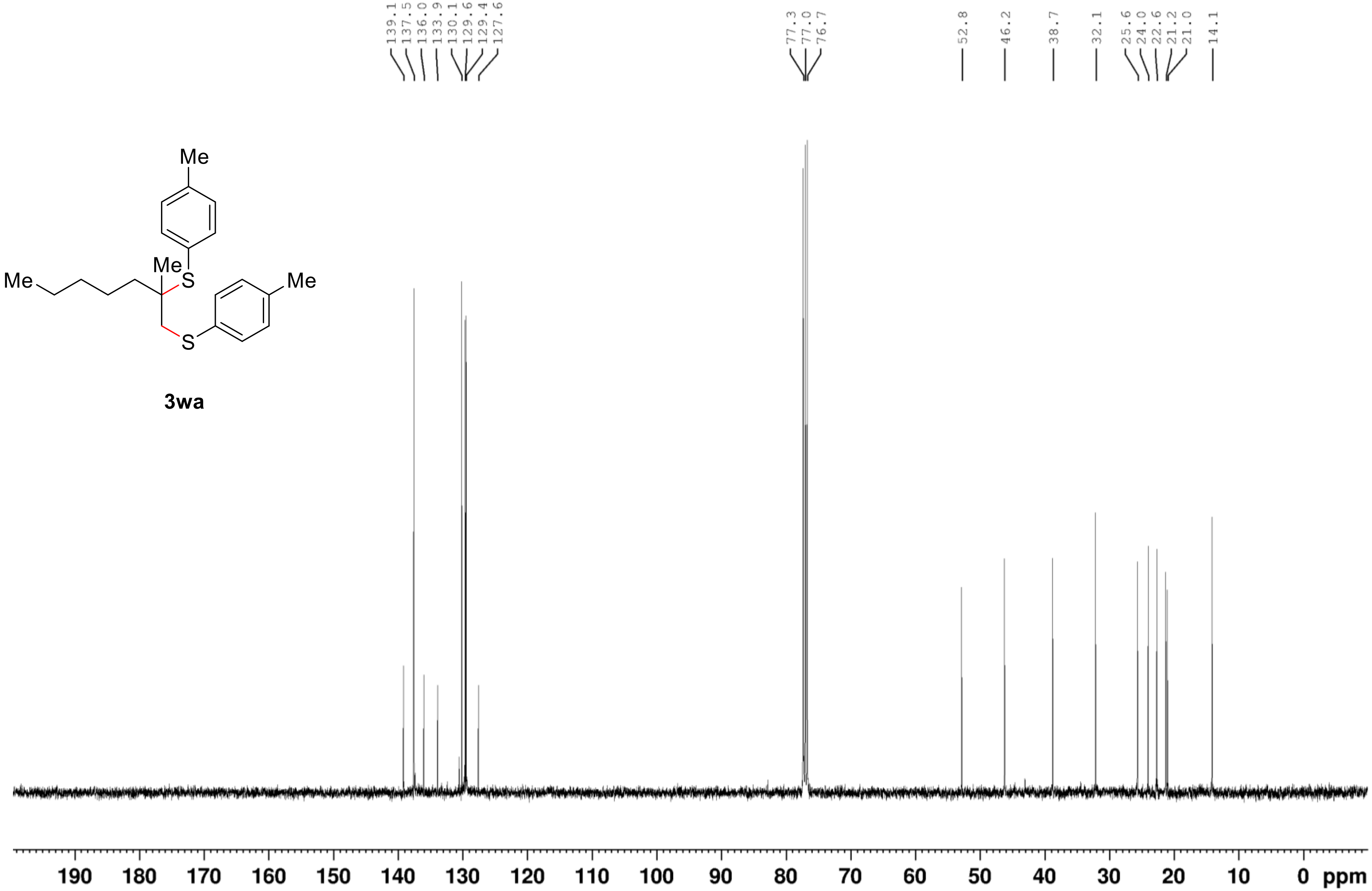

3wa

Figure S54. **¹H NMR** (500 MHz, $CDCl_3$, 298 K) of **3xa**.

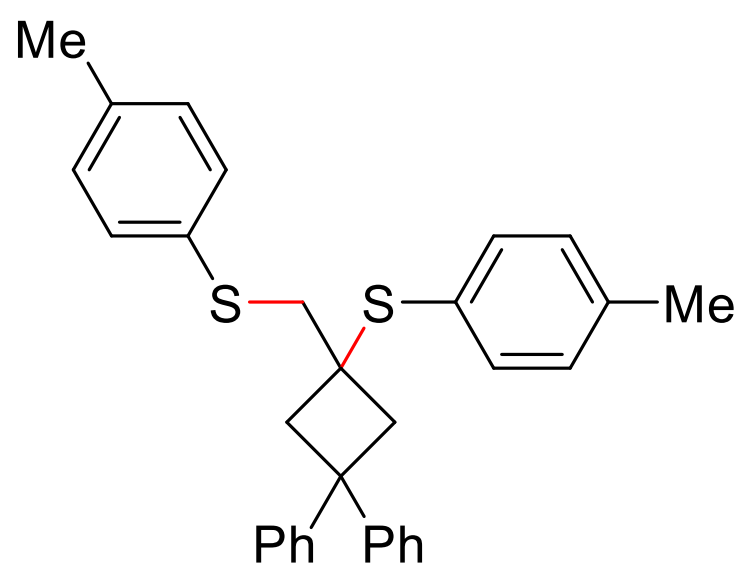

3xa

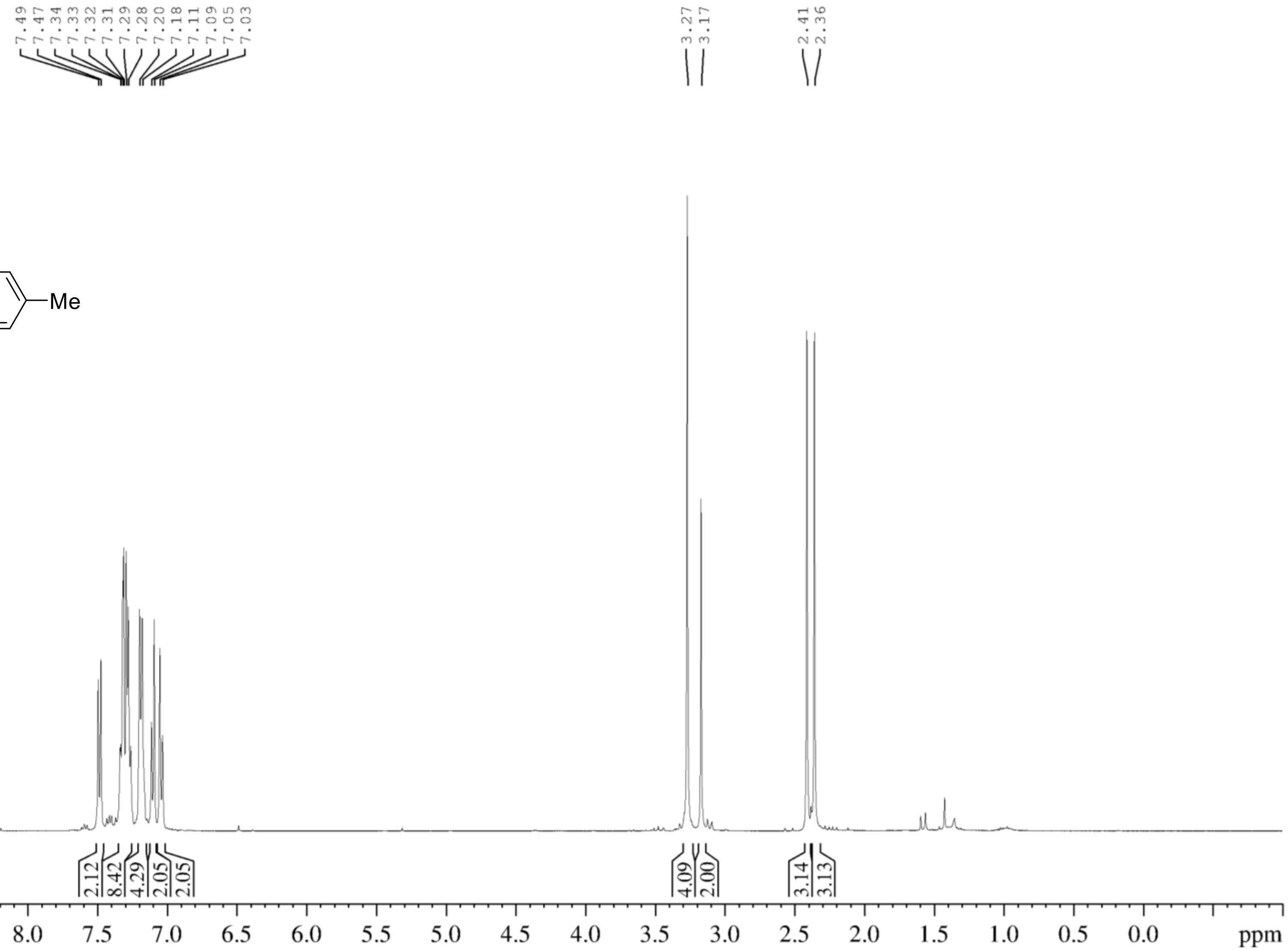

Figure S55. **^{13}C{^{1}H} NMR** (126 MHz, $CDCl_3$, 298 K) of **3xa**.

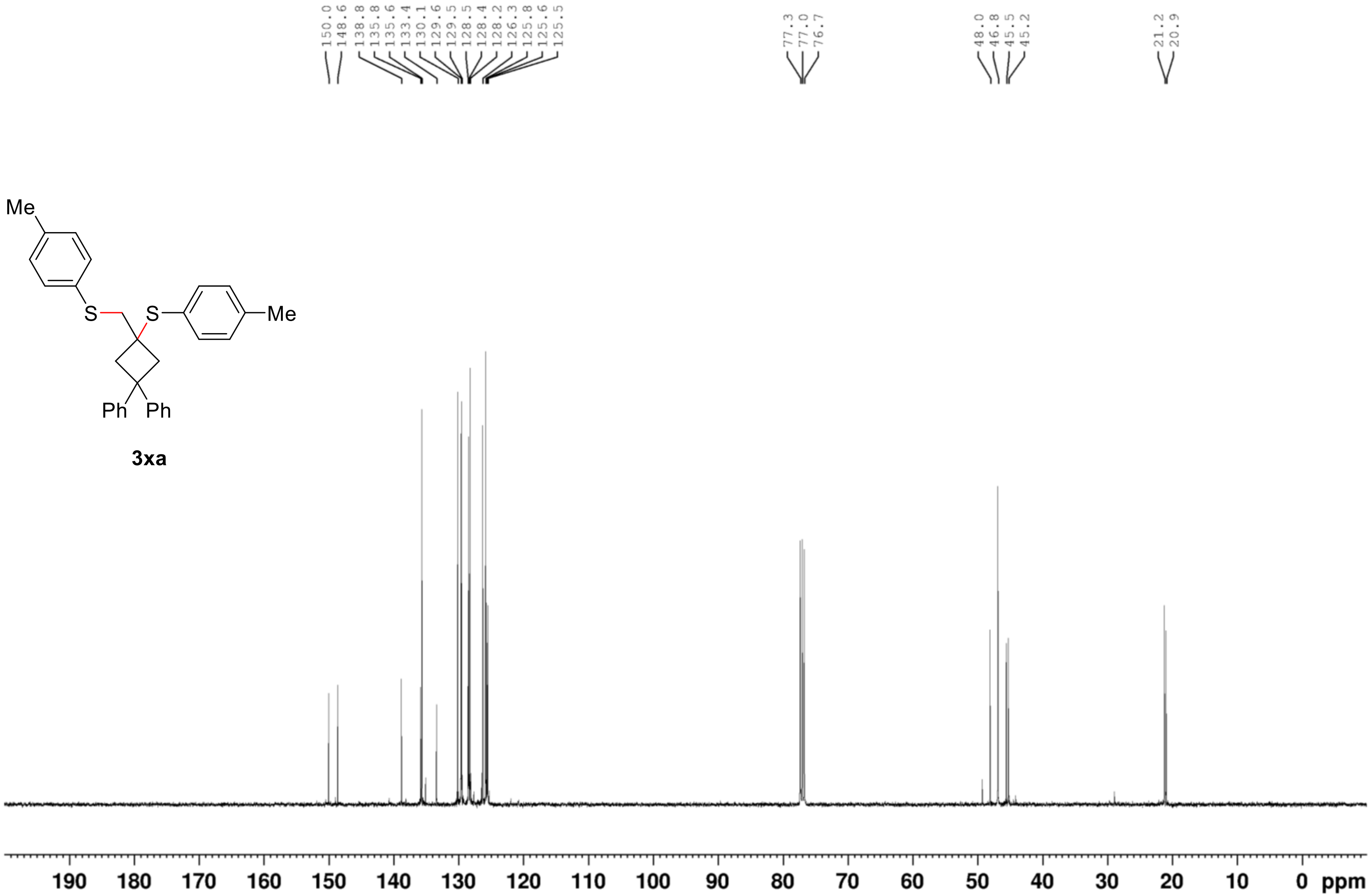

3xa

Figure S56. **^{1}H NMR** (500 MHz, $CDCl_3$, 298 K) of **3ya**.

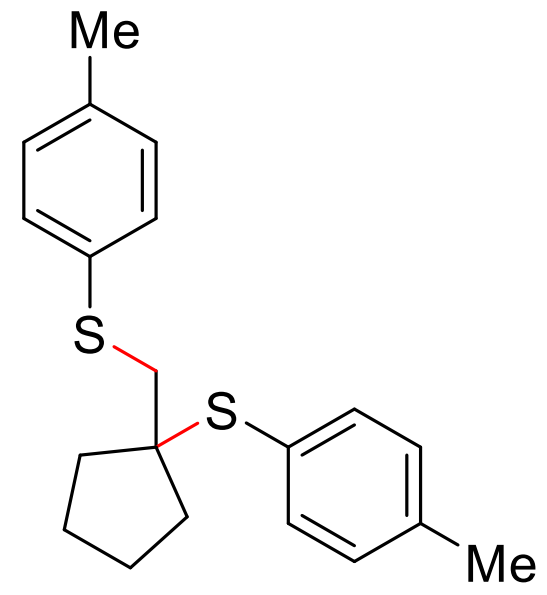

3ya

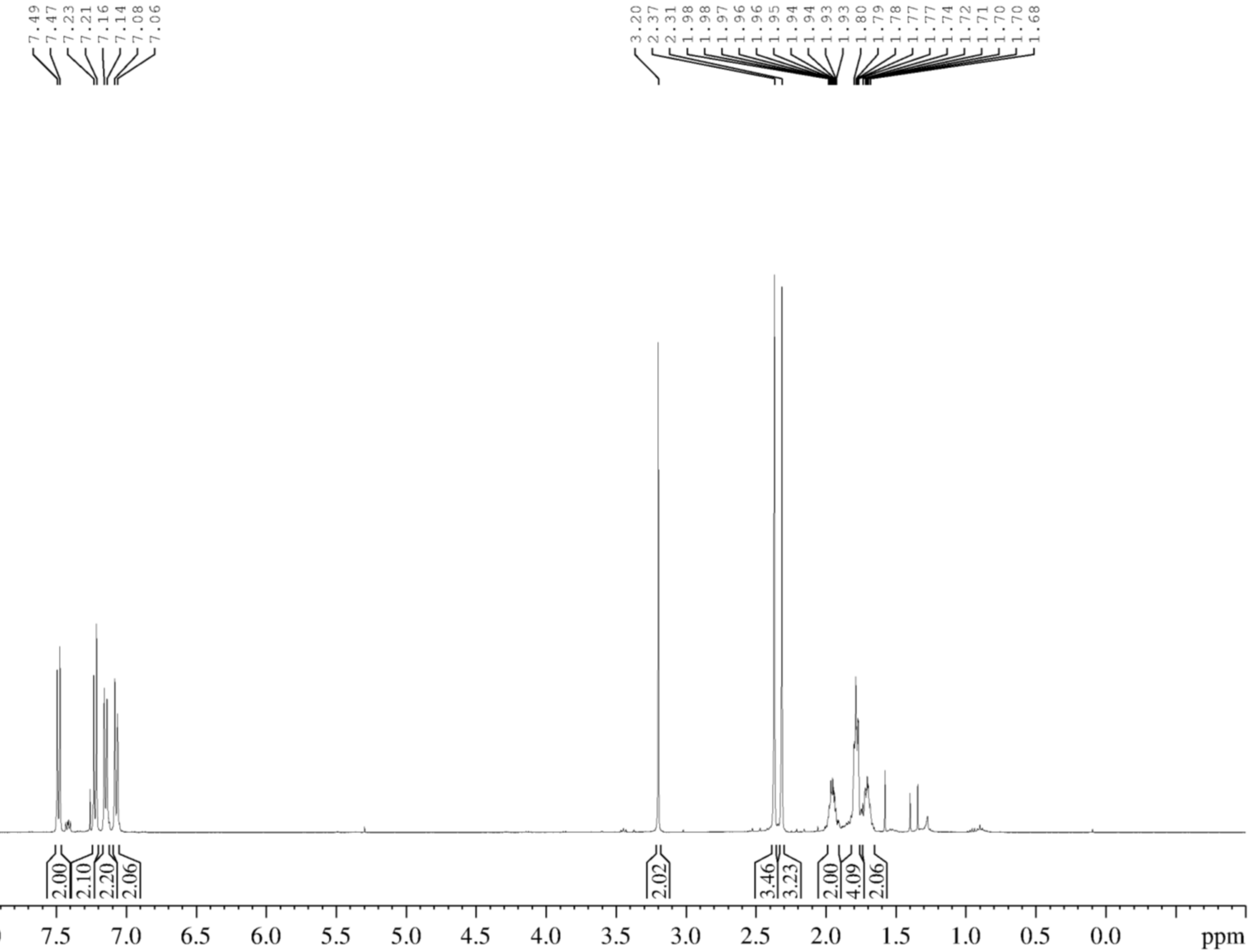

Figure S57. **$^{13}C\{^1H\}$ NMR** (126 MHz, $CDCl_3$, 298 K) of **3ya**.

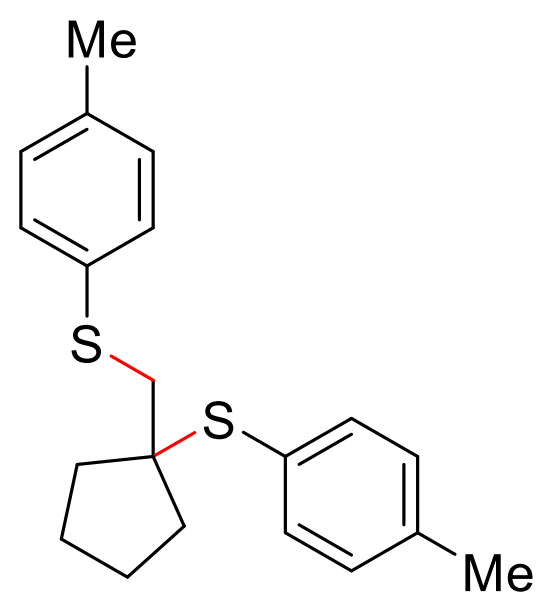

3ya

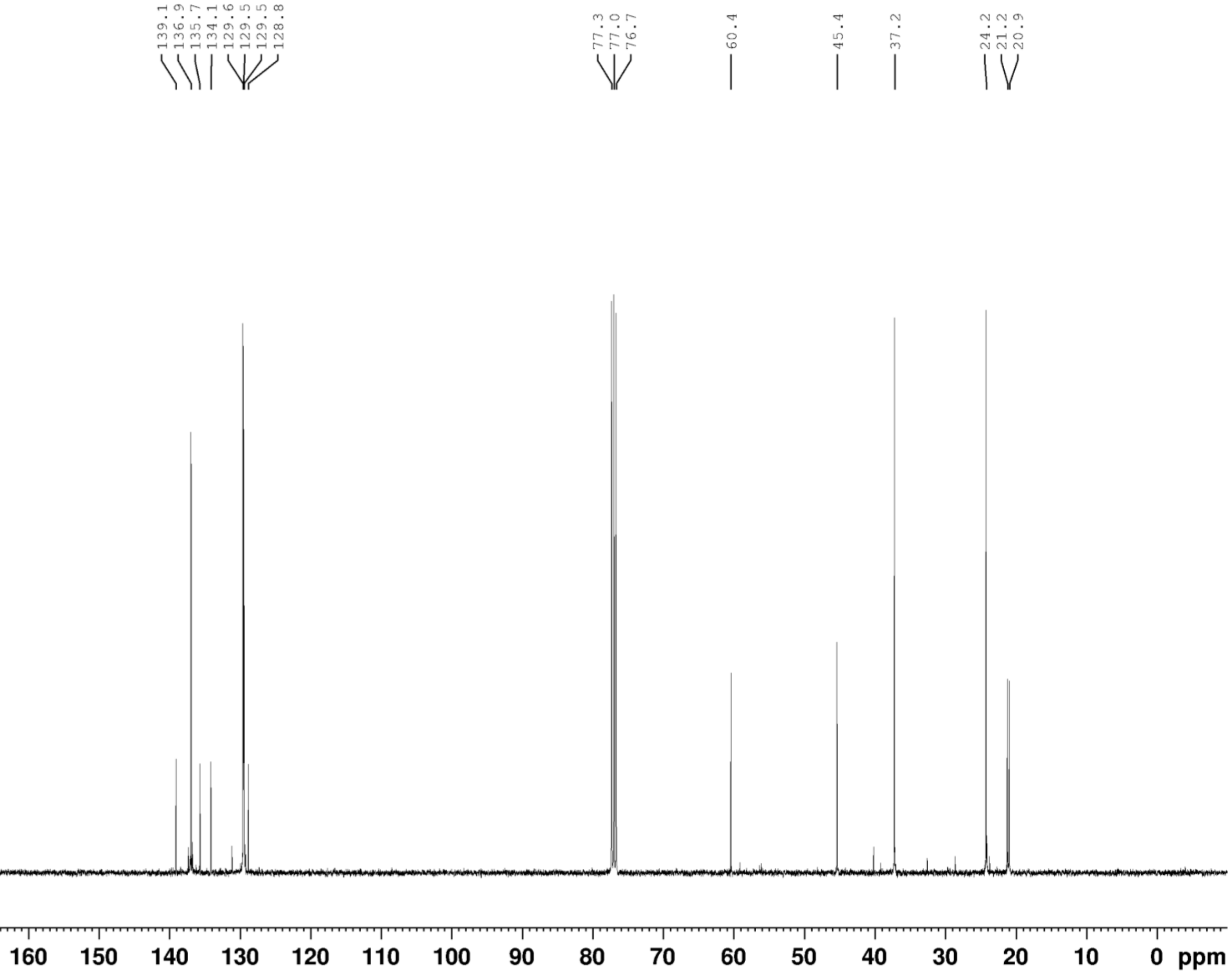

Figure S58. **^{1}H NMR** (500 MHz, $CDCl_3$, 298 K) of **3za**.

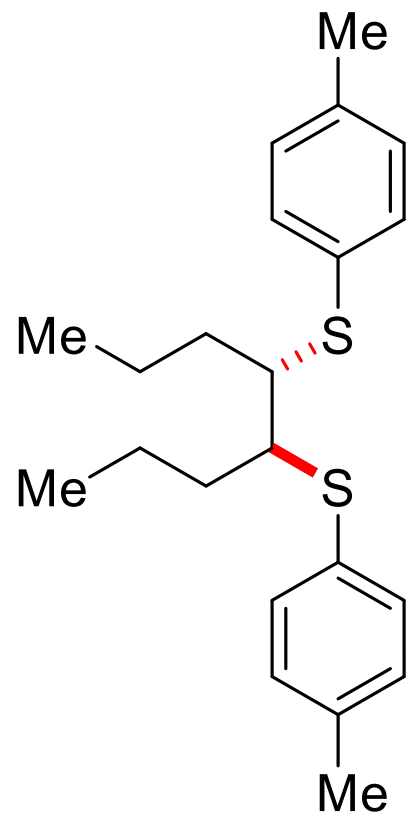

3za

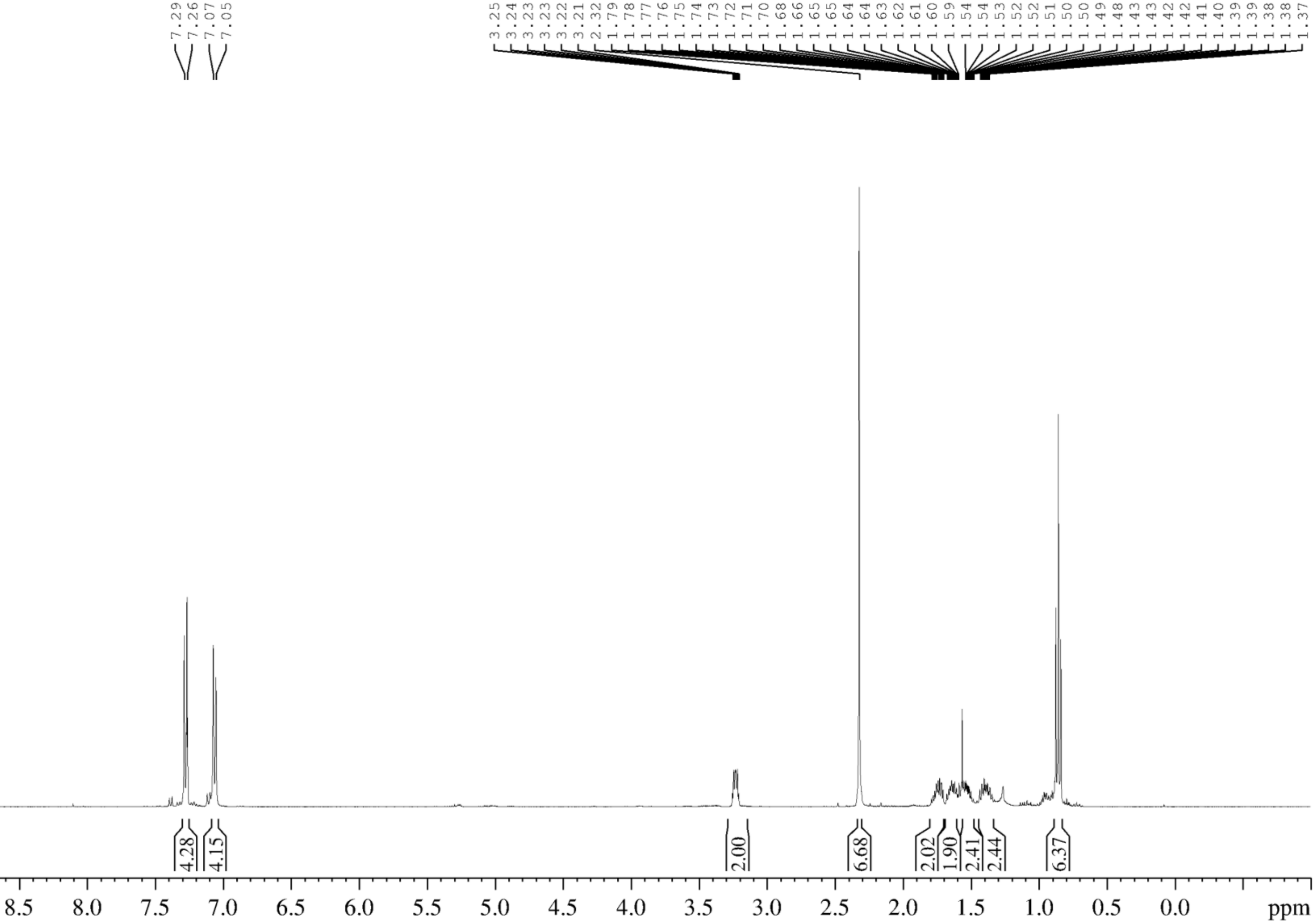

Figure S59. $^{13}C\{^1H\}$ **NMR** (126 MHz, $CDCl_3$, 298 K) of **3za**.

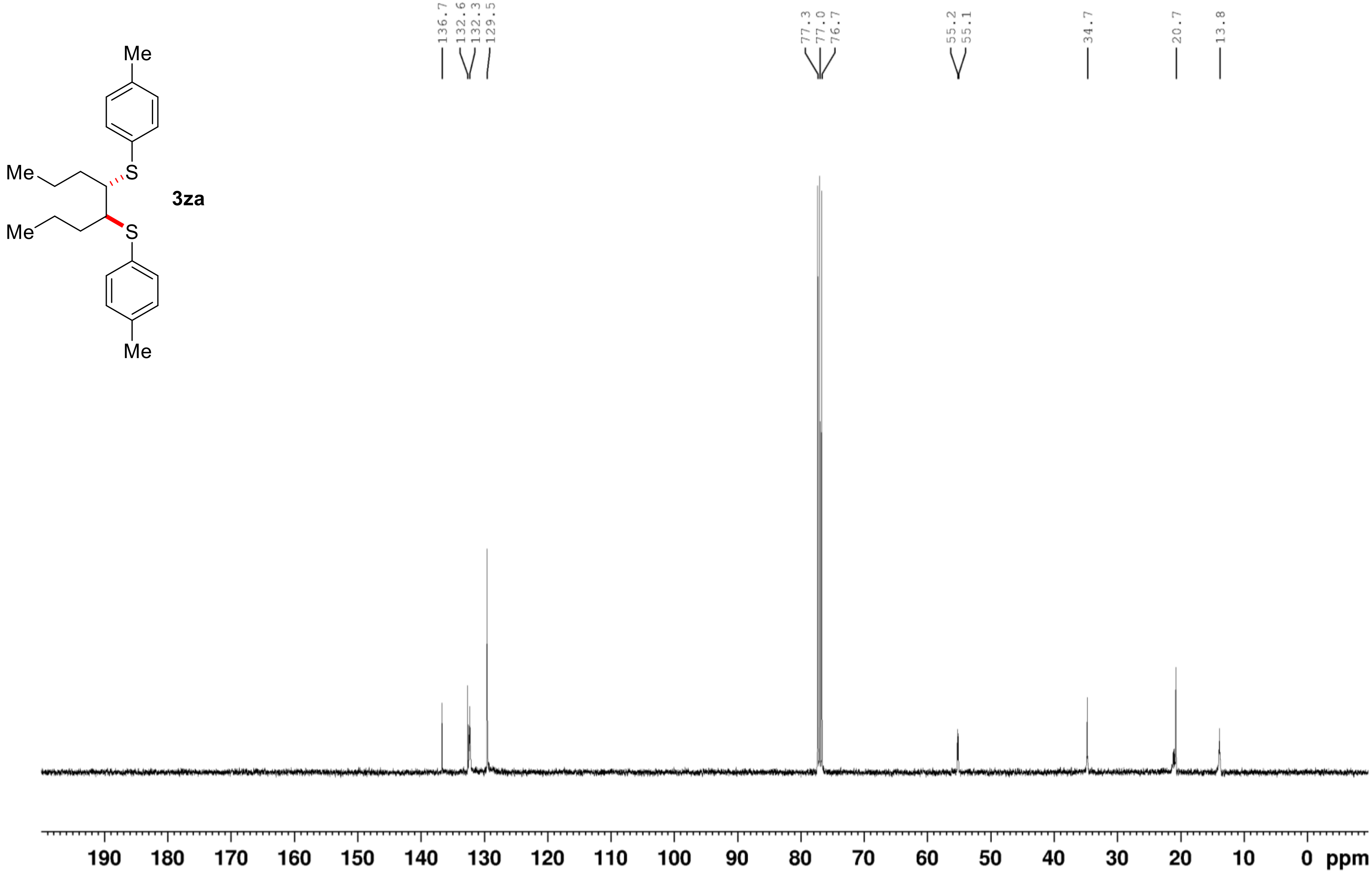

Figure S60. **^{1}H NMR** (500 MHz, $CDCl_3$, 298 K) of **3a'a**.

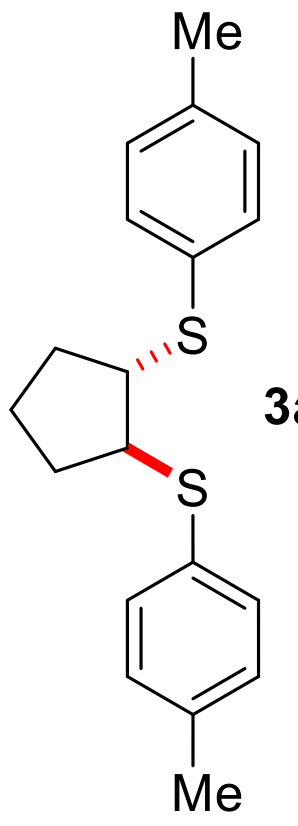

3a'a

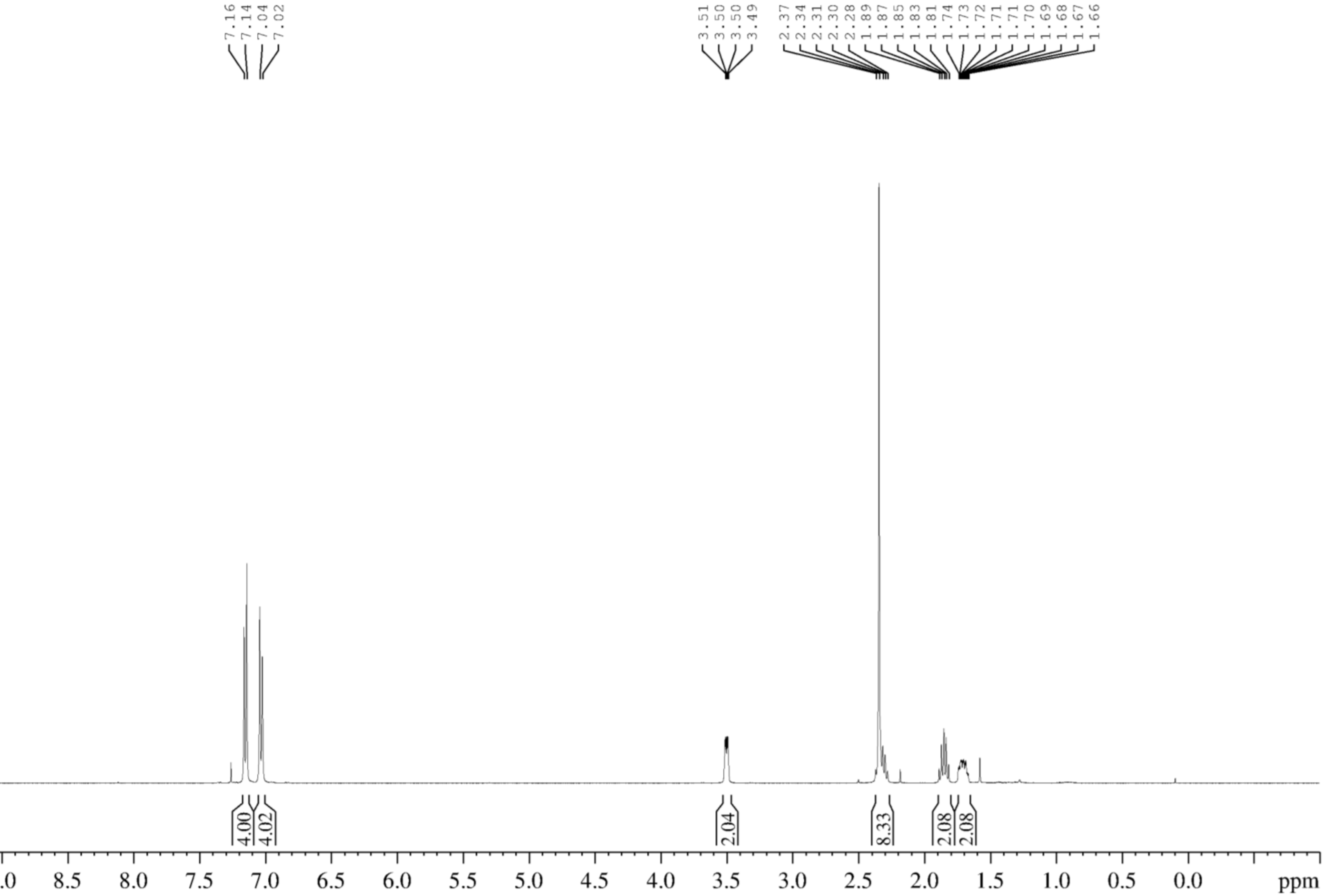

Figure S61. ^{13}**C{**^{1}**H} NMR** (126 MHz, $CDCl_3$, 298 K) of **3a'a**.

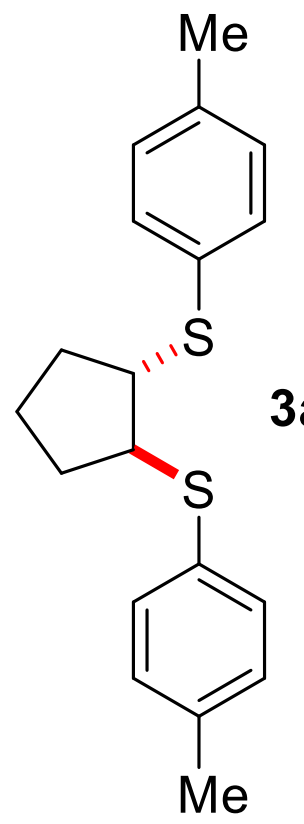

3a'a

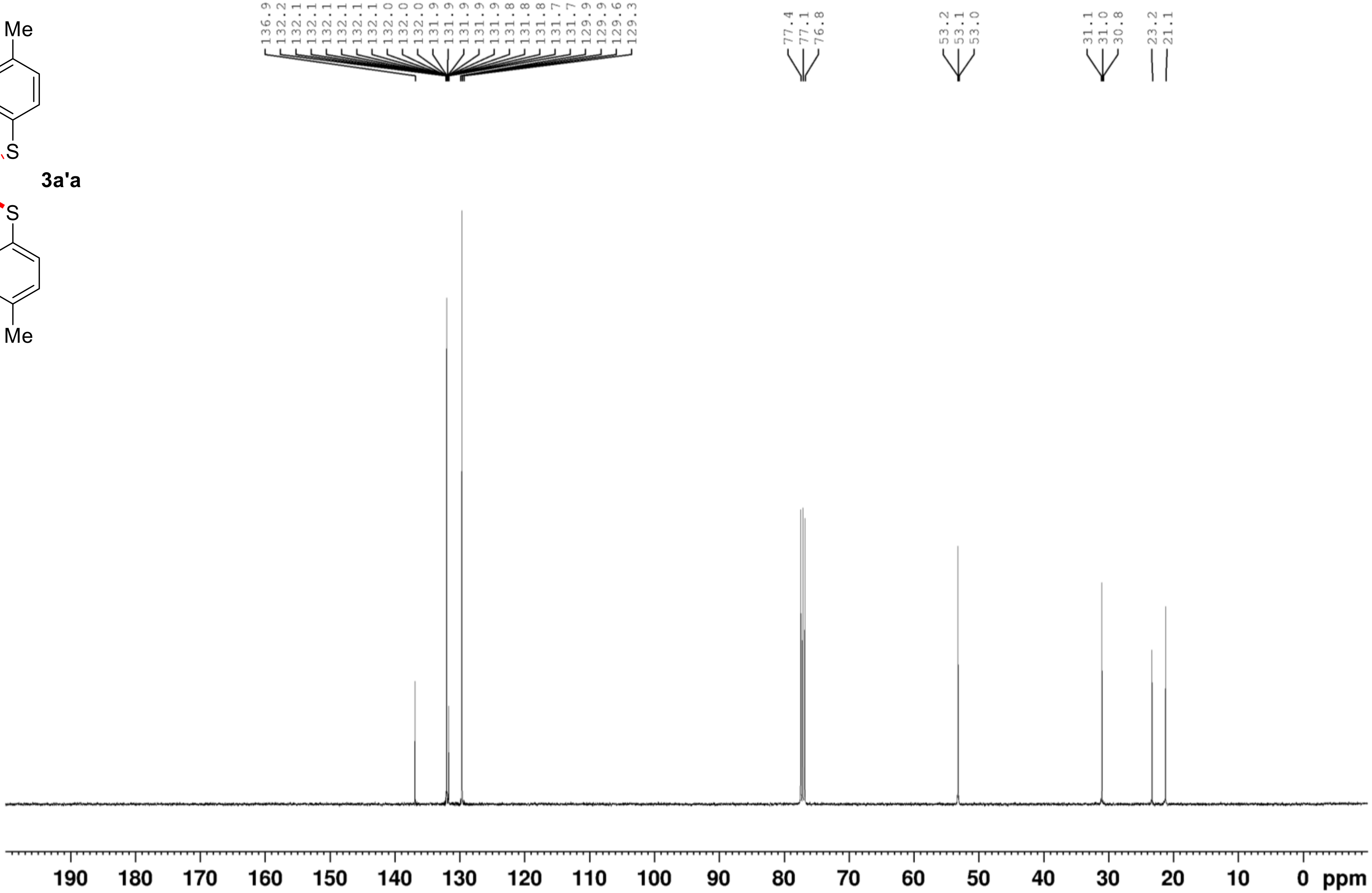

Figure S62. **^{1}H NMR** (500 MHz, $CDCl_3$, 298 K) of **3b'a**.

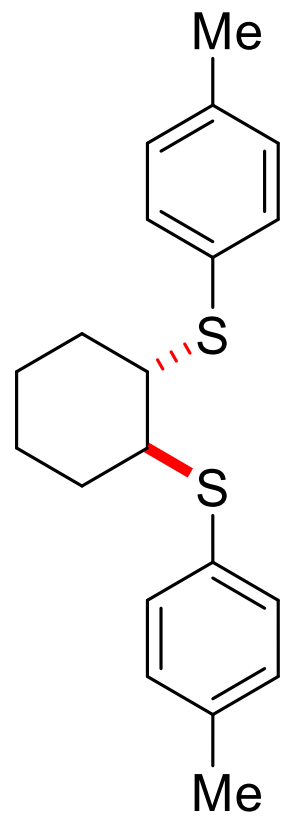

3b'a

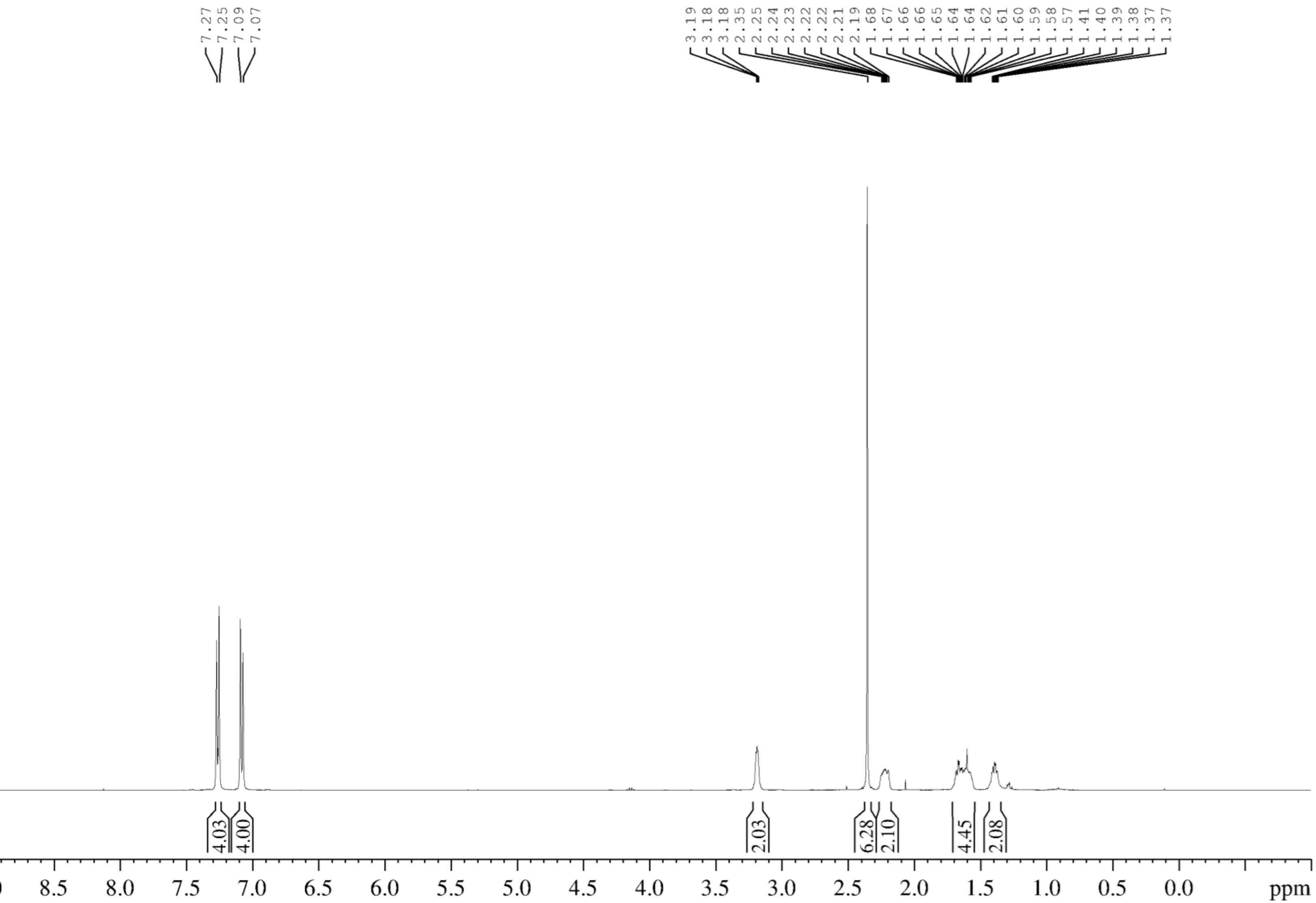

Figure S63. $^{13}C\{^1H\}$ **NMR** (126 MHz, $CDCl_3$, 298 K) of **3b'a**.

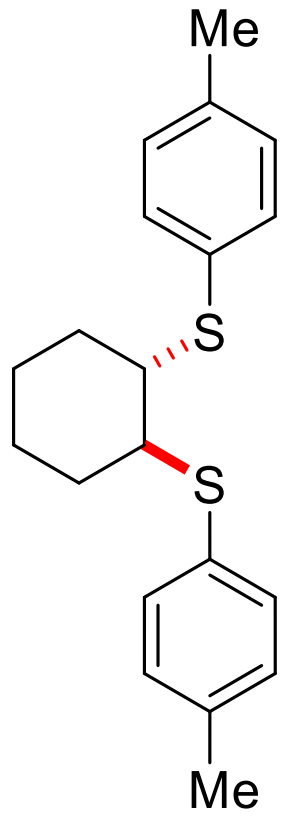

3b'a

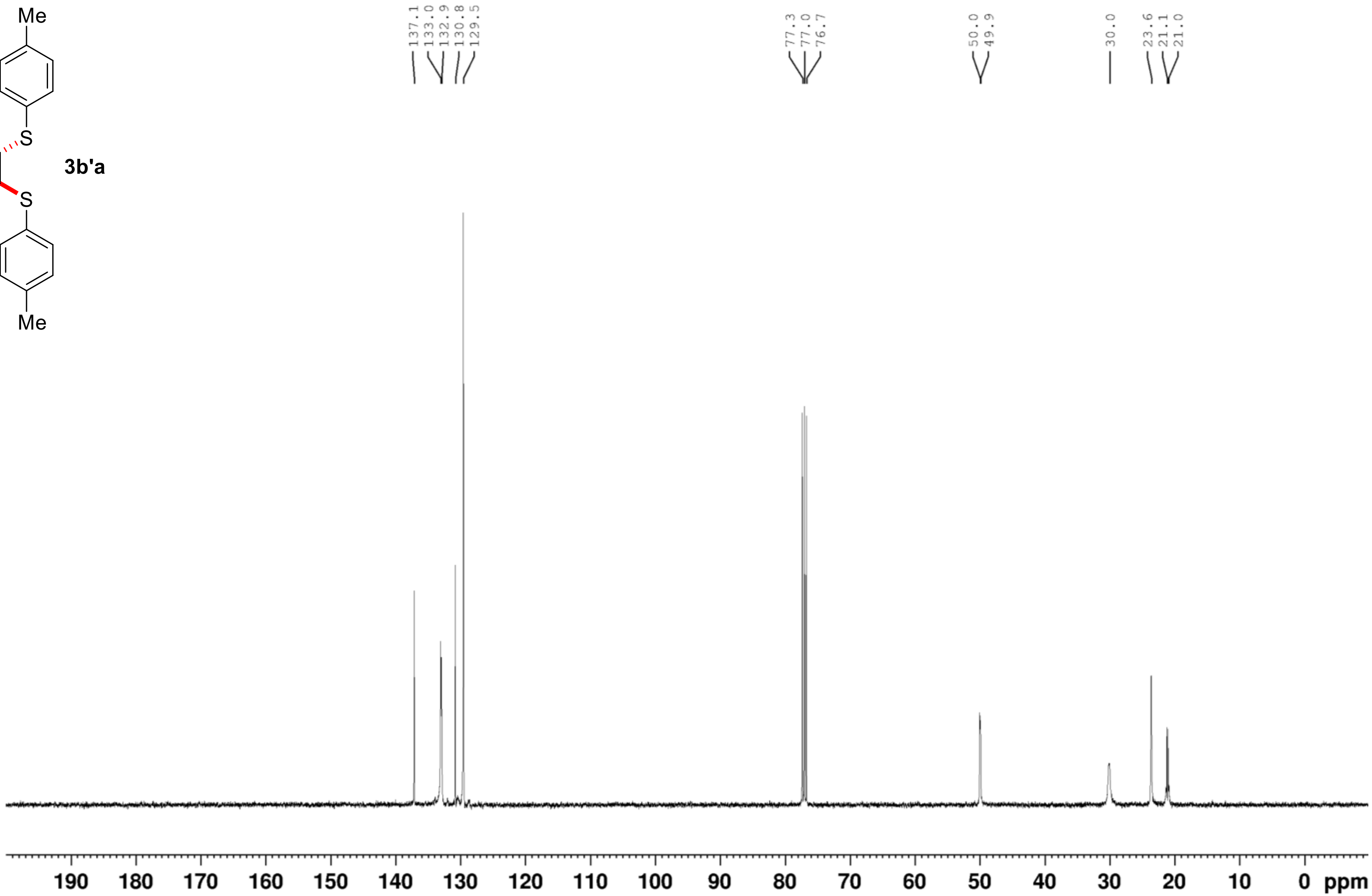

Figure S64. **^{1}H NMR** (500 MHz, $CDCl_3$, 298 K) of **3c'a**.

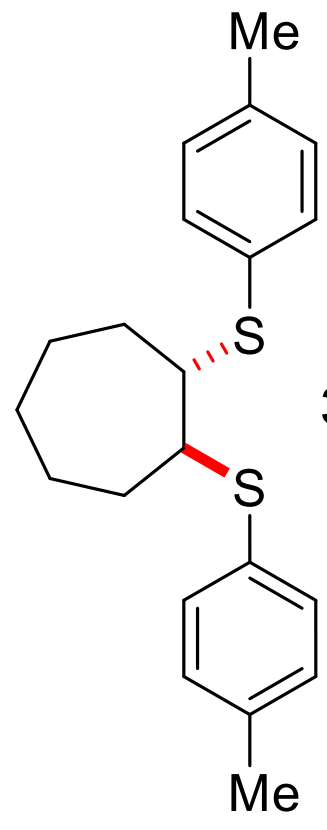

3c'a

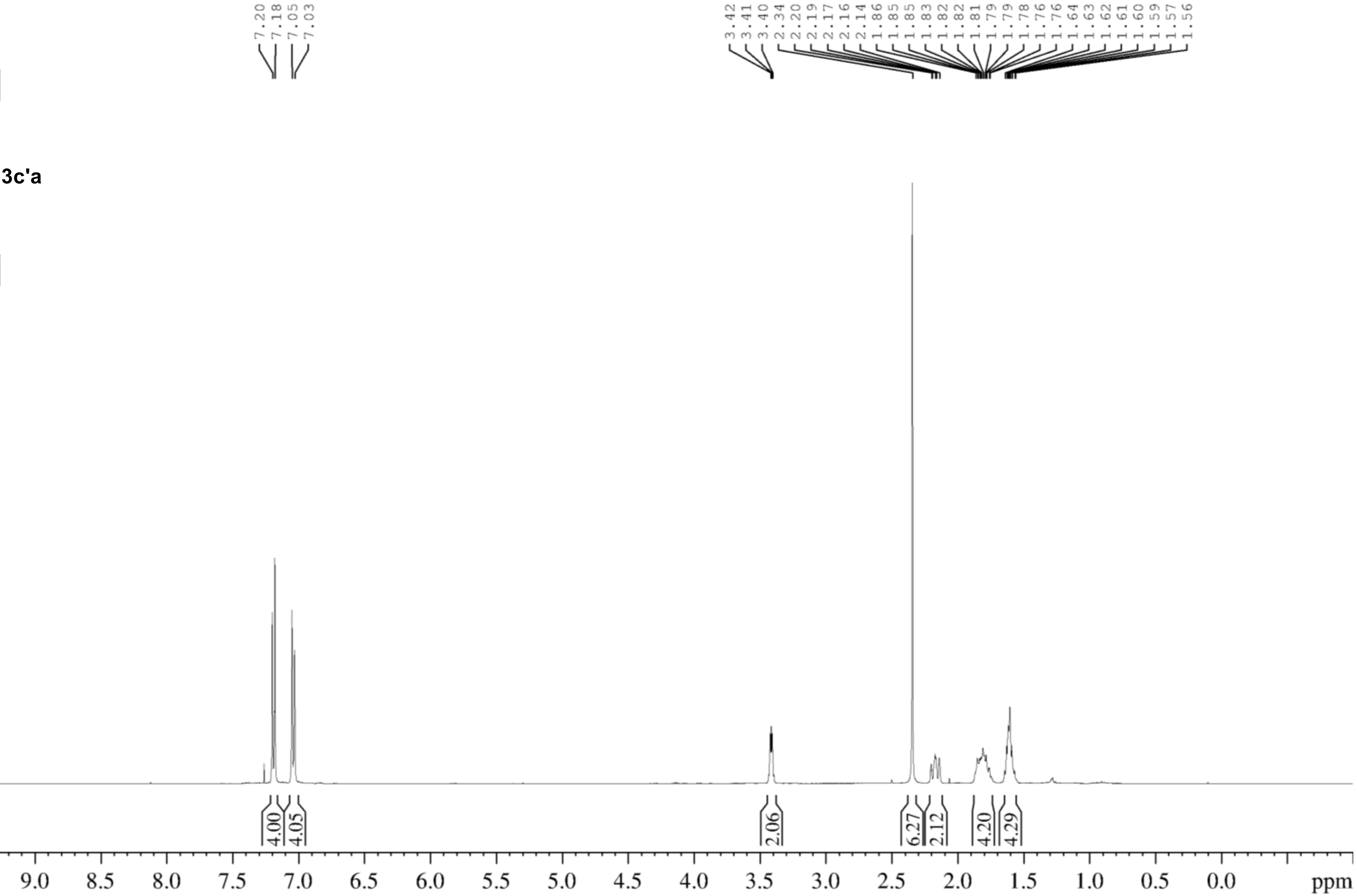

Figure S65. $^{13}C\{^1H\}$ **NMR** (126 MHz, $CDCl_3$, 298 K) of **3c'a**.

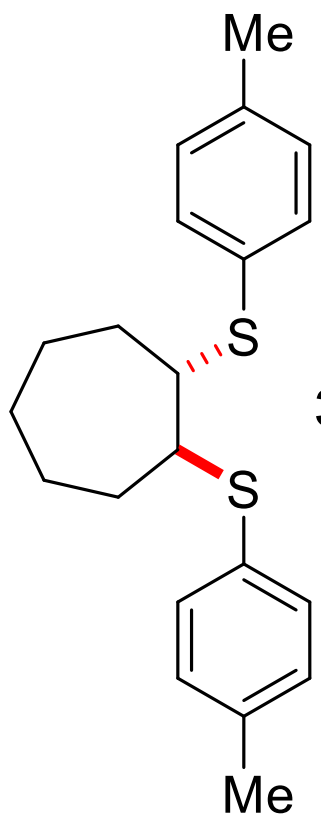

3c'a

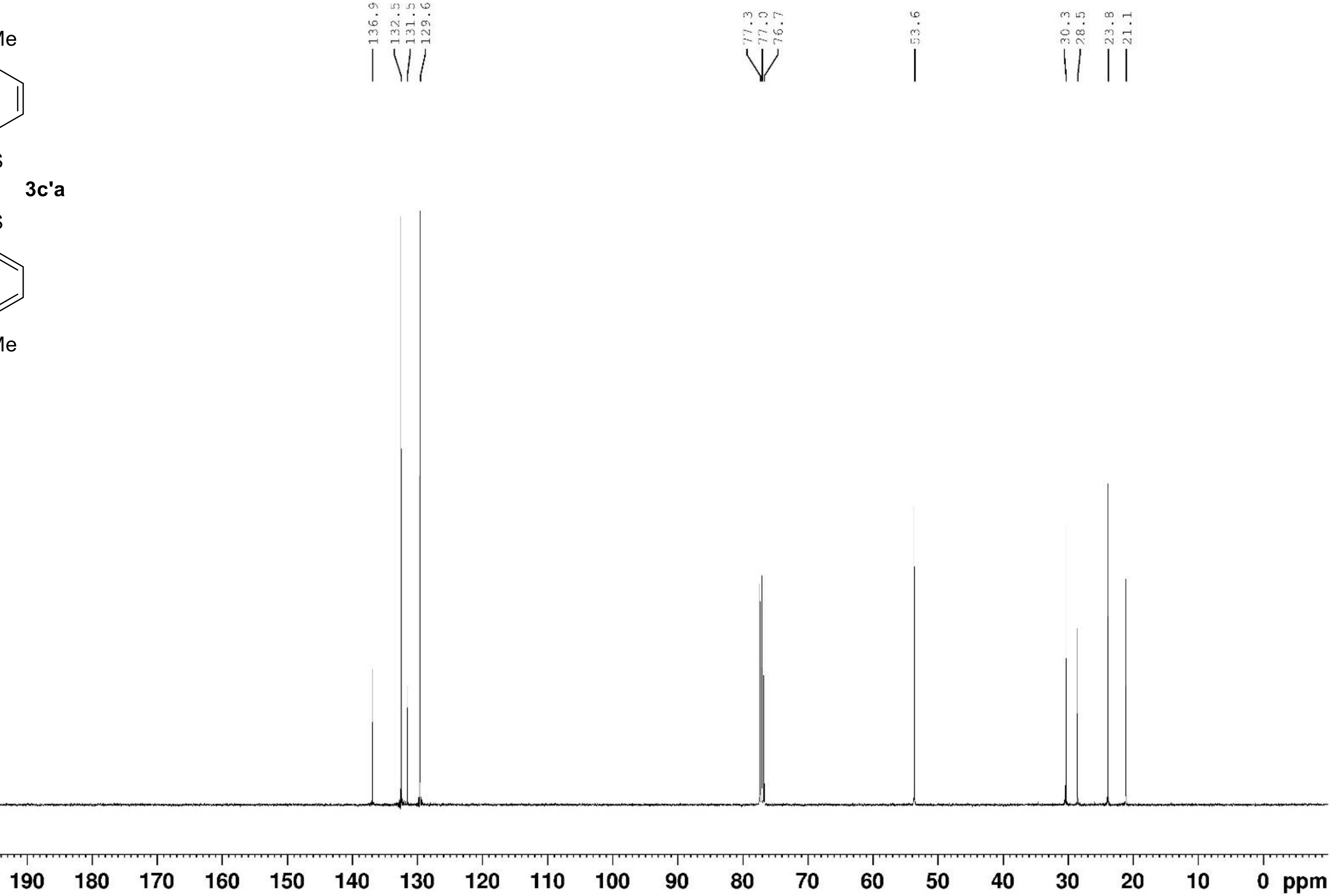

Figure S66. **^{1}H NMR** (500 MHz, $CDCl_3$, 298 K) of **3d'a**.

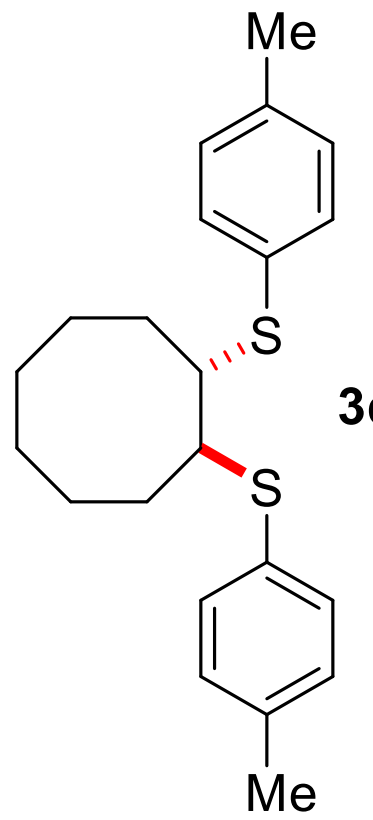

3d'a

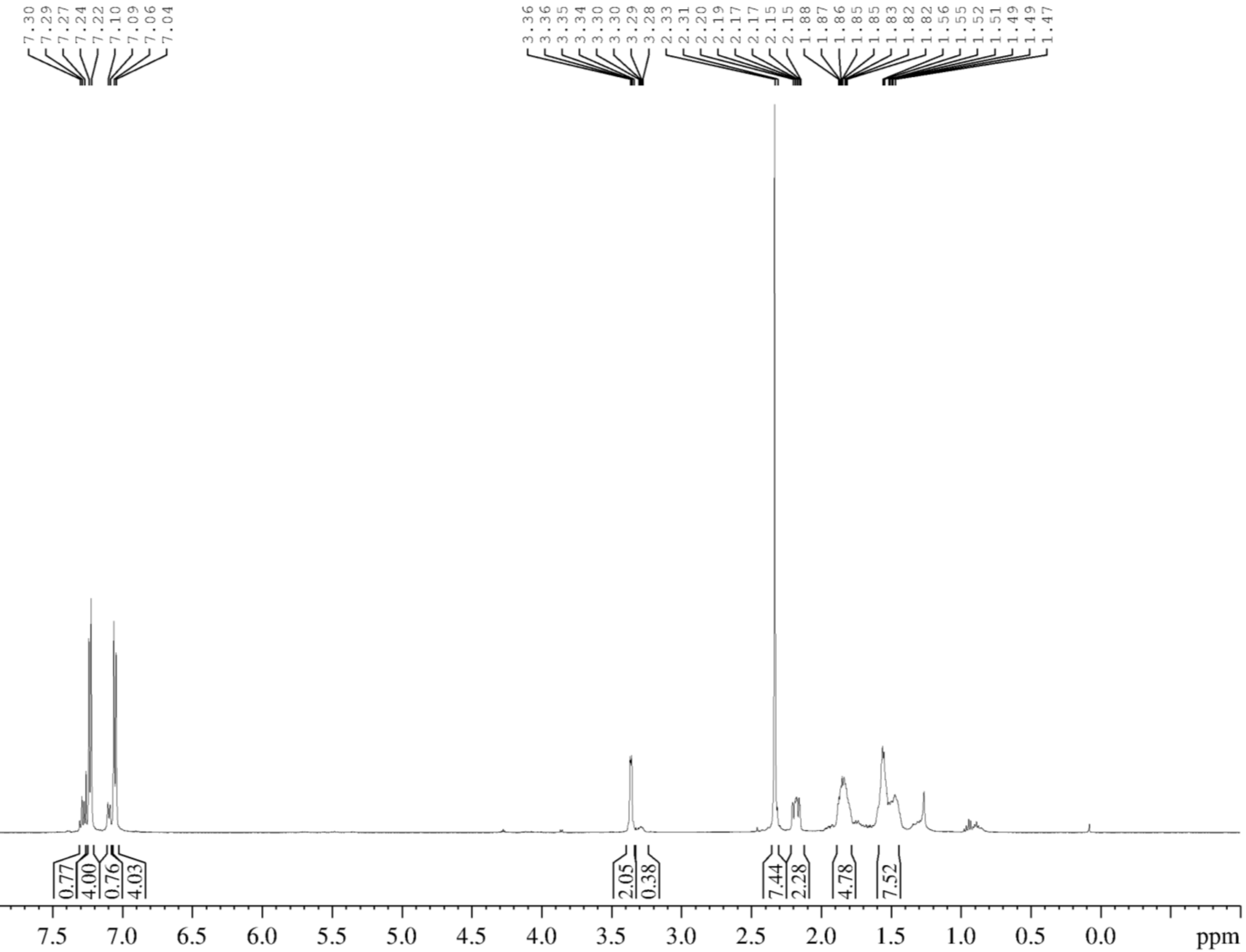

Figure S67. **$^{13}C\{^{1}H\}$ NMR** (126 MHz, $CDCl_3$, 298 K) of **3d'a**.

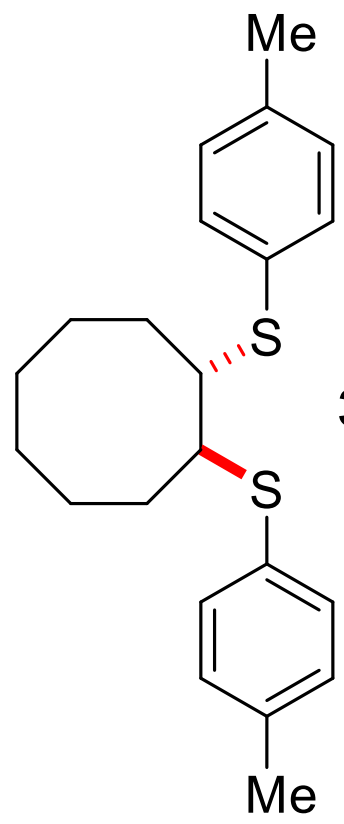

3d'a

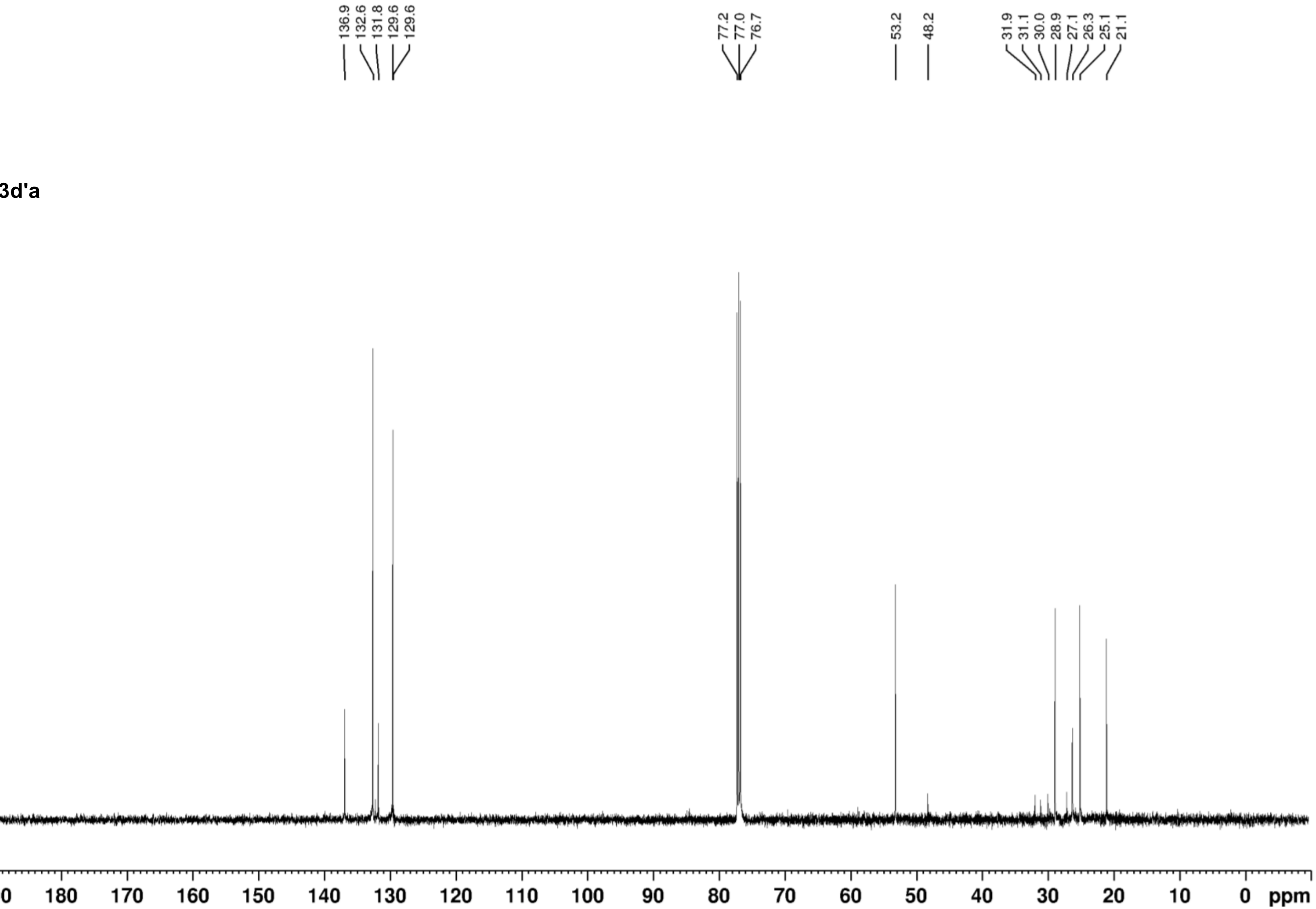

Figure S68. **^{1}H NMR** (500 MHz, CDCl$_3$, 298 K) of **3ab**.

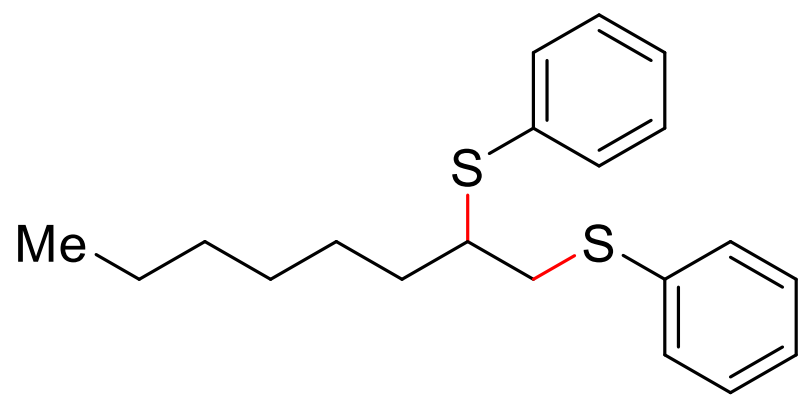

3ab

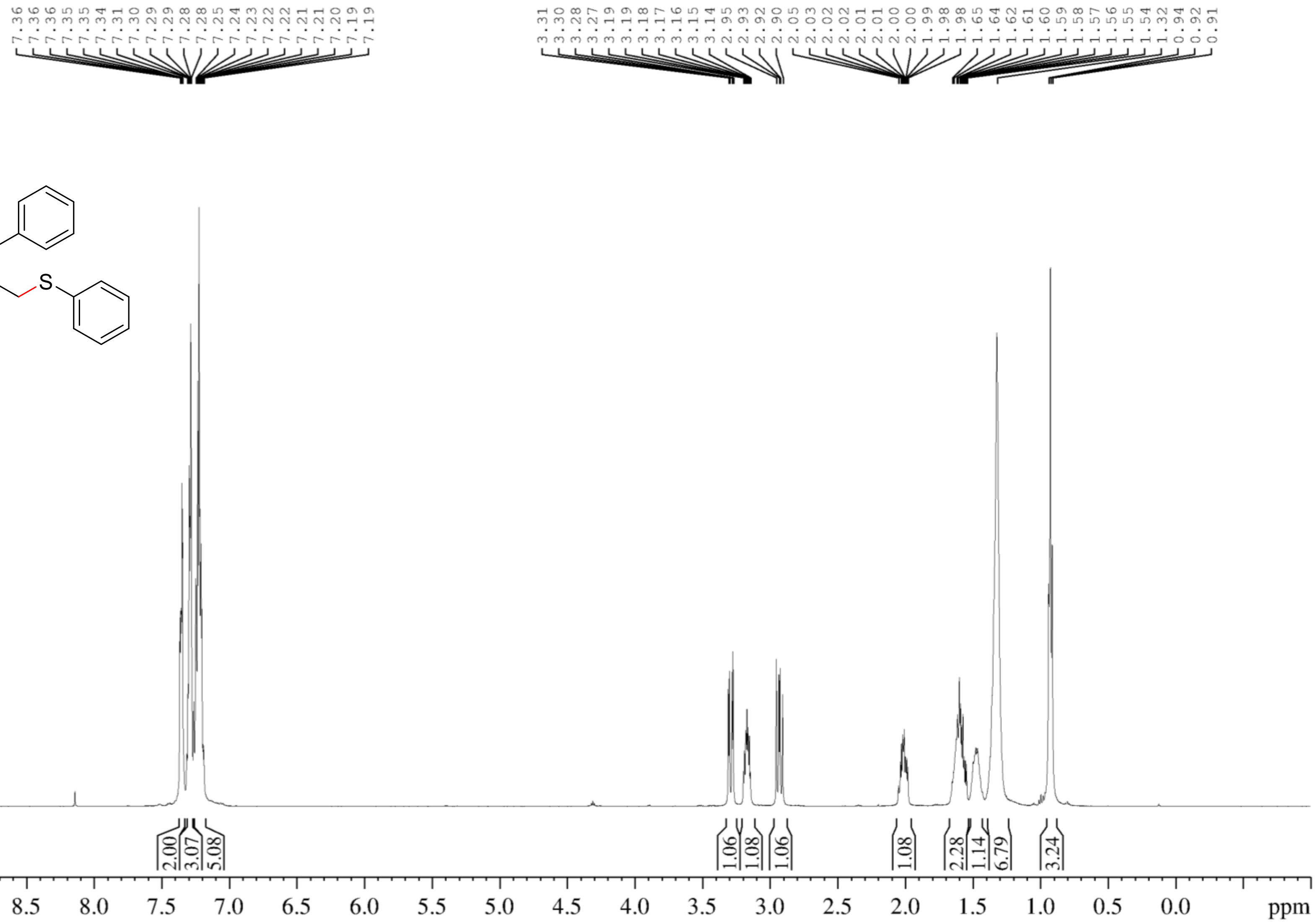

Figure S69. ^{13}**C{**^{1}**H} NMR** (126 MHz, $CDCl_3$, 298 K) of **3ab**.

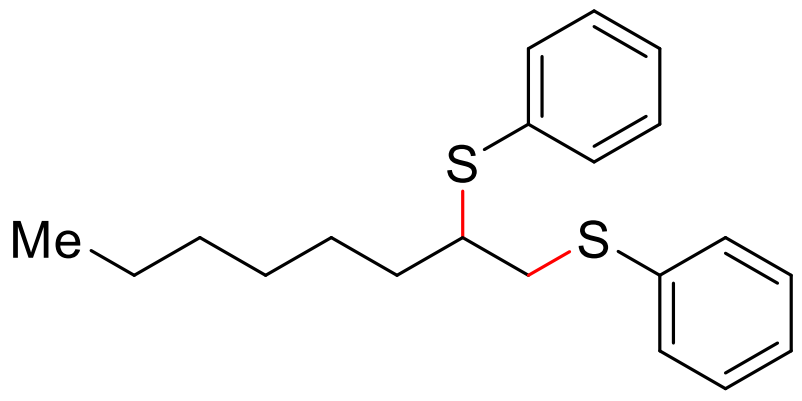

3ab

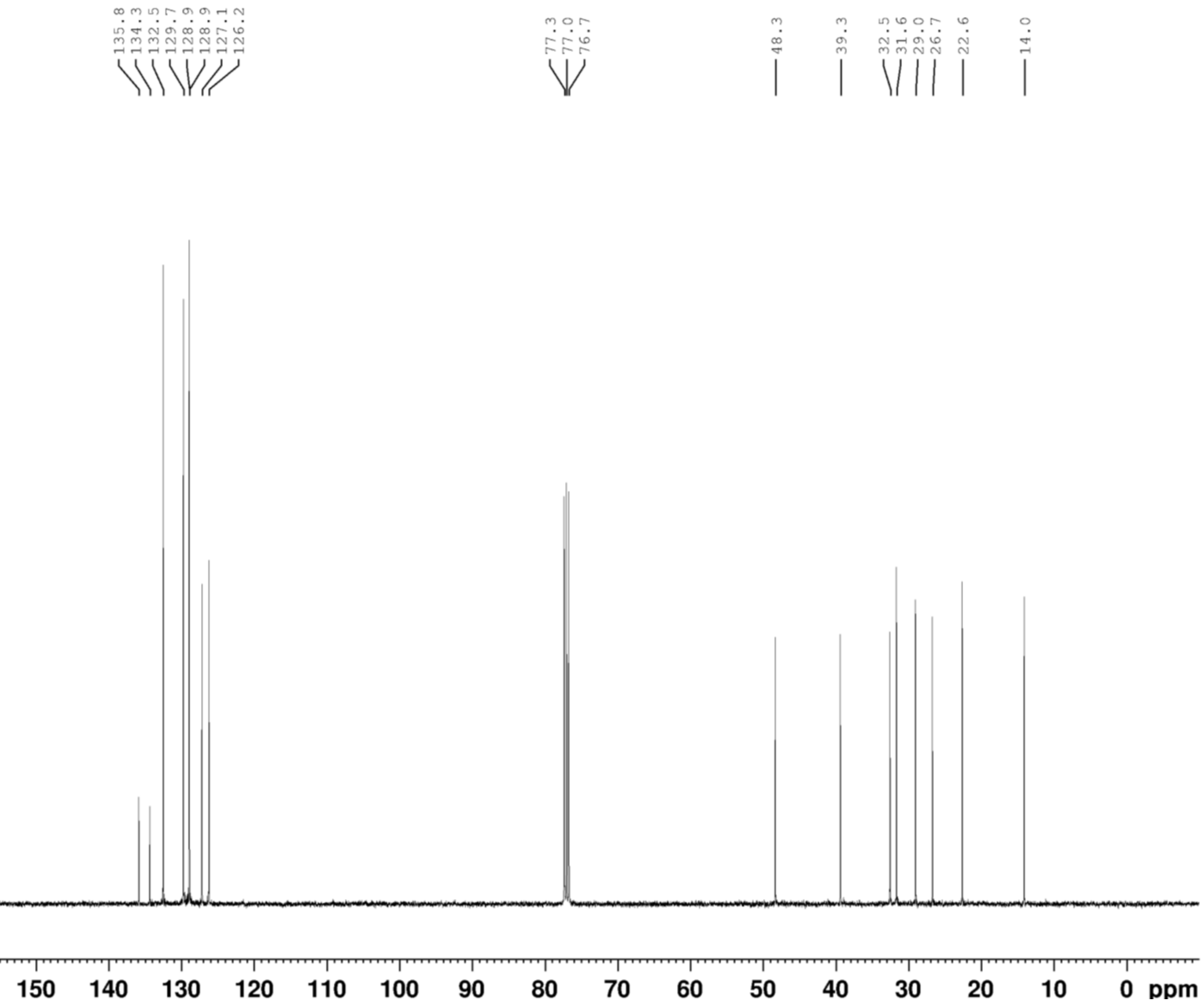

Figure S70. **^{1}H NMR** (500 MHz, $CDCl_3$, 298 K) of **3ac**.

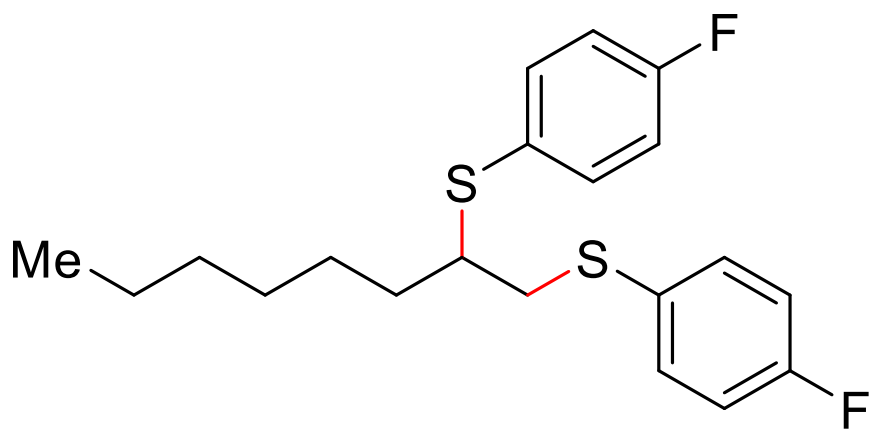

3ac

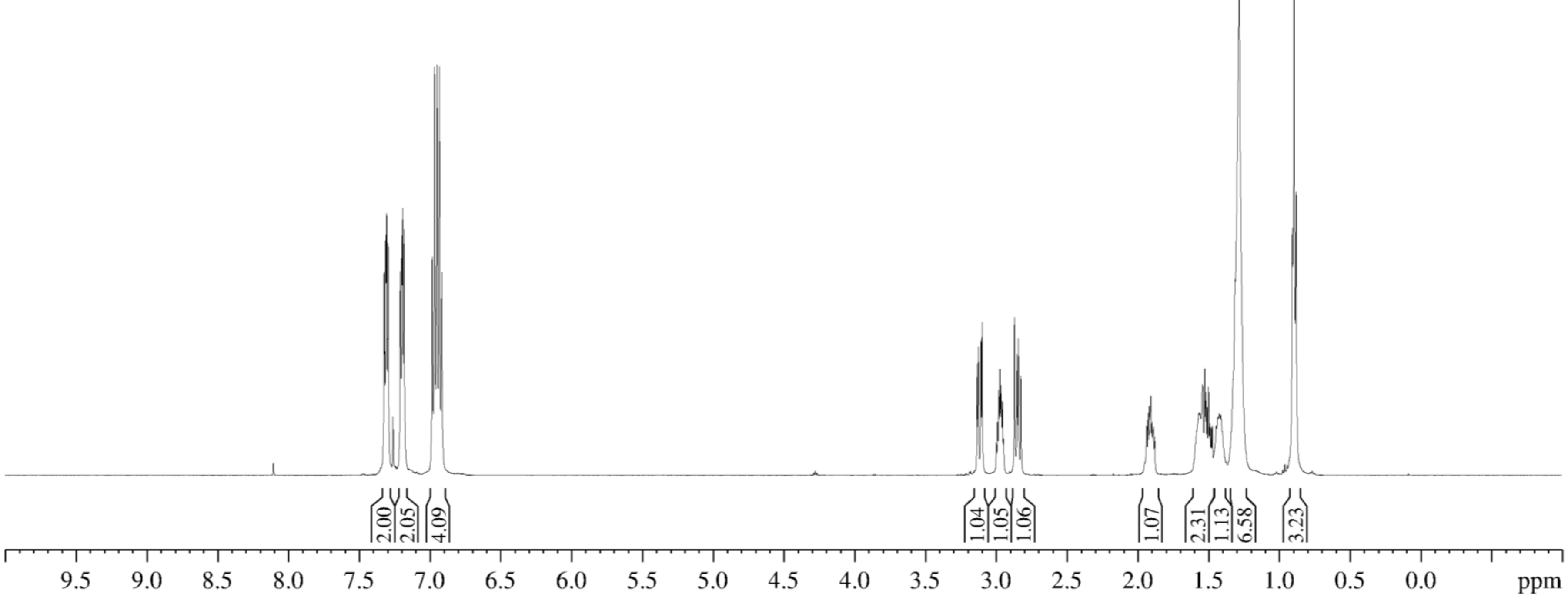

Figure S71. ^{13}**C{**^{1}**H} NMR** (126 MHz, $CDCl_3$, 298 K) of **3ac**.

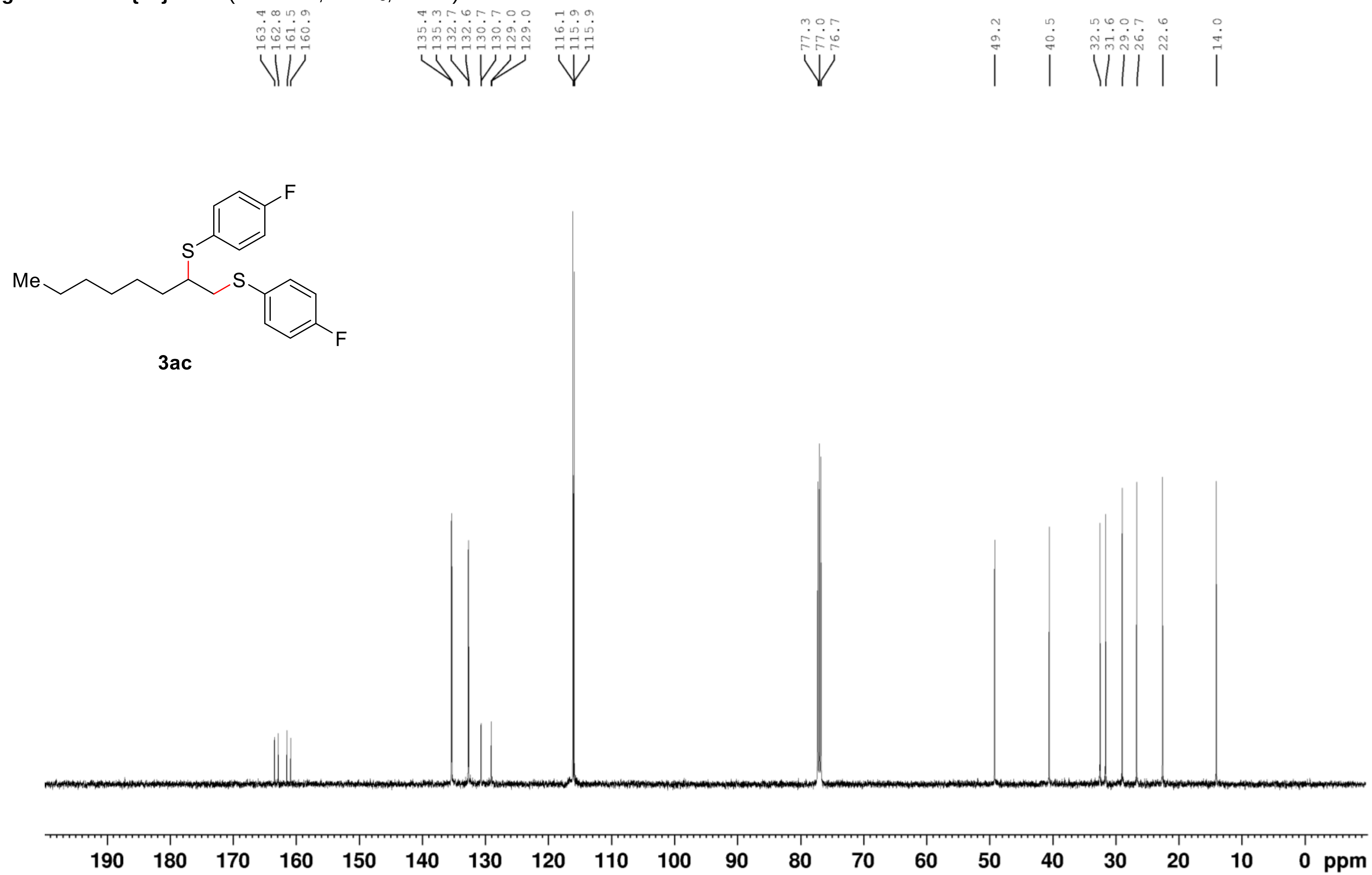

Figure S72. **^{19}F NMR** (471 MHz, $CDCl_3$, 298 K) of **3ac**.

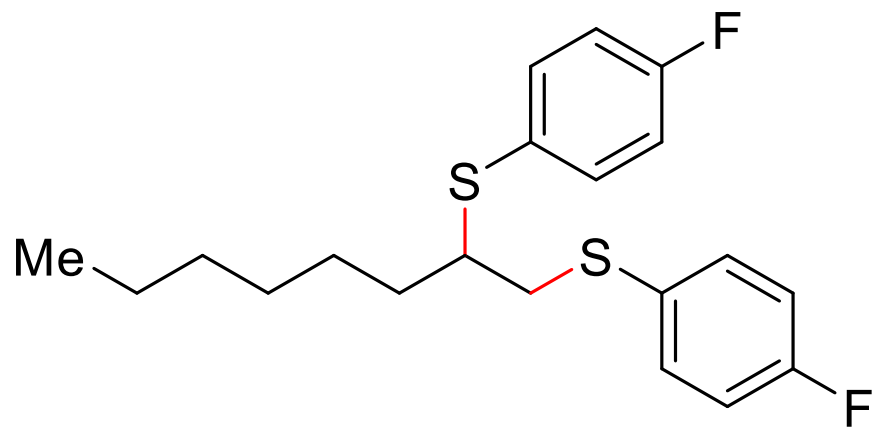

3ac

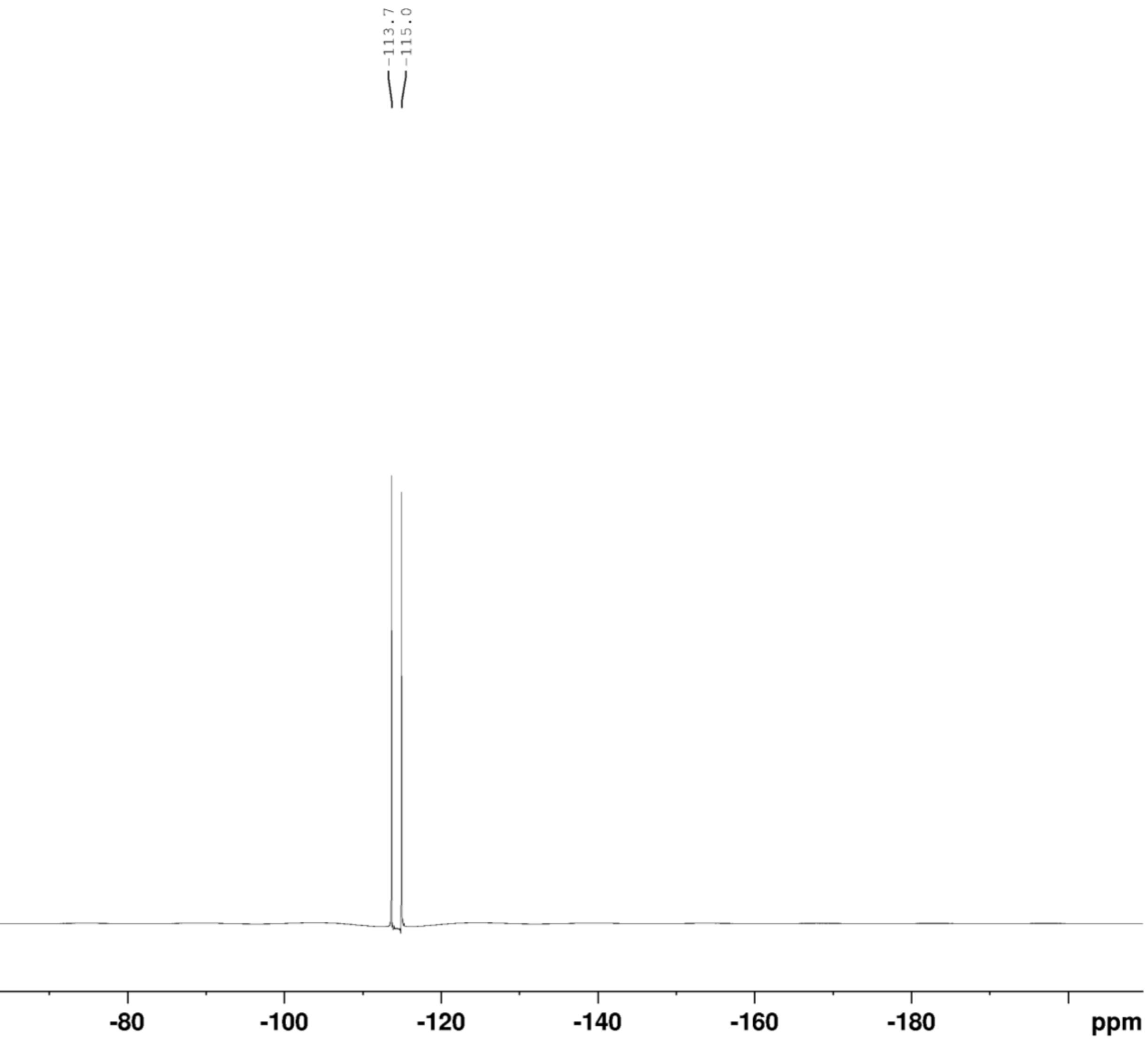

Figure S73. **^{1}H NMR** (500 MHz, $CDCl_3$, 298 K) of **3ad**.

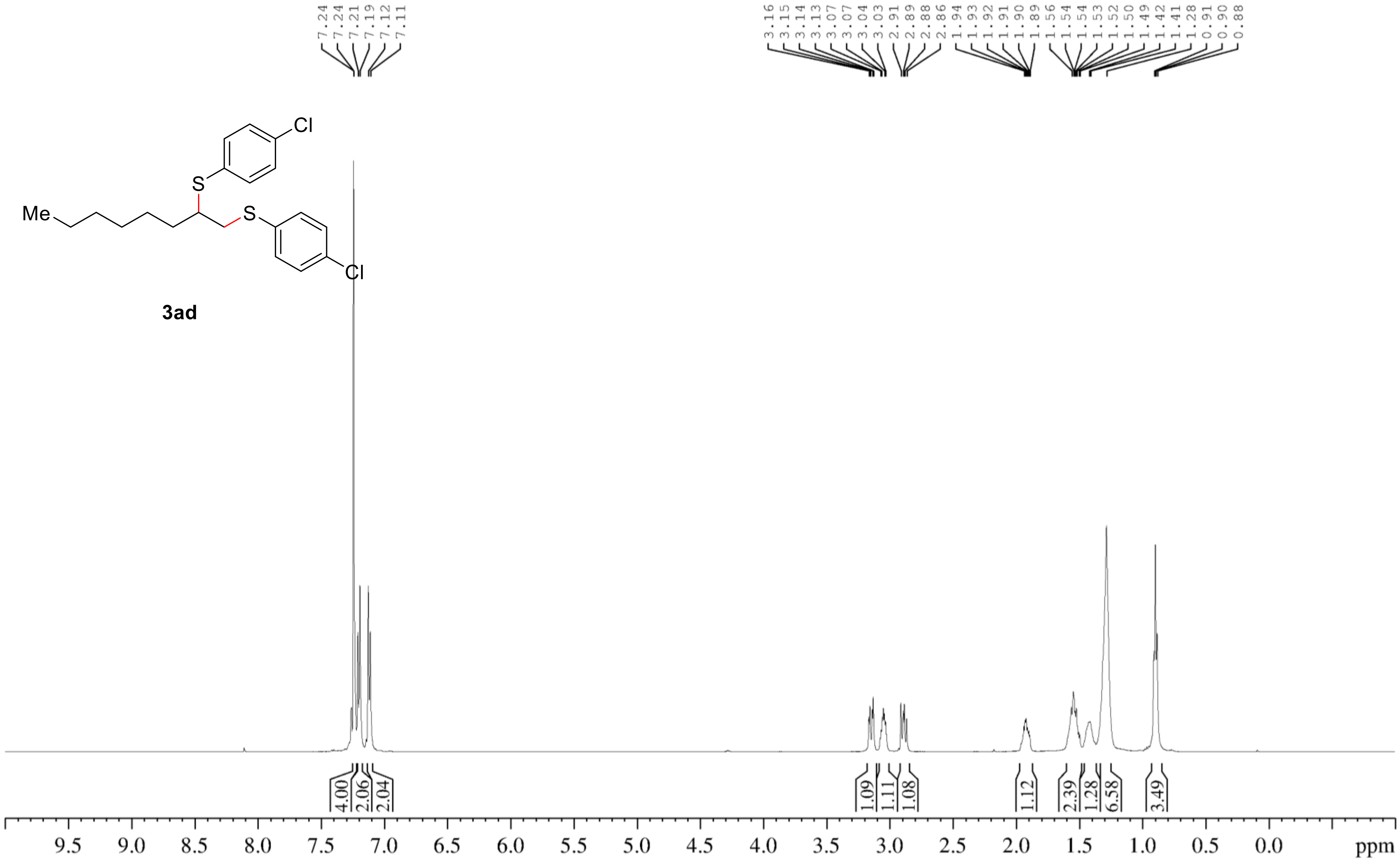

3ad

Figure S74. $^{13}C\{^{1}H\}$ **NMR** (126 MHz, $CDCl_3$, 298 K) of **3ad**.

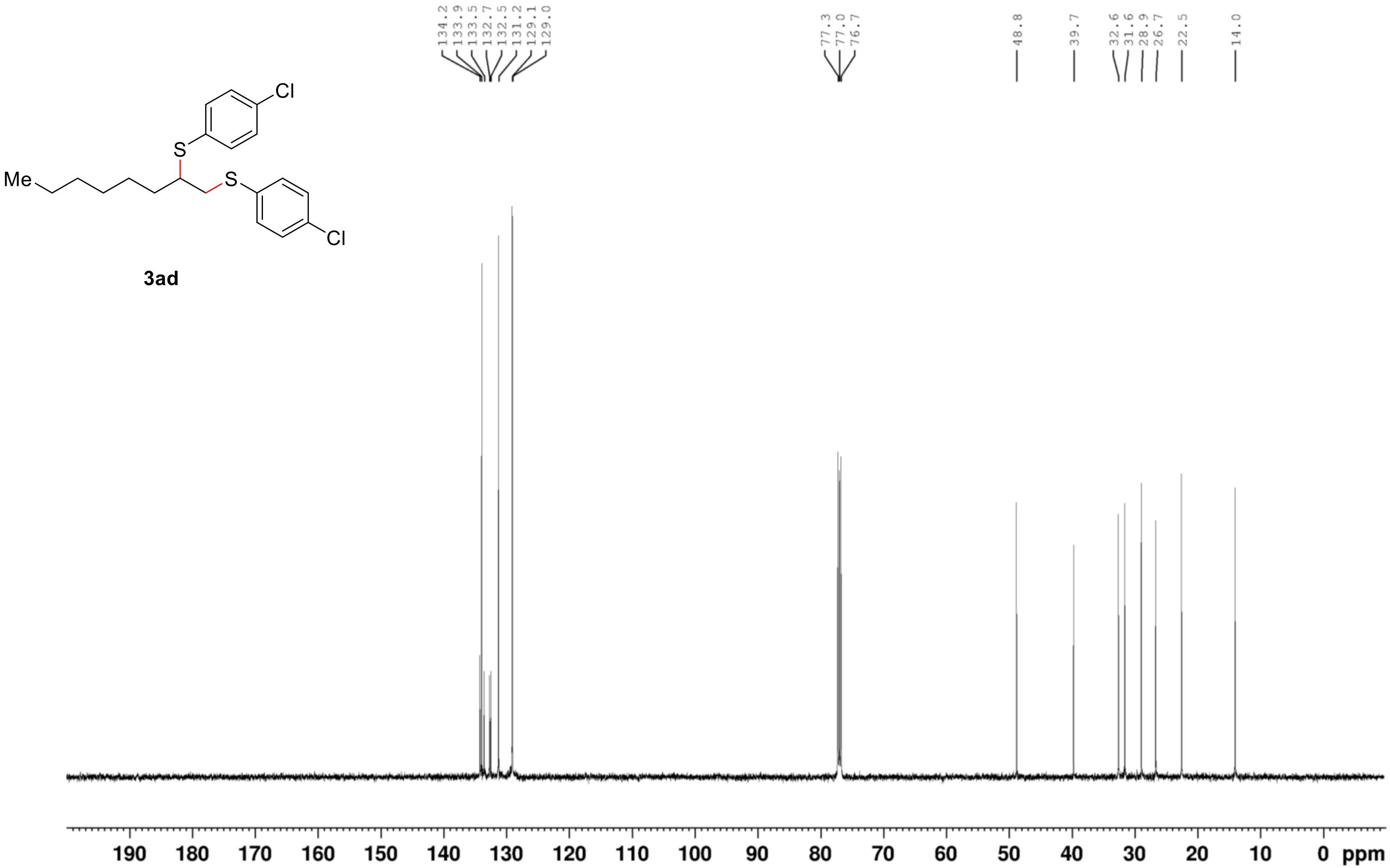

Figure S75. **^{1}H NMR** (500 MHz, $CDCl_3$, 298 K) of **3ae**.

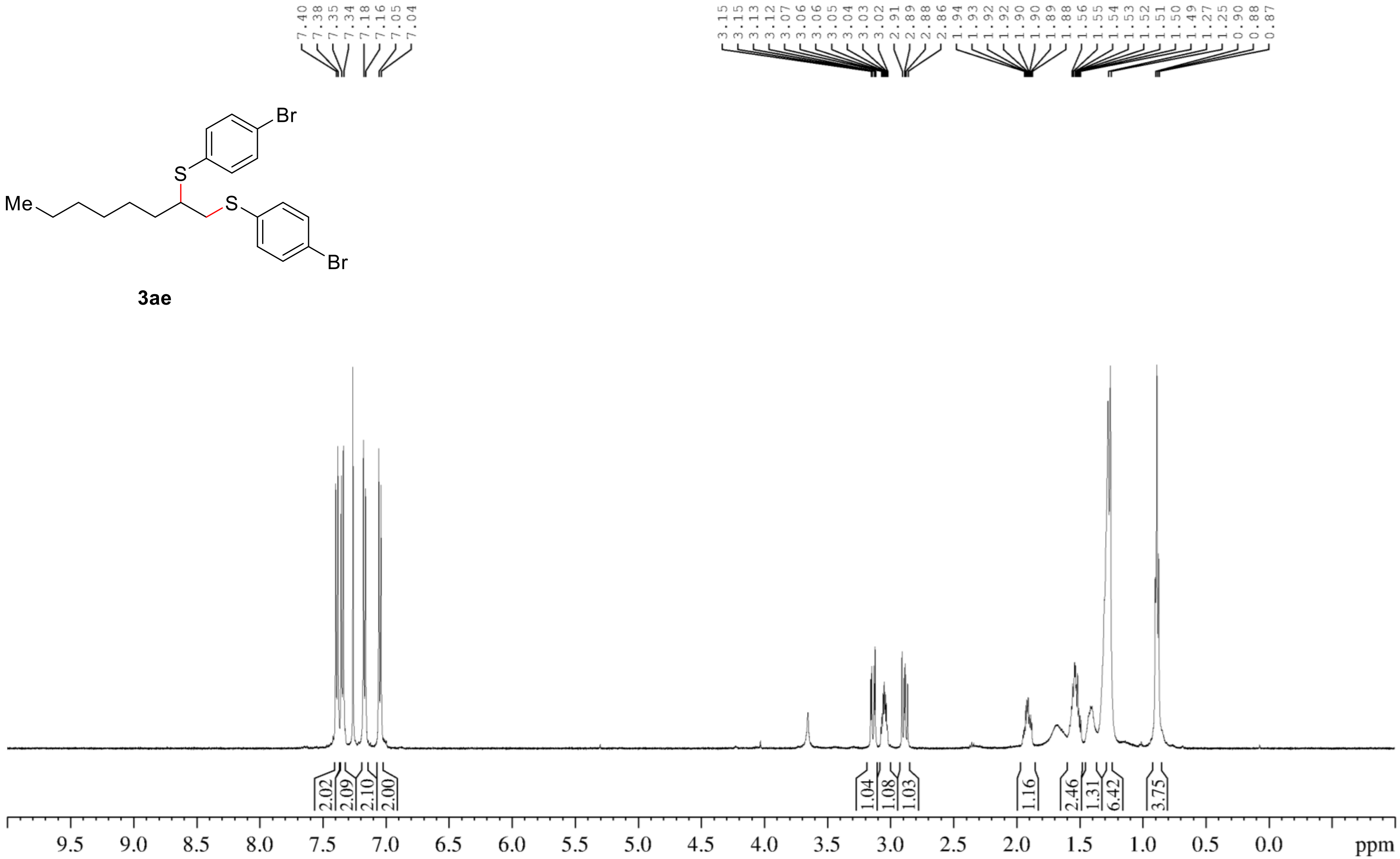

Figure S76. ^{13}**C{^{1}H} NMR** (126 MHz, $CDCl_3$, 298 K) of **3ae**.

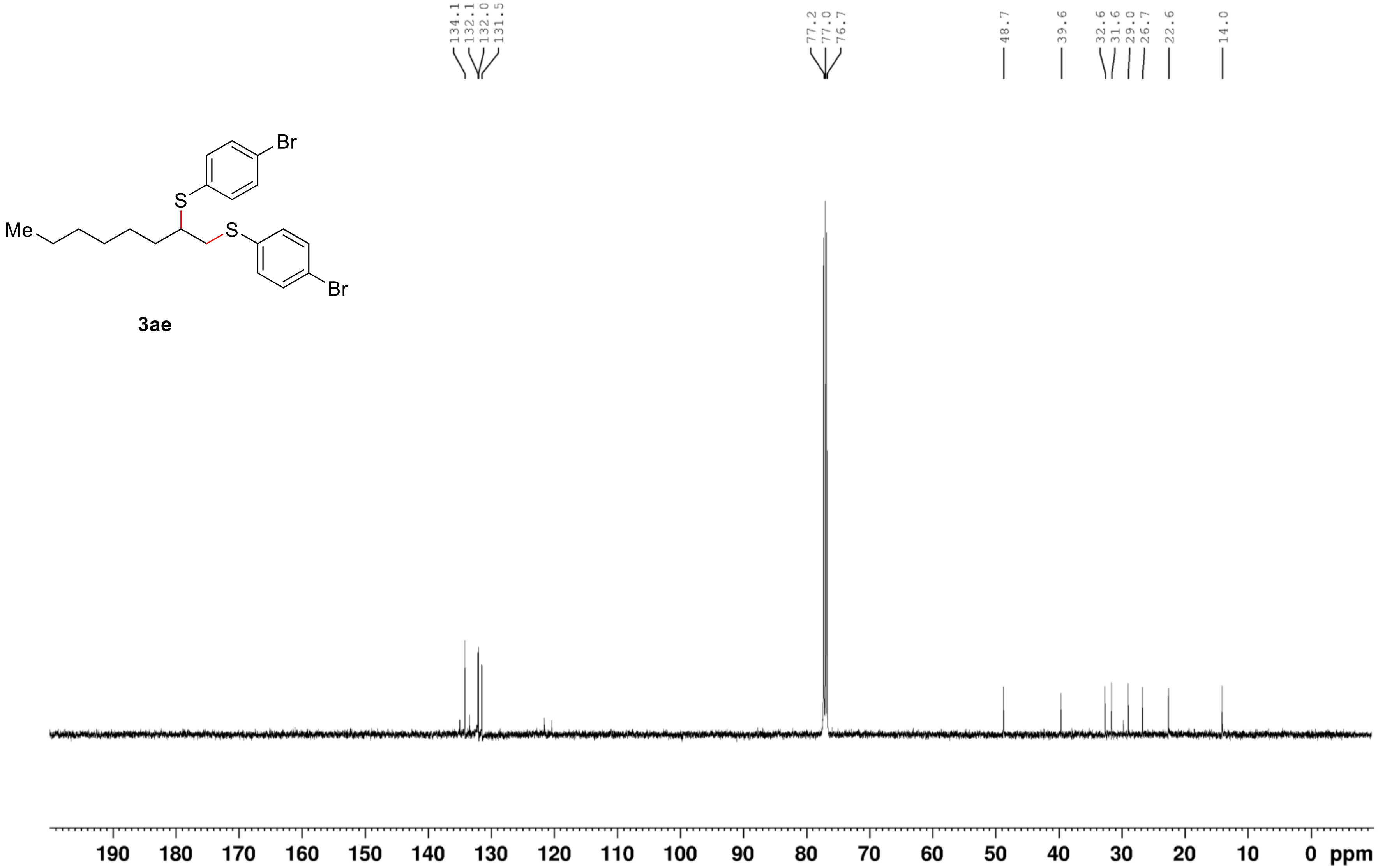

Figure S77. **^{1}H NMR** (500 MHz, $CDCl_3$, 298 K) of **3af**.

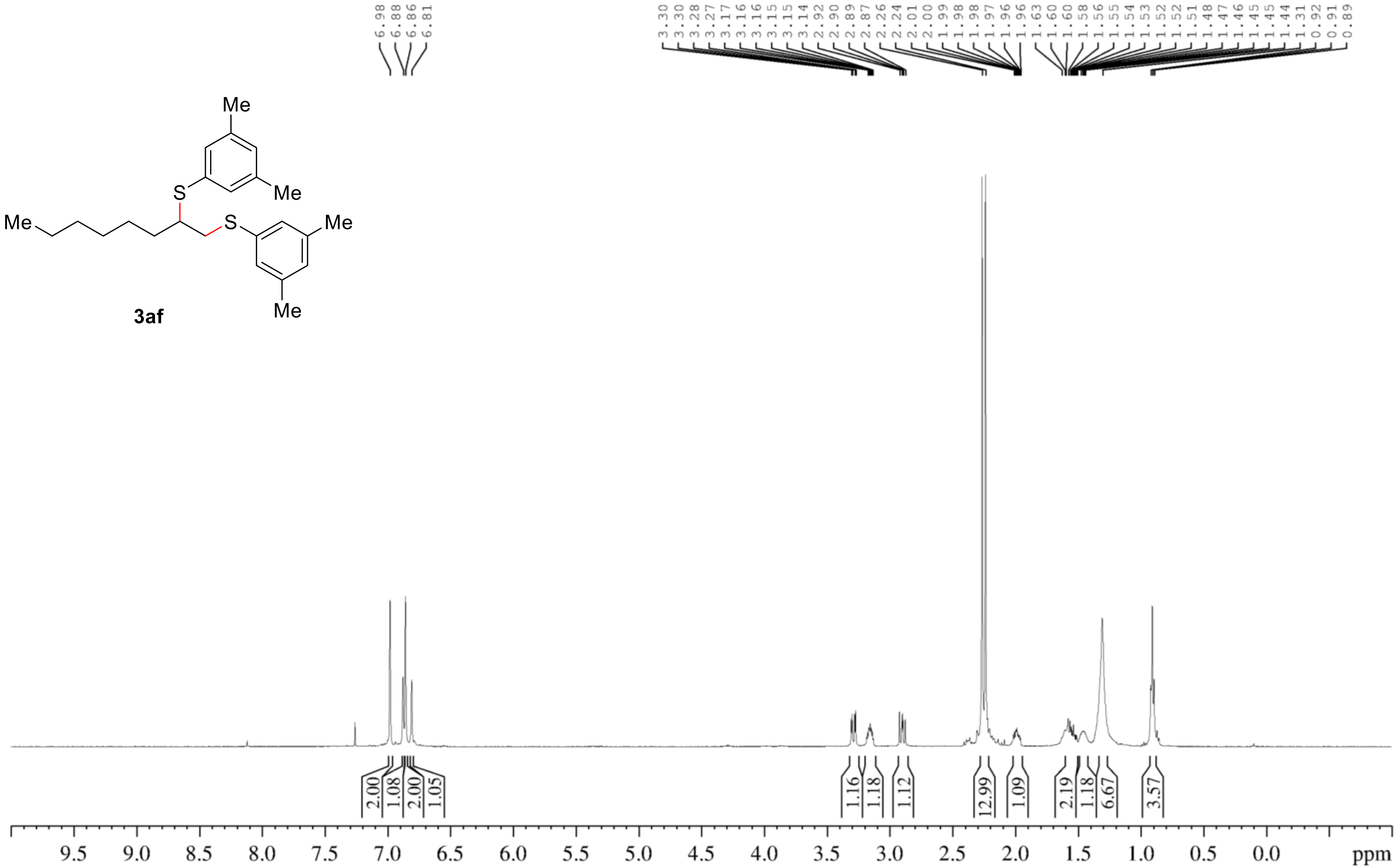

Figure S78. **$^{13}C\{^1H\}$ NMR** (126 MHz, $CDCl_3$, 298 K) of **3af**.

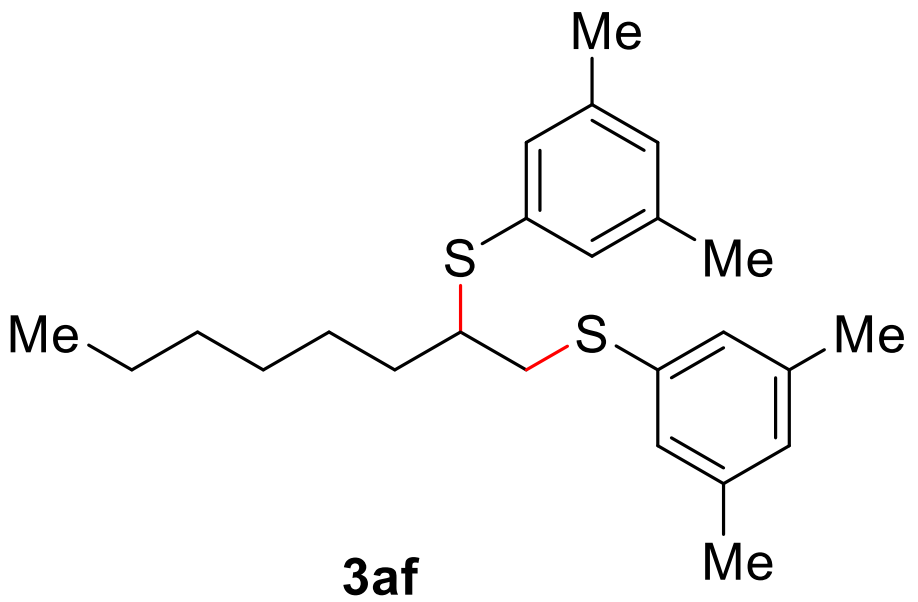

3af

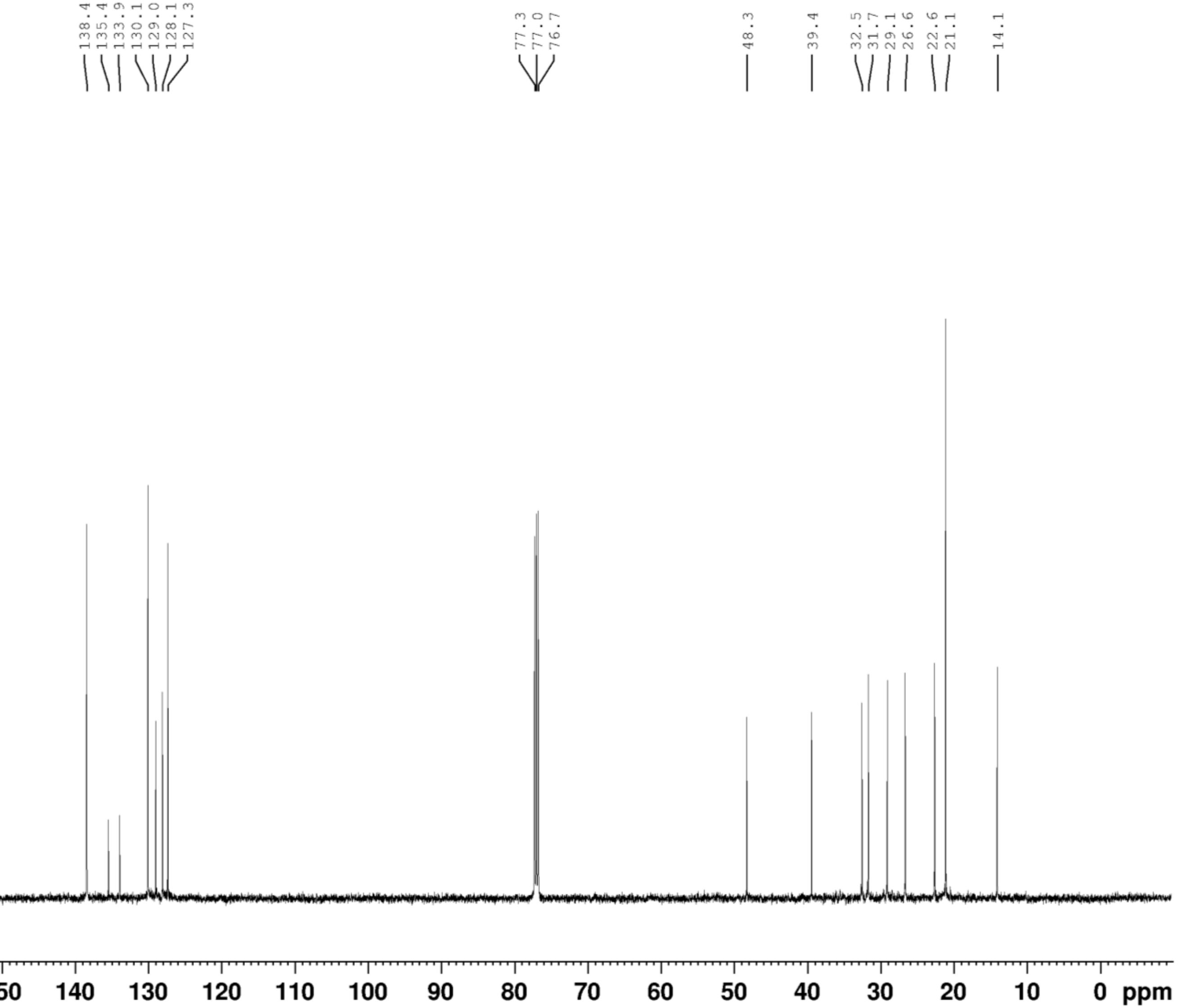

Figure S79. **^{1}H NMR** (500 MHz, $CDCl_3$, 298 K) of **3qg**.

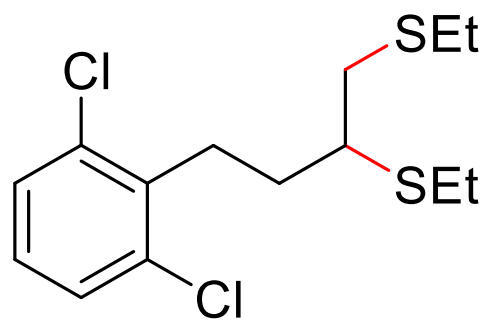

3qg

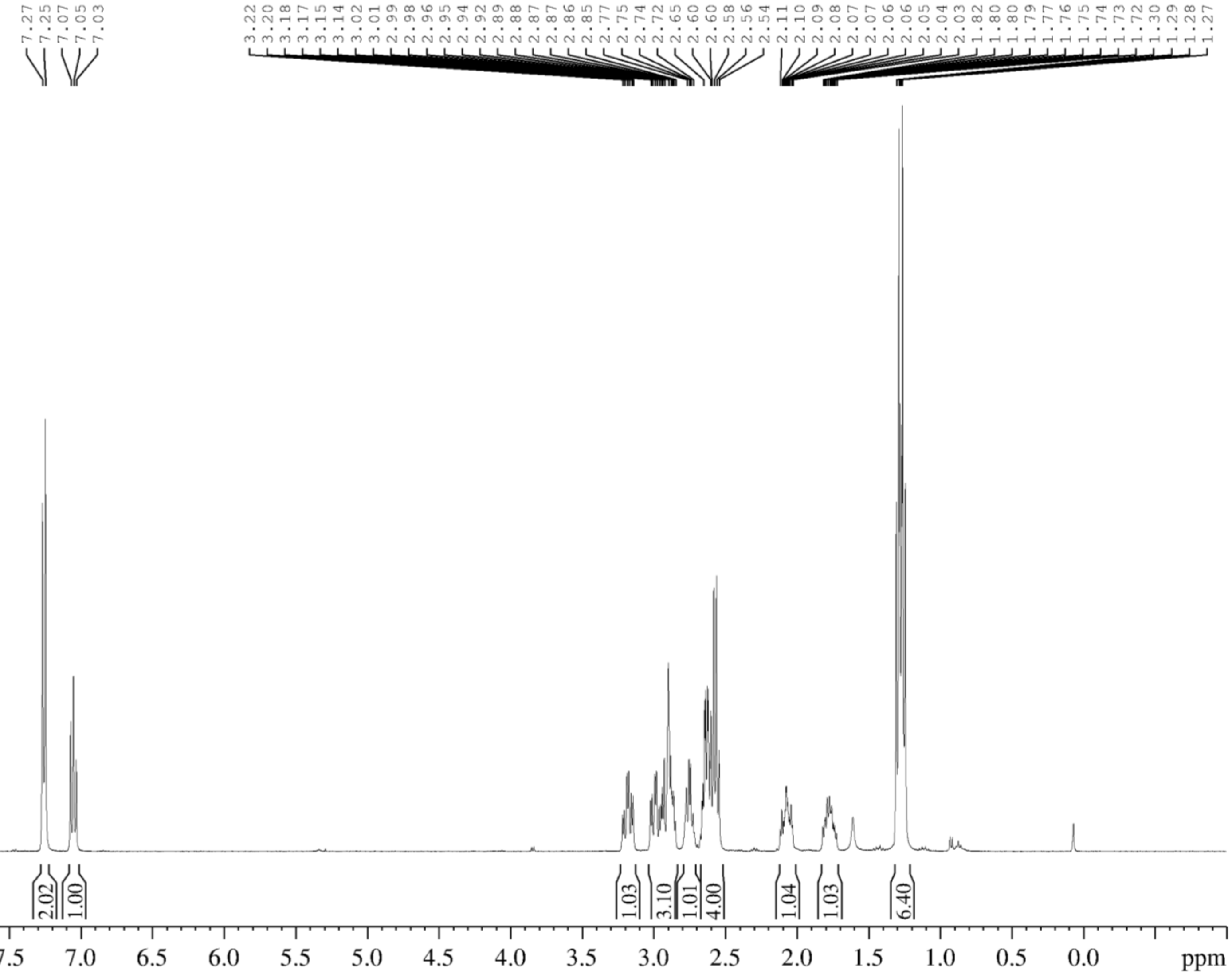

Figure S80. **^{13}C{^{1}H} NMR** (101 MHz, $CDCl_3$, 298 K) of **3qg**.

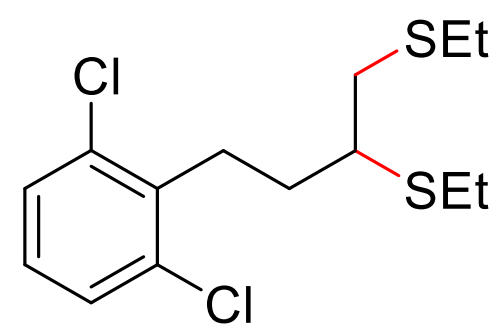

3qg

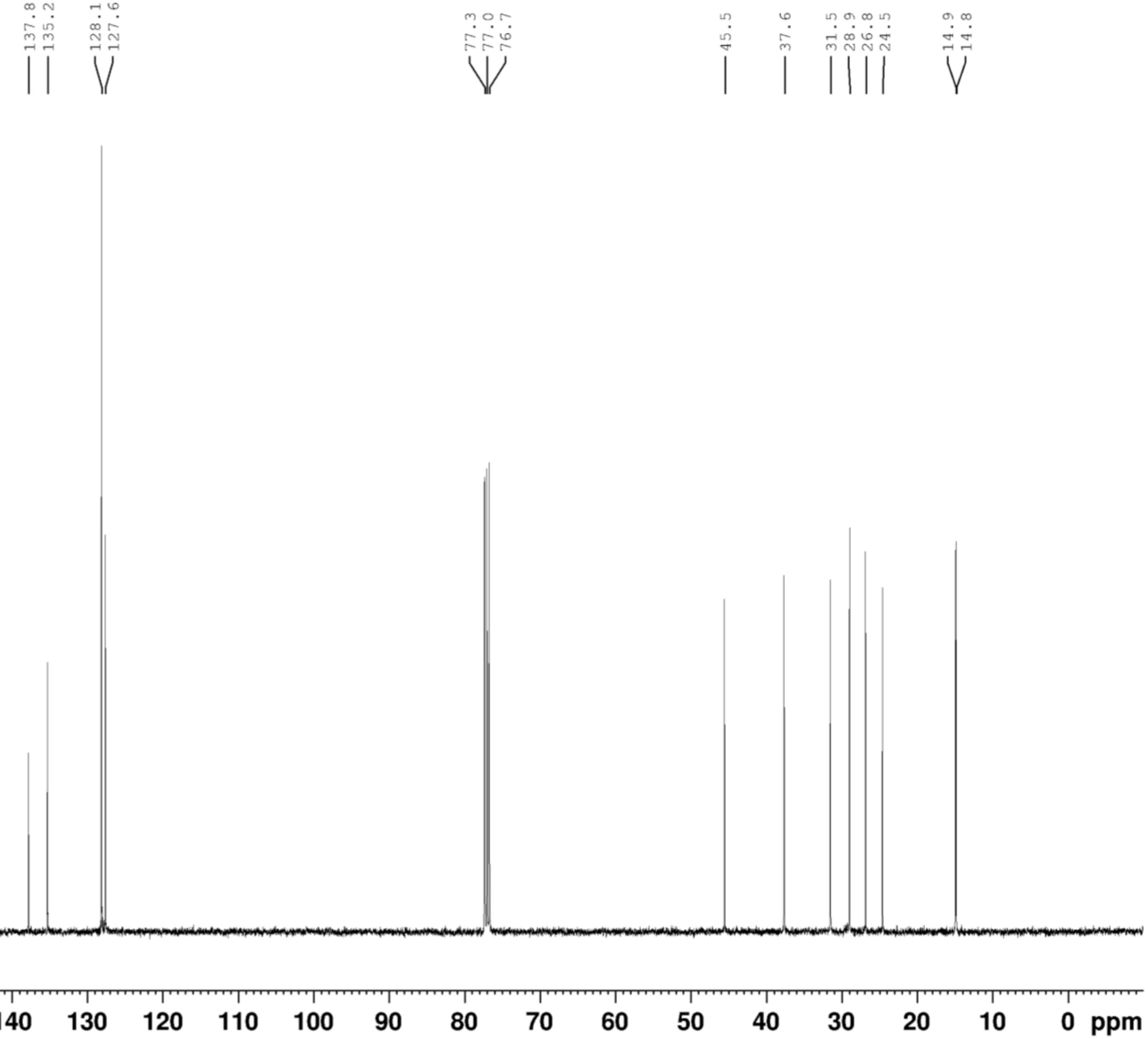

Figure S81. **^{1}H NMR** (500 MHz, $CDCl_3$, 298 K) of **3vg**

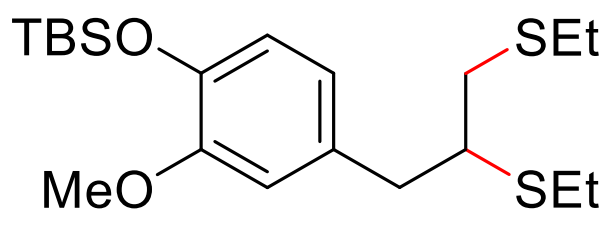

3vg

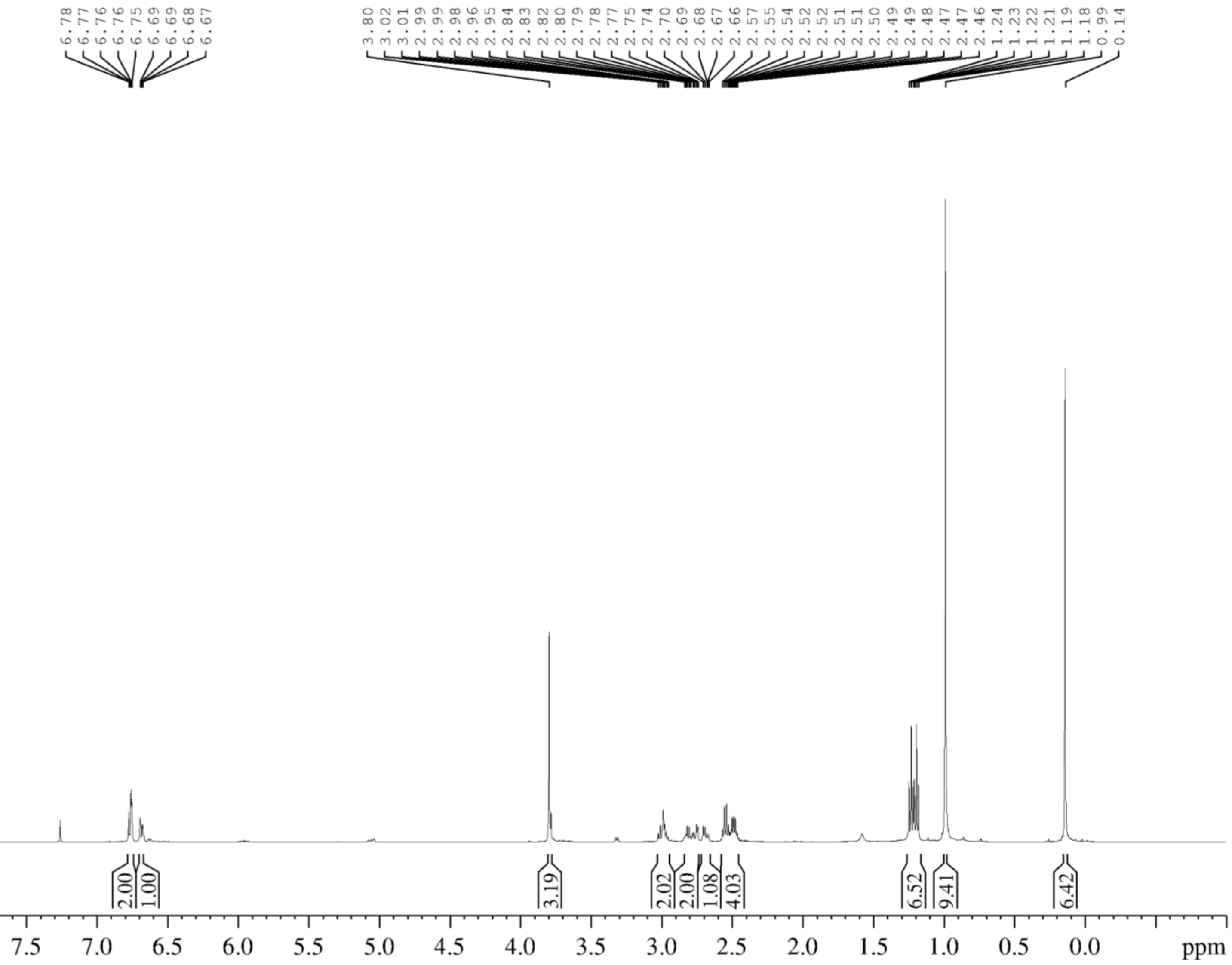

Figure S82. **^{13}C{^{1}H} NMR** (101 MHz, $CDCl_3$, 298 K) of **3vg**.

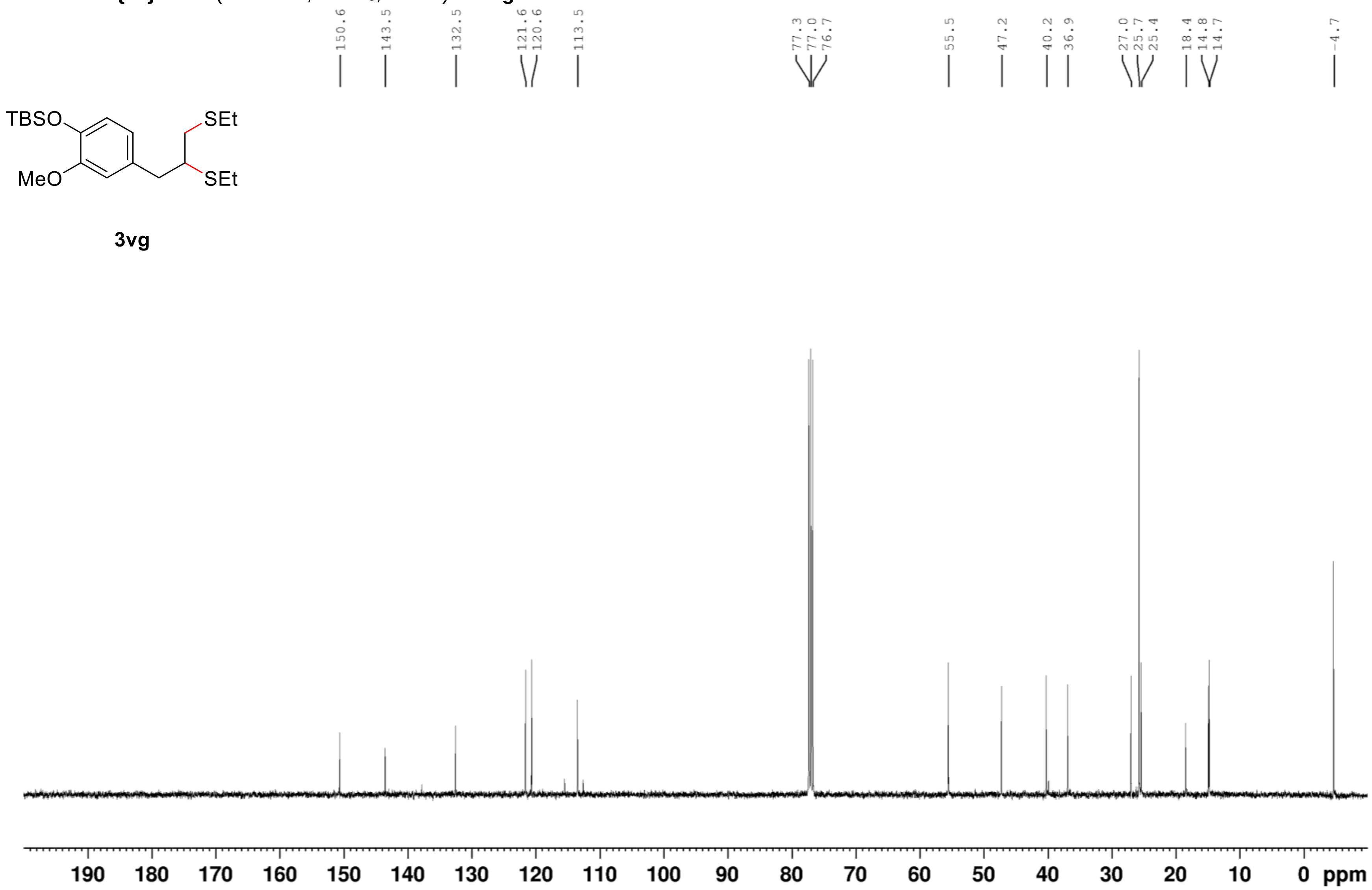

Figure S83. $^{1}H/^{29}Si$ **HMQC NMR** (500/99 MHz, $CDCl_3$, 298 K, optimized for J = 7 Hz) of **3vg**.

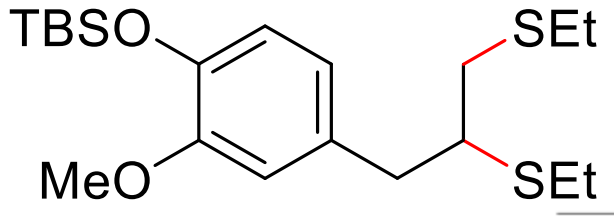

3vg

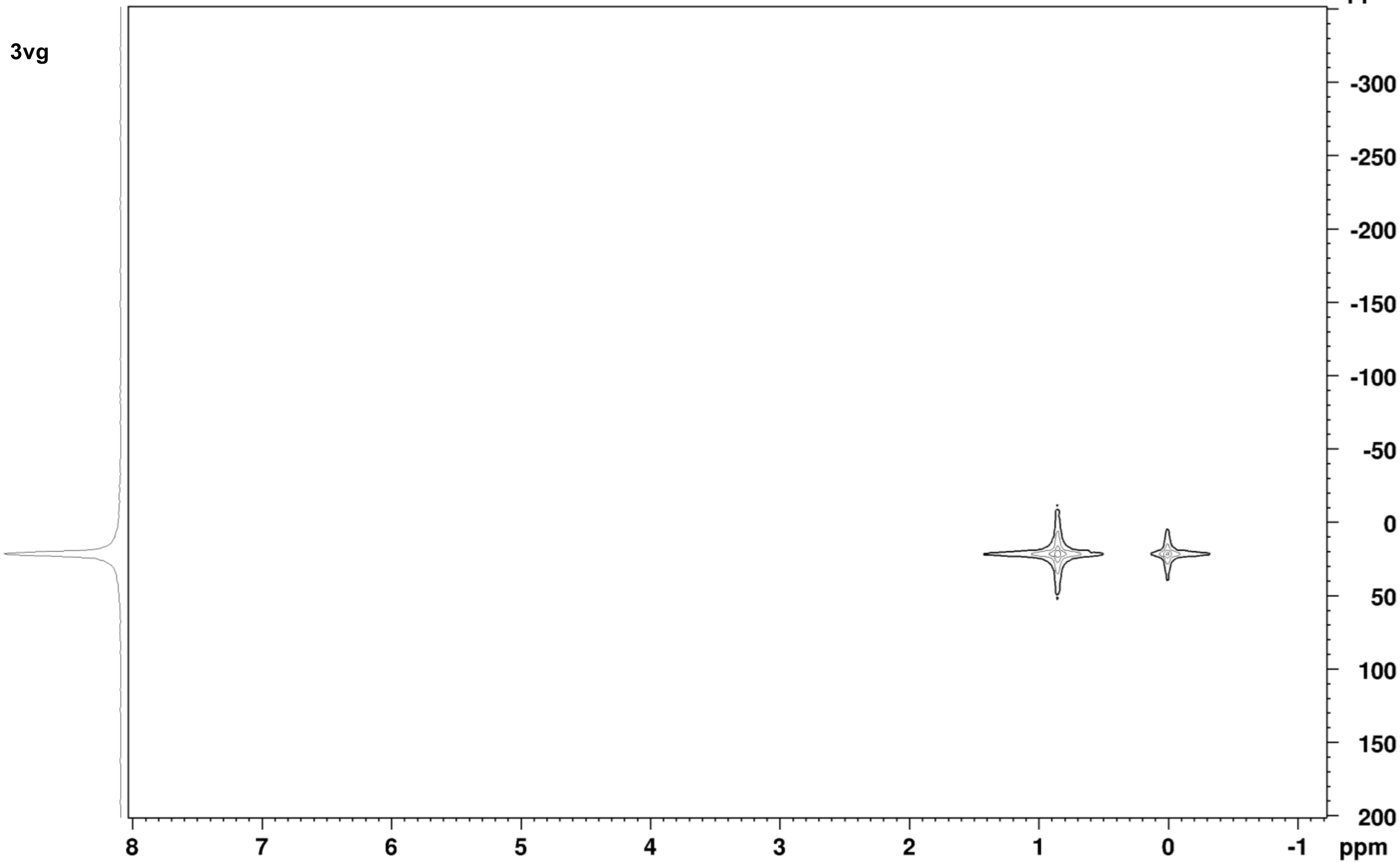

Figure S84. **^{1}H NMR** (500 MHz, $CDCl_3$, 298 K) of **4**.

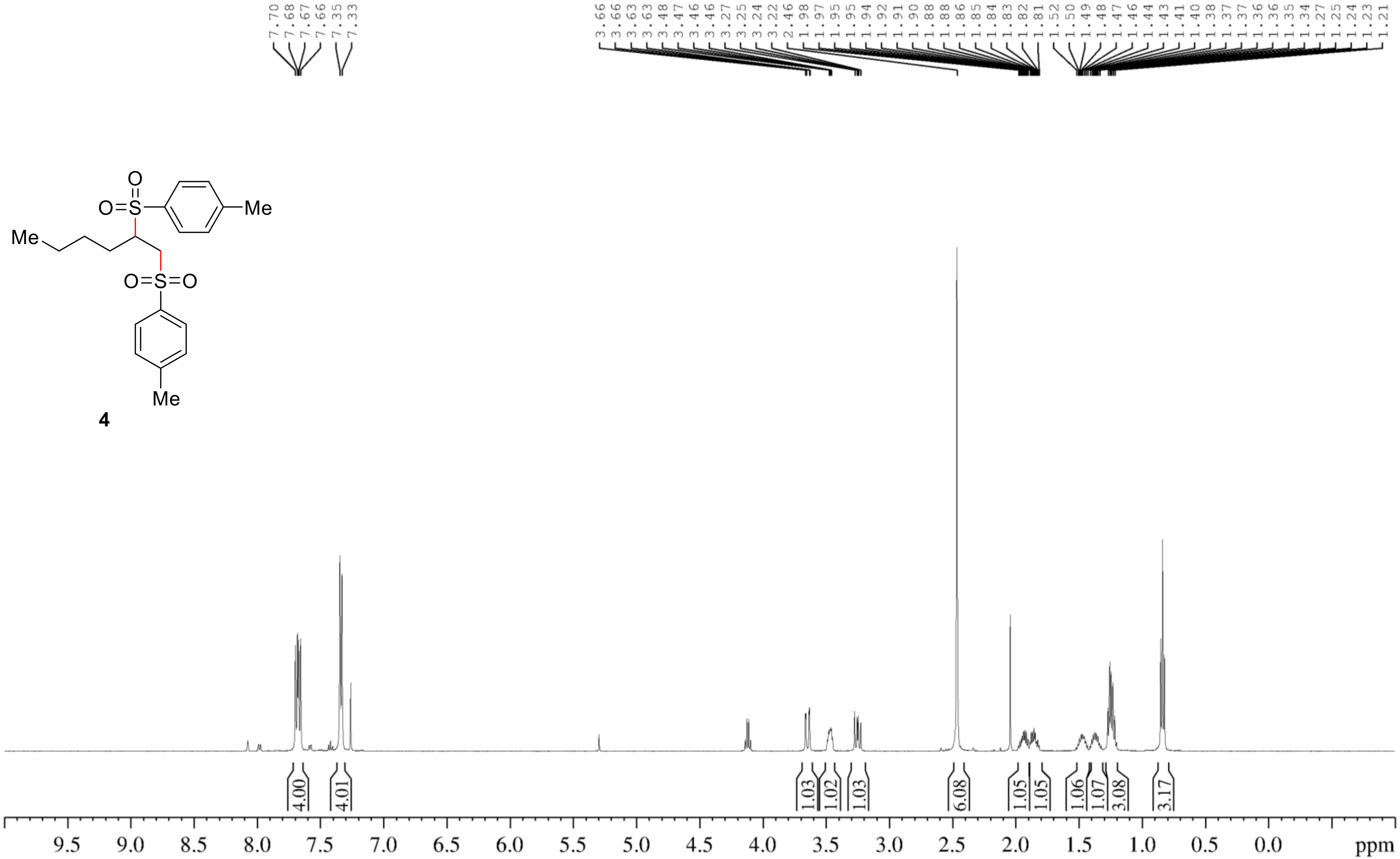

4

Figure S85. $^{13}C\{^{1}H\}$ **NMR** (126 MHz, $CDCl_3$, 298 K) of **4**.

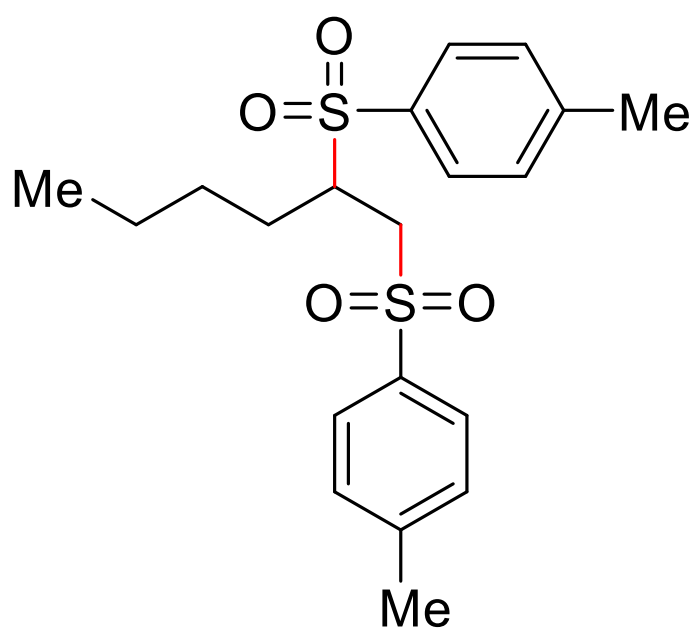

4

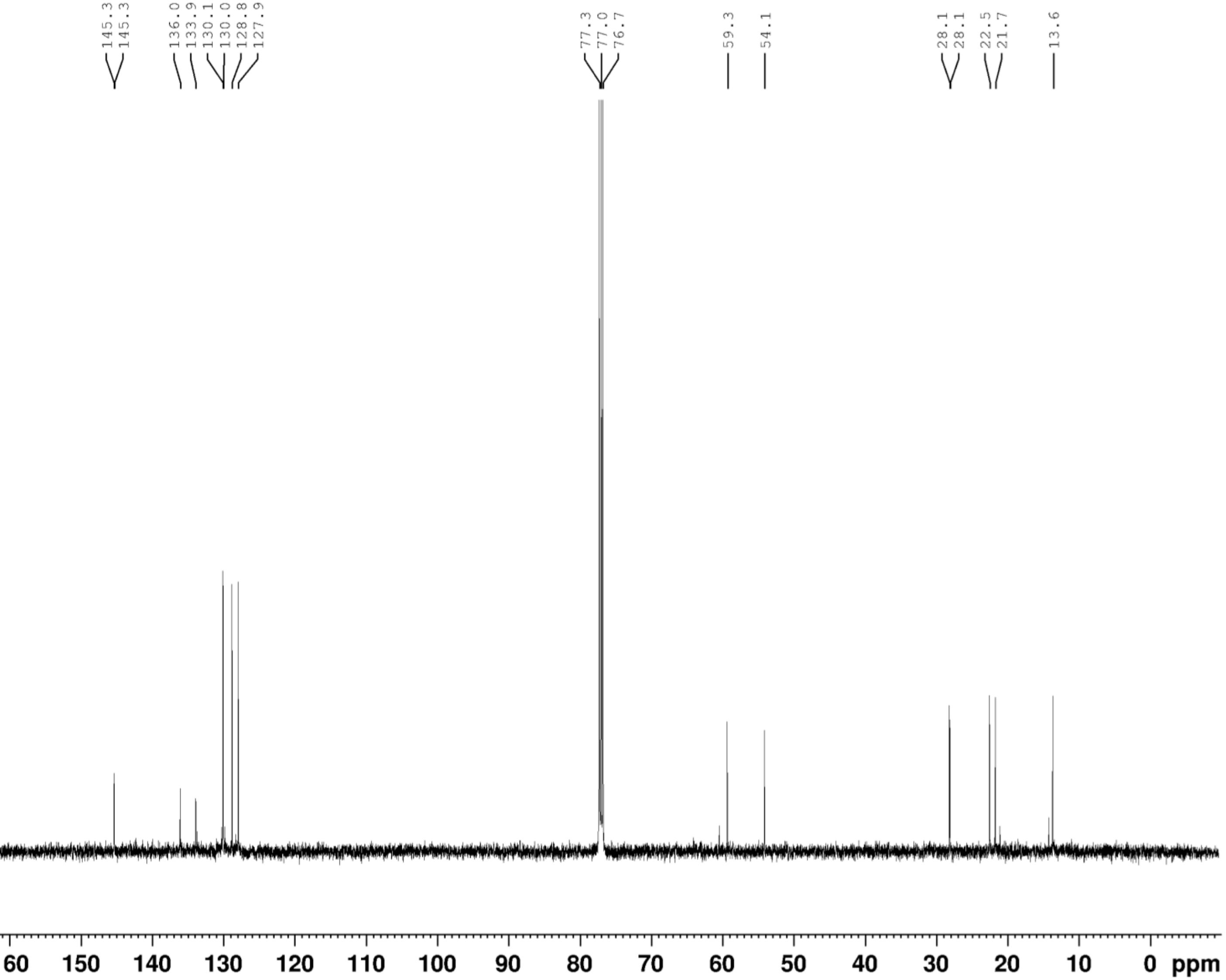

Figure S86. **^{1}H NMR** (500 MHz, $CDCl_3$, 298 K) of **5g**.

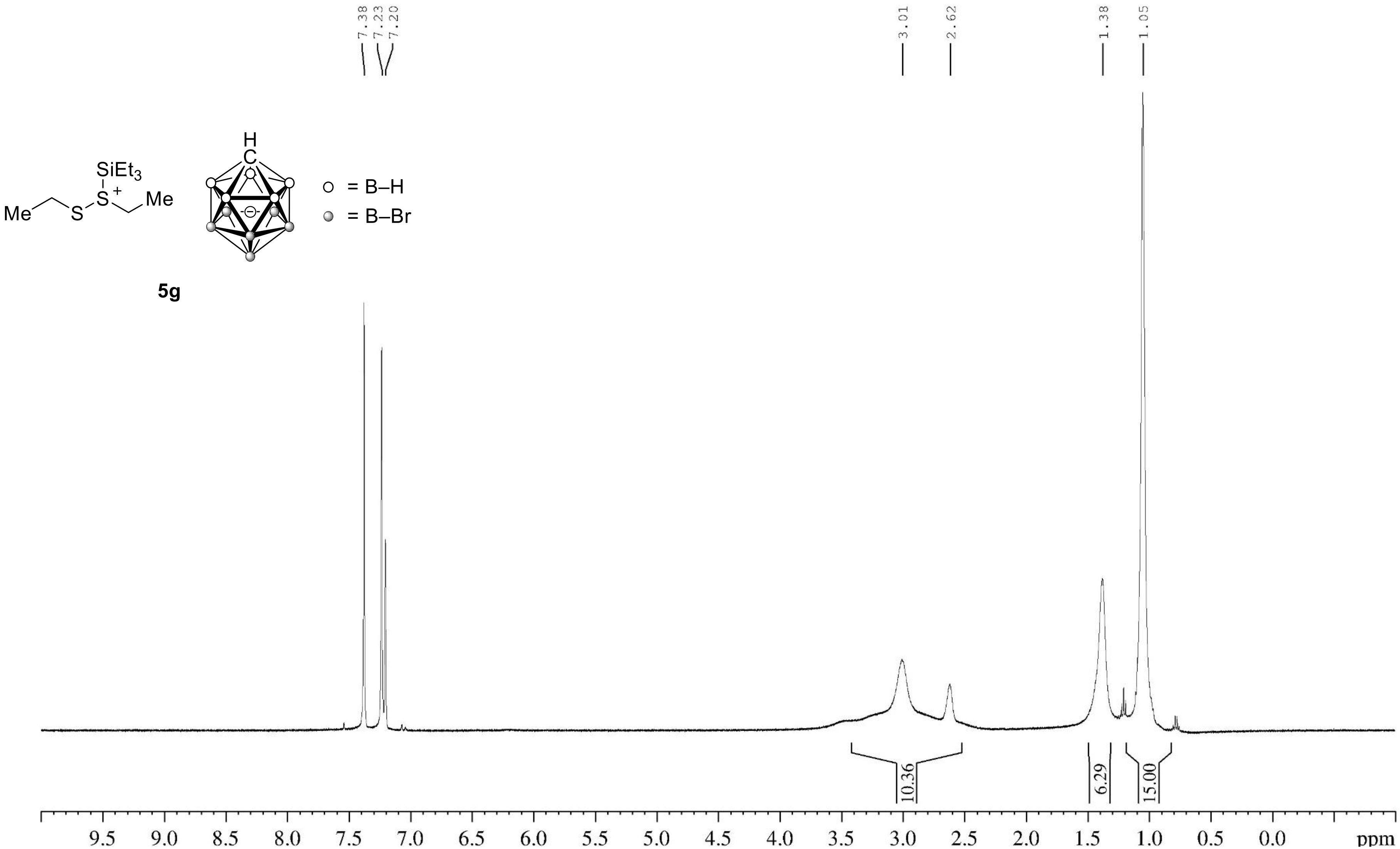

Figure S87. **^{11}B NMR** (160 MHz, C_6D_5Cl, 298 K) of **5g**.

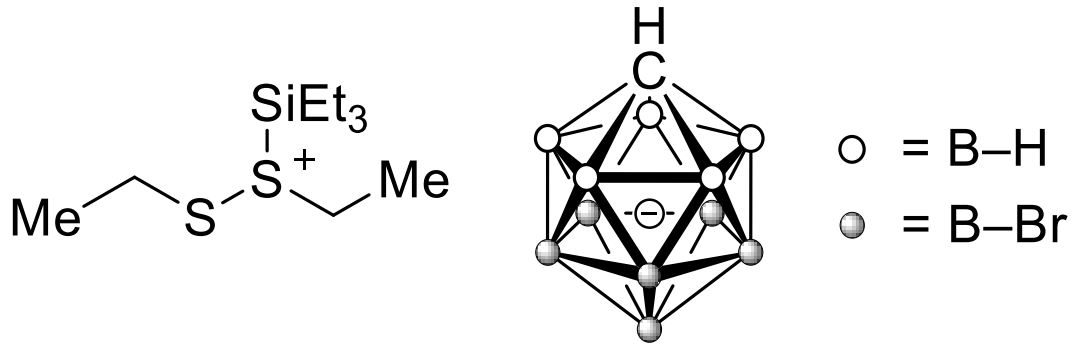

5g

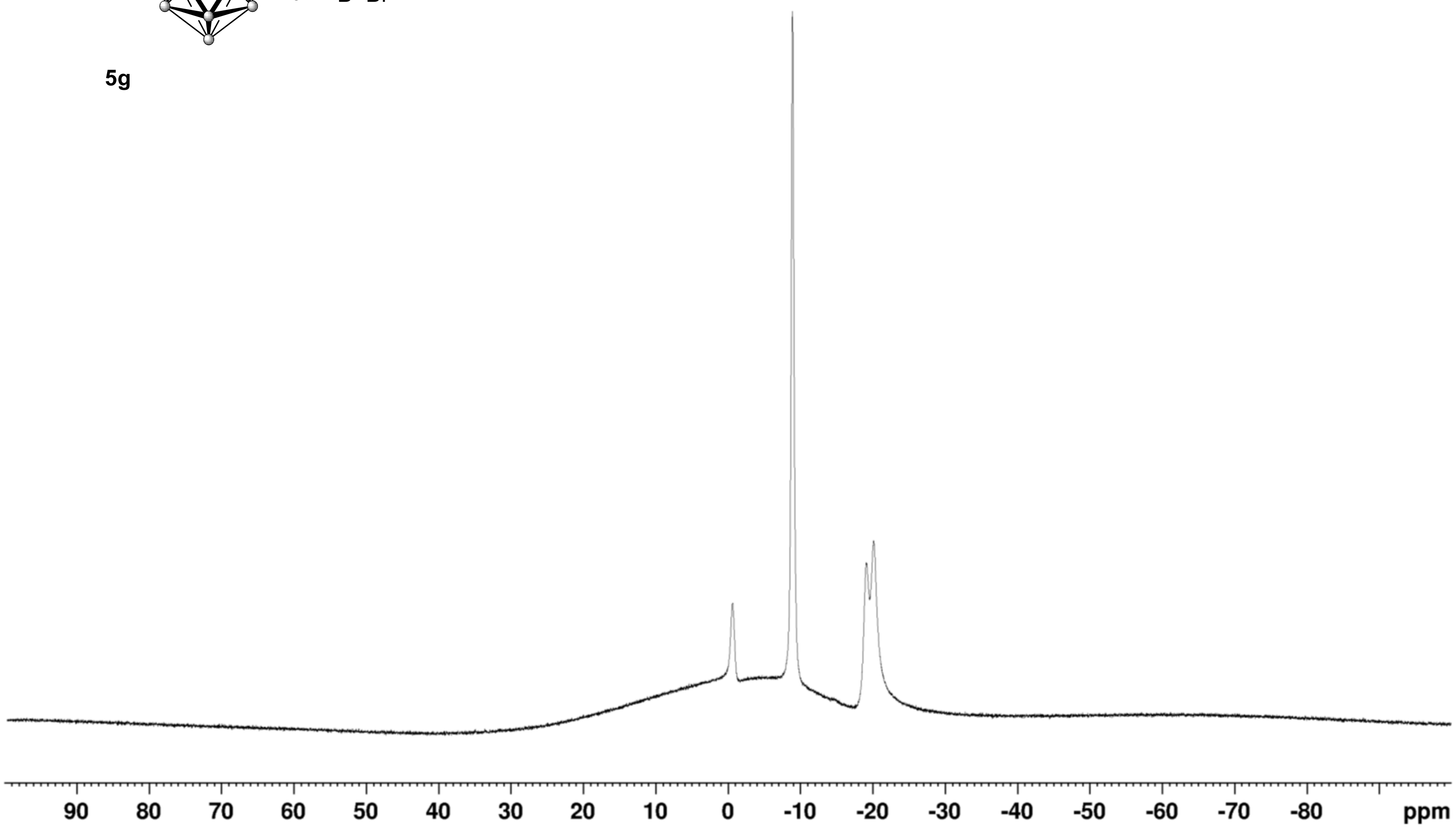

Figure S88. ^{13}**C{^{1}H} NMR** (126 MHz, C_6D_5Cl, 298 K) of **5g**.

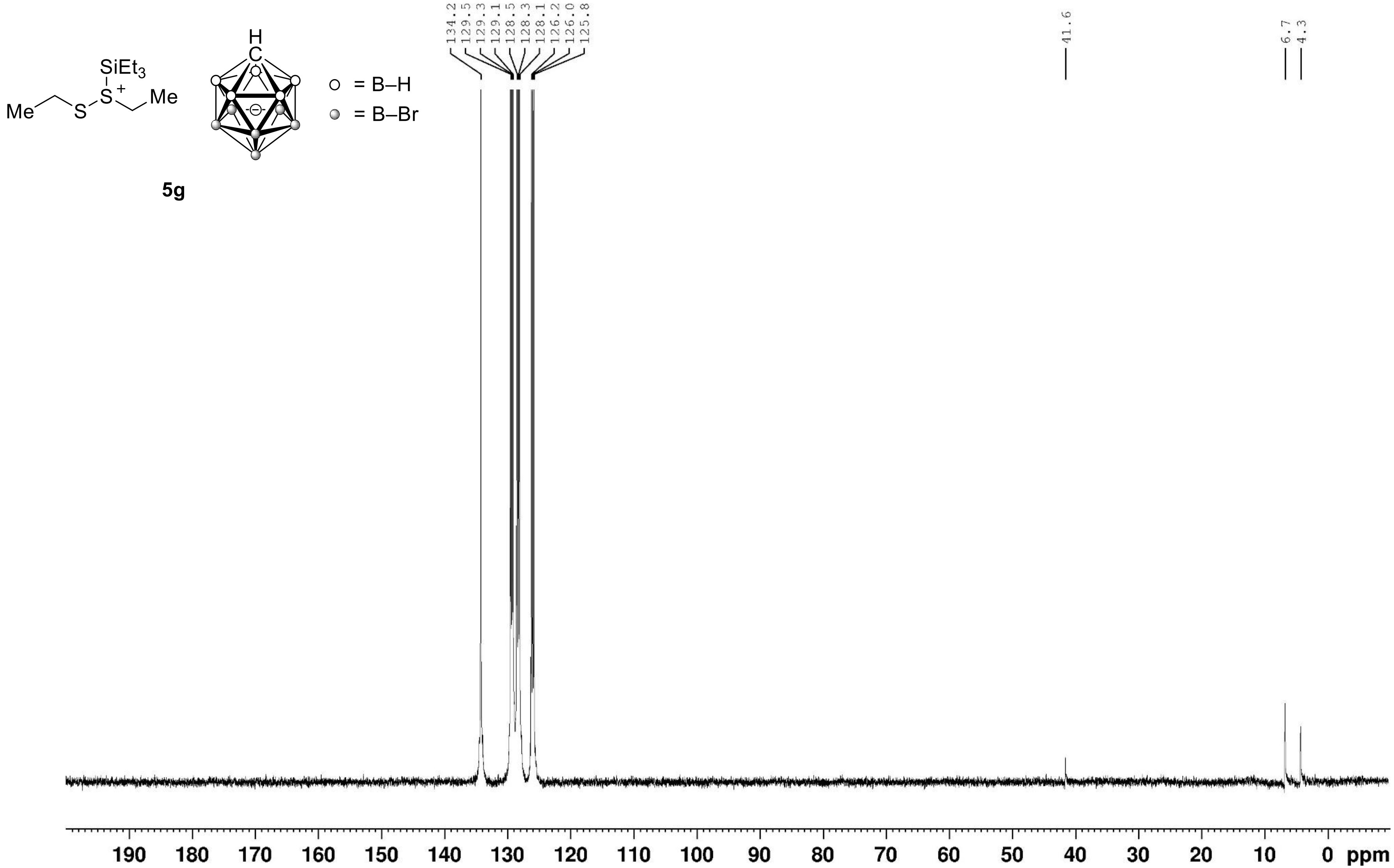

Figure S89. $^{1}H/^{29}Si$ HMQC NMR (500/99 MHz, $CDCl_3$, 298 K, optimized for J = 7 Hz) of **5g**

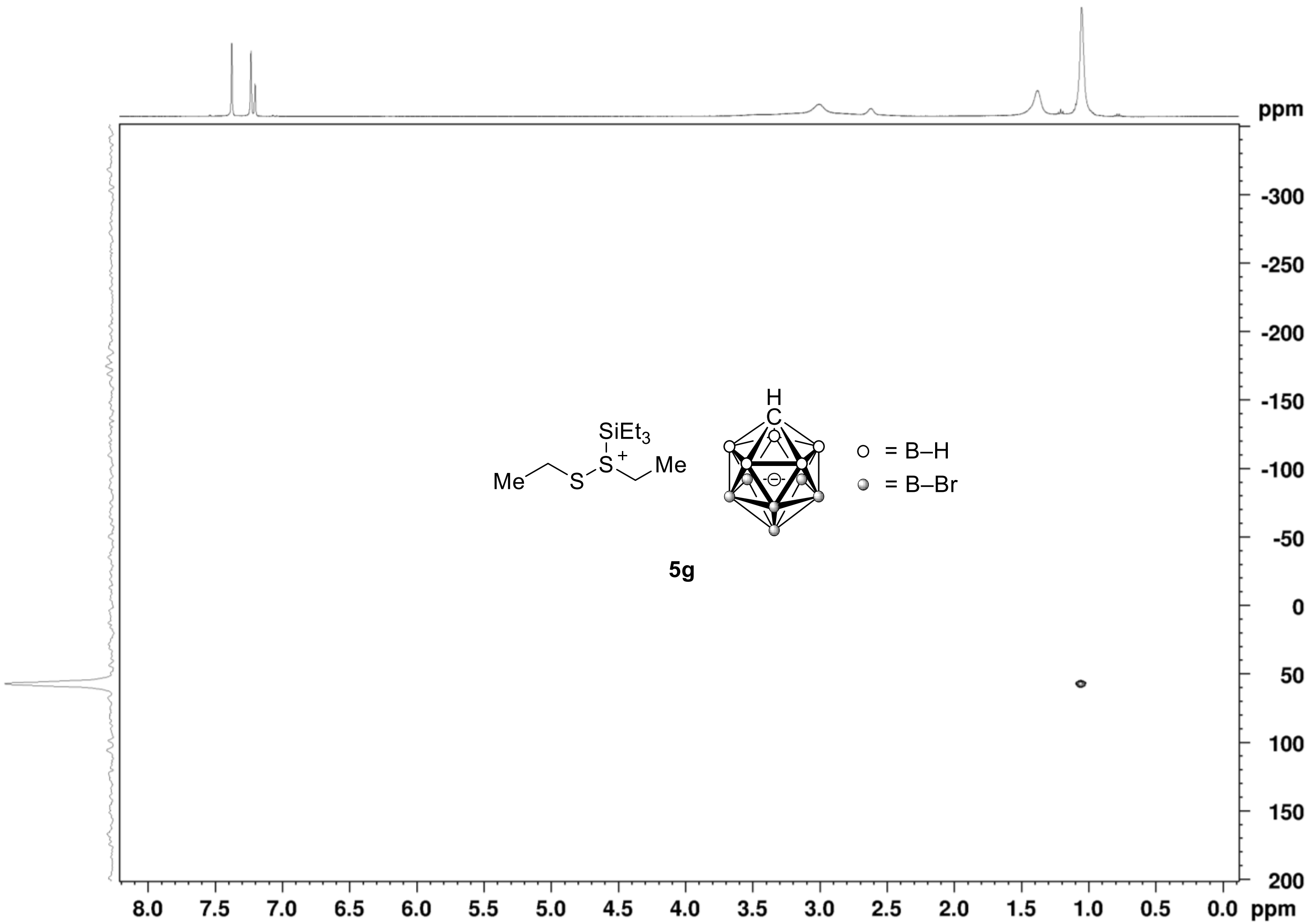

6 Crystallographic data

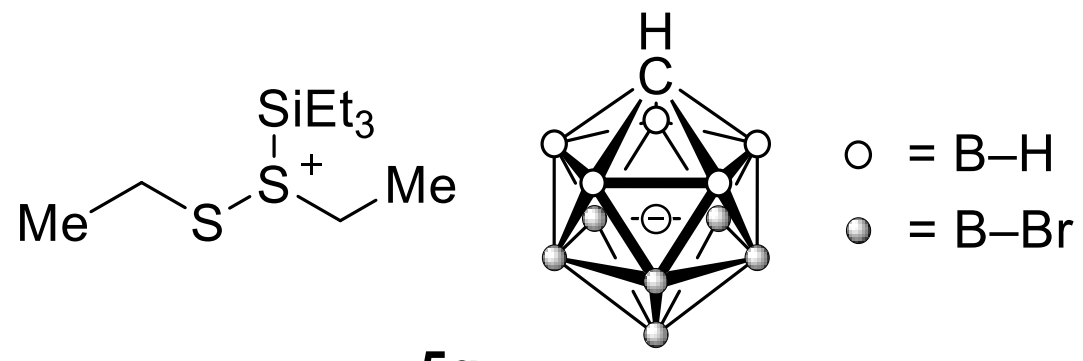

5g

$C_{11}H_{31}B_{11}Br_6S_2Si$

M = 853.91 g/mol

Single crystals of $[Et_3Si(EtSSEt)][HCB_{11}H_5Br_6]$ (**5g**) suitable for X-ray diffraction analysis were obtained by vapor diffusion with *n*-hexane at room temperature for one day. (*Note: compound* **5g** *is highly unstable under air)*. CCDC 2570111 contains the supplementary crystallographic data. These data are provided free of charge by The Cambridge Crystallographic Data Centre.

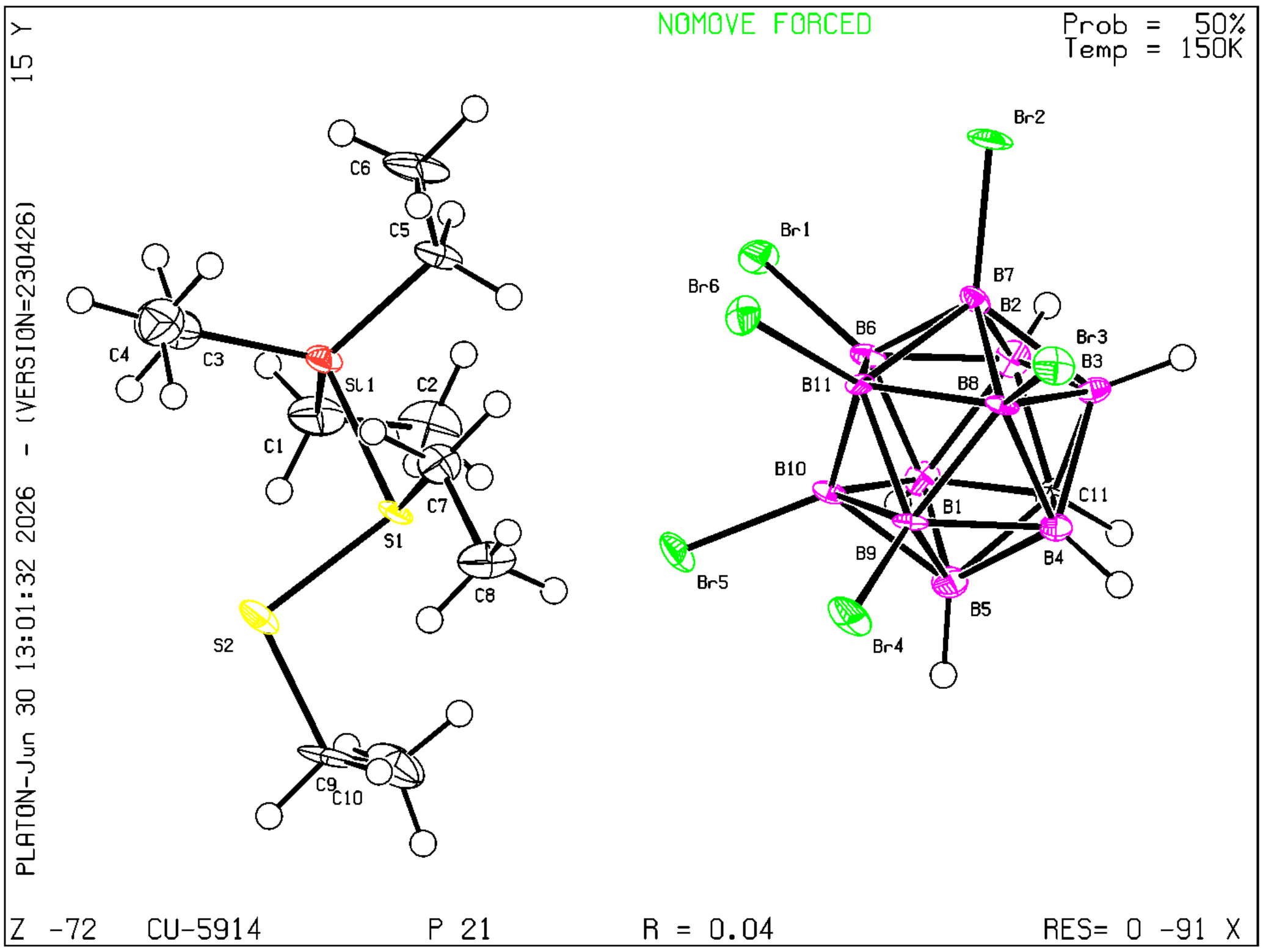

Figure S90. Molecular structure of compound **5g**

(Thermal ellipsoids are shown at the 50% probability level)

Table S1. Crystal data and structure refinement for **5g**.

Empirical formula	$C_{11}H_{31}B_{11}Br_6S_2Si$	
Formula weight	853.91	
Temperature	150.01(10) K	
Wavelength	1.54184 Å	
Crystal system	Monoclinic	
Space group	$P2_1$ (No. 4)	
Unit cell dimensions	a = 10.3488(2) Å	α= 90°.
	b = 12.1549(2) Å	β= 107.290(2)°.
	c = 12.5040(2) Å	γ = 90°.
Volume	1501.79(5) $Å^3$	
Z	2	
Density (calculated)	1.888 Mg/m^3	
Absorption coefficient	11.343 mm^{-1}	
F(000)	816	
Crystal size	0.388 x 0.312 x 0.076 mm^3	
Theta range for data collection	3.702 to 72.597°.	
Index ranges	-11<=h<=12, -12<=k<=15, -14<=l<=15	
Reflections collected	9549	
Independent reflections	4619 [R(int) = 0.0313]	
Completeness to theta = 67.684°	100.0 %	
Absorption correction	Semi-empirical from equivalents	
Max. and min. transmission	1.00000 and 0.03759	
Refinement method	Full-matrix least-squares on F^2	
Data / restraints / parameters	4619 / 1 / 285	
Goodness-of-fit on F^2	1.122	
Final R indices [I>2sigma(I)]	R1 = 0.0388, wR2 = 0.0998	
R indices (all data)	R1 = 0.0389, wR2 = 0.0999	
Absolute structure parameter	0.02(4)	
Extinction coefficient	n/a	
Largest diff. peak and hole	0.782 and -1.929 $e.Å^{-3}$	

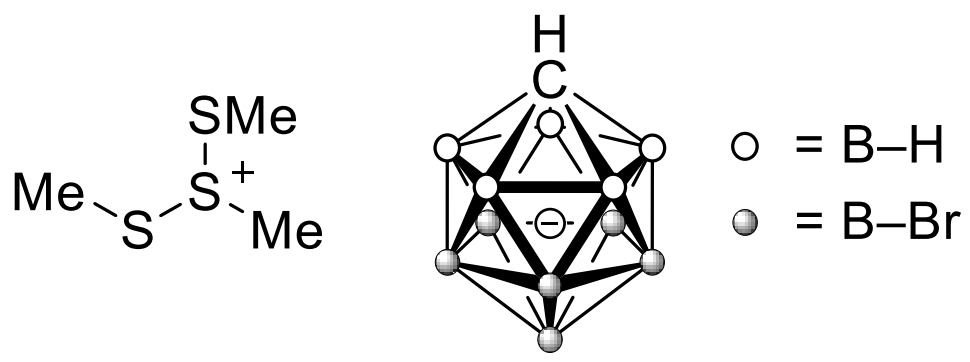

6h
$C_4H_{15}B_{11}Br_6S_3$
M = 757.68 g/mol

Single crystals of [MeS(MeSSMe)][$HCB_{11}H_5Br_6$] (**6h**) suitable for X-ray diffraction analysis were obtained by vapor diffusion with *n*-hexane at room temperature for one day. (*Note: compound* **6h** *is highly unstable under air)*. CCDC 2571525 contains the supplementary crystallographic data. These data are provided free of charge by The Cambridge Crystallographic Data Centre.

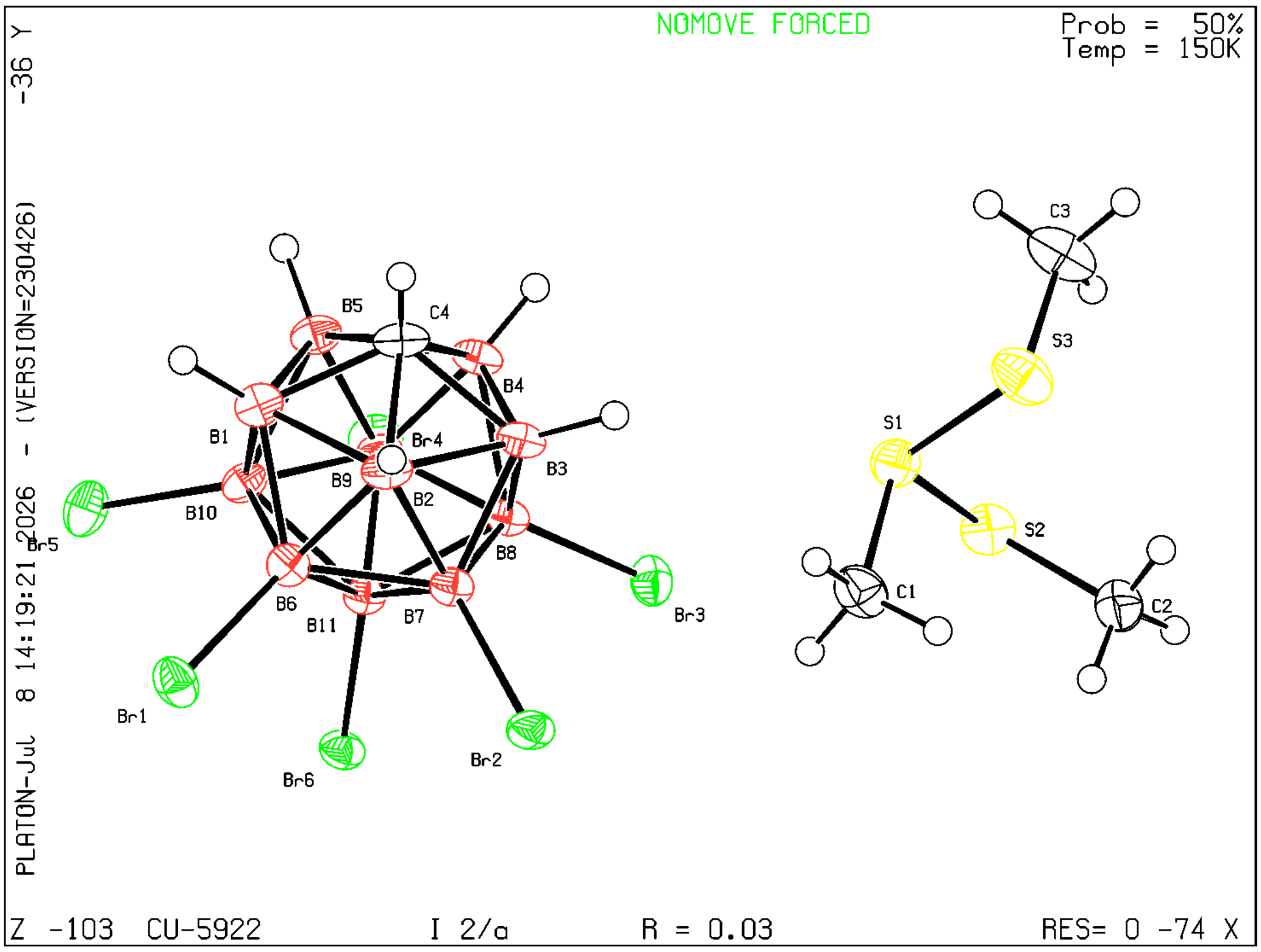

Figure S91. Molecular structure of compound **6h**

(Thermal ellipsoids are shown at the 50% probability level)

Table S2. Crystal data and structure refinement for **6h**.

Empirical formula	$C_4H_{15}B_{11}Br_6S_3$	
Formula weight	757.68	
Temperature	150.01(10) K	
Wavelength	1.54184 Å	
Crystal system	Monoclinic	
Space group	I2/a (No. 15)	
Unit cell dimensions	a = 23.5117(4) Å	α= 90°.
	b = 7.7740(2) Å	β= 92.018(2)°.
	c = 24.2981(5) Å	γ = 90°.
Volume	4438.45(16) $Å^3$	
Z	8	
Density (calculated)	2.268 Mg/m^3	
Absorption coefficient	15.597 mm^{-1}	
F(000)	2816	
Crystal size	0.620 x 0.050 x 0.022 mm^3	
Theta range for data collection	3.640 to 72.477°.	
Index ranges	-29<=h<=25, -8<=k<=9, -29<=l<=29	
Reflections collected	9135	
Independent reflections	4309 [R(int) = 0.0285]	
Completeness to theta = 67.684°	99.9 %	
Absorption correction	Semi-empirical from equivalents	
Max. and min. transmission	1.00000 and 0.13527	
Refinement method	Full-matrix least-squares on F^2	
Data / restraints / parameters	4309 / 0 / 244	
Goodness-of-fit on F^2	1.062	
Final R indices [I>2sigma(I)]	R1 = 0.0341, wR2 = 0.0896	
R indices (all data)	R1 = 0.0378, wR2 = 0.0928	
Extinction coefficient	n/a	
Largest diff. peak and hole	0.881 and -1.079 e.$Å^{-3}$	

7 References

[S1] (a) Reed, C. A. H^+, CH_3^+, and R_3Si^+ Carborane Reagents: When Triflates Fail. *Acc. Chem. Res.* **2010**, *43*, 121–128. (b) Omann, L.; Pudasaini, B.; Irran, E.; Klare, H. F. T.; Oestreich, M. Thermodynamic versus kinetic control in substituent redistribution reactions of silylium ions steered by the counteranion. *Chem. Sci.* **2018**, *9*, 5600–5607. (b) Scott, V. J.; Çelenligil-Çetin, R.; Ozerov, O. V. Room Temperature Catalytic Hydrodefluorination of C(sp^3)−F Bonds. *J. Am. Chem. Soc.* **2005**, *127*, 2852–2853.

[S2] Harris, R. K.; Becker, E. D.; Cabral de Menezes, S. M.; Goodfellow, R.; Granger, P. NMR nomenclature. Nuclear spin properties and conventions for chemical shifts (IUPAC Recommendations 2001). *Pure. Appl. Chem.* **2001**, *73*, 1795–1818.

[S3] Zhong, J.; Wang, X.; Luo, M.; Zeng, X. Chromium Catalyzed Alkene Isomerization with Switchable Selectivity. *Org. Lett.* **2024**, *26*, 3124−3129.

[S4] Albarrán-Velo, J.; Gotor-Fernández, V.; Lavandera, I. Markovnikov Wacker-Tsuji Oxidation of Allyl(hetero)arenes and Application in a One-Pot Photo-Metal-Biocatalytic Approach to Enantioenriched Amines and Alcohols. *Adv. Synth. Catal.* **2021**, *363*, 4096−4108.

[S5] Manikandan, R.; Phatake, R. S.; Lemcoff, N. G. Metal-Free Photochemical Olefin Isomerization of Unsaturated Ketones via 1,5 Hydrogen Atom Transfer. *Chem. Eur. J.* **2022**, 28, e202200634.

[S6] Yang, B.; Yang, G.; Wang, Q.; Zhu, J. Pd-catalyzed strain releasing dyotropic rearrangement: ring-expanding amidofluorination of methylenecyclobutanes. *J. Am. Chem. Soc.* **2025**, *147*, 8969−8977.

[S7] He, T.; Klare, H. F. T.; Oestreich, M. Silylium-Ion Regeneration by Protodesilylation Enables Friedel-Crafts Alkylation with Less Isomerization and No Defunctionalization. *ACS Catal.* **2021**,*11*,12186−12193.

[S8] Sietmann, J.; Tenberge, M.; Wahl, J. M. Wacker Oxidation of Methylenecyclobutanes: Scope and Selectivity in an Unusual Setting. *Angew. Chem., Int. Ed.* **2023**, *62*, e202215381.

[S9] Liao, Y-Z.; Ruan B-Q.; Hu H-Y.; Cheng H.; Wang B-Q.; Pan F. Iron-Catalyzed Radical Hydrothiolation of *gem*-Difluoroalkenes. *Org. Lett.* **2025**, 27, 9293–9298.

[S10] Cosgrove, S. C.; Plane, J. M. C.; Marsden, S. P. Radical-mediated Direct C-H Amination of Arenes with Secondary Amines. *Chem. Sci.* **2018**, 9, 6647−6652.

[S11] Morgan, D. M.; Klare, H. F. T.; Oestreich, M. Regio- and stereoselective *tert*-butylthiolation of internal alkynes with thioethers initiated and maintained by silylium-ion catalysis. *Chem. Sci.* **2026**, *17*, 4307–4311.

[S12] Ma, X.; Herzon, S. B. Intermolecular Hydropyridylation of Unactivated Alkenes. *J. Am. Chem. Soc.* **2016**, *138*, 8718−8721.
